# *CUL4A* contributes to the biology of basal-like breast tumors through modulation of cell growth and antitumor immune response

**DOI:** 10.18632/oncotarget.1915

**Published:** 2014-04-18

**Authors:** Laura P. Saucedo-Cuevas, Isabel Ruppen, Pilar Ximénez-Embún, Samuel Domingo, Javier Gayarre, Javier Muñoz, Jose M. Silva, María J. García, Javier Benítez

**Affiliations:** ^1^ Group of Human Genetics, Human Cancer Genetics Program, Spanish National Cancer Research Centre (CNIO), Spain; ^2^ ProteoRed-ISCIII, Proteomics Core Unit, Biotechnology Program, Spanish National Cancer Research Centre (CNIO), Spain; ^3^ Institute for Cancer Genetics, Columbia University, New York, NY, USA; ^4^ Spanish Network Research on Rare Diseases (CIBERER), Spain

**Keywords:** CUL4A, 13q34 amplification, breast cancer, basal-like, proteomics, immune system

## Abstract

The *CUL4A* E3 ubiquitin ligase is involved in the regulation of many cellular processes and its amplification and/or overexpression has been observed in breast cancer. The 13q34 amplification, which is associated with the basal-like breast cancer subtype, has been proposed as one of the mechanism behind *CUL4A* up-regulation. However, the specific contribution of *CUL4A* to the biology of basal-like breast tumors has not yet been elucidated. In this work, by using cellular models of basal phenotype, we show the inhibitory effect of *CUL4A* silencing in the proliferation and growth of breast cancer cells both, *in vitro* and *in vivo.* We also demonstrate the transforming capacity of *CUL4A* exogenous overexpression in the 184B5 human mammary epithelial cells *in vitro*. Our results suggest a synergistic effect between *CUL4A* high levels and the activation of the *RAS* pathway in the tumorigenesis of basal-like breast cancer tumors. In addition, by using a proteomics approach we have defined novel candidate proteins and pathways that might mediate the oncogenic effect of *CUL4A.* In particular, we report a putative role of *CUL4A* in bypassing the immune system in breast cancer through the down-regulation of several molecules involved in the immune surveillance. These findings provide insight into the oncogenic properties of *CUL4A* in basal-like breast cancer and highlight the therapeutic opportunities to target *CUL4A*.

## INTRODUCTION

The *CUL4A* E3 ubiquitin ligase belongs to the family of cullin proteins and is a component of the cullin-RING ligase 4A complex [1]. This complex plays a crucial role in the regulation of both stability and degradation of a huge spectrum of cellular proteins through the ubiquitin-proteasome system [1, 2]. *CUL4A* confers substrate specificity and it is involved in the control of cell cycle, DNA replication and DNA repair depending on the nature of ubiquitinated proteins [3-5]. *CUL4A* has been found amplified and/or overexpressed in different types of carcinomas and a putative oncogenic role has been proposed for this gene [6-9]. Breast cancer patients with strong expression of *CUL4* show shorter overall and disease-free survival [6, 10] which has led to the proposal that *CUL4A* might confer an aggressive behavior in this malignancy. The functional *in vitro* and *in vivo* characterization of the putative oncogenic role of *CUL4A* has been previously studied in prostate cancer and mesothelioma [7, 8]. In breast cancer, a recent functional analysis of *CUL4A* has revealed a pivotal role of the gene in regulating the metastatic behavior of breast cancer cells [11].

In previous studies we showed that the overexpression of *CUL4A* was associated with markers of proliferation and tumor aggressiveness in familiar and sporadic primary breast tumors [12]. In addition we found that, although not exclusively, the 13q34 amplification would be one of the mechanisms triggering *CUL4A* overexpression in breast cancer. Importantly, we observed that the 13q34 amplification presents increased frequency in BRCA1-defective tumors and seems to be associated with primary breast tumors of basal-like phenotype [12, 13]. Since basal-like breast cancers are characterized by lack of effective targeted therapy and poor clinical outcome [14], the elucidation of the specific role of *CUL4A* in the carcinogenic process of this tumor subtype is of major interest.

In this study we have evaluated *in vitro* and *in vivo* the functional significance of *CUL4A* down-regulation and up-regulation in basal-like breast cancer models that exhibit features resembling those found in primary breast tumors. Our results support the role of *CUL4A* as a driver oncogene at the 13q34 amplification in basal-like breast cancer. Also, suggest an implication of *CUL4A* overexpression in cancer aggressiveness and progression rather than a major function as a transformation-initiating event. In addition, our proteomics-derived data provide evidence for novel molecules and pathways that might be involved in mediating the tumorigenic process orchestrated by *CUL4A.*

## RESULTS

### *CUL4A* is overexpressed in breast cancer cell lines

**Figure 1:**
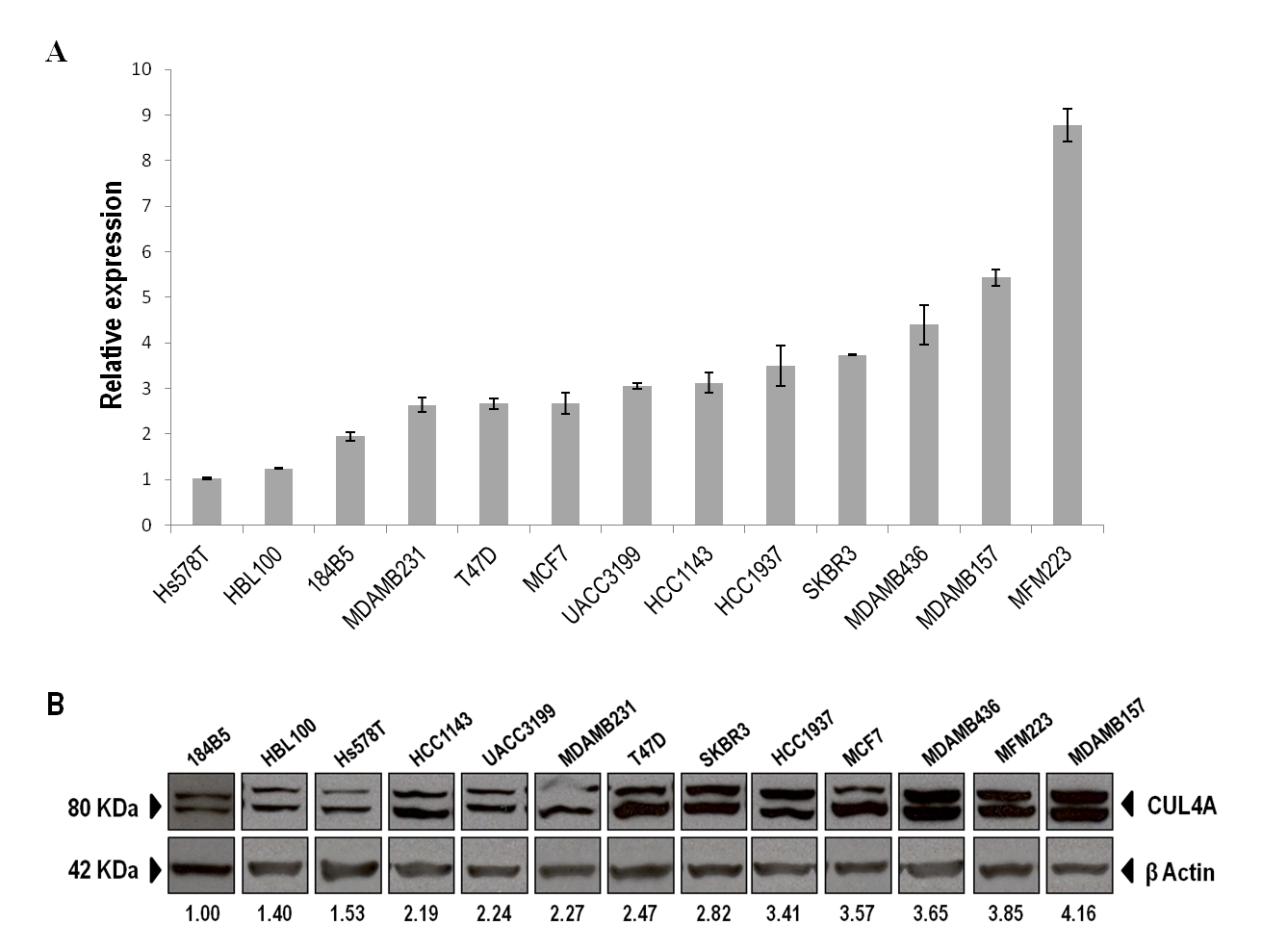
*CUL4A* expression in the panel of human breast epithelial cell lines (A) *CUL4A* mRNA expression was evaluated by qRT-PCR in the panel of breast cell lines and normalized to β-actin expression. Cell lines are shown from low to high *CUL4A* expression. Bars represent median and standard deviation (SD) of three technical replicates. (B) CUL4A protein levels were assessed by Western blot. Image of WB was cropped to organize cell lines from low to high CUL4A protein levels. The anti-CUL4A antibody recognizes a doublet of CUL4A. The upper band corresponds to the neddylated form of the protein. Numbers under the image correspond to intensity of CUL4A bands (neddylated and non-neddylated forms) for each cell line normalized to β-ACTIN intensity value and relative to the lowest CUL4A-expressing cells (184B5).

We examined the expression of *CUL4A* in 11 human breast cancer cell lines and two non-transformed human mammary epithelial cell lines by quantitative PCR (qPCR) and Western blot (WB). In both analysis the non-transformed cells (HBL100 and 184B5) were within the lower relative expression levels indicating that *CUL4A* is overall up-regulated in breast cancer cell lines compared with normal mammary epithelial cells (Figure 1A and B). *CUL4A* expression varied across the breast cancer cell lines. MDAMB157 with extra DNA copies at 13q34 was found among those cell lines exhibiting the highest *CUL4A* relative expression values. Also, HCC1937 and MDAMB436 cells, with *BRCA1*-impairment, were found in the upper limit of the expression range. With few exceptions cell lines ordered according to their *CUL4A* relative expression by qPCR matched the order obtained by WB (Figure 1A and B), with both data set exhibiting a high correlation (*ρ*=0.714, *P=*0.004). Since the 13q34 amplification and/or *CUL4A* overexpression is more frequent in *BRCA1*-associated and basal-like primary breast tumors [12] we selected the *BRCA1*-null HCC1937 and the 13q34 amplified MDAMB157 cell lines to further characterize *CUL4A* function in breast cancer progression. Both cell lines are of basal-like subtype [15, 16] and as shown by our experiments express high levels of *CUL4A*. In addition, we included the basal-type and low *CUL4A*-expressing 184B5 mammary epithelial cells as a non-tumorigenic model to assess the implication of *CUL4A* overexpression in the initial steps of the transformation process.

### *CUL4A* down-regulation inhibits breast cancer tumorigenesis *in vitro* and *in vivo*

**Figure 2:**
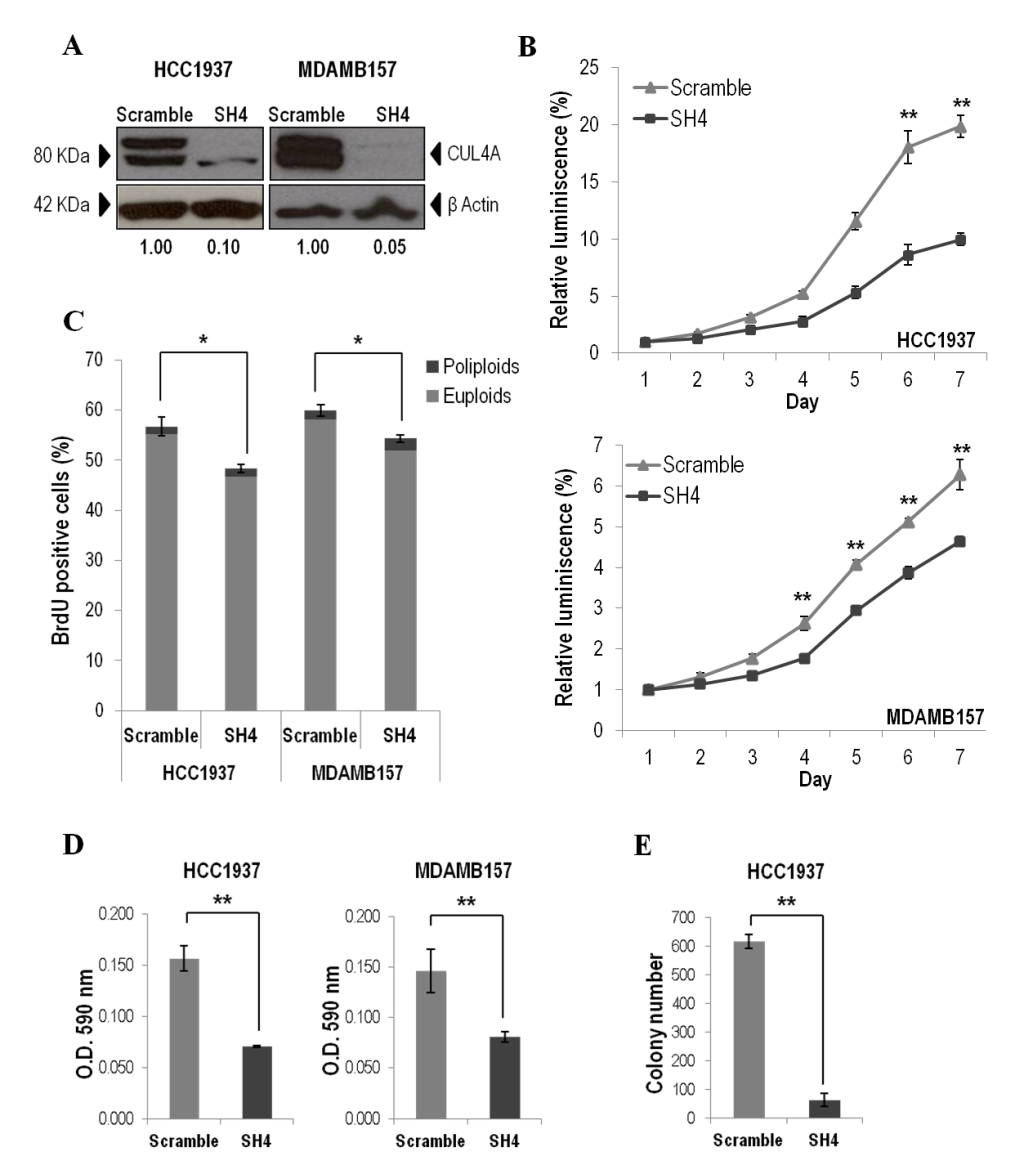
*CUL4A* down-regulation in HCC1937 and MDAMB157 breast cancer cell lines (A) Western blot analysis of CUL4A expression in cells transduced with *CUL4A* specific shRNA (SH4) and control shRNA (Scramble). Numbers under the image correspond to intensity of CUL4A bands (neddylated and non-neddylated forms) for each cell line normalized to β-ACTIN intensity value. (B) Viability of *CUL4A*-silenced (SH4) and Scrambled cells. Cells were seeded in 96-well plates at 1500 cells/well and incubated for indicated time. The quantity of viable cells was determined by CellTiter-Glow assay. Each point represents mean and standard deviation (SD) of 6 replicates. Experiments were carried out 3 times and showed similar results. (C) Percentage of BrdU positive cells in *CUL4A*-knocked down (SH4) and Scrambled cells. Bars represent the means of triplicate experiments and error bars indicate the SD. Light and dark grey illustrates the partial contribution of euploids and poliploids cells, respectively, to the total counting. (D) *CUL4A-*depleted breast cancer cell lines (SH4) and control cells (Scramble) were grown in anchorage-dependent conditions. Cells were fixed and stained with crystal violet. Crystal violet was solubilized and readouts (optical density at 590 nm) were obtained. Representative example of three-independent experiments is shown. Data are expressed as mean ± SD of three replicates. (E) *CUL4A*-knocked down (SH4) HCC1937 and control cells (Scramble) were grown in soft agar to evaluate anchorage-independent growth ability. Graphics represents the average number of colonies ± SD. **P<0.01, *P<0.05.

**Figure 3:**
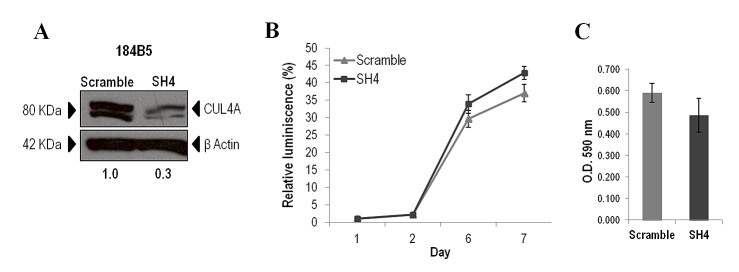
*CUL4A* down-regulation in the non-transformed 184B5 mammary epithelial cells (A) Western blot analysis of CUL4A expression in 184B5 cells transduced with *CUL4A* specific shRNA (SH4) and control shRNA (Scramble). Numbers under the image correspond to intensity of CUL4A bands (neddylated and non-neddylated forms) for each cell line normalized to β-ACTIN intensity value. (B) Viability of *CUL4A*-silenced (SH4) and Scrambled cells (Scramble). Cells were seeded in 96-well plates at 1500 cells/well and incubated for indicated time. The quantity of viable cells was determined by CellTiter-Glow assay. Each point represents mean and standard deviation (SD) of 6 replicates. Experiments were carried out 3 times and showed similar results. (C) *CUL4A*-depleted 184B5 cells (SH4) and control cells (Scramble) were grown in anchorage-dependent conditions. Cells were fixed and stained with crystal violet. Then crystal violet was solubilized and readouts (optical density at 590 nm) were obtained. Representative example of three-independent experiments is shown. Data are expressed as mean ± SD of three replicates.

**Figure 4:**
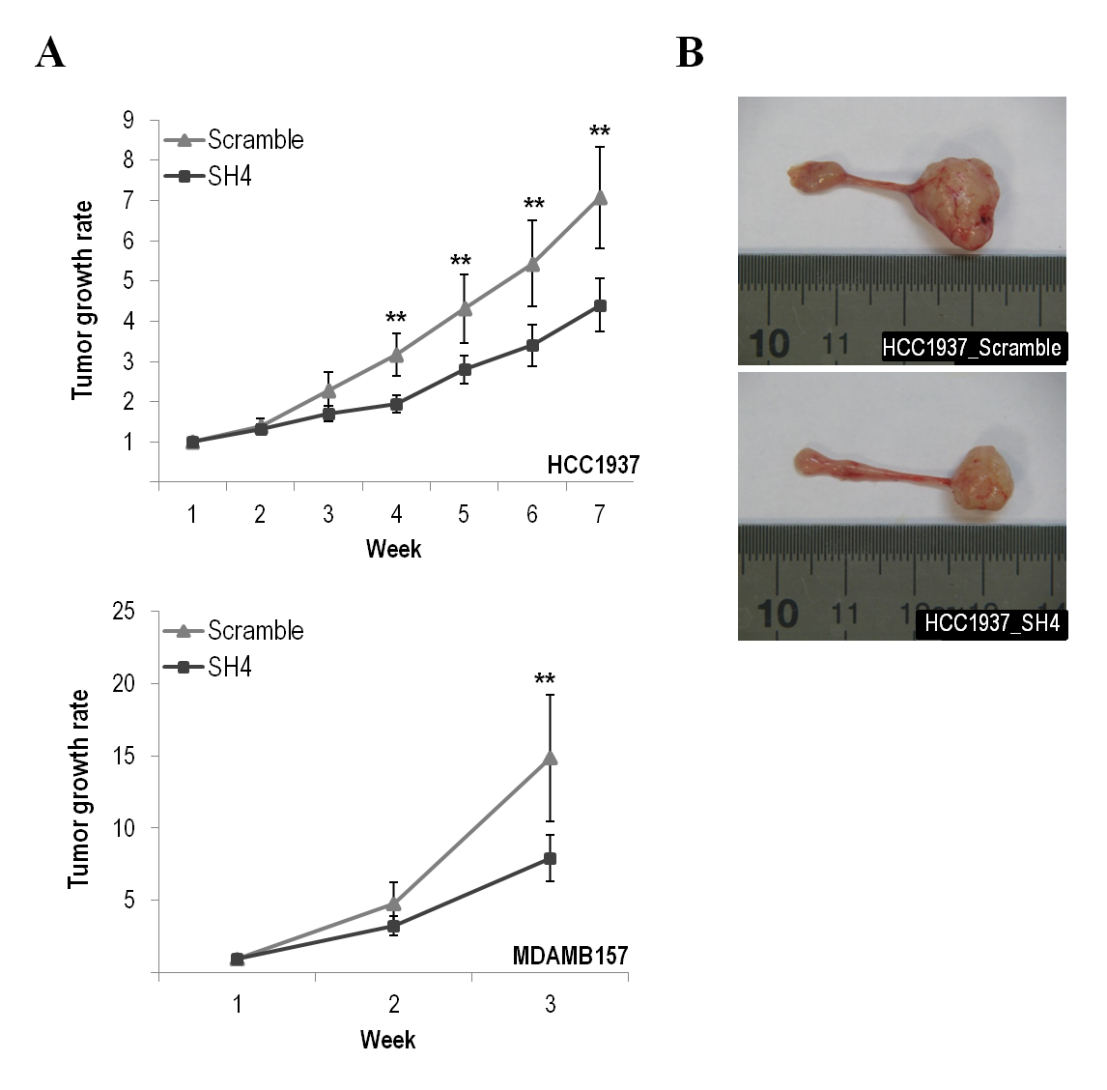
*CUL4A* down-regulation effect in the tumor growth abilities of the breast cancer cell lines (A) *CUL4A*-silenced (SH4) and control cells (Scramble) were orthotopically injected into the mammary glands of nude mice and the tumor onset and growth were evaluated at the indicated time points. Error bars represent 95% confidence intervals. (B) Representative images showing the tumor size formed by HCC1937-silenced and HCC1937-scramble cells. **P<0.01.

To directly assess the contribution of endogenous *CUL4A* overexpression on the transformed phenotype of human breast cancer cells, we examined the effects of knocking down *CUL4A* in the HCC1937 and MDAMB157 breast cancer cell lines where *CUL4A* is amplified and/or overexpressed. Cells transduced with a *CUL4A-*specific shRNA construct (SH4) (see Methods) presented reduced *CUL4A* protein levels by at least 90% compared with control cells (transduced with scrambled vector) (Figure 2A). The *CUL4A*-knocked-down cells showed decreased proliferation compared with control cells in viability assays (Figure 2B). In agreement, a reduced S-phase population was observed in both *CUL4A* knocked-down cells compared with controls (Figure 2C). In addition, HCC1937- and MDAMB157-*CUL4A*-silenced cells showed reduced ability to form colonies in anchorage-dependent conditions relative to controls (Figure 2D). Proliferation was also inhibited in *CUL4A*-knocked-down HCC1937 cells when grown in anchorage-independent conditions (Figure 2E). Modifications in anchorage-independent growth could not be evaluated in the MDAMB157 model as these cells do not grow in soft-agar. The 184B5-*CUL4A*-silenced cells (Figure 3A) did not show significant changes in proliferation (Figure 3B) and colony formation abilities (Figure 3C). This would be consistent with a specific dependence of the *CUL4A* function acquired in *CUL4A-*overexpressing cancer cells. In agreement with our *in vitro* results, orthotopical inoculation of *CUL4A*-silenced cells in immunodeficient mice generated tumors with significantly slower growth rate than that of Scramble-derived tumors (Figure 4A). At the end point of these in vivo experiments the average volume of tumors induced by *CUL4A*-silenced cells was half the average volume exhibited by tumors produced by control cells (Figure 4B).

### *CUL4A* overexpression promotes *in vitro* transformation of human mammary epithelial cells

**Figure 5:**
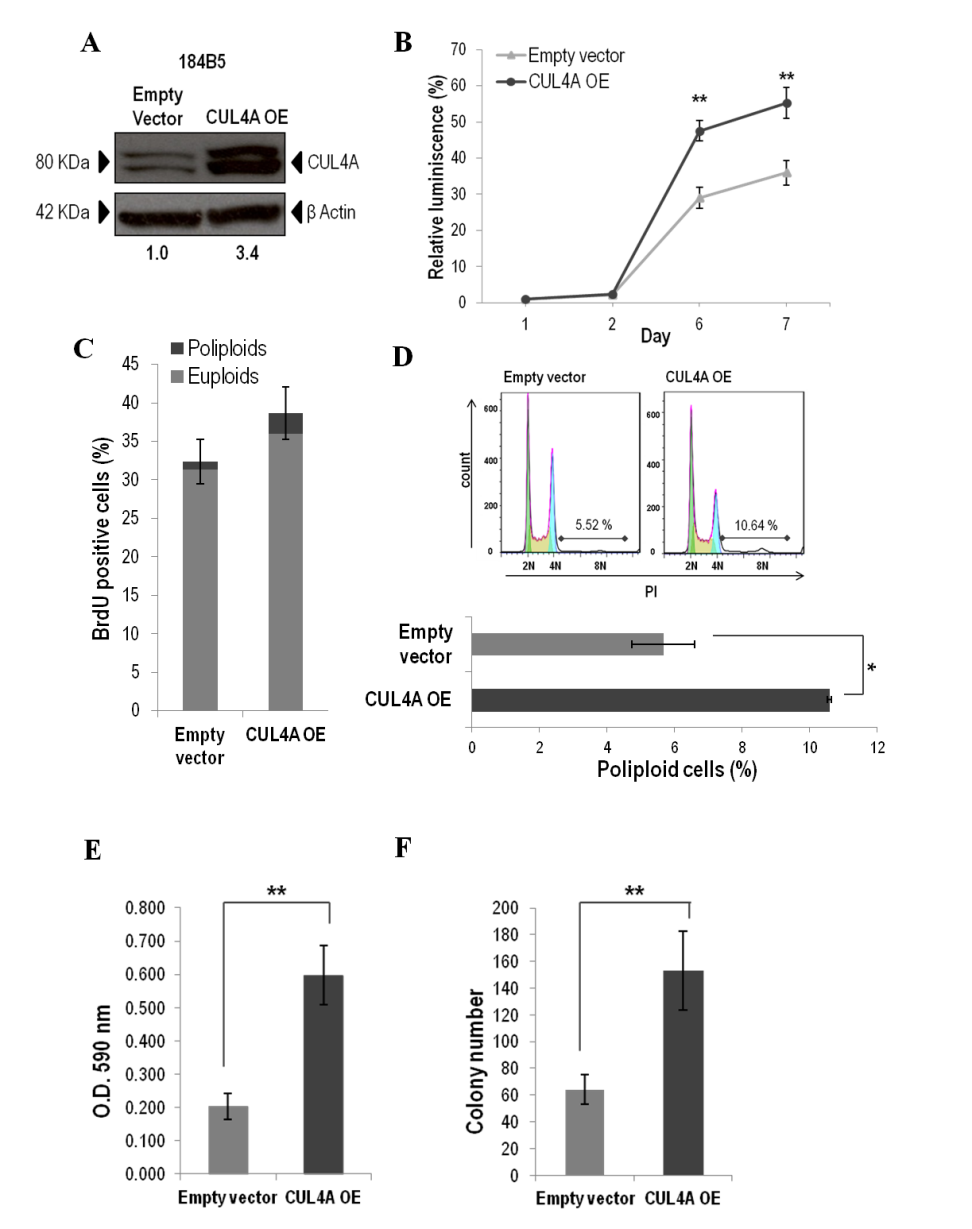
*CUL4A* overexpression in the 184B5 human mammary epithelial cells (A) Western blot analysis of CUL4A expression in *CUL4A*-overexpressing cells (OE) and cells infected with the empty vector. Numbers under the image correspond to intensity of CUL4A bands (neddylated and non-neddylated forms) for each cell line normalized to β-ACTIN intensity value. (B) Viability of *CUL4A*-overexpressing and control cells. Cells at a concentration of 1500 cells were seeded in a 96-well plate and incubated for indicated time. The quantity of viable cells was determined by CellTiter-Glow assay. Each point represents mean and standard deviation (SD) of 6 replicates. Experiments were carried out 3 times and showed similar results. (C) Percentage of BrdU positive cells in *CUL4A*-overexpressing and control cells. Bars represent the mean of triplicate experiments and error bars indicate the SD. Light and dark grey illustrates the partial contribution of euploids and poliploids cells, respectively, to the total counting. (D) Cell cycle distribution of *CUL4A*-overexpressing and control cells. Upper, representative graphs. (E) *CUL4A*-overexpressing and control cells were grown in anchorage-dependent conditions. Cells were fixed and stained with crystal violet. Then crystal violet was solubilized and readouts (optical density at 590 nm) were obtained. Representative example of three-independent experiments is shown. Data are expressed as mean ± SD of three replicates. (F) *CUL4A*-overexpressing and control cells were grown in soft agar to evaluate anchorage-independent growth ability Graphics represents the average number of colonies ± SD. **P<0.01, *P<0.05.

To elucidate the role played by *CUL4A* in the initial steps of the carcinogenic process the 184B5 immortalized non-transformed human mammary breast cells were transduced with a *CUL4A* expression vector. Cells with exogenous *CUL4A* expression showed effective increase in CUL4A protein levels compared with cells infected with empty vector (Figure 5A) and showed increased viability with respect to control cells in cell-based luminescence assays (Figure 5B). Although *CUL4A* overexpression also induced an S-phase population increase relative to control (Figure 5C) differences did not reach statistically significance. However, when we specifically compared the subpopulation of polyploid cells we found significantly higher BrdU incorporation in the *CUL4A-*overexpressing cells compared to control cells (3-fold increase, *P<*0.05). These results suggest that *CUL4A* overexpression might enhance proliferation through promotion of S-phase entry. In particular, significant increase of poliploid cells in S-phase suggests that *CUL4A* expression might contribute to tetraploid checkpoint failure mostly leading to propagation of poliploid cells. Consistently, cell cycle analysis revealed that 184B5 cells transduced with the *CUL4A* vector exhibited a significant increase of polyploid cells compared to those transduced with the empty vector (Figure 5D). We next performed colony formation assays and observed a significant enhancement of the capability of the *CUL4A* up-regulated cells to form colonies both, in anchorage-dependent (Figure 5E) and anchorage-independent conditions (Figure 5F). Altogether these results support the *CUL4A* contribution to malignant transformation of normal breast epithelial cells. Despite these *in vitro* evidences overexpression of *CUL4A* did not generate 184B5-derived tumors in orthotopically xenografted mice.

### *CUL4A* overexpression collaborates with *H-Ras* in the transformation of 184B5 cells

In order to continue deciphering *CUL4A* role in the transformation of human mammary epithelial cells we evaluated *CUL4A* cooperation with known oncogenes. To this end by using a retroviral system we further modified the CUL4A-overexpressing and control (transduced with the empty vector) 184B5 cells to stably express *c-Myc* or the mutant active *H-Ras (H-Ras-V12)*.

**Figure 6:**
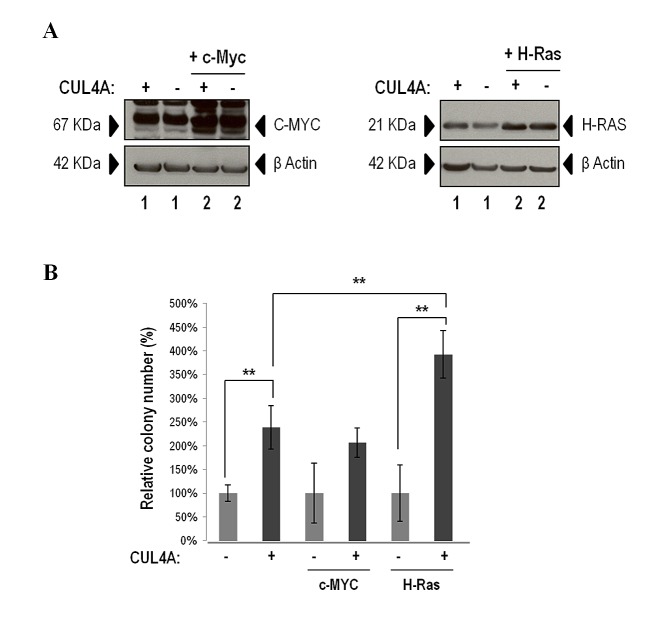
*CUL4A* cooperation experiment (A) Western blot analysis of C-MYC (left) and H-RAS (right) ectopic expression in CUL4A-overexpressing and control cells. Numbers under the image correspond to intensity of C-MYC or H-RAS bands for each cell line normalized to β-ACTIN intensity. (B) *CUL4A*-overexpresing and control cells transduced with *c-Myc* and *H-Ras-V12* were seeded in soft agar, stained with MTT and counted. Values correspond to mean number of colonies ± standard deviation (SD) of three independent replicates. Data show percentage of colonies relative to control. **P<0.01. (+) *CUL4A* overexpression; (-) Empty vector.

Overexpression of *c-Myc* or *H-Ras-V12* was confirmed by western blot (Figure 6A) and transforming capacities were evaluated by growing cells in soft agar. Cells with exogenous expression of *H-Ras-V12* that concomitantly overexpressed *CUL4A* presented a statistically significant increase in the number of colonies compared with control cells (transduced with empty vector and *H-Ras-V12*) (Figure 6B). Cells with ectopic expression of *c-Myc* that in addition overexpressed *CUL4A* also presented higher number of colonies than control cells (cells transduced with empty vector and *c-Myc*) however, differences did not reach significance (Figure 6B). The magnitude of the relative increment in colony number in 184B5 cells that co-expressed *H-Ras-V12* and *CUL4A* was significantly larger (4 fold versus control) than the effect induced in cells that overexpressed *CUL4A* alone (2 fold versus control) (Figure 6B).

### Proteome expression profiling analysis reveals several mediators of CUL4A activity

**Figure 7:**
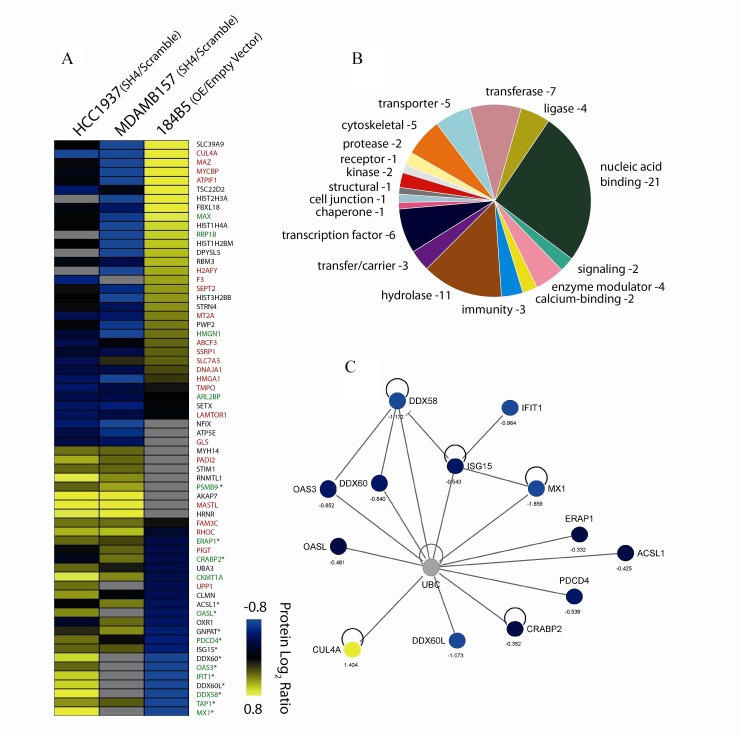
Differentially regulated proteins (A) Candidate proteins modulated by *CUL4A* expression identified by quantitative proteomics in the silencing (HCC1937, MDAMB157) and overexpression (184B5) cell line models. Protein log_2_ ratios are represented by colors, where “blue” means differentially down-regulated protein and “yellow”, differentially up-regulated protein. Grey and black colors refer to not found or not modified proteins, respectively. Protein names in red indicate collaboration with the oncogenic process, green involvement in tumor suppressor activities, and (*) implication in the immune system, as described in the literature. Detailed information such as accession number, gene symbol, averaged ratio, and full protein names for these proteins are listed in Supplementary Table S4. (B) Classification of candidate proteins based on their molecular functional annotations using gene ontology (GO). (C) Network generated by IPA including the immune system-related proteins and CUL4A. Color code for differential regulation as described in (A).

To better understand the molecular consequences associated with the modulation of *CUL4A* expression, we examined changes in protein expression of our *CUL4A*-modified cellular models. To this end, *CUL4A*-silenced and overexpressed cells and their corresponding controls (cells transduced with scrambled shRNA and empty vector, respectively) were analyzed using a quantitative proteomic approach. We confidently identified and quantified 5,087 proteins (Supplementary Table S4) from which 764 showed differential expression in at least one of the three experiments conducted (Supplementary Figure S1) (see Materials and Methods). As expected, and technically validating our approach, CUL4A was one of the top-ranking differentially expressed proteins being down-regulated in silenced cells and up-regulated in overexpressed cells (Figure 7A). We then defined a subset of 64 differentially expressed proteins that showed opposite behavior between the *CUL4A-*overexpressing cells and at least one of the *CUL4A* knocked down models. Proteins that did not show regulation or with no available data in the *CUL4A*-overexpressing cells but that showed same behavior in both *CUL4A* silenced cells were also included in the list. Among the 64 differentially expressed proteins we found a large variety of protein classes (Figure 7B) including nucleic acid binding proteins (e.g. HMGA1, PDCD4, MAX), hydrolases (e.g. DDX60, SETX, MX1) and transporters (e.g. TAP1, ABCF3, SLC39A9).

To get further insights into the functional roles of such proteomic changes, we performed enrichment analysis of the 64 proteins by using Ingenuity Pathway Analysis (IPA) which showed a significant over-representation of functions such as “cell cycle”, “cellular growth and proliferation”, “cellular movement” or “antigen presentation” and of canonical pathways such as “interferon signaling” (Benjamini-Hochberg test, *P<*0.05). Interestingly, several known or putative oncogenes showed up-regulation upon *CUL4A* overexpression in the 184B5 cells and exhibited down-regulation in the *CUL4A*-silenced models (Figure 7A). Among them, SSRP1 and SEPT2 that participate in cell cycle progression [17, 18] or MYCBP, HMGA1 and MT2A involved in cell growth and proliferation and previously linked to tumor progression [19-21]. Also, replication-dependent histones such as HIST1H4A and HIST2H3A were up-regulated in CUL4A-overexpressing cells and down-regulated in at least one of the silencing models. Conversely, a number of putative or known tumor suppressors were down-regulated in *CUL4A*-overexpressing cells and/or overexpressed in *CUL4A* knocked down cells (Figure 7A). Among them, the *Programmed cell death protein 4* (PDCD4) that blocks cell cycle progression and cell proliferation and the anti-tumoral activity-related protein CRABP2 that is involved in cellular growth inhibition [22, 23]. In addition a set of 15 proteins that participate in the antitumor immune response such as the tumor suppressors PSMB9, ERAP1 and TAP1 or the interferon-stimulated genes IFIT1 and MX1 were found down-regulated in *CUL4A*-overexpresing cells and up-regulated in *CUL4A*-silencing models (Figure 7A). Given the novelty of a possible relation between the CUL4A E3 ubiquitin ligase and immune response modulation in breast cancer we interrogated IPA to specifically evaluate the potential link between the ubiquitin degradation system and the differentially expressed immune system-related proteins from our list. Remarkably, IPA generated a protein network showing multiple connections between this set of proteins and ubiquitin, which would be consistent with a putative role of CUL4A as modulator of immune system processes (Figure 7C).

## DISCUSSION

The overexpression of the *CUL4A* E3 ubiquitin ligase has been related to tumor aggressiveness and poor clinical outcome in breast cancer [6, 10, 24]. One of the mechanisms triggering *CUL4A* overexpression is the 13q34 amplification and this genomic aberration has been shown to be associated with breast tumors characterized by exhibiting *BRCA1* impairment or a basal-like phenotype [12, 13]. With these antecedents we were interested in studying the role of *CUL4A* in the carcinogenic process of the basal-like breast tumor subtype, which is aggressive in nature and lacks effective targeted therapies. Our results show that *CUL4A* would contribute to the tumorigenicity of basal-like breast cancers through the modulation of cell growth and anti-tumor immune response.

*CUL4A* silencing in *CUL4A*-overexpressing basal-like breast cancer cells induced a reduction of cell proliferation and colony formation in short-term assays and long-term soft agar assays and decreased the tumor growth rate of cells injected into mammary glands of nude mice. Importantly, we specifically demonstrated this effect in 13q34 amplification-bearing cells, which is biologically relevant given the association found between this amplification and primary breast tumors of basal-like phenotype [12, 13]. We previously found an increased frequency of the 13q34 amplification in BRCA1 tumors [12] and in this study we also demonstrate the growth inhibitory effect of *CUL4A* silencing in *BRCA1-*deficient cells. Altogether our results support the role of *CUL4A* as target gene of the 13q34 amplification. We provide evidence that, rather than being a passenger alteration derived from the increased genomic instability shown by basal-like and *BRCA1* primary breast tumors [14, 25], *CUL4A* overexpression would confer selective advantage to tumor cells.

This dependence or ‘addiction’ to *CUL4A* for maintenance of the malignant phenotype and cell survival of *CUL4A*-overexpressing cells would be similar to that observed in breast tumor cells that overexpress *ERBB2* or C-*MYC* [26, 27]. Hence, it may offer the opportunity to take advantage of *CUL4A* up-regulation in order to develop targeted therapies. In this regard our data suggest that the 13q34 amplification could be a robust biomarker for identification of up to 20% basal-like breast cancer patients [12, 13] that might eventually benefit from *CUL4A*-targeted therapy. Interestingly, in previous studies we observed increased sensitivity to trabectedin in breast cancer cell lines exhibiting high levels of *CUL4A* in combination with a “BRCAness” status [28]. The potential therapeutic option of *CUL4A-* targeting in certain subtypes of breast cancer has also been discussed in a recent study reporting a fundamental role of *CUL4A* in regulating the metastatic behavior of breast cancer cells [11]. However, the authors did not provide data on the effect of *CULA4* silencing in normal mammary cells. We showed that *CUL4A* silencing would not present detrimental consequences on the non-tumorigenic 184B5 mammary epithelial cells. These observations would be consistent with lack of *CUL4A* dependence in cells that do not overexpress the gene and might suggest low toxicity of eventual *CUL4A-*targeted inhibitory therapeutic approaches.

Although exogenous overexpression of *CUL4A* was sufficient to promote proliferation of 184B5 cells, *CUL4A* up-regulation alone was not enough to generate 184B5-derived tumors in xenografted mice. This suggests that *CUL4A* overexpression, rather than an initiating event, would constitute a secondary oncogenic hit acquired and selected during tumoral evolution. Our results support a potential synergistic effect between *CUL4A* overexpression and the constitutive activation of the RAS mitogenic signal in the transformation of human mammary epithelial cells, which is consistent with the high frequency of RAS pathway activation in basal-like breast tumors [29, 30].

To our knowledge, this is the first study that aims to define mediators of *CUL4A* transforming capacities in breast cancer using a proteomics approach. By using this methodology we propose novel molecules and pathways that might modulate the oncogenic effect of *CUL4A* in basal-like tumors. This can shed light on the understanding of the molecular events driven by *CUL4A* that may be explored in order to develop new therapeutic strategies.

Among the proteins that showed up-regulation upon induction of *CUL4A* exogenous expression were HMGA1, SSRP1 and MT2A. Interestingly, *HMGA1, SSRP1* and *MT2A* overexpression has been reported in breast cancer [19, 20, 31] and HMGA1 was previously described as a master regulator of the tumor progression in triple-negative breast cancer cells [32]. These molecules may be good candidates to develop targeted therapies in *CUL4A*-overexpressing tumors. In particular, *SSRP1* is reported to be a prospective target for anti-cancer small molecules [31, 33]. On the other hand, PDCD4 and CRABP2 protein levels were reduced with CUL4A overexpression. Consistently, low levels of PDCD4 in breast tumors have been associated with poor prognosis [34] and high CRABP2 levels have been shown to be indicative of longer overall survival in breast cancer patients [22].

For the first time, we report a putative role of *CUL4A* in bypassing the immune system in breast cancer through the down-regulation of several molecules involved in the anti-tumor immune surveillance. The overexpression of CUL4A induced a reduction of TAP1, IFIT1 and MX1 protein levels. Low or defective TAP1 in breast tumors predicts higher risks for developing metastasis [35] and down-regulation of the interferon-stimulated genes *IFIT1* and *MX1* has been linked to immune evasion mechanisms and tumor progression [36-39]. Interestingly, E3 ligases are involved in the efficient regulation of the immune system [40-42] and alterations in the ubiquitination machinery/pathway are associated with immune deficiencies and cancer [43].

In summary, we propose *CUL4A* as an important contributor to the development and progression of basal-like breast cancers. Our results indicate that *CUL4A* might play a central role in the oncogenic process by modulating the expression of a number of oncogenes and tumor suppressors. Interestingly, there is a growing number of E3 ubiquitin ligases being involved in cancer. For example, MDM2 and IAP are highly expressed in several human cancers [44, 45] and inhibitors for these enzymes are being evaluated in clinical trials [43]. Thereby *CUL4A* would constitute a promising target for therapeutic intervention and our data reinforce its clinical value in basal-like breast cancers.

## MATERIALS AND METHODS

### Cell lines and cell culture

A panel of 13 human breast epithelial cell lines was included in this study (Supplementary Table S1). Eight corresponded to sporadic breast cancer tumors (MDAMB157, MFM223, HCC1143, SKBR3, Hs578T, MCF7, MDAMB231 and T47D). Two derived from breast tumors of *BRCA1* mutation carrier patients (HCC1937 and MDAMB436) and one derived from a breast tumor with somatic inactivation of the *BRCA1* gene (UACC3199). Two human mammary breast cell lines derived from normal breast tissue (184B5, HBL100). SKBR3, T47D, HBL100, MCF7, MDAMB231, Hs578T, and UACC3199 were obtained from the Cancer Epigenetics Group at the Bellvitge Institute for Biomedical Research (Barcelona, Spain); MDAMB157, HCC1143 and HCC1937 were kindly provided by Dr. P. Edwards (Department of Pathology, University of Cambridge, Cambridge, UK); MDAMB436 was provided by Dr. K.S. Massey- Brown (Department of Pharmacology and Toxicology, University of Arizona, Tucson, AZ); MFM223 cells were purchased from DSMZ (Braunschweig, Germany); 184B5 was acquired from the ATCC (Manassas, VA, USA). Authentication was not carried out. MDAMB157 presented amplification at the 13q34 region [12, http://www.sanger.ac.uk/] and were grown in Dulbecco's Modified Eagle's Medium (Gibco, Life Technologies, Grand Island, NY). 184B5 was cultured in a 1:1 mixture of Dulbecco's modified Eagle's medium and F12 medium (DMEM-F12, Sigma-Aldrich, St. Louis, MO) supplemented with 5% horse serum (Invitrogen, Life Technologies, Carlsbad, CA), insulin (10 μg/ml), hydrocortisone (0.5 μg/ml), cholera toxin (100 ng/ml), and epidermal growth factor (20 ng/ml) (Sigma-Aldrich). The remaining cell lines were grown in RPMI-1640 (Sigma-Aldrich) or DMEM (Gibco, Life Technologies). All mediums were completed with 10% FBS, 1% penicillin/streptomycin, and 0.5% fungizone (Gibco, Life Technologies). Cells were maintained in an atmosphere of 5% CO^2^ in air at 37ºC.

### RNA isolation and real-time quantitative PCR (qPCR)

Total RNA was extracted from three independent plates for each cell line (RNAeasy Kit, Quiagen, Valencia, CA) and converted to cDNA by using 500ng as template (High Capacity cDNA RT kit, Applied Biosystems, Foster City, CA). qPCR assays were designed for target gene (*CUL4A*) and endogenous control (*β-ACTIN* ) using the Roche Universal Probe Library Assay Design Centre web site (Roche Applied Science, Indianapolis, IN) (Supplementary Table S2). Reactions were performed in triplicate using the ABI Prism 7900HT Sequence Detection System according to the manufacturer's protocol (Applied Biosystems, Foster City, CA). Relative expression was determined using the qBase software that allows for PCR efficiency correction and implements normalization by endogenous genes [46].

### Protein extraction and immunoblot analysis

Cells were harvested and lysed in RIPA buffer [50 mmol/L Tris-HCl, pH 8.0, with 150 mmol/L NaCl, 1.0% Igepal CA-630 (NP-40), 0.5% sodium deoxycholate, and 0.1% SDS; Sigma-Aldrich, St. Louis, MO] and protease inhibitor cocktail (Roche Applied Science, Foster City, CA). Protein concentration of samples was determined by detergent-compatible colorimetric assay (Bio-Rad Laboratories, Hercules, CA). Ninety micrograms of total protein were subjected to electrophoresis on NUPAGE 4% to 12% Bis–Tris gels and MOPS running buffer (Novex, Carlsbad, CA) followed by blotting to nitrocellulose membranes. The blots were analized using polyclonal rabbit anti-CUL4A (1:100; #2699; Cell Signaling Technology, Danvers, MA), polyclonal rabbit anti-Myc (1:1000; #06-340; Millipore, Billerica, MA), mouse anti-Pan-Ras (1:1000; #OP40; Millipore, Billerica, MA) and monoclonal mouse anti-β-actin (1:12,000, clone AC-15, #A5441, Sigma-Aldrich, St. Louis, MO) antibodies. The immunoblot signals were quantified using the ImageJ 1.43u software (Wayne Rasband, National Institutes of Health). CUL4A bands intensity were normalized to β-actin bands.

### Lentiviral constructs

Cell line models were modified for the expression of *CUL4A*. For gene down-regulation short hairpin RNAs (shRNA) for human *CUL4A* (NM_003589) were obtained from Sigma-Aldrich (MISSION shRNA Bacterial Glycerol Stock). Five different shRNA constructs were transduced, and the one providing better knockdown efficiency and non-citotoxic effects was selected (SH4, TRCN0000006530; Supplementary Table S3) to silence *CUL4A* expression in the HCC1937 and MDAMB157 cell lines. A MISSION Non-target shRNA vector (Scramble SHC002) was used as negative control. In order to up-regulate the expression of *CUL4A* a human cDNA of the gene (ID CNIO: hAE5834) containing an HA tag was cloned into FG12-CMV vector (kindly provided by Dr. M. Soengas at CNIO) that expresses green fluorescent protein (GFP). FG12-CMV empty vector was used as negative control.

CUL4A-overexpressing cell models and controls were modified for the expression of *H-Ras-V12* and *c-MYC* by using a retroviral system. The retroviral vectors pBabe-Neo-*H-Ras*-*V12* and pBabe-Puro*-c-Myc* were kindly provided by Dr. Víctor Javier Sánchez-Arévalo at CNIO.

### Generation of stable cell lines

Lentiviral vectors were cotransfected with lentiviral packaging plasmids into 293FT cells (Invitrogen, Carlsbad, CA) in the presence of Fugene 6 (Promega, Madison, WI). The virus-containing supernatants were collected after 36 hours of transfection and filtered. Cells were infected and the stably transduced cells were selected either by adding medium containing 1 mg/mL of puromycin (*CUL4A* down-regulation) or by GFP sorting by flow cytometry (FACS Calibur; BD Biosciences, San Jose, CA) (*CUL4A* up-regulation). Modification of *CUL4A* levels was confirmed by q-PCR and Western blot. Retroviral vectors (pBabe-Neo-*H-Ras*-*V12* or pBabe-Puro*-c-Myc*) were cotransfected with *pCL-Ampho* retroviral packaging plasmid into 293FT cells. Infection was carried out as explained above and the selection was performed by using neomycin or puromycin, respectively.

### Cell viability assay

Viability was measured by using the CellTiter-Glo Luminescent Cell Viability Assay from Promega, according to the manufacturer's recommendations. Cells were seeded on 96-well plates at densities of 1500 or 2500 cells per well. Luminescence was recorded using a plate reader (Wallac 1420 VICTOR2™, PerkinElmer, Waltham, MA).

### BrdU incorporation assay

Cells were incubated with 10 μM bromodeoxyuridine (BrdU) for 30 minutes, fixed, permeabilized and processed using anti-BrdU antibody (BD Bioscence, San Jose CA) and fluorescein isothiocianate (FITC) secondary antibody (Dako Cytomation, Glostrup, Denmark). At least 10,000 single events were assembled. Staining was analyzed by flow cytometry (FACS Calibur; BD Biosciences). All data were analyzed using FlowJo software (TreeStar, Oregon).

### Cell-cycle analysis

Cells were fixed in ethanol and incubated with 50 μg/ml propidium iodide and 10 mg/ml RNAse A (Sigma-Aldrich, St. Louis, MO) followed by analysis on a FACS Calibur flow cytometer (BD Biosciences). At least 10,000 single events were collected. All data were analyzed using FlowJo software (TreeStar, Oregon).

### Colony formation assay

Cells were seeded at densities of 1,500 or 2500 cells per well in 6-well plates and incubated for 9 to 10 days. Medium was removed and cells were washed, fixed, and stained with 0.1% crystal violet. Cell density was assessed by solubilization of crystal violet with 15% acetic acid and absorbance of the solution was measured at 590 nm (Wallac 1420 VICTOR2™, PerkinElmer, Waltham, MA).

### Soft agar assay

30,000 cells were embedded in 0.3% agar over a 0.6% base agar layer. Soft agar cultures were fed with growth media once a week and incubated during 3 weeks. To visualize colonies, cultures were stained with MTT and counted manually.

### Orthotopic xenografts

Primary tumorigeneis *in vivo* was evaluated by orthotopicaly inoculating *CUL4A*-modified breast cell lines into the mammary glands of 6-month-old female adult athymic nude mice (Hsd:Athymic Nude-Foxn1^nu^). One million HCC1937 cells and two million MDAMB157 cells infected with the *CUL4A*-shRNA vector (or with the Scramble-shRNA) were inoculated. Tumor growth was monitored weekly with a caliper and tumor volumes were calculated using the formula volume= [(length x width^2^)/2]. The experiment was stopped, mice were sacrificed, and tumors were harvested when tumors in control animals reached approximately 1 cm^3^. Mean volume of tumors per group of animals (injected with *CUL4A*-silenced and control cells) was measured each week. Animal experimentation at the CNIO, Madrid, was performed according to protocols approved by the CNIO-ISCIII Ethics Committee for Research and Animal Welfare (CEIyBA).

### Proteomic analysis

An iTRAQ proteomics approach was performed to identify proteins and pathways associated with *CUL4A* modulation. To this end we looked for differentially expressed proteins between each of the *CUL4A*-overexpressing cell line models (HCC1937 and MDAMB157 transduced with *CUL4A* specific shRNA, SH4) and their corresponding control cells (transduced with scrambled shRNA). We also looked for differentially expressed proteins between the CUL4A-overexpresing 184B5 cells (transduced with CUL4A cDNA) and control cells (transduced with empty vector). Cell pellets were extracted and proteins were digested using the FASP protocol [47]. Peptides were labeled with iTRAQ reagents and samples were pooled. Then, the complex mixture was subjected to IEF fractionation. The resulting fractions were separated by on-line nano-LC and analyzed by electrospray MS/MS using a LTQ Orbitrap Velos mass spectrometer (Thermo Scientific, Bremen, Germany). Raw files were searched against SwissProt human database (release date: March 21, 2012; 20329 entries) using MASCOT [48] as a search engine through the Proteome Discoverer (Thermo Scientific) software. Peptides were filtered at 1% FDR using a concatenated target-decoy database. Proteins were considered differentially regulated using a cutoff > 1.25-fold. A more detailed description of methodology is provided in the Supplementary Information.

### Statistical analyses

We used SPSS version 13 software to conduct the statistical analysis of our data. Two-tailed Student t tests were used for comparisons between different groups. Statistical analysis of the mean tumor volumes was done by U test of Mann-Whitney analysis. *P*< 0.05 and *P<* 0.01 were considered to be significant. Unless otherwise stated, the error bars represent standard deviation of the biologic triplicates.

## SUPPLEMENTARY FIGURE TABLES AND METHODS




