# Supplementary material for: CUL4A contributes to the biology of basal-like breast tumors through modulation of cell growth and antitumor immune response
Source: Oncotarget. 2014 Apr 18;5(8):2330–43. doi: 10.18632/oncotarget.1915 (PMC4039166; doi:10.18632/oncotarget.1915)
Supplement: Supplementary file 1 [file oncotarget-05-2330-s001.pdf]

CUL4A contributes to the biology of basal-like breast tumors through modulation of cell growth and antitumor immune response

SUPPLEMENTARY INFORMATION

Supplementary Table S1: Human breast epithelial cell lines features.

Cell line	Subtype†‡	ER†‡	PR†‡	ERBB2†‡	BRCA1 status§
HCC1937[a]	Basal-like	-	-	-	LOH/mut
MDAMB436[a]	Basal-like	-	-	-	LOH/mut
UACC3199[b, *]	Basal-like	-	-	-	LOH/met
MDAMB157	Basal-like	-	-	-	LOH/wt
MFM223[$]	NA	-	-	-	NA
HCC1143	Basal-like	-	-	-	NA
SK-BR-3	Luminal	-	-	+	LOH/wt
Hs578T	Basal-like	-	-	-	LOH/wt
MCF7	Luminal	+	+	-	LOH/wt
MDAMB231	Basal-like	-	-	-	LOH/wt
T47D	Luminal	+	+	-	no LOH/wt
184B5[¶]	Basal-like	-	-	-	NA
HBL-100	Basal-like	-	-	-	NA

[a, b] *BRCA1*-null cell lines; †Ref. [1]; ‡Ref. [2]; §Ref. [3]; * Ref. [4]; $ Ref. [5]; ¶ Ref. [6]. (+) positive, (-) negative; NA: not available.

Supplementary Table S2: Primer designed for *CUL4A* and *BACT* genes.

Gene	*Primers*	Universal Probe #
CUL4A	FW: ggaaagcacagtggtcgaa<br>RV: gggacacctggaattccttc	#59: cagtggca
BACT	FW: ccaaccgcgagaagatga<br>RV: ccagaggcgtacagggatag	#64: ccaggctg

Supplementary Table S3: *CUL4*A specific shRNAs.

clon	region	shRNA sequence
NM_003589.1-1076s1c1	CDS	SH1: CCGGGCAGAACTGATCGCAAAGCATCTCGAGATGCTTTGCGATCAGTTCTGCTTTTT
NM_003589.1-629s1c1	CDS	SH2: CCGGCCAGAATATCTTAACCATGTACTCGAGTACATGGTTAAGATATTCTGGTTTTT
NM_003589.1-600s1c1	CDS	SH3: CCGGGCCAAAGGTTAATGCAGGAAACTCGAGTTTCCTGCATTAACCTTTGGCTTTTT
NM_003589.1-532s1c1	CDS	SH4: CCGGGCAGGTGTATAAAGATTCATTCTCGAGAATGAATCTTTATACACCTGCTTTTT
NM_003589.1-3467s1c1	3UTR	SH5: CCGGCCATGATATGTGGTCTAAGAACTCGAGTTCTTAGACCACATATCATGGTTTTT

Supplementary Table S4: Protein list including all the identified proteins in each of the cell lines, showing Accession numbers based on UniProtKB/Swiss-Prot database, description, gene name and log_2 protein ratios for HCC1937 (SH4/Scramble), MDAMB157 (SH4/Scramble) and 184B5 (Over-expression/Empty vector).

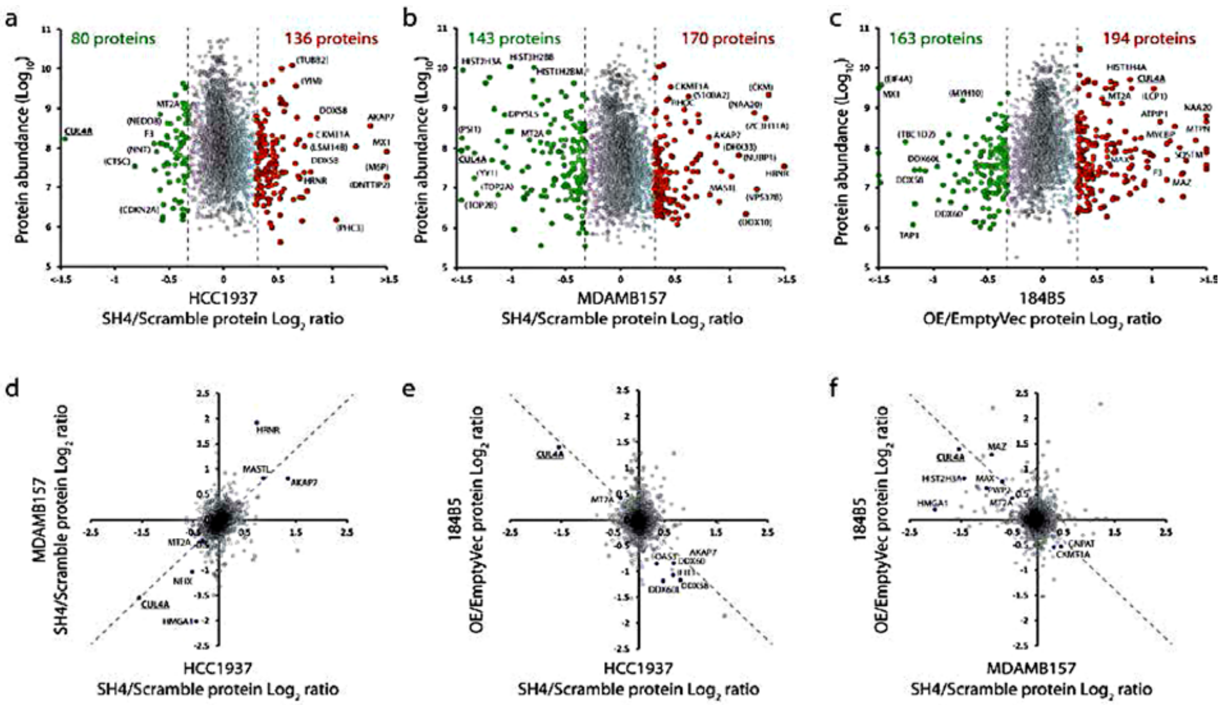

Supplementary Figure S1: Proteomic analysis of CUL4A-silenced or over-expressed cellular models and their correlation. (**a-c**) The protein cellular model profiles. The protein ratios ($\log_2$) are plotted against the protein abundances ($\log_{10}$). The spot color indicates proteins up- (in red) or down-(in green) regulated using a cutoff of >1.25-fold (±0.32 in $\log_2$). Selected individuals among the 64 differentially expressed proteins are highlighted. In brackets, some examples of significant proteins in a specific cell line. (**d-f**) Correlation among cell models. The protein ratios ($\log_2$) between the different cell lines are plotted. There is a positive correlation between CUL4A silenced cell lines, HCC1937 and MDAMB157. On the contrary, there is a negative correlation between CUL4A over-expressed 184B5 cell line and HCC1937 and MDAMB157 cell lines. Selected proteins are highlighted.

SUPPLEMENTARY METHODS

Proteomic analysis

Sample Preparation. For protein extraction, HCC1937, MDAMB157 and 184B5 cell pellets were washed 3 times with cold PBS containing protease inhibitors (Mini Complete, Roche, Indianapolis, IN) and them resuspended in 500 µL of ice-cold RIPA lysis buffer (Sigma-Aldrich, St. Louis, MO))

containing 150 mM NaCl, 1.0% IGEPAL® CA-630, 0.5% sodium deoxycholate, 0.1% SDS, 50 mM Tris, pH 8.0. Samples were vortexed, sonicated and clarified by centrifugation at 4°C and 16,100 × *g* for 15 min. The supernatants containing the protein fraction were collected and cleaned up by acetone precipitation with 6 volumes of ice-cold acetone. Pellets were dissolved in 7M urea 2M tiourea. The protein concentration of the samples was determined according to the Bradford assay using BSA as standard (Protein Assay Kit, Bio-Rad, Hercules, CA).

Protein Digestion and labeling with iTRAQ reagents. Samples were digested using the filter aided sample preparation (FASP) method [7]. Briefly, 100 ug of each sample dissolved in 7M urea 2M thiourea was loaded on the filter, reduced with 10 mM DTT 1 h at 37ºC and alkylated using 50 mM iodoacetamide for 20 min in the dark. The excess of reduction and alkylation reagents was washed. The proteins were digested overnight using endoproteinase Lys-C (Wako, Germany) with 1:50 enzyme to protein ratio. Finally, trypsin (Promega, Madison, WI) was added and samples were subjected to a second digestion for 6 h. Each tryptic digest was labeled according to the manufacturer's instructions (AB Sciex, Foster City, CA) with one isobaric amine-reactive tag. The labeling design for the three independent iTRAQ assays included three 2-plex experiments. For cell line HCC1937, cells transduced with *CUL4A* specific shRNA (SH4) and control shRNA (Scramble) were labeled with tags 114 and 116 respectively; for cell line MDAMB157, cells transduced with *CUL4A* specific shRNA (SH4) and control shRNA (Scramble) were labeled with tags 115 and 117 respectively. Lastly, for cell line 184B5, *CUL4A*-over-expressing cells and cells infected with the empty vector were labeled with tags 116 and 117 respectively. After one hour incubation, labeled samples were pooled and evaporated to dryness in a vacuum centrifuge. The iTRAQ sample was cleaned up using a Sep-Pak C18 cartridge for SPE (Waters Corp., Milford, MA) [8]. Eluted peptides were vacuum-dried and reconstituted in 8M urea, 5% glycerol and 1% ampholytes pH 3-10 prior to electrofocusing.

OFFGEL Fractionation. For pI-based peptide separation, we used the 3100 OFFGEL Fractionator system (Agilent Technologies, Böblingen, Germany) with a 24-well set-up. The IPG gel strips of 24 cm-long (GE Healthcare, München, Germany) with a 3–10 linear pH range were rehydrated for 15 min with the Peptide IPG Strip Rehydratation Solution according to the protocol of the manufacturer. Subsequently, 150 µL of sample was loaded in each well. Electrofocusing of the peptides was performed at 20°C and 50 µA until the 50 kVh level was reached. After focusing, the 24 peptide fractions were withdrawn and the wells rinsed with 100 µL of a solution of 0.1%TFA. Rinsing solutions were pooled with their corresponding peptide fraction. All fractions were evaporated by centrifugation under vacuum. Solid phase extraction and salt removal was performed with home–made columns based on Stage Tips with C8 Empore Disks (3M, Minneapolis, MN) [9] filled with R3 resin (Applied Biosystems). Eluates were evaporated to dryness and maintained at 4°C. Just prior nano-LC, the fractions were resuspended in H_2O with 0.1% (v/v) FA.

Peptide analysis by nanoLC-MS/MS. Digested samples were separated by on-line reversed-phase nanoscale capillary LC and analyzed by electrospray MS/MS. The experiments were performed on an Eksigent nano LC system (Eksigent, Redwood City, CA) coupled to an LTQ Orbitrap Velos mass spectrometer (Thermo Scientific, Bremen) equipped with a nanoelectrospray ion source (Proxeon Biosystems, part of Thermo Scientific). Peptides were resuspended in 0.1% FA and loaded from a cooled nanoLC AS-2 autosampler (Eksigent). In order to pre-concentrate and desalt the samples before switching the pre-column in line with the separation column, 5 µL from each sample was loaded onto a reversed-phase ReproSil Pur C18-Aq 5 µm 0.3 x 10 mm trapping cartridge (SGE Analytical, Victoria, Australia), and washed for 5 min at 2.5 µL/min with loading buffer (0.1% FA). The peptides were eluted from a RP ReproSil Pur C18-AQ 3 µm 200 x 0.075 mm (Dr. Maisch GmbH, Ammerbuch-Entringen) by application of a binary gradient consisting of 2% ACN in 0.1% FA (buffer A) and 100% ACN in 0.1%FA (buffer B), with a flow rate of 300 nL/min. Peptides were separated using the following gradient: 0-5 min 2% B, 5-150 min 45% B and 150-165 min 98% B.

The column was operated at a constant temperature of 30ºC. The LTQ Orbitrap Velos was operated in positive ionization mode. The MS survey scan was performed in the FT analyzer scanning a window between 250 and 1750 *m*/*z*. The resolution was set to 60,000 FWHM at *m/z* 400 and the automatic gain control (AGC) was set to 500,000 ions. The *m*/*z* values triggering MS/MS with a repeat count of 1 were put on an exclusion list for 60 s. The minimum MS signal for triggering MS/MS was set to 1,000 counts. In all cases, one microscan was recorded. The lock mass option was enabled for both MS and MS/MS mode and the polydimethylcyclosiloxane ions (PDMS, protonated $(Si(CH_3)_2O))_6$; *m*/*z* 445.120025) were used for internal recalibration of the mass spectra [10]. For the HCD, up to the 15 most abundant isotope patterns with charge ≥2 from the survey scan were selected with an isolation window of 2 *m*/*z* fragmented in the C-trap collision cell. Normalized collision energy was set to 42%, the Q value to 0.25 and an activation time to 0.10 ms. Waveform filter was activated. The resulting fragments were detected in the Orbitrap system with a resolution of 7,500 FWHM at *m/z* 400. The maximum ion injection times for the survey scan and the MS/MS scans were 500 ms and 250 ms respectively and the ion target values were set to 1E6 and 4E5, respectively for each scan mode.

Data analysis. The raw files were processed using the Proteome Discoverer 1.3.0.339 software suite (Thermo Scientific). The fragmentation spectra were searched against the SwissProt Human database (release date: March 21, 2012; 20,329 entries) using MASCOT [11] as the search engine (v 2.2) with the precursor and fragment mass tolerances set to 20 ppm and 0.02 Da, respectively, and with up to two missed cleavages. Lysine and peptide N-termini labelling with iTRAQ-4plex reagent as well as carbamidomethylation of cysteine were considered as fixed modifications, while oxidation of methionine was chosen as variable modification for database searching. Peptide identification was filtered at 1% false discovery rate (FDR) and thus not dependent on the peptide score. Only peptides with Maximun rank =1, 6 or more amino acids length and Ionscore (Mascot) above 20 were considered. The results were then exported into Excel for manual data interpretation. Although relative quantification and coefficients of variation data were provided by the Proteome Discoverer software, we chose a 1.25-fold change cutoff for all iTRAQ ratios (ratio <0.8 or >1.25) was selected to classify proteins as down- or up-regulated [12-14]. The criterion was based on the distribution of all quantified proteins in each experiment, using 1.5 times the standard deviation (i.e. 5% of most differentially expressed proteins). Proteins with iTRAQ ratios below the low range (0.8) were considered to be under-expressed, while those above the high range (1.25) were considered overexpressed. (Supplementary Table S4). We elaborated a list of 64 candidate proteins to be regulated upon modulation of CUL4A expression. We included proteins that showed an opposite behavior as it were in the overexpressing model or in the silencing models. This premise was established in the case of proteins identified and significantly regulated in the three cellular models but also for proteins identified and significantly regulated in the 184B5 cells and in one of the silencing models. Finally, proteins showing the same behavior in the two silencing models were included in the final list. The 64 proteins were classified according to PANTHER protein classes using the entire list of identified proteins as the reference data set [15].

REFERENCES

1. Kao J, Salari K, Bocanegra M, Choi YL, Girard L, Gandhi J, Kwei KA, Hernandez-Boussard T, Wang P, Gazdar AF, Minna JD and Pollack JR. Molecular profiling of breast cancer cell lines defines relevant tumor models and provides a resource for cancer gene discovery. PLoS One. 2009; 4(7):e6146.
2. Neve RM, Chin K, Fridlyand J, Yeh J, Baehner FL, Fevr T, Clark L, Bayani N, Coppe JP, Tong F, Speed T, Spellman PT, DeVries S, Lapuk A, Wang NJ, Kuo WL, et al. A collection of breast

cancer cell lines for the study of functionally distinct cancer subtypes. Cancer Cell. 2006; 10(6):515-527.
3. Elstrodt F, Hollestelle A, Nagel JH, Gorin M, Wasielewski M, van den Ouweland A, Merajver SD, Ethier SP and Schutte M. BRCA1 mutation analysis of 41 human breast cancer cell lines reveals three new deleterious mutants. Cancer Res. 2006; 66(1):41-45.
4. Mackay A, Tamber N, Fenwick K, Iravani M, Grigoriadis A, Dexter T, Lord CJ, Reis-Filho JS and Ashworth A. A high-resolution integrated analysis of genetic and expression profiles of breast cancer cell lines. Breast Cancer Res Treat. 2009; 118(3):481-498.
5. Turner N, Lambros MB, Horlings HM, Pearson A, Sharpe R, Natrajan R, Geyer FC, van Kouwenhove M, Kreike B, Mackay A, Ashworth A, van de Vijver MJ and Reis-Filho JS. Integrative molecular profiling of triple negative breast cancers identifies amplicon drivers and potential therapeutic targets. Oncogene. 2010; 29(14):2013-2023.
6. Ross DT and Perou CM. A comparison of gene expression signatures from breast tumors and breast tissue derived cell lines. Dis Markers. 2001; 17(2):99-109.
7. Wisniewski JR, Zougman A, Nagaraj N and Mann M. Universal sample preparation method for proteome analysis. Nat Methods. 2009; 6(5):359-362.
8. Ernoult E, Gamelin E and Guette C. Improved proteome coverage by using iTRAQ labelling and peptide OFFGEL fractionation. Proteome Sci. 2008; 6:27.
9. Rappsilber J, Ishihama Y and Mann M. Stop and go extraction tips for matrix-assisted laser desorption/ionization, nanoelectrospray, and LC/MS sample pretreatment in proteomics. Anal Chem. 2003; 75(3):663-670.
10. Olsen JV, de Godoy LM, Li G, Macek B, Mortensen P, Pesch R, Makarov A, Lange O, Horning S and Mann M. Parts per million mass accuracy on an Orbitrap mass spectrometer via lock mass injection into a C-trap. Mol Cell Proteomics. 2005; 4(12):2010-2021.
11. Perkins DN, Pappin DJ, Creasy DM and Cottrell JS. Probability-based protein identification by searching sequence databases using mass spectrometry data. Electrophoresis. 1999; 20(18):3551-3567.
12. Chen Y, Choong LY, Lin Q, Philp R, Wong CH, Ang BK, Tan YL, Loh MC, Hew CL, Shah N, Druker BJ, Chong PK and Lim YP. Differential expression of novel tyrosine kinase substrates during breast cancer development. Mol Cell Proteomics. 2007; 6(12):2072-2087.
13. Gan CS, Chong PK, Pham TK and Wright PC. Technical, experimental, and biological variations in isobaric tags for relative and absolute quantitation (iTRAQ). J Proteome Res. 2007; 6(2):821-827.
14. Ho J, Kong JW, Choong LY, Loh MC, Toy W, Chong PK, Wong CH, Wong CY, Shah N and Lim YP. Novel breast cancer metastasis-associated proteins. J Proteome Res. 2009; 8(2):583-594.
15. Mi H, Muruganujan A and Thomas PD. PANTHER in 2013: modeling the evolution of gene function, and other gene attributes, in the context of phylogenetic trees. Nucleic Acids Res. 2013; 41(Database issue):D377-386.
