# Supplementary material for: CUL4A contributes to the biology of basal-like breast tumors through modulation of cell growth and antitumor immune response
Source: Oncotarget. 2014 Apr 18;5(8):2330–43. doi: 10.18632/oncotarget.1915 (PMC4039166; doi:10.18632/oncotarget.1915)
Supplement: Supplementary file 2 [file oncotarget-05-2330-s002.pdf]

Accession	Description	Gene name	HCC1937 (SH4/Scramble)	MDAMB157 (SH4/Scramble)	184B5 (Over/Empty vector)
P58546	Myotrophin	MTPN	-0.216	-0.219	3.077
P61599-2	Isoform 2 of N-alpha-acetyltransferase 20	NAA20		1.227	2.292
H3BS21	Haptoglobin (Fragment)	HP			2.214
G3V5J8	Zinc transporter ZIP9	SLC39A9	-0.014	-0.879	2.212
Q53FA7	Quinone oxidoreductase PIG3	TP53I3			1.542
Q13501-2	Isoform 2 of Sequestosome-1	SQSTM1	-0.088	0.203	1.539
Q13619	Cullin-4A	CUL4A	-1.551	-1.548	1.404
Q70UQ0-4	Isoform 4 of Inhibitor of nuclear factor kappa-B kinase-interacting protein	IKBIP			1.317
Q01995	Transgelin	TAGLN	0.676		1.286
I3L2Z5	Myc-associated zinc finger protein (Fragment)	MAZ	0.035	-0.899	1.286
Q01433-2	Isoform Ex1A-2-3 of AMP deaminase 2	AMPD2	0.006	0.096	1.275
Q99417	C-Myc-binding protein	MYCBP	-0.095	-1.074	1.257
F8W696	Truncated apolipoprotein A-I	APOA1			1.213
Q9UII2	ATPase inhibitor, mitochondrial	ATPIF1	-0.058	-1.124	1.168
Q9UN37	Vacuolar protein sorting-associated protein 4A	VPS4A	-0.032	-0.208	1.140
Q6ICB0	Desumoylating isopeptidase 1	DESI1	-0.150	0.004	1.138
A2A2Y8	Collagen alpha-1(XVII) chain	COL17A1			1.138
Q96AD5	Patatin-like phospholipase domain-containing protein 2	PNPLA2			1.090
P01584	Interleukin-1 beta	IL1B			1.078
P13796	Plastin-2	LCP1	0.092		1.020
Q02952-3	Isoform 3 of A-kinase anchor protein 12	AKAP12	0.345		1.010
P82909	28S ribosomal protein S36, mitochondrial	MRPS36			0.990
F5H163	Mevalonate kinase	MVK	-0.151	0.384	0.978
O75437-2	Isoform 2 of Zinc finger protein 254	ZNF254			0.962
Q69YN2	CWF19-like protein 1	CWF19L1	0.085	-0.311	0.961
H7BXW3	Melanoma-associated antigen D2	MAGED2		0.153	0.937
Q5BJD5-3	Isoform 3 of Transmembrane protein 41B	TMEM41B	-0.118	-0.083	0.936
P53365-3	Isoform 3 of Arfaptin-2	ARFIP2	-0.190	-0.141	0.932
P28799-2	Isoform 2 of Granulins	GRN	-0.139		0.904
O95219	Sorting nexin-4	SNX4		-0.051	0.857
Q00765	Receptor expression-enhancing protein 5	REEP5	0.254	0.192	0.852
P05121	Plasminogen activator inhibitor 1	SERPINE1			0.845
Q9ULF5	Zinc transporter ZIP10	SLC39A10			0.830
O75157-2	Isoform 2 of TSC22 domain family protein 2	TSC22D2	-0.504	-0.112	0.819
Q7KZI7-13	Isoform 13 of Serine/threonine-protein kinase MARK2	MARK2	-0.004	-0.009	0.818
P19784	Casein kinase II subunit alpha'	CSNK2A2	-0.035	0.004	0.814
Q71DI3	Histone H3.2	HIST2H3A		-1.441	0.814
P29692-3	Isoform 3 of Elongation factor 1-delta	EEF1D	-0.261	0.003	0.801
P46977	Dolichyl-diphosphooligosaccharide--protein glycosyltransferase subunit STT3A	STT3A	0.076	0.146	0.801
Q6DKI1	60S ribosomal protein L7-like 1	RPL7L1	0.488	-0.616	0.800
Q96ME1-4	Isoform 4 of F-box/LRR-repeat protein 18	FBXL18	-0.080	-0.490	0.796
Q9UHI6	Probable ATP-dependent RNA helicase DDX20	DDX20	-0.075	-0.064	0.774
Q5ZPR3-3	Isoform 3 of CD276 antigen	CD276	0.038		0.760
Q15041	ADP-ribosylation factor-like protein 6-interacting protein 1	ARL6IP1	-0.036	-0.207	0.759
Q9BY76-2	Isoform 2 of Angiopoietin-related protein 4	ANGPTL4			0.755
P61244-2	Isoform 2 of Protein max	MAX	-0.038	-0.693	0.752
P01583	Interleukin-1 alpha	IL1A			0.725
P01023	Alpha-2-macroglobulin	A2M			0.705
P49441	Inositol polyphosphate 1-phosphatase	INPP1			0.683
Q5T653	39S ribosomal protein L2, mitochondrial	MRPL2	-0.052	0.165	0.680
P47974	Zinc finger protein 36, C3H1 type-like 2	ZFP36L2	0.038		0.670
P62805	Histone H4	HIST1H4A	-0.026	-1.199	0.670
P29317	Ephrin type-A receptor 2	EPHA2	0.101		0.666
H7C3X5	Syntaxin-5 (Fragment)	STX5	0.146		0.655
O15254-2	Isoform 2 of Peroxisomal acyl-coenzyme A oxidase 3	ACOX3			0.649
Q8NFH4	Nucleoporin Nup37	NUP37	-0.163	0.020	0.646
Q14684-2	Isoform 2 of Ribosomal RNA processing protein 1 homolog B	RRP1B		-1.175	0.646
F5GXU9	2-oxoisovalerate dehydrogenase subunit alpha, mitochondrial (Fragment)	BCKDHA	0.430	-0.004	0.643
Q9Y508-2	Isoform 2 of RING finger protein 114	RNF114	0.003	-0.021	0.640
P68431	Histone H3.1	HIST1H3A		0.079	0.636
Q99879	Histone H2B type 1-M	HIST1H2BM	0.001	-1.007	0.624
H0YK64	CREB-regulated transcription coactivator 3 (Fragment)	CRTC3			0.623
P46013	Antigen KI-67	MKI67	-0.055	-0.316	0.620
Q8IYD1	Eukaryotic peptide chain release factor GTP-binding subunit ERF3B	GSPT2	-0.111	-0.021	0.617
Q9ULX9-2	Isoform 2 of Transcription factor MafF	MAFF		0.378	0.602
Q9BPU6	Dihydropyrimidinase-related protein 5	DPYSL5		-1.155	0.598
P31151	Protein S100-A7	S100A7	0.287		0.598
P32926	Desmoglein-3	DSG3			0.594
P06132	Uroporphyrinogen decarboxylase	UROD	0.079	-0.056	0.591
E7ERZ4	Mitochondrial-processing peptidase subunit beta	PMPCB	-0.035	-0.177	0.590
Q07866-7	Isoform P of Kinesin light chain 1	KLC1	-0.154	-0.151	0.588
P98179	Putative RNA-binding protein 3	RBM3	-0.091	-0.351	0.581
K7EK07	Histone H3 (Fragment)	H3F3B		-0.302	0.578
P60983	Glia maturation factor beta	GMFB	0.005	0.066	0.577
P02545-2	Isoform C of Prelamin-A/C	LMNA	0.061	0.270	0.566
H0YKV4	Family with sequence similarity 96, member A, isoform CRA_a	FAM96A	-0.018	0.235	0.563
D6RA00	Enolase-phosphatase E1	ENOPH1	-0.002	0.056	0.554
P55795	Heterogeneous nuclear ribonucleoprotein H2	HNRNPH2	0.120	-0.171	0.547
O75367-3	Isoform 3 of Core histone macro-H2A.1	H2AFY		-1.401	0.546
Q08AD1-2	Isoform 2 of Calmodulin-regulated spectrin-associated protein 2	CAMSAP2		-0.019	0.537
Q9NVI1	Fanconi anemia group I protein	FANCI	0.168	0.029	0.534
P51178	1-phosphatidylinositol 4,5-bisphosphate phosphodiesterase delta-1	PLCD1			0.528
P05187	Alkaline phosphatase, placental type	ALPP	0.081		0.517
P13726	Tissue factor	F3	-0.564		0.512
Q8TBX8-2	Isoform 2 of Phosphatidylinositol 5-phosphate 4-kinase type-2 gamma	PIP4K2C	0.118		0.508
P27361	Mitogen-activated protein kinase 3	MAPK3	0.048	0.324	0.492

Q16850	Lanosterol 14-alpha demethylase	CYP51A1	0.207	0.058	0.492
C9IY94	Septin-2 (Fragment)	SEPT2	0.064	-0.611	0.492
P02751-12	Isoform 12 of Fibronectin	FN1			0.486
B4DXE2	BAG family molecular chaperone regulator 2	BAG2	-0.131		0.482
Q8N257	Histone H2B type 3-B	HIST3H2BB	0.012	-1.012	0.477
Q96QA5	Gasdermin-A	GSDMA			0.477
Q53GA4	Pleckstrin homology-like domain family A member 2	PHLDA2			0.466
Q13596-2	Isoform 1A of Sorting nexin-1	SNX1	-0.008	-0.188	0.464
O14733	Dual specificity mitogen-activated protein kinase kinase 7	MAP2K7			0.464
M0R317	Striatin-4 (Fragment)	STRN4	0.039	-0.423	0.464
Q01581	Hydroxymethylglutaryl-CoA synthase, cytoplasmic	HMGCS1	0.407	0.204	0.459
Q92466	DNA damage-binding protein 2	DDB2	0.429	0.297	0.458
P35269	General transcription factor IIF subunit 1	GTF2F1	0.017	-0.047	0.455
H7C0M3	Valacyclovir hydrolase (Fragment)	BPHL			0.455
O14787-2	Isoform 2 of Transportin-2	TNPO2	-0.026	0.029	0.453
Q8WX92	Negative elongation factor B	NELFB	0.006	-0.087	0.452
F8W679	Succinate dehydrogenase assembly factor 2, mitochondrial	SDHAF2	0.119		0.448
O14880	Microsomal glutathione S-transferase 3	MGST3	0.355	0.327	0.445
Q8IWX8	Calcium homeostasis endoplasmic reticulum protein	CHERP	-0.151	0.053	0.445
Q05048	Cleavage stimulation factor subunit 1	CSTF1	0.083	-0.045	0.443
B7Z817	Delta(24)-sterol reductase	DHCR24	-0.128		0.436
H0YJ30	Molybdopterin molybdenumtransferase (Fragment)	GPHN	-0.005		0.432
Q9UK41	Vacuolar protein sorting-associated protein 28 homolog	VPS28	0.187	-0.169	0.429
B4DPY8	TOX high mobility group box family member 4	TOX4			0.429
P56545	C-terminal-binding protein 2	CTBP2	-0.019	-0.018	0.426
B4DK69	Aldo-keto reductase family 1 member C2	AKR1C2			0.425
Q9NRW3	DNA dC->dU-editing enzyme APOBEC-3C	APOBEC3C			0.425
P02795	Metallothionein-2	MT2A	-0.357	-0.498	0.424
Q14512	Fibroblast growth factor-binding protein 1	FGFBP1	0.370		0.424
Q9HD15	Steroid receptor RNA activator 1	SRA1	0.051	0.261	0.423
Q96C86	m7GpppX diphosphatase	DCPS	0.028	0.160	0.420
B3KXE0	General transcription factor IIH subunit 1	GTF2H1			0.416
P05120	Plasminogen activator inhibitor 2	SERPINB2			0.415
P29692	Elongation factor 1-delta	EEF1D	-0.181	-0.129	0.415
Q53EU6	Glycerol-3-phosphate acyltransferase 3	AGPAT9	-0.199		0.413
Q9Y6J9	TAF6-like RNA polymerase II p300/CBP-associated factor-associated factor 65 kDa	TAF6L			0.408
O00186	Syntaxin-binding protein 3	STXBP3	0.105	0.352	0.408
Q9NX55	Huntingtin-interacting protein K	HYPK	-0.021	-0.113	0.408
Q15269	Periodic tryptophan protein 2 homolog	PWP2	-0.024	-0.679	0.407
A9QM74	Importin subunit alpha-8	KPNA7	-0.083	0.047	0.406
P35625	Metalloproteinase inhibitor 3	TIMP3	-0.130		0.405
Q9NRL2-2	Isoform 2 of Bromodomain adjacent to zinc finger domain protein 1A	BAZ1A	0.039		0.403
P16144-4	Isoform Beta-4D of Integrin beta-4	ITGB4	-0.082	-0.138	0.402
G3XAN4	Nuclear receptor coactivator 3	TRAM1	-0.012	0.020	0.396
E5RGR9	Protein YIPF5 (Fragment)	YIPF5	-0.095	0.027	0.390
Q6PGP7	Tetratricopeptide repeat protein 37	TTC37		-0.167	0.389
H0YMQ8	Protein-glutamine gamma-glutamyltransferase K (Fragment)	TGM1			0.386
P05114	Non-histone chromosomal protein HMG-14	HMGN1	-0.265	-0.833	0.383
C9JP16	Cartilage-associated protein	CRTAP	0.004	0.225	0.381
P23434	Glycine cleavage system H protein, mitochondrial	GCSH	-0.149		0.380
Q13247-3	Isoform SRP55-3 of Serine/arginine-rich splicing factor 6	SRSF6	0.088	-0.014	0.379
P15531	Nucleoside diphosphate kinase A	NME1	-0.074	-0.088	0.379
P62256-2	Isoform 2 of Ubiquitin-conjugating enzyme E2 H	UBE2H	-0.020	0.000	0.377
Q92747	Actin-related protein 2/3 complex subunit 1A	ARPC1A	-0.043	-0.165	0.374
P49585	Choline-phosphate cytidylyltransferase A	PCYT1A	0.018	-0.047	0.373
Q15125	3-beta-hydroxysteroid-Delta(8),Delta(7)-isomerase	EBP	0.120	0.103	0.373
H7BZ18	Multiple coagulation factor deficiency protein 2 (Fragment)	MCFD2	-0.054		0.372
F8WAK8	Cohesin subunit SA-2	STAG2	0.056	0.035	0.369
B4DET3	Niemann-Pick C1 protein	NPC1			0.369
F8WD12	SET domain-containing protein 5	SETD5			0.369
Q13085-3	Isoform 3 of Acetyl-CoA carboxylase 1	ACACA	-0.015	-0.038	0.362
E9PIZ0	Protein YIF1A (Fragment)	YIF1A	-0.014	-0.150	0.361
Q9BW72	HIG1 domain family member 2A, mitochondrial	HIGD2A			0.360
Q16527	Cysteine and glycine-rich protein 2	CSRP2	0.420	-0.063	0.360
P51571	Translocon-associated protein subunit delta	SSR4	-0.049	-0.131	0.360
Q9BQC3	Diphthamide biosynthesis protein 2	DPH2			0.360
Q15800-2	Isoform 2 of Methylsterol monooxygenase 1	MSMO1	0.478	0.162	0.360
Q9NYF8-3	Isoform 3 of Bcl-2-associated transcription factor 1	BCLAF1	-0.028	0.070	0.360
P15121	Aldose reductase	AKR1B1	-0.279	0.314	0.354
O00267-2	Isoform 2 of Transcription elongation factor SPT5	SUPT5H	0.200	0.093	0.351
Q9UHQ9	NADH-cytochrome b5 reductase 1	CYB5R1	0.245		0.346
Q9UHR4	Brain-specific angiogenesis inhibitor 1-associated protein 2-like protein 1	BAIAP2L1	-0.045		0.345
G3V2D5	Zinc finger protein 36, C3H1 type-like 1 (Fragment)	ZFP36L1			0.345
Q05519-2	Isoform 2 of Serine/arginine-rich splicing factor 11	SRSF11	-0.091	-0.144	0.344
P00734	Prothrombin	F2			0.343
P52895	Aldo-keto reductase family 1 member C2	AKR1C2			0.343
B7ZBM4	Syntaxin-16 (Fragment)	STX16			0.342
Q16531	DNA damage-binding protein 1	DDB1	0.226	0.028	0.342
P07476	Involucrin	IVL			0.342
H7C1Z9	Peptidyl-prolyl cis-trans isomerase (Fragment)	FKBP14			0.341
Q9BRX2	Protein pelota homolog	PELO	-0.074	0.127	0.340
A5A3E0	POTE ankyrin domain family member F	POTEF			0.340
Q9NUQ8-2	Isoform 2 of ATP-binding cassette sub-family F member 3	ABCF3	-0.358	-0.074	0.339
Q9UPY5	Cystine/glutamate transporter	SLC7A11	0.187		0.339
Q6ZYK7	Prostaglandin G/H synthase 2	COX-2			0.338
P52292	Importin subunit alpha-2	KPNA2	-0.024	-0.104	0.338
P22392-2	Isoform 3 of Nucleoside diphosphate kinase B	NME2	-0.193	-0.139	0.338

P52732	Kinesin-like protein KIF11	KIF11	-0.015	0.048	0.336
E9PNM1	Squalene synthase	FDFT1	0.171	0.532	0.336
Q7LBC6-2	Isoform 2 of Lysine-specific demethylase 3B	KDM3B	-0.217	0.088	0.336
Q9BXW9-2	Isoform 2 of Fanconi anemia group D2 protein	FANCD2	0.045	0.238	0.335
Q8TD16	Protein bicaudal D homolog 2	BICD2	-0.012	0.102	0.335
Q9BST9-2	Isoform 2 of Rhotekin	RTKN	0.106	-0.104	0.334
Q96A35	39S ribosomal protein L24, mitochondrial	MRPL24	0.027	0.088	0.332
P54098	DNA polymerase subunit gamma-1	POLG			0.331
Q08945	FACT complex subunit SSRP1	SSRP1	-0.208	-0.340	0.329
Q8WXQ8-2	Isoform 2 of Carboxypeptidase A5	CPA5	-0.224	-0.090	0.328
P21291	Cysteine and glycine-rich protein 1	CSRP1	0.111	0.192	0.328
P37235	Hippocalcin-like protein 1	HPCAL1	0.002	-0.009	0.327
Q96HJ9-2	Isoform 2 of UPF0562 protein C7orf55	C7orf55	0.005	0.141	0.326
P35754	Glutaredoxin-1	GLRX			0.326
Q9Y5K6	CD2-associated protein	CD2AP	0.016		0.323
Q9UHB6	LIM domain and actin-binding protein 1	LIMA1	0.139		0.322
Q07157-2	Isoform Short of Tight junction protein ZO-1	TJP1	-0.004	-0.152	0.322
Q9Y3M8-4	Isoform 4 of StAR-related lipid transfer protein 13	STARD13	0.111	-0.083	0.322
I3L159	Heme oxygenase 2 (Fragment)	HMOX2	-0.001	-0.063	0.322
O75376	Nuclear receptor corepressor 1	NCOR1		0.015	0.321
Q01650	Large neutral amino acids transporter small subunit 1	SLC7A5	-0.386	0.161	0.321
Q16706	Alpha-mannosidase 2	MAN2A1			0.319
Q14966-4	Isoform 4 of Zinc finger protein 638	ZNF638	0.037	0.233	0.319
Q96AY3	Peptidyl-prolyl cis-trans isomerase FKBP10	FKBP10	-0.154		0.317
Q15388	Mitochondrial import receptor subunit TOM20 homolog	TOMM20	0.016	0.000	0.316
Q01105-2	Isoform 2 of Protein SET	SET	-0.060	0.008	0.316
O14745	Na(+)/H(+) exchange regulatory cofactor NHE-RF1	SLC9A3R1	0.097	-0.181	0.316
E9PH29	Thioredoxin-dependent peroxide reductase, mitochondrial	PRDX3	0.007	-0.078	0.315
E7ES52	Cyclin-dependent kinase inhibitor 1B	CDKN1B			0.315
H3BV49	Putative WAS protein family homolog 4 (Fragment)	WASH4P	0.076	-0.250	0.314
P68371	Tubulin beta-4B chain	TUBB4B	-0.107	-0.224	0.313
O60343-2	Isoform 2 of TBC1 domain family member 4	TBC1D4			0.311
Q01518	Adenylyl cyclase-associated protein 1	CAP1	0.092	0.086	0.310
P11908	Ribose-phosphate pyrophosphokinase 2	PRPS2	-0.133	-0.063	0.310
Q9ULP9-2	Isoform 2 of TBC1 domain family member 24	TBC1D24			0.307
P62942	Peptidyl-prolyl cis-trans isomerase FKBP1A	FKBP1A	-0.054	-0.129	0.306
P02792	Ferritin light chain	FTL	0.069		0.304
Q96KB5	Lymphokine-activated killer T-cell-originated protein kinase	PBK	-0.018	-0.128	0.303
Q8IWS0	PHD finger protein 6	PHF6	0.059	-0.063	0.303
Q16763	Ubiquitin-conjugating enzyme E2 S	UBE2S	0.122	-0.210	0.301
Q86UY8	5'-nucleotidase domain-containing protein 3	NT5DC3	0.043	0.118	0.299
P08195-2	Isoform 2 of 4F2 cell-surface antigen heavy chain	SLC3A2	-0.273	0.233	0.298
Q13509	Tubulin beta-3 chain	TUBB3	0.632	-0.424	0.298
Q04323	UBX domain-containing protein 1	UBXN1	0.038	0.042	0.297
E7EVJ5	Cytoplasmic FMR1-interacting protein 2	CYFIP2			0.296
Q9BRT6	Protein LLP homolog	LLPH	0.105	0.015	0.294
Q9BVA1	Tubulin beta-2B chain	TUBB2B	0.244	0.317	0.293
P40938	Replication factor C subunit 3	RFC3	-0.010	-0.131	0.293
Q9Y305-4	Isoform 4 of Acyl-coenzyme A thioesterase 9, mitochondrial	ACOT9	0.256	0.029	0.292
P02766	Transthyretin	TTR			0.292
P28482	Mitogen-activated protein kinase 1	MAPK1	0.091	0.056	0.292
Q6N069	N-alpha-acetyltransferase 16, NatA auxiliary subunit	NAA16	-0.286		0.291
Q13751	Laminin subunit beta-3	LAMB3	-0.010	0.346	0.291
O95486	Protein transport protein Sec24A	SEC24A	-0.145	-0.110	0.290
Q7Z4V5	Hepatoma-derived growth factor-related protein 2	HDGFRP2	0.021	-0.117	0.287
Q01804	OTU domain-containing protein 4	OTUD4	0.236	-0.155	0.287
P35658-2	Isoform 2 of Nuclear pore complex protein Nup214	NUP214	-0.025	-0.052	0.285
C9J0X4	Histone deacetylase 4 (Fragment)	HDAC4			0.285
Q13753	Laminin subunit gamma-2	LAMC2	-0.150		0.285
P21333-2	Isoform 2 of Filamin-A	FLNA	0.030	0.141	0.285
O43399	Tumor protein D54	TPD52L2	-0.084	-0.029	0.283
O14896	Interferon regulatory factor 6	IRF6	-0.552		0.283
O95456	Proteasome assembly chaperone 1	PSMG1	-0.067	-0.019	0.281
O43818	U3 small nucleolar RNA-interacting protein 2	RRP9	-0.012	-0.209	0.278
B3KWS3	Procollagen-lysine,2-oxoglutarate 5-dioxygenase 2	PLOD2	-0.222	0.345	0.278
F8W914	Reticulon-4	RTN4	-0.145	-0.131	0.277
P26006	Integrin alpha-3	ITGA3	0.003		0.274
P38159-2	Isoform 2 of RNA-binding motif protein, X chromosome	RBMX	-0.017	-0.794	0.273
P33240-2	Isoform 2 of Cleavage stimulation factor subunit 2	CSTF2	0.073	0.034	0.273
P51580	Thiopurine S-methyltransferase	TPMT	-0.109		0.272
B7Z382	Cytosolic purine 5'-nucleotidase	NT5C2	0.120	0.019	0.271
Q96H55-3	Isoform 3 of Unconventional myosin-XIX	MYO19	0.211		0.271
Q9Y6A5	Transforming acidic coiled-coil-containing protein 3	TACC3	-0.031	0.022	0.270
M0R0S1	SH3KBP1-binding protein 1 (Fragment)	SHKBP1			0.270
Q9BT09	Protein canopy homolog 3	CNPY3	-0.186	0.099	0.270
O00629	Importin subunit alpha-4	KPNA4	0.024	-0.021	0.268
Q9GZT3-2	Isoform 2 of SRA stem-loop-interacting RNA-binding protein, mitochondrial	SLIRP	-0.073	-0.071	0.266
P27482	Calmodulin-like protein 3	CALML3			0.265
Q99986	Serine/threonine-protein kinase VRK1	VRK1	-0.298	-0.206	0.265
P31689	DnaJ homolog subfamily A member 1	DNAJA1	-0.321	-0.339	0.265
P02786	Transferrin receptor protein 1	TFRC	-0.010	0.109	0.264
Q9BVR6	Gamma-tubulin complex component 4	TUBGCP4			0.264
Q9Y5P4-2	Isoform 2 of Collagen type IV alpha-3-binding protein	COL4A3BP			0.263
P42330	Aldo-keto reductase family 1 member C3	AKR1C3			0.263
P10644	cAMP-dependent protein kinase type I-alpha regulatory subunit	PRKAR1A	-0.308	0.027	0.263
F5H8J3	Cleft lip and palate transmembrane protein 1	CLPTM1	0.080	-0.027	0.263
O75362	Zinc finger protein 217	ZNF217	0.100	-0.348	0.262

P53701	Cytochrome c-type heme lyase	HCCS	0.159	-0.002	0.262
Q6UXN9	WD repeat-containing protein 82	WDR82	0.048	0.030	0.261
P83916	Chromobox protein homolog 1	CBX1	0.191	-0.197	0.260
C9JTZ6	5'-nucleotidase domain-containing protein 2	NT5DC2	0.126	0.057	0.259
Q9BXX2	Ankyrin repeat domain-containing protein 30B	ANKRD30B	-0.117	-0.134	0.258
Q06210-2	Isoform 2 of Glutamine--fructose-6-phosphate aminotransferase [isomerizing] 1	GFPT1	0.198	0.121	0.257
M0QZI4	Phospholipase D3 (Fragment)	PLD3	0.108		0.257
O75475	PC4 and SFRS1-interacting protein	PSIP1	-0.098	-1.495	0.256
Q7RTV0	PHD finger-like domain-containing protein 5A	PHF5A	0.025	-0.273	0.256
P07858	Cathepsin B	CTSB	-0.294	0.102	0.254
Q9H871	Protein RMD5 homolog A	RMND5A			0.253
O43731	ER lumen protein retaining receptor 3	KDELR3			0.252
Q9H2P0	Activity-dependent neuroprotector homeobox protein	ADNP	0.008	-0.090	0.251
Q8IVF2-3	Isoform 3 of Protein AHNAK2	AHNAK2	0.168	0.092	0.250
F8WF48	Translocation protein SEC62	SEC62	0.394	0.143	0.250
Q9NU22	Midasin	MDN1	-0.032	0.098	0.249
H0Y6X5	Mitochondrial import inner membrane translocase subunit Tim23 (Fragment)	TIMM23			0.249
Q5T8N1	Nuclear pore complex protein Nup133	NUP133	0.103	-0.041	0.249
P19957	Elafin	PI3			0.248
Q96C19	EF-hand domain-containing protein D2	EFHD2	0.012	0.145	0.248
Q9H7N4	Splicing factor, arginine/serine-rich 19	SCAF1	0.042	0.012	0.247
H0YD64	Solute carrier family 43 member 3 (Fragment)	SLC43A3	0.229	0.262	0.246
H0Y488	AT-rich interactive domain-containing protein 1A (Fragment)	ARID1A		-0.104	0.246
J3KQT3	Piezo-type mechanosensitive ion channel component 1 (Fragment)	PIEZO1			0.245
Q16777	Histone H2A type 2-C	HIST2H2AC			0.245
P46060	Ran GTPase-activating protein 1	RANGAP1	-0.012	-0.004	0.244
Q14997	Proteasome activator complex subunit 4	PSME4	-0.048	-0.018	0.243
Q92896	Golgi apparatus protein 1	GLG1	-0.261	0.355	0.243
P67809	Nuclease-sensitive element-binding protein 1	YBX1	-0.103	-0.059	0.242
Q14156-3	Isoform 3 of Protein EFR3 homolog A	EFR3A			0.242
Q96EL3	39S ribosomal protein L53, mitochondrial	MRPL53	-0.101		0.240
Q7Z7E8	Ubiquitin-conjugating enzyme E2 Q1	UBE2Q1	0.010	-0.135	0.240
P35611-2	Isoform 2 of Alpha-adducin	ADD1		0.207	0.240
Q9BR76	Coronin-1B	CORO1B	-0.175	0.022	0.239
Q8NEW0	Zinc transporter 7	SLC30A7		0.113	0.237
O75083	WD repeat-containing protein 1	WDR1	0.011	0.056	0.237
P41743	Protein kinase C iota type	PRKCI	-0.069	-0.181	0.237
Q9NX24	H/ACA ribonucleoprotein complex subunit 2	NHP2		0.096	0.236
P49748	Very long-chain specific acyl-CoA dehydrogenase, mitochondrial	ACADVL	-0.324	-0.097	0.236
P26641	Elongation factor 1-gamma	EEF1G	-0.163	-0.142	0.235
P61513	60S ribosomal protein L37a	RPL37A	-0.123	-0.088	0.234
Q14435	Polypeptide N-acetylgalactosaminyltransferase 3	GALNT3	-0.157		0.234
Q9H4M9	EH domain-containing protein 1	EHD1	-0.069	0.023	0.233
P33993	DNA replication licensing factor MCM7	MCM7	0.052	-0.003	0.233
B4DST5	Tyrosine-protein phosphatase non-receptor type 23	PTPN23	0.100	-0.142	0.233
F5H4L7	Vacuolar protein sorting-associated protein 26A	VPS26A	-0.157	0.147	0.232
Q8WXD5	Gem-associated protein 6	GEMIN6	-0.005		0.232
Q8IZW8	Tensin-4	TNS4			0.232
Q9Y5A9-2	Isoform 2 of YTH domain family protein 2	YTHDF2	-0.126	-0.078	0.232
P12814	Alpha-actinin-1	ACTN1	0.025	0.182	0.231
P30405	Peptidyl-prolyl cis-trans isomerase F, mitochondrial	PPIF	-0.210	-0.184	0.231
P51991-2	Isoform 2 of Heterogeneous nuclear ribonucleoprotein A3	HNRNPA3	0.023	-0.661	0.231
C9JEL3	Eukaryotic translation initiation factor 4E type 2 (Fragment)	EIF4E2		0.237	0.230
E2QRH7	Elongation of very long chain fatty acids protein 5	ELOVL5	0.000	0.031	0.230
Q9BUF5	Tubulin beta-6 chain	TUBB6	0.011	-0.066	0.230
B7Z2L7	TOM1-like protein 2	TOM1L2			0.229
P25205	DNA replication licensing factor MCM3	MCM3	-0.007	-0.059	0.228
Q9Y3B3-2	Isoform 2 of Transmembrane emp24 domain-containing protein 7	TMED7	-0.001	0.104	0.228
A6NNI4	CD9 antigen	CD9	0.079	0.228	0.228
Q658Y4	Protein FAM91A1	FAM91A1	-0.065	-0.028	0.226
Q9BW04	Specifically androgen-regulated gene protein	SARG	0.031		0.226
P11166	Solute carrier family 2, facilitated glucose transporter member 1	SLC2A1	-0.073	-0.316	0.225
O60678	Protein arginine N-methyltransferase 3	PRMT3	-0.137		0.225
Q96J02-3	Isoform 3 of E3 ubiquitin-protein ligase Itchy homolog	ITCH	0.104		0.225
P50443	Sulfate transporter	SLC26A2			0.224
Q9H0U4	Ras-related protein Rab-1B	RAB1B	-0.011	0.078	0.223
P22059	Oxysterol-binding protein 1	OSBP	0.095	-0.021	0.223
Q9UNX4	WD repeat-containing protein 3	WDR3	0.150	-0.148	0.220
Q9UKV8-2	Isoform 2 of Protein argonaute-2	AGO2	0.290	0.218	0.220
Q9UGM6-2	Isoform 2 of Tryptophan--tRNA ligase, mitochondrial	WARS2			0.220
E5RG71	RNA-binding Raly-like protein (Fragment)	RALYL	-0.079	-1.060	0.220
H7C440	DIS3-like exonuclease 2 (Fragment)	DIS3L2	-0.100		0.219
F8W7Q4	Protein FAM162A	FAM162A	-0.124	-0.016	0.219
Q9Y316	Protein MEMO1	MEMO1	-0.007	-0.057	0.218
Q9H3Z4-2	Isoform 2 of DnaJ homolog subfamily C member 5	DNAJC5	-0.066	0.011	0.218
B4E1G1	Derlin-1	DERL1	0.062	0.007	0.218
P82933	28S ribosomal protein S9, mitochondrial	MRPS9	0.018	0.152	0.218
P48307	Tissue factor pathway inhibitor 2	TFPI2			0.217
E7EVH9	Pseudouridine-5'-monophosphatase (Fragment)	HDHD1		0.070	0.216
Q16851-2	Isoform 2 of UTP--glucose-1-phosphate uridylyltransferase	UGP2	0.123	0.137	0.216
Q92759	General transcription factor IIH subunit 4	GTF2H4	0.084		0.215
O15355	Protein phosphatase 1G	PPM1G	0.023	-0.070	0.215
P01579	Interferon gamma	IFNG	-0.278	0.019	0.215
Q86UV5-2	Isoform 2 of Ubiquitin carboxyl-terminal hydrolase 48	USP48	0.017	0.028	0.214
O75821	Eukaryotic translation initiation factor 3 subunit G	EIF3G	-0.190	-0.011	0.214
Q9H7Z7	Prostaglandin E synthase 2	PTGES2	-0.034	0.118	0.213
P24534	Elongation factor 1-beta	EEF1B2	-0.142	-0.167	0.213

B1AZU4	HLA class I histocompatibility antigen, alpha chain F	HLA-F	0.859		0.212
Q9NRG9-2	Isoform 2 of Aladin	AAAS	0.099	0.062	0.211
P30154	Serine/threonine-protein phosphatase 2A 65 kDa regulatory subunit A beta isoforr	PPP2R1B	-0.020	-0.004	0.211
P00414	Cytochrome c oxidase subunit 3	MT-CO3	0.047	0.040	0.210
P68032	Actin, alpha cardiac muscle 1	ACTC1	-0.003	0.225	0.210
A6NKV8	Polyadenylate-binding protein-interacting protein 1	PAIP1	-0.033	-0.113	0.210
O43399-2	Isoform 2 of Tumor protein D54	TPD52L2	-0.166	-0.070	0.209
O15294	UDP-N-acetylglucosamine--peptide N-acetylglucosaminyltransferase 110 kDa subu	OGT	0.009	0.008	0.209
Q92620	Pre-mRNA-splicing factor ATP-dependent RNA helicase PRP16	DHX38	-0.043	-0.012	0.208
Q96D46	60S ribosomal export protein NMD3	NMD3	0.089	0.231	0.206
Q9Y5X1	Sorting nexin-9	SNX9	-0.041	-0.223	0.206
P62995-3	Isoform 3 of Transformer-2 protein homolog beta	TRA2B	0.025	-0.270	0.206
P14618	Pyruvate kinase isozymes M1/M2	PKM	-0.046	0.002	0.205
Q9BVP2-2	Isoform 2 of Guanine nucleotide-binding protein-like 3	GNL3	0.013	-0.038	0.205
B4DUQ5	Mitochondrial folate transporter/carrier	SLC25A32	-0.019	-0.113	0.204
Q9H2J4	Phosducin-like protein 3	PDCL3	-0.424	-0.271	0.204
O00762-2	Isoform 2 of Ubiquitin-conjugating enzyme E2 C	UBE2C	-0.010	0.017	0.204
O15427	Monocarboxylate transporter 4	SLC16A3	-0.003		0.202
Q13162	Peroxiredoxin-4	PRDX4	-0.011	0.010	0.202
K7EIG1	Clustered mitochondria protein homolog	CLUH	-0.075	-0.046	0.201
P82664	28S ribosomal protein S10, mitochondrial	MRPS10	-0.060	-0.075	0.201
P17096	High mobility group protein HMG-I/HMG-Y	HMGA1	-0.441	-2.010	0.201
Q9UBM7	7-dehydrocholesterol reductase	DHCR7	0.153	-0.212	0.201
Q9NR12	PDZ and LIM domain protein 7	PDLIM7	0.267	-0.713	0.200
Q00688	Peptidyl-prolyl cis-trans isomerase FKBP3	FKBP3	-0.045	-0.031	0.200
P00167-2	Isoform 2 of Cytochrome b5	CYB5A		0.164	0.199
P36952	Serpin B5	SERPINB5	0.066		0.199
Q8NFQ8	Torsin-1A-interacting protein 2	TOR1AIP2	0.067	0.029	0.199
O60701-2	Isoform 2 of UDP-glucose 6-dehydrogenase	UGDH	0.163	-0.002	0.199
Q8WVY7	Ubiquitin-like domain-containing CTD phosphatase 1	UBLCP1	0.025	-0.135	0.198
Q6P3X3	Tetratricopeptide repeat protein 27	TTC27	-0.233		0.197
H0Y7C8	Torsin-1B (Fragment)	TOR1B	0.081		0.197
O15269	Serine palmitoyltransferase 1	SPTLC1	0.032	0.095	0.197
P54709	Sodium/potassium-transporting ATPase subunit beta-3	ATP1B3	-0.119	0.043	0.197
Q9NRX4	14 kDa phosphohistidine phosphatase	PHPT1	0.036		0.196
P55084	Trifunctional enzyme subunit beta, mitochondrial	HADHB	0.118	0.198	0.195
Q5TIH2	Vesicle transport protein SFT2B	SFT2D2		-0.015	0.195
P12429	Annexin A3	ANXA3	0.133		0.195
Q13907	Isopentenyl-diphosphate Delta-isomerase 1	IDI1	0.139	-0.103	0.194
Q15404	Ras suppressor protein 1	RSU1	0.167	-0.078	0.194
Q9P0J7	E3 ubiquitin-protein ligase KCMF1	KCMF1	0.006	-0.130	0.194
P06493	Cyclin-dependent kinase 1	CDK1	-0.027	-0.043	0.193
F5GWR6	Homeobox protein cut-like 2 (Fragment)	CUX2	-0.152	-0.313	0.193
P13995	Bifunctional methylenetetrahydrofolate dehydrogenase/cyclohydrolase, mitochond	MTHFD2	-0.036	-0.069	0.193
Q9UH99	SUN domain-containing protein 2	SUN2	0.090	0.056	0.193
Q14978	Nucleolar and coiled-body phosphoprotein 1	NOLC1	-0.070	-0.299	0.192
P02794	Ferritin heavy chain	FTH1	-0.018	0.182	0.192
F5H365	Protein transport protein Sec23A	SEC23A	0.055	-0.082	0.192
Q16719	Kynureninase	KYNU			0.191
Q6UN15-3	Isoform 3 of Pre-mRNA 3'-end-processing factor FIP1	FIP1L1	0.042	0.095	0.191
P33992	DNA replication licensing factor MCM5	MCM5	0.014	0.021	0.191
Q9UNS2-2	Isoform 2 of COP9 signalosome complex subunit 3	COPS3	-0.176	0.002	0.190
P63241	Eukaryotic translation initiation factor 5A-1	EIF5A	-0.057	-0.092	0.190
Q9BSL1	Ubiquitin-associated domain-containing protein 1	UBAC1	0.021		0.189
O14976	Cyclin-G-associated kinase	GAK	0.484	0.041	0.189
P01024	Complement C3	C3	0.121	0.925	0.189
Q9BZG8-3	Isoform 3 of Diphthamide biosynthesis protein 1	DPH1	-0.375	-0.086	0.189
Q8NFT6-2	Isoform 2 of Protein DBF4 homolog B	DBF4B			0.188
O15460-2	Isoform IIa of Prolyl 4-hydroxylase subunit alpha-2	P4HA2	-0.145	0.342	0.188
E9PHA2	Condensin complex subunit 2	NCAPH	-0.075	-0.026	0.188
P30533	Alpha-2-macroglobulin receptor-associated protein	LRPAP1	0.089	-0.199	0.187
Q15645	Pachytene checkpoint protein 2 homolog	TRIP13	-0.056		0.187
F5GWX5	Chromodomain-helicase-DNA-binding protein 4	CHD4	0.009	-0.012	0.187
B4E2V5	Erythrocyte band 7 integral membrane protein	STOM			0.187
O75116	Rho-associated protein kinase 2	ROCK2	0.181	0.096	0.187
O14979-3	Isoform 3 of Heterogeneous nuclear ribonucleoprotein D-like	HNRPDL	-0.030	-0.681	0.186
P49773	Histidine triad nucleotide-binding protein 1	HINT1	-0.110	-0.059	0.186
B7ZKK9	PPP2R5E protein	PPP2R5E	0.025	0.175	0.186
P17844	Probable ATP-dependent RNA helicase DDX5	DDX5	-0.037	-0.130	0.186
Q9NRY4	Rho GTPase-activating protein 35	ARHGAP35			0.186
Q9BY44	Eukaryotic translation initiation factor 2A	EIF2A	-0.153	-0.052	0.186
F8VSZ4	Plexin-A1	PLXNA1	-0.165	0.048	0.185
Q9H2W6	39S ribosomal protein L46, mitochondrial	MRPL46	0.264		0.185
B4DU75	U3 small nucleolar RNA-associated protein 15 homolog	UTP15			0.185
Q70UQ0-2	Isoform 2 of Inhibitor of nuclear factor kappa-B kinase-interacting protein	IKBIP			0.185
P07686	Beta-hexosaminidase subunit beta	HEXB	0.002	0.079	0.184
G8JLK7	Nucleolar protein 6	NOL6	0.039	-0.051	0.184
Q96CB9-2	Isoform 2 of 5-methylcytosine rRNA methyltransferase NSUN4	NSUN4	0.296		0.183
O95394	Phosphoacetylglucosamine mutase	PGM3	0.004	0.036	0.183
Q9UHD1	Cysteine and histidine-rich domain-containing protein 1	CHORDC1	-0.090	-0.125	0.182
E9PF19	Transducin beta-like protein 2	TBL2	-0.032	0.478	0.181
Q9P035	Very-long-chain (3R)-3-hydroxyacyl-[acyl-carrier protein] dehydratase 3	PTPLAD1	0.031	-0.057	0.180
O94855	Protein transport protein Sec24D	SEC24D			0.179
Q6P158	Putative ATP-dependent RNA helicase DHX57	DHX57	-0.113	0.018	0.179
Q13433	Zinc transporter ZIP6	SLC39A6			0.179
O94967-2	Isoform 2 of WD repeat-containing protein 47	WDR47		0.013	0.178
Q9BV20	Methylthioribose-1-phosphate isomerase	MRI1	0.109	-0.098	0.178

H3BUL2	COMM domain-containing protein 4	COMMD4			0.178
P46734-2	Isoform 1 of Dual specificity mitogen-activated protein kinase kinase 3	MAP2K3	-0.025	0.140	0.177
Q15021	Condensin complex subunit 1	NCAPD2	-0.079	-0.090	0.177
P09525	Annexin A4	ANXA4	0.270	0.170	0.177
P14866	Heterogeneous nuclear ribonucleoprotein L	HNRNPL	-0.073	-0.494	0.177
Q8IXB1-2	Isoform 2 of DnaJ homolog subfamily C member 10	DNAJC10	0.211		0.176
E9PIY1	Protein kinase C and casein kinase substrate in neurons protein 3	PACSIN3	-0.090		0.176
P41567	Eukaryotic translation initiation factor 1	EIF1	-0.103	-0.085	0.176
P68104	Elongation factor 1-alpha 1	EEF1A1	-0.032	-0.107	0.176
Q99459	Cell division cycle 5-like protein	CDC5L	-0.011	-0.253	0.175
P07355	Annexin A2	ANXA2	0.108	0.145	0.175
Q6NVY1	3-hydroxyisobutyryl-CoA hydrolase, mitochondrial	HIBCH	0.097		0.175
B4DDF4	Calponin-2	CNN2	-0.291	-0.077	0.175
P22626	Heterogeneous nuclear ribonucleoproteins A2/B1	HNRNPA2B1	-0.014	-0.787	0.174
P00750-4	Isoform 4 of Tissue-type plasminogen activator	PLAT			0.174
Q9BUQ8	Probable ATP-dependent RNA helicase DDX23	DDX23		-0.070	0.174
P07996	Thrombospondin-1	THBS1		0.125	0.174
P49755	Transmembrane emp24 domain-containing protein 10	TMED10	-0.023	0.015	0.174
Q9UKG1	DCC-interacting protein 13-alpha	APPL1	0.193	-0.059	0.174
P47895	Aldehyde dehydrogenase family 1 member A3	ALDH1A3	-0.168		0.173
Q8NHH9-3	Isoform 3 of Atlastin-2	ATL2	0.009	0.094	0.173
P40939	Trifunctional enzyme subunit alpha, mitochondrial	HADHA	0.104	0.124	0.171
H0YA70	Multiple C2 and transmembrane domain-containing protein 1 (Fragment)	MCTP1		0.159	0.171
P04181	Ornithine aminotransferase, mitochondrial	OAT	0.145	-0.060	0.171
P21397	Amine oxidase [flavin-containing] A	MAOA			0.170
O14773	Tripeptidyl-peptidase 1	TPP1	0.153	0.273	0.170
J3KTG7	Transcription elongation factor, mitochondrial	TEFM			0.170
P23528	Cofilin-1	CFL1	-0.076	-0.036	0.169
Q5SSJ5	Heterochromatin protein 1-binding protein 3	HP1BP3	0.120	0.013	0.168
M0R050	Exosome complex component RRP46	EXOSC5	-0.052	0.078	0.167
H7C4J4	Armadillo repeat-containing protein 8 (Fragment)	ARMC8			0.167
Q14573	Inositol 1,4,5-trisphosphate receptor type 3	ITPR3	0.146		0.167
O14617-5	Isoform 5 of AP-3 complex subunit delta-1	AP3D1	0.046	-0.052	0.166
Q8IUE6	Histone H2A type 2-B	HIST2H2AB		-1.234	0.166
Q09028-3	Isoform 3 of Histone-binding protein RBBP4	RBBP4	-0.147	-0.238	0.165
Q86TI2-4	Isoform 3 of Dipeptidyl peptidase 9	DPP9	0.348	0.143	0.165
E9PKG1	Protein arginine N-methyltransferase 1	PRMT1	-0.093	-0.059	0.164
E7ENJ6	AP-1 complex subunit mu-1	AP1M1	0.029	0.015	0.164
F8VZC8	Transporter	SLC6A15			0.164
Q9Y3A4	Ribosomal RNA-processing protein 7 homolog A	RRP7A	-0.046		0.164
Q99959-2	Isoform 1 of Plakophilin-2	PKP2	-0.199		0.164
Q01082	Spectrin beta chain, non-erythrocytic 1	SPTBN1	-0.055	-0.014	0.164
P46199	Translation initiation factor IF-2, mitochondrial	MTIF2	0.030	0.127	0.164
O94979-6	Isoform 6 of Protein transport protein Sec31A	SEC31A	-0.084	0.027	0.163
E5RH51	Small integral membrane protein 12	SMIM12			0.163
Q9BSH4	Translational activator of cytochrome c oxidase 1	TACO1	0.014	0.030	0.163
P80188	Neutrophil gelatinase-associated lipocalin	LCN2	0.122		0.163
P33991	DNA replication licensing factor MCM4	MCM4	-0.002	-0.027	0.162
Q5GLZ8-6	Isoform 6 of Probable E3 ubiquitin-protein ligase HERC4	HERC4	0.140	0.251	0.162
O95470	Sphingosine-1-phosphate lyase 1	SGPL1	0.071	0.057	0.162
Q13813-3	Isoform 3 of Spectrin alpha chain, non-erythrocytic 1	SPTAN1	-0.035	0.017	0.162
P11940	Polyadenylate-binding protein 1	PABPC1	-0.162	-0.166	0.162
P00338	L-lactate dehydrogenase A chain	LDHA	-0.216	-0.055	0.162
P27348	14-3-3 protein theta	YWHAQ	-0.044	-0.061	0.162
P05556	Integrin beta-1	ITGB1	-0.015	-0.163	0.161
Q14789	Golgin subfamily B member 1	GOLGB1	-0.087	0.108	0.161
Q7L576	Cytoplasmic FMR1-interacting protein 1	CYFIP1	0.021	-0.063	0.161
Q7LG56-2	Isoform 2 of Ribonucleoside-diphosphate reductase subunit M2 B	RRM2B			0.161
P18031	Tyrosine-protein phosphatase non-receptor type 1	PTPN1	0.024	0.081	0.161
P43304	Glycerol-3-phosphate dehydrogenase, mitochondrial	GPD2	0.016	0.004	0.161
D6R9P3	Heterogeneous nuclear ribonucleoprotein A/B	HNRNPAB	-0.025	-0.451	0.160
P07437	Tubulin beta chain	TUBB	-0.019	0.020	0.160
Q8NGA1	Olfactory receptor 1M1	OR1M1	-0.059	0.039	0.160
Q9HCN4-4	Isoform 4 of GPN-loop GTPase 1	GPN1		0.289	0.159
P60891	Ribose-phosphate pyrophosphokinase 1	PRPS1	-0.068	-0.047	0.159
E9PQA5	Zinc finger protein-like 1 (Fragment)	ZFPL1	0.064	0.018	0.159
Q9Y490	Talin-1	TLN1	-0.016	0.162	0.159
Q53H82	Beta-lactamase-like protein 2	LACTB2	0.024	0.087	0.159
Q05655	Protein kinase C delta type	PRKCD	-0.080		0.158
O75506	Heat shock factor-binding protein 1	HSBP1	-0.183	-0.227	0.158
A8MUB1	Tubulin alpha-4A chain	TUBA4A	-0.052	-0.032	0.158
Q9ULA0	Aspartyl aminopeptidase	DNPEP	0.008	0.221	0.157
P08758	Annexin A5	ANXA5	0.031	0.045	0.157
G3V1R5	Nardilysin	NRD1	0.013	-0.110	0.157
F8VY35	Nucleosome assembly protein 1-like 1 (Fragment)	NAP1L1	-0.228	-0.182	0.157
Q15061	WD repeat-containing protein 43	WDR43	-0.168	-0.512	0.157
P48507	Glutamate--cysteine ligase regulatory subunit	GCLM	-0.210	-0.036	0.156
Q8TCT9-5	Isoform 5 of Minor histocompatibility antigen H13	HM13	0.030	0.085	0.156
F5H6I7	Atlastin-3	ATL3	0.092	0.058	0.156
O60488-2	Isoform Short of Long-chain-fatty-acid--CoA ligase 4	ACSL4	-0.029		0.156
P20338	Ras-related protein Rab-4A	RAB4A		0.042	0.156
B4DWP2	Prostasin	PRSS8			0.155
P49736	DNA replication licensing factor MCM2	MCM2	0.020	-0.008	0.154
O00767	Acyl-CoA desaturase	SCD	-0.125	0.216	0.154
Q9Y697-2	Isoform Cytoplasmic of Cysteine desulfurase, mitochondrial	NFS1	-0.108	-0.039	0.154
Q9UID3-2	Isoform 2 of Vacuolar protein sorting-associated protein 51 homolog	VPS51	0.005		0.154
P27144	GTP:AMP phosphotransferase AK4, mitochondrial	AK4	0.131		0.153

Q6IAN0	Dehydrogenase/reductase SDR family member 7B	DHRS7B	0.486		0.153
E9PGT3	Ribosomal protein S6 kinase	RPS6KA1	0.064	0.042	0.152
Q9NR45	Sialic acid synthase	NANS	-0.102	0.040	0.152
R4GND1	Ubiquitin-conjugating enzyme E2 E3	UBE2E3	0.006	0.031	0.152
Q96J01	THO complex subunit 3	THOC3	0.087	-0.062	0.151
Q6WCQ1-3	Isoform 3 of Myosin phosphatase Rho-interacting protein	MPRIP	0.299	-0.119	0.151
P56182	Ribosomal RNA processing protein 1 homolog A	RRP1	0.034	-0.079	0.151
P01040	Cystatin-A	CSTA			0.150
P43246	DNA mismatch repair protein Msh2	MSH2	0.015	-0.044	0.150
Q15785	Mitochondrial import receptor subunit TOM34	TOMM34	-0.143		0.150
D3YTI9	Tapasin	TAPBP	0.455	0.312	0.150
Q16576	Histone-binding protein RBBP7	RBBP7	-0.024	-0.075	0.150
O75146	Huntingtin-interacting protein 1-related protein	HIP1R	0.185	0.067	0.150
P18754	Regulator of chromosome condensation	RCC1	-0.204	-0.516	0.150
P30520	Adenylosuccinate synthetase isozyme 2	ADSS	0.131	-0.040	0.149
O43390	Heterogeneous nuclear ribonucleoprotein R	HNRNPR	-0.049	-0.336	0.149
P53803	DNA-directed RNA polymerases I, II, and III subunit RPABC4	POLR2K	-0.094	-0.382	0.149
O75569	Interferon-inducible double stranded RNA-dependent protein kinase activator A	PRKRA	0.017	-0.412	0.149
Q14247	Src substrate cortactin	CTTN	-0.010	-0.104	0.149
Q32MZ4-3	Isoform 3 of Leucine-rich repeat flightless-interacting protein 1	LRRFIP1	0.007	0.002	0.148
Q9Y2H0-3	Isoform 3 of Disks large-associated protein 4	DLGAP4	0.067		0.148
E9PL01	Signal peptidase complex subunit 2	SPCS2	-0.003	0.063	0.148
Q14566	DNA replication licensing factor MCM6	MCM6	-0.006	-0.074	0.147
Q9ULX3	RNA-binding protein NOB1	NOB1	0.121	0.273	0.147
Q14134-2	Isoform Beta of Tripartite motif-containing protein 29	TRIM29	-0.170		0.146
P06703	Protein S100-A6	S100A6	0.111	-0.114	0.146
Q13769	THO complex subunit 5 homolog	THOC5	0.090	-0.149	0.146
P18085	ADP-ribosylation factor 4	ARF4	0.002	-0.052	0.146
I3L543	NmrA-like family domain-containing protein 1 (Fragment)	NMRAL1	-0.252		0.145
P33176	Kinesin-1 heavy chain	KIF5B	-0.197	-0.165	0.145
Q9UBB4	Ataxin-10	ATXN10	-0.100	-0.029	0.145
P13639	Elongation factor 2	EEF2	-0.038	-0.025	0.145
B2WTI3	Bifunctional arginine demethylase and lysyl-hydroxylase JMJD6	JMJD6		-0.071	0.145
Q9Y2Z0	Suppressor of G2 allele of SKP1 homolog	SUGT1	0.032	-0.010	0.145
P09497-2	Isoform Non-brain of Clathrin light chain B	CLTB	0.010	0.055	0.144
Q8IWV7	E3 ubiquitin-protein ligase UBR1	UBR1	-0.160		0.143
P47897	Glutamine--tRNA ligase	QARS	0.009	-0.022	0.143
P62633-2	Isoform 2 of Cellular nucleic acid-binding protein	CNBP	-0.012	-0.115	0.143
Q03135	Caveolin-1	CAV1	0.003		0.143
C9JLV4	Apoptotic protease-activating factor 1	APAF1	-0.103	-0.315	0.143
H7C003	Protein phosphatase 1 regulatory subunit 7 (Fragment)	PPP1R7	0.178	0.139	0.143
B7Z641	V-type proton ATPase 116 kDa subunit a isoform 1	ATP6V0A1	-0.032	0.194	0.143
P12955	Xaa-Pro dipeptidase	PEPD	-0.052	0.091	0.143
Q9UGC7-4	Isoform 4 of Peptide chain release factor 1-like, mitochondrial	MTRF1L	-0.225	-1.029	0.142
Q14444-2	Isoform 2 of Caprin-1	CAPRIN1	-0.212	-0.197	0.142
Q15691	Microtubule-associated protein RP/EB family member 1	MAPRE1	-0.059	-0.086	0.142
Q7L0Y3	Mitochondrial ribonuclease P protein 1	TRMT10C	0.026	-0.128	0.142
P60709	Actin, cytoplasmic 1	ACTB	0.033	0.170	0.142
P37059	Estradiol 17-beta-dehydrogenase 2	HSD17B2			0.142
Q9HCE7-2	Isoform Short of E3 ubiquitin-protein ligase SMURF1	SMURF1			0.141
Q9BTC0	Death-inducer obliterator 1	DIDO1		0.194	0.141
Q9Y5S2	Serine/threonine-protein kinase MRCK beta	CDC42BPB		0.049	0.141
B4DNY1	RNA-binding protein 6	RBM6	-0.083	-0.130	0.140
Q9C0D9	Ethanolaminephosphotransferase 1	EPT1	0.062	0.062	0.140
Q14192	Four and a half LIM domains protein 2	FHL2	0.116	0.139	0.140
Q9NQ88	Fructose-2,6-bisphosphatase TIGAR	TIGAR	0.144		0.139
J3KRJ6	Hydrocephalus-inducing protein homolog (Fragment)	HYDIN	-0.231	-0.277	0.138
P07339	Cathepsin D	CTSD	-0.233	-0.043	0.138
P51532-5	Isoform 5 of Transcription activator BRG1	SMARCA4	0.227	-0.014	0.138
H0YI92	Protein lin-7 homolog A (Fragment)	LIN7A	-0.004	-0.200	0.138
Q9NSV4-7	Isoform 7 of Protein diaphanous homolog 3	DIAPH3	-0.052	0.143	0.138
Q9UM54-5	Isoform 5 of Unconventional myosin-VI	MYO6	0.066	0.188	0.138
Q9NR31	GTP-binding protein SAR1a	SAR1A	-0.032	0.013	0.138
H0YCY6	FAD-AMP lyase (cyclizing) (Fragment)	DAK	0.355		0.137
Q96IJ6	Mannose-1-phosphate guanyltransferase alpha	GMPPA	-0.022		0.137
Q9Y3D5	28S ribosomal protein S18c, mitochondrial	MRPS18C	-0.113	0.083	0.136
P26572	Alpha-1,3-mannosyl-glycoprotein 2-beta-N-acetylglucosaminyltransferase	MGAT1	-0.022	-0.011	0.136
Q9H853	Putative tubulin-like protein alpha-4B	TUBA4B	-0.011	-0.099	0.136
Q9BV68-2	Isoform 2 of RING finger protein 126	RNF126			0.136
Q6P9B6	TLD domain-containing protein KIAA1609	KIAA1609	0.105		0.135
Q16222-3	Isoform 3 of UDP-N-acetylhexosamine pyrophosphorylase	UAP1	-0.246	-0.103	0.135
P42677	40S ribosomal protein S27	RPS27	-0.193	-0.152	0.135
Q9UHD8-7	Isoform 7 of Septin-9	SEPT9	-0.011	0.006	0.135
Q15582	Transforming growth factor-beta-induced protein ig-h3	TGFBI	0.271		0.134
P49005	DNA polymerase delta subunit 2	POLD2	-0.044	0.029	0.134
O75607	Nucleoplasmin-3	NPM3	-0.088	-0.209	0.133
A8MTT3	HCG1988162	hCG_1988162	-0.279	-0.004	0.133
Q9UQE7	Structural maintenance of chromosomes protein 3	SMC3	-0.071	-0.100	0.132
Q7L2H7	Eukaryotic translation initiation factor 3 subunit M	EIF3M	-0.053	-0.005	0.132
O95758-7	Isoform 7 of Polypyrimidine tract-binding protein 3	PTBP3	-0.279	-0.221	0.132
Q9Y3B4	Pre-mRNA branch site protein p14	SF3B14	0.012	0.032	0.132
Q9NY33	Dipeptidyl peptidase 3	DPP3	-0.002	0.023	0.132
Q9UM00-2	Isoform 2 of Transmembrane and coiled-coil domain-containing protein 1	TMCO1	-0.113	0.126	0.132
E9PCT3	Caveolin	CAV2	-0.022		0.132
P04350	Tubulin beta-4A chain	TUBB4A	-0.024	0.077	0.132
P04075	Fructose-bisphosphate aldolase A	ALDOA	-0.027	-0.022	0.132
Q5J8M3	ER membrane protein complex subunit 4	EMC4	0.096	0.203	0.131

Q8NE86-3	Isoform 3 of Calcium uniporter protein, mitochondrial	MCU	0.027	0.570	0.131
Q00610-2	Isoform 2 of Clathrin heavy chain 1	CLTC	0.068	0.047	0.131
P49257	Protein ERGIC-53	LMAN1	0.051	0.065	0.131
Q9UI26	Importin-11	IPO11	-0.215	0.045	0.131
H0YDB7	Ras-related protein Rab-38 (Fragment)	RAB38			0.130
P43034	Platelet-activating factor acetylhydrolase IB subunit alpha	PAFAH1B1	-0.007	-0.004	0.130
Q9BYC8	39S ribosomal protein L32, mitochondrial	MRPL32	-0.006	0.065	0.130
P13693	Translationally-controlled tumor protein	TPT1	-0.149	-0.282	0.130
O75844	CAAX prenyl protease 1 homolog	ZMPSTE24	-0.147	-0.112	0.129
Q96HV5	Transmembrane protein 41A	TMEM41A	-0.015		0.129
Q16787-1	Isoform 1 of Laminin subunit alpha-3	LAMA3	-0.030		0.129
P19105	Myosin regulatory light chain 12A	MYL12A	0.021	-0.133	0.129
P62913-2	Isoform 2 of 60S ribosomal protein L11	RPL11	-0.105	-0.118	0.128
Q9NYU2-2	Isoform 2 of UDP-glucose:glycoprotein glucosyltransferase 1	UGGT1	0.100	0.192	0.128
E9PEZ3	Protein diaphanous homolog 1	DIAPH1	-0.028	-0.049	0.128
O95372	Acyl-protein thioesterase 2	LYPLA2	0.041	-0.090	0.128
Q14019	Coactosin-like protein	COTL1	0.059	0.068	0.127
M0R2C2	Neuropathy target esterase (Fragment)	PNPLA6			0.127
Q9Y676	28S ribosomal protein S18b, mitochondrial	MRPS18B	-0.094	-0.023	0.127
P51858	Hepatoma-derived growth factor	HDGF	-0.033	-0.032	0.126
P07099	Epoxide hydrolase 1	EPHX1	-0.199	-0.067	0.126
P07602	Proactivator polypeptide	PSAP	-0.183	0.062	0.126
Q9BSJ2	Gamma-tubulin complex component 2	TUBGCP2	-0.047	0.047	0.126
H0YBN0	Vacuolar protein sorting-associated protein 37A (Fragment)	VPS37A	0.068	0.254	0.126
O15230	Laminin subunit alpha-5	LAMA5	0.061	0.055	0.126
P07947	Tyrosine-protein kinase Yes	YES1	-0.142		0.125
H3BMZ4	Autophagy-related protein 101 (Fragment)	C12orf44			0.125
P57088	Transmembrane protein 33	TMEM33	-0.102	0.093	0.125
P41240	Tyrosine-protein kinase CSK	CSK			0.125
P60981	Destrin	DSTN	0.018	-0.091	0.125
Q15257-2	Isoform 1 of Serine/threonine-protein phosphatase 2A activator	PPP2R4	0.005	0.121	0.124
Q14651	Plastin-1	PLS1	0.021	0.155	0.124
P61254	60S ribosomal protein L26	RPL26	-0.248	-0.227	0.124
Q9BWJ5	Splicing factor 3B subunit 5	SF3B5	-0.143	-0.338	0.124
O95347	Structural maintenance of chromosomes protein 2	SMC2	-0.064	-0.004	0.124
Q9BZX2	Uridine-cytidine kinase 2	UCK2	0.079	-0.106	0.124
Q2M2Z5-5	Isoform 5 of Centrosomal protein kizuna	PLK1S1			0.123
P47989	Xanthine dehydrogenase/oxidase	XDH		0.106	0.122
E5RHW4	Erlin-2 (Fragment)	ERLIN2	-0.032	0.227	0.122
H7C0V0	Uncharacterized protein C2orf47, mitochondrial (Fragment)	C2orf47	0.159		0.121
Q9NTK5	Obg-like ATPase 1	OLA1	-0.076	-0.076	0.121
O43865	Putative adenosylhomocysteinase 2	AHCYL1	-0.006	0.031	0.121
Q9NP79	Vacuolar protein sorting-associated protein VTA1 homolog	VTA1	-0.010	0.241	0.121
H0YBL1	Inositol monophosphatase 1 (Fragment)	IMPA1	0.183	0.190	0.121
Q9BRP4-2	Isoform 2 of Proteasomal ATPase-associated factor 1	PAAF1	0.027		0.120
P31749	RAC-alpha serine/threonine-protein kinase	AKT1		0.079	0.120
P53582	Methionine aminopeptidase 1	METAP1	-0.104	-0.328	0.120
O60763	General vesicular transport factor p115	USO1	0.147	0.083	0.120
P54105	Methylosome subunit pICln	CLNS1A	0.059	0.049	0.119
P06756-3	Isoform 3 of Integrin alpha-V	ITGAV	0.173		0.118
P15374	Ubiquitin carboxyl-terminal hydrolase isozyme L3	UCHL3	-0.130	-0.256	0.118
P11413	Glucose-6-phosphate 1-dehydrogenase	G6PD	-0.438	-0.092	0.118
C9J2I0	Arf-GAP domain and FG repeat-containing protein 1 (Fragment)	AGFG1	0.078	0.042	0.118
Q9BQE3	Tubulin alpha-1C chain	TUBA1C	-0.029	-0.073	0.117
F5H223	Dynactin subunit 2	DCTN2	0.059	0.055	0.117
Q13151	Heterogeneous nuclear ribonucleoprotein A0	HNRNPA0	-0.053	-0.519	0.117
P39023	60S ribosomal protein L3	RPL3	-0.127	-0.087	0.117
Q6UWP7-3	Isoform 3 of Lysocardiolipin acyltransferase 1	LCLAT1	-0.012	-0.292	0.117
B7Z6B8	2,4-dienoyl-CoA reductase, mitochondrial	DECR1	0.210	0.083	0.117
Q5T3Q7	HEAT repeat-containing protein 1	HEATR1	-0.021	-0.127	0.117
B4DH61	Probable inactive protein kinase-like protein SgK071	C9orf96	-0.151		0.117
G3V161	Kelch repeat and BTB (POZ) domain containing 3, isoform CRA_a	KBTBD3			0.116
Q9BV86	N-terminal Xaa-Pro-Lys N-methyltransferase 1	NTMT1	-0.350		0.116
P27797	Calreticulin	CALR	-0.042	0.074	0.116
P61619	Protein transport protein Sec61 subunit alpha isoform 1	SEC61A1	-0.063	0.028	0.116
A8MXV4	Nucleoside diphosphate-linked moiety X motif 19, mitochondrial	NUDT19	-0.069	0.005	0.116
B4DDM2	Adenosine 3'-phospho 5'-phosphosulfate transporter 1	SLC35B2	-0.002		0.116
P00918	Carbonic anhydrase 2	CA2			0.115
P63172	Dynein light chain Tctex-type 1	DYNLT1	-0.042	0.138	0.115
Q15121	Astrocytic phosphoprotein PEA-15	PEA15	0.030	-0.015	0.115
F5H3P5	Actin-related protein 3	ACTR3	0.059	-0.047	0.115
O00232	26S proteasome non-ATPase regulatory subunit 12	PSMD12	-0.093	-0.030	0.115
P52888	Thimet oligopeptidase	THOP1	-0.014	-0.024	0.115
O95202	LETM1 and EF-hand domain-containing protein 1, mitochondrial	LETM1	-0.054	-0.011	0.115
P62195-2	Isoform 2 of 26S protease regulatory subunit 8	PSMC5	-0.075	-0.088	0.115
Q6NUQ4	Transmembrane protein 214	TMEM214	0.221	0.088	0.114
P49903	Selenide, water dikinase 1	SEPHS1	-0.178	-0.041	0.114
P05089-3	Isoform 3 of Arginase-1	ARG1		0.075	0.114
Q9NZJ7-2	Isoform 2 of Mitochondrial carrier homolog 1	MTCH1	0.002	0.320	0.114
Q9NQP4	Prefoldin subunit 4	PFDN4		-0.108	0.113
P51572	B-cell receptor-associated protein 31	BCAP31	0.131	0.088	0.113
Q5U5X0	Complex III assembly factor LYRM7	LYRM7	0.003	-0.081	0.113
O60869-2	Isoform 2 of Endothelial differentiation-related factor 1	EDF1	-0.383	-0.110	0.113
Q9P1F3	Costars family protein ABRACL	ABRACL	0.002	-0.193	0.113
P48059	LIM and senescent cell antigen-like-containing domain protein 1	LIMS1	0.006	0.079	0.113
O95232	Luc7-like protein 3	LUC7L3	-0.080	-0.114	0.112
B1AUU8	Epidermal growth factor receptor substrate 15	EPS15	0.154	0.299	0.112

P00387-2	Isoform 2 of NADH-cytochrome b5 reductase 3	CYB5R3	0.174	0.112	0.112
P68402	Platelet-activating factor acetylhydrolase IB subunit beta	PAFAH1B2	0.150	-0.059	0.111
P09651-3	Isoform 2 of Heterogeneous nuclear ribonucleoprotein A1	HNRNPA1	-0.101	-0.539	0.111
E5KLK1	Dynamin-like 120 kDa protein, mitochondrial	OPA1	-0.008	0.059	0.111
Q9BPX3	Condensin complex subunit 3	NCAPG	-0.085	-0.174	0.111
P47755	F-actin-capping protein subunit alpha-2	CAPZA2	-0.078	-0.021	0.110
Q07955	Serine/arginine-rich splicing factor 1	SRSF1	-0.060	-0.248	0.110
Q9BYN0	Sulfiredoxin-1	SRXN1	-0.230	-0.148	0.110
O14929	Histone acetyltransferase type B catalytic subunit	HAT1	-0.043	-0.049	0.110
E9PG39	Acylglycerol kinase, mitochondrial	AGK	0.012	0.134	0.110
B4DXZ6	Fragile X mental retardation syndrome-related protein 1	FXR1	-0.020	0.029	0.110
P04080	Cystatin-B	CSTB	0.029	-0.330	0.110
P23526	Adenosylhomocysteinase	AHCY	0.035	-0.055	0.110
Q96T76	MMS19 nucleotide excision repair protein homolog	MMS19	0.033	0.113	0.109
Q9BRA2	Thioredoxin domain-containing protein 17	TXNDC17	-0.107	-0.146	0.109
Q9UN86-2	Isoform B of Ras GTPase-activating protein-binding protein 2	G3BP2	-0.359	-0.172	0.109
Q5H9A7	Metalloproteinase inhibitor 1	TIMP1			0.109
Q14116-2	Isoform 2 of Interleukin-18	IL18	0.094		0.109
P24928	DNA-directed RNA polymerase II subunit RPB1	POLR2A	0.143	-0.075	0.108
Q15102	Platelet-activating factor acetylhydrolase IB subunit gamma	PAFAH1B3	0.153	0.048	0.108
O60256	Phosphoribosyl pyrophosphate synthase-associated protein 2	PRPSAP2	0.058	0.058	0.108
E9PQX9	Dr1-associated corepressor	DRAP1	-0.275	0.091	0.108
Q9H845	Acyl-CoA dehydrogenase family member 9, mitochondrial	ACAD9	-0.023	0.068	0.108
H7BYZ4	Thyroid adenoma-associated protein (Fragment)	THADA	0.106		0.108
Q96RP9	Elongation factor G, mitochondrial	GFM1	-0.130	-0.013	0.108
P15153	Ras-related C3 botulinum toxin substrate 2	RAC2			0.107
Q15286	Ras-related protein Rab-35	RAB35	-0.049	0.019	0.107
Q8WVC2	40S ribosomal protein S21	RPS21	-0.217	-0.492	0.107
B7ZBJ4	Calcium-binding protein 39-like	CAB39L			0.106
Q68DL0	Phosphatidylinositol 4,5-bisphosphate 3-kinase catalytic subunit beta isoform	DKFZp779K1237			0.106
P61201	COP9 signalosome complex subunit 2	COPS2	-0.132	-0.012	0.106
Q9UKI8-3	Isoform 3 of Serine/threonine-protein kinase tousled-like 1	TLK1			0.106
P78417	Glutathione S-transferase omega-1	GSTO1	0.003	0.068	0.106
P61586	Transforming protein RhoA	RHOA	-0.002	0.139	0.105
Q15024	Exosome complex component RRP42	EXOSC7	-0.054	-0.122	0.105
O60783	28S ribosomal protein S14, mitochondrial	MRPS14	0.074	0.149	0.105
P46940	Ras GTPase-activating-like protein IQGAP1	IQGAP1	0.048	0.106	0.105
P36551	Coproporphyrinogen-III oxidase, mitochondrial	CPOX	0.223	0.294	0.105
P31943	Heterogeneous nuclear ribonucleoprotein H	HNRNPH1	-0.112	-0.220	0.105
C9JFR7	Cytochrome c (Fragment)	CYCS	-0.083	-0.077	0.104
P22307-4	Isoform 4 of Non-specific lipid-transfer protein	SCP2	-0.125	-0.148	0.104
Q99584	Protein S100-A13	S100A13	0.012	0.205	0.104
Q96EA0	BET1 homolog	BET1			0.104
Q15717	ELAV-like protein 1	ELAVL1	0.007	-0.074	0.104
H3BV55	Phosphomannomutase 2	PMM2	0.166	0.049	0.103
P13984	General transcription factor IIF subunit 2	GTF2F2	-0.003	-0.086	0.103
O43665-2	Isoform 2 of Regulator of G-protein signaling 10	RGS10			0.103
P07204	Thrombomodulin	THBD			0.103
Q9UBT2	SUMO-activating enzyme subunit 2	UBA2	0.017	-0.018	0.103
P31153	S-adenosylmethionine synthase isoform type-2	MAT2A	0.015	0.001	0.103
P98175-4	Isoform 4 of RNA-binding protein 10	RBM10	0.174	0.055	0.102
Q15006	ER membrane protein complex subunit 2	EMC2	0.015	0.055	0.102
O00567	Nucleolar protein 56	NOP56	0.148	0.003	0.102
P62158	Calmodulin	CALM1	0.154	-0.077	0.102
P55735-2	Isoform 2 of Protein SEC13 homolog	SEC13	-0.063	0.054	0.101
Q8WU68-3	Isoform 3 of Splicing factor U2AF 26 kDa subunit	U2AF1L4			0.101
H7C1I0	Solute carrier family 35 member E1 (Fragment)	SLC35E1			0.101
Q9P2R3	Ankyrin repeat and FYVE domain-containing protein 1	ANKFY1	0.054	0.011	0.100
B1AKY9	Sodium/potassium-transporting ATPase subunit alpha-2	ATP1A2	-0.151		0.100
P15311	Ezrin	EZR	0.039	-0.019	0.100
O95782-2	Isoform B of AP-2 complex subunit alpha-1	AP2A1	0.060	0.116	0.099
P19404	NADH dehydrogenase [ubiquinone] flavoprotein 2, mitochondrial	NDUFV2	0.099	0.265	0.099
Q99598	Translin-associated protein X	TSNAX	0.030	-0.066	0.099
P53007	Tricarboxylate transport protein, mitochondrial	SLC25A1	0.138	-0.032	0.099
G3V1S6	Polypeptide N-acetylgalactosaminyltransferase 2 soluble form	GALNT2	-0.188	0.223	0.099
P52565	Rho GDP-dissociation inhibitor 1	ARHGDIA	0.066	-0.057	0.098
P37802	Transgelin-2	TAGLN2	0.077	-0.095	0.098
P09972	Fructose-bisphosphate aldolase C	ALDOC			0.098
Q9Y5Q9	General transcription factor 3C polypeptide 3	GTF3C3	0.032	0.030	0.097
R4GMU8	Ragulator complex protein LAMTOR5	LAMTOR5	-0.364		0.097
Q8NFH3	Nucleoporin Nup43	NUP43	0.106	-0.136	0.097
O95573	Long-chain-fatty-acid--CoA ligase 3	ACSL3	0.005	-0.060	0.097
Q52LJ0-2	Isoform 2 of Protein FAM98B	FAM98B	0.011	0.054	0.097
Q9BSJ8	Extended synaptotagmin-1	ESYT1	-0.076	-0.033	0.097
Q9ULE0-2	Isoform 2 of Protein WWC3	WWC3	-0.078	-0.120	0.097
Q8N543	2-oxoglutarate and iron-dependent oxygenase domain-containing protein 1	OGFOD1	0.085	-0.056	0.097
Q7Z7K6-2	Isoform 2 of Centromere protein V	CENPV	-0.024	-0.257	0.096
Q99536	Synaptic vesicle membrane protein VAT-1 homolog	VAT1	0.170		0.096
O15144	Actin-related protein 2/3 complex subunit 2	ARPC2	0.037	0.012	0.096
P04406	Glyceraldehyde-3-phosphate dehydrogenase	GAPDH	-0.049	-0.038	0.096
O15143	Actin-related protein 2/3 complex subunit 1B	ARPC1B	0.060	0.076	0.096
P00568	Adenylate kinase isoenzyme 1	AK1	-0.064	-0.026	0.096
O00151	PDZ and LIM domain protein 1	PDLIM1	0.044	-0.112	0.096
Q13362-2	Isoform Gamma-1 of Serine/threonine-protein phosphatase 2A 56 kDa regulatory	PPP2R5C	-0.089	0.084	0.095
F8VX55	Alpha-globin transcription factor CP2	TFCP2	-0.068	-0.163	0.095
P61019	Ras-related protein Rab-2A	RAB2A	-0.107	-0.020	0.094
Q9UJA5	tRNA (adenine(58)-N(1))-methyltransferase non-catalytic subunit TRM6	TRMT6	-0.066	0.077	0.094

O15145	Actin-related protein 2/3 complex subunit 3	ARPC3	0.033	-0.032	0.094
P30825	High affinity cationic amino acid transporter 1	SLC7A1	-0.178	-0.006	0.094
B7Z254	Protein disulfide-isomerase A6	PDIA6	0.068	0.066	0.094
O00505	Importin subunit alpha-3	KPNA3	0.038	-0.013	0.094
P00374	Dihydrofolate reductase	DHFR	-0.146	-0.089	0.094
O75306-2	Isoform 2 of NADH dehydrogenase [ubiquinone] iron-sulfur protein 2, mitochondr	NDUFS2	0.024	0.148	0.094
Q13409-6	Isoform 2F of Cytoplasmic dynein 1 intermediate chain 2	DYNC1I2	-0.032	0.009	0.094
P11498	Pyruvate carboxylase, mitochondrial	PC		0.055	0.094
Q14764	Major vault protein	MVP			0.093
Q00341	Vigilin	HDLBP	-0.032	-0.010	0.093
A1L0T0	Acetolactate synthase-like protein	ILVBL	0.002	0.151	0.093
E7EU42	Lysophospholipase	PLA2G4A			0.093
O43156	TELO2-interacting protein 1 homolog	TTI1			0.093
O15511	Actin-related protein 2/3 complex subunit 5	ARPC5	0.009	0.106	0.092
P22087	rRNA 2'-O-methyltransferase fibrillarin	FBL	0.125	-0.167	0.092
P62979	Ubiquitin-40S ribosomal protein S27a	RPS27A	-0.116	-0.100	0.092
O00264	Membrane-associated progesterone receptor component 1	PGRMC1	0.005	-0.099	0.092
P53621	Coatomer subunit alpha	COPA	-0.062	-0.019	0.092
Q02218	2-oxoglutarate dehydrogenase, mitochondrial	OGDH	0.024	0.181	0.092
P18669	Phosphoglycerate mutase 1	PGAM1	-0.057	-0.051	0.092
Q9UGV2	Protein NDRG3	NDRG3	-0.071	0.011	0.092
P48960-2	Isoform 2 of CD97 antigen	CD97	-0.074	0.041	0.092
Q9Y376	Calcium-binding protein 39	CAB39	-0.012	-0.022	0.092
F5H4G7	Importin subunit alpha	KPNA6	-0.011	-0.113	0.091
O00410	Importin-5	IPO5	-0.116	-0.046	0.090
Q92696	Geranylgeranyl transferase type-2 subunit alpha	RABGGTA	0.128		0.090
Q14203-3	Isoform 3 of Dynactin subunit 1	DCTN1	-0.016	0.066	0.090
O00116	Alkyldihydroxyacetonephosphate synthase, peroxisomal	AGPS	-0.038	-0.371	0.089
Q8TBQ9	Protein kish-A	TMEM167A	0.037		0.089
Q15165	Serum paraoxonase/arylesterase 2	PON2	0.214	0.419	0.089
Q56VL3	OCIA domain-containing protein 2	OCIAD2	-0.009	-0.204	0.089
Q9Y570	Protein phosphatase methylesterase 1	PPME1	0.065	-0.036	0.088
Q9UIJ7	GTP:AMP phosphotransferase AK3, mitochondrial	AK3	0.013	-0.084	0.088
K7EMW4	Nicalin	NCLN	0.056	0.025	0.088
H0YJ13	Protein C14orf64 (Fragment)	C14orf64			0.088
H7C2W9	60S ribosomal protein L31 (Fragment)	RPL31	-0.032	-0.168	0.088
J3KTE9	Cadherin-3	CDH3	0.019		0.087
P50570-2	Isoform 2 of Dynamin-2	DNM2	-0.014	0.120	0.087
P45880-2	Isoform 2 of Voltage-dependent anion-selective channel protein 2	VDAC2	0.005	-0.018	0.087
O75832	26S proteasome non-ATPase regulatory subunit 10	PSMD10	-0.124	0.002	0.087
F5H0B0	Tumor protein D52	TPD52	0.227	-0.184	0.087
P17900	Ganglioside GM2 activator	GM2A			0.086
Q5UIP0-2	Isoform 2 of Telomere-associated protein RIF1	RIF1	0.036	0.078	0.086
P35613-2	Isoform 2 of Basigin	BSG	-0.142	-0.035	0.086
Q9HC35	Echinoderm microtubule-associated protein-like 4	EML4	0.085	0.164	0.086
Q6P1N9	Putative deoxyribonuclease TATDN1	TATDN1	-0.082	-0.118	0.085
Q96CG1	ETF1 protein	ETF1	-0.098	-0.098	0.085
Q9Y285	Phenylalanine--tRNA ligase alpha subunit	FARSA	0.045	-0.110	0.085
Q14847	LIM and SH3 domain protein 1	LASP1	-0.117	-0.054	0.084
Q9BVL2-3	Isoform 3 of Nucleoporin p58/p45	NUPL1	-0.003	0.149	0.084
P07237	Protein disulfide-isomerase	P4HB	-0.239	-0.129	0.084
Q01968-2	Isoform B of Inositol polyphosphate 5-phosphatase OCRL-1	OCRL	-0.361		0.084
O43747	AP-1 complex subunit gamma-1	AP1G1	-0.048	0.029	0.084
Q4J6C6-4	Isoform 4 of Prolyl endopeptidase-like	PREPL	0.254		0.084
O14530	Thioredoxin domain-containing protein 9	TXNDC9	-0.035	-0.067	0.084
Q07960	Rho GTPase-activating protein 1	ARHGAP1	-0.015	0.072	0.083
K7EIG7	Unconventional myosin-Id	MYO1D	0.039		0.083
Q58FF6	Putative heat shock protein HSP 90-beta 4	HSP90AB4P	-0.293	0.042	0.083
J3QQX3	NADPH:adrenodoxin oxidoreductase, mitochondrial	FDXR			0.083
Q6IA86-2	Isoform 2 of Elongator complex protein 2	ELP2	0.088	0.141	0.083
Q99816	Tumor susceptibility gene 101 protein	TSG101			0.083
Q9UNH7	Sorting nexin-6	SNX6	-0.015	-0.092	0.083
P40925	Malate dehydrogenase, cytoplasmic	MDH1	0.134	0.052	0.083
P22234	Multifunctional protein ADE2	PAICS	-0.068	-0.079	0.083
O95197-3	Isoform 3 of Reticulon-3	RTN3	0.205	-0.046	0.083
O43396	Thioredoxin-like protein 1	TXNL1	-0.092	-0.065	0.082
P08621	U1 small nuclear ribonucleoprotein 70 kDa	SNRNP70	-0.027	-0.158	0.082
P42167	Lamina-associated polypeptide 2, isoforms beta/gamma	TMPO	-0.486	-0.323	0.082
Q7Z2Z2	Elongation factor Tu GTP-binding domain-containing protein 1	EFTUD1		-0.035	0.082
P55060-3	Isoform 3 of Exportin-2	CSE1L	0.001	-0.043	0.082
Q13283	Ras GTPase-activating protein-binding protein 1	G3BP1	-0.096	-0.123	0.081
P26038	Moesin	MSN	-0.062	0.057	0.081
O95926	Pre-mRNA-splicing factor SYF2	SYF2		-0.121	0.081
Q08J23	tRNA (cytosine(34)-C(5))-methyltransferase	NSUN2	-0.254	-0.171	0.081
P53634	Dipeptidyl peptidase 1	CTSC	-0.816		0.081
Q15843	NEDD8	NEDD8	-0.582	-0.030	0.081
P42025	Beta-centractin	ACTR1B		-0.124	0.081
P42166	Lamina-associated polypeptide 2, isoform alpha	TMPO	-0.203	-0.110	0.081
P09493-3	Isoform 3 of Tropomyosin alpha-1 chain	TPM1	0.381	0.268	0.080
Q96AG4	Leucine-rich repeat-containing protein 59	LRRC59	-0.062	-0.017	0.080
P82912-2	Isoform 2 of 28S ribosomal protein S11, mitochondrial	MRPS11	0.090	0.195	0.080
Q6ZRR7-2	Isoform 2 of Leucine-rich repeat-containing protein 9	LRRC9			0.080
P12236	ADP/ATP translocase 3	SLC25A6	-0.016	0.034	0.080
E9PIR7	Thioredoxin reductase 1, cytoplasmic	TXNRD1	-0.516	-0.250	0.079
H0YJN0	Ena/VASP-like protein (Fragment)	EVL		0.027	0.079
Q5TFE4	5'-nucleotidase domain-containing protein 1	NT5DC1	0.017	0.058	0.079
P20839-2	Isoform 2 of Inosine-5'-monophosphate dehydrogenase 1	IMPDH1	-0.138	0.156	0.079

P49959-2	Isoform 2 of Double-strand break repair protein MRE11A	MRE11A	0.229	0.032	0.079
Q9BYZ2	L-lactate dehydrogenase A-like 6B	LDHAL6B	-0.156	-0.015	0.078
E7ENQ1	Mitogen-activated protein kinase kinase kinase kinase 4	MAP4K4	-0.242		0.078
O95299	NADH dehydrogenase [ubiquinone] 1 alpha subcomplex subunit 10, mitochondrial	NDUFA10	-0.102	0.000	0.078
Q9GZN8	UPF0687 protein C20orf27	C20orf27	-0.122	-0.183	0.078
P07108	Acyl-CoA-binding protein	DBI	0.090	-0.267	0.078
D6RA31	Alpha-synuclein (Fragment)	SNCA		0.373	0.078
O94903	Proline synthase co-transcribed bacterial homolog protein	PROSC	0.059	0.101	0.077
O00154-6	Isoform 6 of Cytosolic acyl coenzyme A thioester hydrolase	ACOT7	0.090	-0.113	0.077
Q14694	Ubiquitin carboxyl-terminal hydrolase 10	USP10	-0.039	-0.036	0.077
P01130-5	Isoform 5 of Low-density lipoprotein receptor	LDLR	0.097		0.077
C9JEV6	N-acetyl-D-glucosamine kinase	NAGK	0.164	0.176	0.077
Q15233	Non-POU domain-containing octamer-binding protein	NONO	-0.069	-0.235	0.077
O15498	Synaptobrevin homolog YKT6	YKT6	-0.243	-0.055	0.077
O00231	26S proteasome non-ATPase regulatory subunit 11	PSMD11	-0.131	-0.087	0.077
P49327	Fatty acid synthase	FASN	-0.037	-0.051	0.077
Q9BYC5	Alpha-(1,6)-fucosyltransferase	FUT8	0.168		0.077
Q9Y3D9	28S ribosomal protein S23, mitochondrial	MRPS23	-0.087	-0.006	0.077
Q13347	Eukaryotic translation initiation factor 3 subunit I	EIF3I	-0.142	-0.146	0.077
Q9Y2I1-4	Isoform 4 of Nischarin	NISCH	-0.089		0.076
P07919	Cytochrome b-c1 complex subunit 6, mitochondrial	UQCRH	-0.067	-0.106	0.076
Q96M27	Protein PRRC1	PRRC1	0.025	0.204	0.076
Q9H6T0-2	Isoform 2 of Epithelial splicing regulatory protein 2	ESRP2	-0.177		0.076
Q12788	Transducin beta-like protein 3	TBL3	-0.076	-0.107	0.076
P26885	Peptidyl-prolyl cis-trans isomerase FKBP2	FKBP2	-0.074	-0.042	0.076
P61163	Alpha-centractin	ACTR1A	0.078	-0.031	0.075
O94973	AP-2 complex subunit alpha-2	AP2A2	-0.084	0.014	0.075
Q13200	26S proteasome non-ATPase regulatory subunit 2	PSMD2	-0.108	-0.130	0.075
Q92520	Protein FAM3C	FAM3C	0.391	0.367	0.075
P53602	Diphosphomevalonate decarboxylase	MVD	0.019	0.096	0.075
C9J6G3	Geranylgeranyl pyrophosphate synthase (Fragment)	GGPS1	0.074	0.094	0.075
Q15723-4	Isoform 4 of ETS-related transcription factor Elf-2	ELF2	-0.037	-0.208	0.075
P52789	Hexokinase-2	HK2	-0.399	-0.273	0.074
P09960	Leukotriene A-4 hydrolase	LTA4H	0.276	0.139	0.074
Q9NRX5	Serine incorporator 1	SERINC1		0.208	0.074
P35606	Coatomer subunit beta'	COPB2	-0.094	-0.011	0.074
C9J000	Enoyl-CoA delta isomerase 2, mitochondrial	ECI2	0.104	0.183	0.074
P29034	Protein S100-A2	S100A2		0.625	0.074
P10768	S-formylglutathione hydrolase	ESD	-0.088	0.040	0.074
K7EQ02	DAZ-associated protein 1 (Fragment)	DAZAP1	-0.030	-0.164	0.073
Q9Y295	Developmentally-regulated GTP-binding protein 1	DRG1	-0.116	-0.347	0.073
O95834	Echinoderm microtubule-associated protein-like 2	EML2	0.118	0.412	0.073
P18124	60S ribosomal protein L7	RPL7	-0.133	-0.058	0.073
H3BPF6	Prefoldin subunit 5 (Fragment)	PFDN5	0.125	-0.102	0.073
O75874	Isocitrate dehydrogenase [NADP] cytoplasmic	IDH1	-0.108	0.039	0.073
P49411	Elongation factor Tu, mitochondrial	TUFM	-0.037	0.018	0.072
P00492	Hypoxanthine-guanine phosphoribosyltransferase	HPRT1	0.005	-0.020	0.072
Q08209-3	Isoform 3 of Serine/threonine-protein phosphatase 2B catalytic subunit alpha isofo	PPP3CA	0.076	0.038	0.072
O95754	Semaphorin-4F	SEMA4F			0.071
P06744	Glucose-6-phosphate isomerase	GPI	-0.032	-0.015	0.071
Q12906-7	Isoform 7 of Interleukin enhancer-binding factor 3	ILF3	-0.013	-0.266	0.071
P61981	14-3-3 protein gamma	YWHAG	-0.054	-0.175	0.071
O95373	Importin-7	IPO7	-0.052	-0.052	0.071
Q9UL25	Ras-related protein Rab-21	RAB21	0.061	0.077	0.071
Q9NZI7-4	Isoform 2 of Upstream-binding protein 1	UBP1	0.095	-0.097	0.071
Q9HDC9	Adipocyte plasma membrane-associated protein	APMAP	0.021	0.022	0.071
C9JFE4	COP9 signalosome complex subunit 1	GPS1	-0.093	-0.002	0.071
C9JLU1	DNA-directed RNA polymerases I, II, and III subunit RPABC3 (Fragment)	POLR2H	0.008	-0.207	0.071
Q14257	Reticulocalbin-2	RCN2	-0.081	0.000	0.071
Q99961	Endophilin-A2	SH3GL1	-0.276	-0.016	0.071
G3V5W5	Protein arginine N-methyltransferase 5	PRMT5	-0.070	0.083	0.071
H0YEH2	Pumilio homolog 1 (Fragment)	PUM1	0.038	-0.266	0.070
Q5VT79	Annexin A8-like protein 2	ANXA8L2	0.177		0.070
P53804-3	Isoform TPRDIII of E3 ubiquitin-protein ligase TTC3	TTC3			0.070
P09874	Poly [ADP-ribose] polymerase 1	PARP1	-0.150	0.107	0.070
Q99460-2	Isoform 2 of 26S proteasome non-ATPase regulatory subunit 1	PSMD1	-0.092	-0.076	0.070
Q86X55-1	Isoform 1 of Histone-arginine methyltransferase CARM1	CARM1	0.151	0.096	0.070
B7Z844	Solute carrier family 2, facilitated glucose transporter member 14	SLC2A14			0.070
Q96TA1-2	Isoform 2 of Niban-like protein 1	FAM129B	-0.136	-0.030	0.070
Q9NP97	Dynein light chain roadblock-type 1	DYNLRB1	-0.061	-0.082	0.070
Q14980-2	Isoform 2 of Nuclear mitotic apparatus protein 1	NUMA1	0.149	0.017	0.069
P62837	Ubiquitin-conjugating enzyme E2 D2	UBE2D2	-0.034	-0.023	0.069
O14656-2	Isoform 2 of Torsin-1A	TOR1A			0.069
O00159-2	Isoform 2 of Unconventional myosin-Ic	MYO1C	-0.006	-0.223	0.069
P11021	78 kDa glucose-regulated protein	HSPA5	0.126	0.119	0.069
O95171-3	Isoform 3 of Sciellin	SCEL			0.069
P50851-2	Isoform 2 of Lipopolysaccharide-responsive and beige-like anchor protein	LRBA	0.005	0.030	0.069
P04844-2	Isoform 2 of Dolichyl-diphosphooligosaccharide--protein glycosyltransferase subur	RPN2	0.082	0.120	0.069
P23229-7	Isoform 7 of Integrin alpha-6	ITGA6	-0.034		0.068
P04083	Annexin A1	ANXA1	0.198	0.213	0.068
Q9Y262	Eukaryotic translation initiation factor 3 subunit L	EIF3L	-0.098	-0.047	0.068
E5RIW3	Tubulin-specific chaperone A	TBCA	-0.088	-0.047	0.068
Q86Y56	HEAT repeat-containing protein 2	HEATR2	-0.224	-0.158	0.068
P48147	Prolyl endopeptidase	PREP	-0.068	-0.024	0.068
O75396	Vesicle-trafficking protein SEC22b	SEC22B	-0.010	0.104	0.067
Q15046	Lysine--tRNA ligase	KARS	-0.317	-0.201	0.067
P31946-2	Isoform Short of 14-3-3 protein beta/alpha	YWHAB	-0.059	-0.003	0.067

Q969H8	UPF0556 protein C19orf10	C19orf10	-0.082	0.004	0.067
P61457	Pterin-4-alpha-carbinolamine dehydratase	PCBD1	-0.080		0.067
Q9BQL6	Fermitin family homolog 1	FERMT1	-0.180		0.067
Q5VW36	Focadhesin	FOCAD		0.063	0.067
Q9BWD1	Acetyl-CoA acetyltransferase, cytosolic	ACAT2	0.182	-0.082	0.066
Q9UBS4	DnaJ homolog subfamily B member 11	DNAJB11	0.145	0.049	0.066
Q5QPE7	Mitochondrial genome maintenance exonuclease 1	MGME1	0.161		0.066
O14964	Hepatocyte growth factor-regulated tyrosine kinase substrate	HGS	0.014	0.086	0.066
O00148	ATP-dependent RNA helicase DDX39A	DDX39A	0.003	-0.101	0.066
Q9H993	UPF0364 protein C6orf211	C6orf211	0.058	0.035	0.066
P50395	Rab GDP dissociation inhibitor beta	GDI2	-0.004	-0.039	0.066
C9JA28	Translocon-associated protein subunit gamma	SSR3			0.066
P63104	14-3-3 protein zeta/delta	YWHAZ	-0.062	-0.007	0.066
Q9BRX8-2	Isoform 2 of Redox-regulatory protein FAM213A	FAM213A	0.246		0.066
Q9BTD8-4	Isoform 4 of RNA-binding protein 42	RBM42	-0.054	-0.069	0.066
E7ER77	Endoplasmic reticulum metallopeptidase 1	ERMP1	0.053		0.066
P53004	Biliverdin reductase A	BLVRA	-0.012	0.081	0.065
Q07666	KH domain-containing, RNA-binding, signal transduction-associated protein 1	KHDRBS1	-0.064	-0.329	0.065
Q9Y3F4	Serine-threonine kinase receptor-associated protein	STRAP	-0.091	-0.091	0.065
Q7KZF4	Staphylococcal nuclease domain-containing protein 1	SND1	-0.007	0.039	0.065
Q12965	Unconventional myosin-Ie	MYO1E	0.362		0.065
P23919	Thymidylate kinase	DTYMK	-0.015	-0.131	0.065
Q12792	Twinfilin-1	TWF1	-0.113	0.020	0.065
Q16555-2	Isoform 2 of Dihydropyrimidinase-related protein 2	DPYSL2	0.343	-0.195	0.065
Q8N183	Mimitin, mitochondrial	NDUFAF2	0.043	-0.094	0.065
B3KVN2	Farnesyltransferase, CAAX box, alpha, isoform CRA_c	FNTA	-0.102	0.002	0.065
Q7Z3C6-2	Isoform 2 of Autophagy-related protein 9A	ATG9A		-0.138	0.064
H7C417	Uncharacterized protein (Fragment)	4			0.064
P49419-2	Isoform 2 of Alpha-aminoadipic semialdehyde dehydrogenase	ALDH7A1	0.223	0.177	0.064
H7C3C7	Synaptosomal-associated protein 47 (Fragment)	SNAP47			0.064
P61160	Actin-related protein 2	ACTR2	0.005	0.004	0.064
C9JJP5	Protein TFG (Fragment)	TFG	0.105	0.041	0.064
Q96RE7	Nucleus accumbens-associated protein 1	NACC1		0.050	0.064
F8VVL1	Density-regulated protein	DENR	-0.046	-0.075	0.063
H7BXH2	Serine/threonine-protein phosphatase 6 regulatory subunit 3	PPP6R3	0.060	-0.069	0.063
C9J9K3	40S ribosomal protein SA (Fragment)	RPSA	-0.112	-0.065	0.063
Q8N9N5-3	Isoform 3 of Protein BANP	BANP	0.047	-0.378	0.063
E9PGT1	Translin	TSN	-0.028	-0.076	0.063
O00422	Histone deacetylase complex subunit SAP18	SAP18	-0.077	-0.544	0.063
Q9NVT9	Armadillo repeat-containing protein 1	ARMC1	0.024	0.143	0.063
P40429	60S ribosomal protein L13a	RPL13A	-0.117	-0.014	0.063
O00303	Eukaryotic translation initiation factor 3 subunit F	EIF3F	-0.017	0.001	0.063
P62191	26S protease regulatory subunit 4	PSMC1	-0.105	-0.105	0.062
Q8WXF1-2	Isoform 2 of Paraspeckle component 1	PSPC1	0.058		0.062
P48444	Coatomer subunit delta	ARCN1	-0.046	-0.012	0.062
Q53S33	BolA-like protein 3	BOLA3			0.062
P31947	14-3-3 protein sigma	SFN	-0.010		0.062
Q9NSD9	Phenylalanine--tRNA ligase beta subunit	FARSB	0.038	-0.002	0.062
Q9UJX5-2	Isoform 2 of Anaphase-promoting complex subunit 4	ANAPC4	0.045	0.297	0.061
A8MW61	Pleiotropic regulator 1	PLRG1	-0.003	-0.159	0.061
B8ZZQ6	Thymosin alpha-1	PTMA	-0.249	-0.031	0.061
H0YJ64	Pleckstrin-2 (Fragment)	PLEK2	-0.180		0.060
Q99436	Proteasome subunit beta type-7	PSMB7	-0.205	-0.092	0.059
Q8TDX7	Serine/threonine-protein kinase Nek7	NEK7	0.156	0.069	0.059
Q86XP3	ATP-dependent RNA helicase DDX42	DDX42	0.105	0.132	0.059
P30566	Adenylosuccinate lyase	ADSL	-0.072	0.046	0.059
P21964-2	Isoform Soluble of Catechol O-methyltransferase	COMT	-0.251	0.126	0.059
O75822	Eukaryotic translation initiation factor 3 subunit J	EIF3J	-0.151	-0.055	0.059
O75534-2	Isoform Short of Cold shock domain-containing protein E1	CSDE1	-0.032	-0.139	0.059
P60174-1	Isoform 2 of Triosephosphate isomerase	TPI1	-0.080	-0.048	0.059
P53618	Coatomer subunit beta	COPB1	-0.094	0.021	0.059
Q9BV40	Vesicle-associated membrane protein 8	VAMP8	0.060	0.035	0.059
O43707	Alpha-actinin-4	ACTN4	-0.352	0.424	0.058
Q96N67-4	Isoform 4 of Dedicator of cytokinesis protein 7	DOCK7	-0.203		0.058
P08574	Cytochrome c1, heme protein, mitochondrial	CYC1	-0.115	0.140	0.058
Q9Y3I0	tRNA-splicing ligase RtcB homolog	C22orf28	-0.050	0.016	0.058
O43852	Calumenin	CALU	0.070	-0.039	0.058
P61289	Proteasome activator complex subunit 3	PSME3	-0.088	-0.066	0.058
Q9Y2W1	Thyroid hormone receptor-associated protein 3	THRAP3	-0.012	0.019	0.058
O60216	Double-strand-break repair protein rad21 homolog	RAD21	-0.118	-0.001	0.057
Q9BTT0	Acidic leucine-rich nuclear phosphoprotein 32 family member E	ANP32E	0.029	0.075	0.057
Q9Y547	Heat shock protein beta-11	HSPB11	0.020	0.141	0.057
B5MCF9	Pescadillo homolog	PES1	-0.069	-0.291	0.056
H3BS70	Enoyl-CoA delta isomerase 1, mitochondrial (Fragment)	ECI1	0.055	0.119	0.056
O43815-2	Isoform 2 of Striatin	STRN	0.015	-0.421	0.056
Q9H5Q4	Dimethyladenosine transferase 2, mitochondrial	TFB2M	-0.069	-0.047	0.056
Q9Y5J7	Mitochondrial import inner membrane translocase subunit Tim9	TIMM9	-0.233	-0.472	0.056
O60841	Eukaryotic translation initiation factor 5B	EIF5B	-0.052	-0.070	0.056
Q04917	14-3-3 protein eta	YWHAH	0.017	0.011	0.056
Q8WZ82	Ovarian cancer-associated gene 2 protein	OVCA2	-0.106	-0.251	0.056
E5RH53	Regulator of microtubule dynamics protein 1	RMDN1	0.069	0.039	0.056
P17174	Aspartate aminotransferase, cytoplasmic	GOT1	0.162	0.107	0.056
P13473	Lysosome-associated membrane glycoprotein 2	LAMP2	-0.013	0.360	0.056
Q9NPD8	Ubiquitin-conjugating enzyme E2 T	UBE2T	0.045	-0.121	0.056
P11216	Glycogen phosphorylase, brain form	PYGB	0.047	-0.078	0.056
Q13418	Integrin-linked protein kinase	ILK	0.039	-0.059	0.056
H0YMB3	GMP reductase 2	GMPR2	0.097	0.136	0.056

Q13045-3	Isoform 3 of Protein flightless-1 homolog	FLII	0.077	0.079	0.055
E5RGS5	RWD domain-containing protein 1 (Fragment)	RWDD1	-0.311	-0.059	0.055
O60271-4	Isoform 4 of C-Jun-amino-terminal kinase-interacting protein 4	SPAG9	0.209	0.085	0.055
P62861	40S ribosomal protein S30	FAU	0.164	-0.051	0.055
H0YD57	CD82 antigen (Fragment)	CD82			0.055
Q6NZI2	Polymerase I and transcript release factor	PTRF	0.055		0.055
Q9UHG3	Prenylcysteine oxidase 1	PCYOX1	0.010	0.110	0.055
E9PN17	ATP synthase subunit g, mitochondrial	ATP5L	-0.156	0.227	0.055
B1AK87	Capping protein (Actin filament) muscle Z-line, beta, isoform CRA_a	CAPZB	0.048	0.089	0.054
P33316-2	Isoform 2 of Deoxyuridine 5'-triphosphate nucleotidohydrolase, mitochondrial	DUT	-0.260	-0.084	0.054
B4DVE7	Annexin	ANXA11	-0.022	-0.185	0.054
P26639	Threonine--tRNA ligase, cytoplasmic	TARS	-0.028	0.016	0.054
P46777	60S ribosomal protein L5	RPL5	-0.125	-0.044	0.054
O15212	Prefoldin subunit 6	PFDN6	-0.066	-0.077	0.054
P15880	40S ribosomal protein S2	RPS2	-0.116	-0.038	0.054
P55769	NHP2-like protein 1	NHP2L1	0.057	-0.216	0.054
Q17RY6	Lymphocyte antigen 6K	LY6K			0.054
C9J3L8	Translocon-associated protein subunit alpha	SSR1	-0.158	-0.031	0.054
P30837	Aldehyde dehydrogenase X, mitochondrial	ALDH1B1	-0.018		0.053
F5H7J5	Regulatory-associated protein of mTOR	RPTOR	0.182	0.263	0.053
Q9Y5P6	Mannose-1-phosphate guanyltransferase beta	GMPPB	-0.079	-0.286	0.053
H3BNW5	Kunitz-type protease inhibitor 1 (Fragment)	SPINT1	0.051	0.590	0.053
Q9H7D7-2	Isoform 2 of WD repeat-containing protein 26	WDR26	0.053	-0.081	0.053
Q96PU8-8	Isoform 5 of Protein quaking	QKI	-0.208	0.115	0.053
H3BTA2	Serine/threonine-protein phosphatase (Fragment)	PPP4C	-0.011	-0.027	0.053
Q96I24	Far upstream element-binding protein 3	FUBP3	-0.139	-0.156	0.053
O14828	Secretory carrier-associated membrane protein 3	SCAMP3		0.020	0.053
Q14204	Cytoplasmic dynein 1 heavy chain 1	DYNC1H1	0.019	0.009	0.053
Q8N3C0	Activating signal cointegrator 1 complex subunit 3	ASCC3	-0.073	0.064	0.053
P13798	Acylamino-acid-releasing enzyme	APEH	-0.055	0.023	0.053
Q96G03	Phosphoglucomutase-2	PGM2	0.173	0.067	0.053
Q86UE4	Protein LYRIC	MTDH	0.034	-0.001	0.052
Q14C86-4	Isoform 4 of GTPase-activating protein and VPS9 domain-containing protein 1	GAPVD1	-0.056	0.037	0.052
Q9NR30	Nucleolar RNA helicase 2	DDX21	-0.093	-0.252	0.052
O00764	Pyridoxal kinase	PDXK	-0.167	-0.022	0.052
Q92888	Rho guanine nucleotide exchange factor 1	ARHGEF1	0.043	-0.012	0.052
O60664	Perilipin-3	PLIN3	0.119	0.003	0.052
E9PLK3	Puromycin-sensitive aminopeptidase	NPEPPS	0.278	0.188	0.052
Q96S52-2	Isoform 2 of GPI transamidase component PIG-S	PIGS	0.156		0.052
Q96IX5	Up-regulated during skeletal muscle growth protein 5	USMG5	-0.091	0.110	0.052
F5GWA6	Myopalladin	MYPN			0.051
E5RHG8	Transcription elongation factor B polypeptide 1 (Fragment)	TCEB1	0.030	-0.079	0.051
P46087-2	Isoform 2 of Putative ribosomal RNA methyltransferase NOP2	NOP2	0.076	0.017	0.051
Q10567-3	Isoform C of AP-1 complex subunit beta-1	AP1B1	0.021	0.007	0.051
Q14289-2	Isoform 2 of Protein-tyrosine kinase 2-beta	PTK2B			0.051
P26599	Polypyrimidine tract-binding protein 1	PTBP1	-0.017	-0.160	0.051
O94763-2	Isoform 2 of Unconventional prefoldin RPB5 interactor 1	URI1	0.043	0.091	0.051
O15260-2	Isoform 2 of Surfeit locus protein 4	SURF4	-0.046	0.106	0.051
H0Y6Y5	Metaxin-1 (Fragment)	MTX1	-0.156	0.094	0.050
Q5SZR1	39S ribosomal protein L9, mitochondrial	MRPL9			0.049
G3V126	ATPase, H+ transporting, lysosomal 50/57kDa, V1 subunit H, isoform CRA_c	ATP6V1H	-0.041	0.009	0.049
H0YJ92	CDK-activating kinase assembly factor MAT1 (Fragment)	MNAT1	0.106	0.149	0.049
F2Z2V0	Copine-1 (Fragment)	CPNE1	0.021	0.061	0.049
Q15149-3	Isoform 3 of Plectin	PLEC	-0.144		0.049
P62888	60S ribosomal protein L30	RPL30	-0.178	-0.094	0.049
Q9Y2X3	Nucleolar protein 58	NOP58	0.092	-0.048	0.049
H0YED9	Wilms tumor protein (Fragment)	WT1			0.049
Q8N8S7-2	Isoform 2 of Protein enabled homolog	ENAH	-0.225	-0.023	0.049
P05386	60S acidic ribosomal protein P1	RPLP1	-0.103	-0.119	0.049
Q15397	Pumilio domain-containing protein KIAA0020	KIAA0020	0.090	0.028	0.049
Q8TF05-2	Isoform 2 of Serine/threonine-protein phosphatase 4 regulatory subunit 1	PPP4R1	0.001		0.049
Q15056-2	Isoform Short of Eukaryotic translation initiation factor 4H	EIF4H	-0.100	-0.167	0.048
P12004	Proliferating cell nuclear antigen	PCNA	-0.095	-0.094	0.048
Q9H0F6	Sharpin	SHARPIN	-0.054		0.048
Q99798	Aconitate hydratase, mitochondrial	ACO2	-0.018	0.104	0.048
O43765	Small glutamine-rich tetratricopeptide repeat-containing protein alpha	SGTA	-0.134	-0.080	0.048
D6RGI3	Septin 11, isoform CRA_b	SEPT11	0.100	-0.142	0.048
H3BV80	RNA-binding protein with serine-rich domain 1	RNPS1	-0.008	-0.120	0.048
P49589	Cysteine--tRNA ligase, cytoplasmic	CARS	0.097	0.134	0.048
Q96LJ7	Dehydrogenase/reductase SDR family member 1	DHRS1	-0.100		0.048
Q5SZE1	Ceramide synthase 2 (Fragment)	CERS2	-0.001	0.029	0.048
Q9NZ32	Actin-related protein 10	ACTR10	0.042		0.048
Q504Z1	EIF4G3 protein	EIF4G3	-0.174	-0.279	0.048
P17096-2	Isoform HMG-Y of High mobility group protein HMG-I/HMG-Y	HMGA1	-0.134		0.047
J3QQT2	60S ribosomal protein L17 (Fragment)	RPL17	-0.003	-0.126	0.047
Q8IWT6	Leucine-rich repeat-containing protein 8A	LRRC8A		-0.305	0.047
Q16563-2	Isoform 2 of Synaptophysin-like protein 1	SYPL1	0.108	0.155	0.047
Q10713	Mitochondrial-processing peptidase subunit alpha	PMPCA	-0.041	-0.077	0.047
Q15459	Splicing factor 3A subunit 1	SF3A1	0.039	-0.025	0.047
Q9Y224	UPF0568 protein C14orf166	C14orf166	-0.023	-0.094	0.047
O60506-2	Isoform 2 of Heterogeneous nuclear ribonucleoprotein Q	SYNCRIP	-0.079	-0.207	0.047
Q15738	Sterol-4-alpha-carboxylate 3-dehydrogenase, decarboxylating	NSDHL	0.179	0.035	0.047
P30044-2	Isoform Cytoplasmic+peroxisomal of Peroxiredoxin-5, mitochondrial	PRDX5	-0.049	0.071	0.047
Q9Y2S7	Polymerase delta-interacting protein 2	POLDIP2	-0.090	0.007	0.046
P83881	60S ribosomal protein L36a	RPL36A	-0.032	-0.030	0.046
P55036	26S proteasome non-ATPase regulatory subunit 4	PSMD4	-0.079	0.008	0.046
P62273	40S ribosomal protein S29	RPS29	-0.147	-0.237	0.046

E9PNU1	Nucleoside diphosphate kinase 7	NME7	-0.170		0.046
P30740	Leukocyte elastase inhibitor	SERPINB1	-0.073		0.046
F5GXX5	Dolichyl-diphosphooligosaccharide--protein glycosyltransferase subunit DAD1	DAD1	0.128	0.090	0.046
P51610-2	Isoform 2 of Host cell factor 1	HCFC1	-0.073	0.047	0.046
P10619	Lysosomal protective protein	CTSA	0.071	-0.130	0.046
I3L300	Adenylate cyclase type 9 (Fragment)	ADCY9			0.046
P56937-3	Isoform 3 of 3-keto-steroid reductase	HSD17B7	0.249		0.046
Q9UBQ7	Glyoxylate reductase/hydroxypyruvate reductase	GRHPR	0.011	0.113	0.045
F5H0L8	SEC23-interacting protein	SEC23IP	-0.021	0.001	0.045
P49593	Protein phosphatase 1F	PPM1F	0.092	-0.102	0.045
Q9UPN6	Protein SCAF8	SCAF8		0.069	0.045
Q92499	ATP-dependent RNA helicase DDX1	DDX1	-0.009	0.023	0.044
Q9H3U1-2	Isoform 2 of Protein unc-45 homolog A	UNC45A	-0.079	-0.131	0.044
P31949	Protein S100-A11	S100A11	0.139	0.423	0.044
P67775	Serine/threonine-protein phosphatase 2A catalytic subunit alpha isoform	PPP2CA	0.023	-0.076	0.044
P15170-2	Isoform 2 of Eukaryotic peptide chain release factor GTP-binding subunit ERF3A	GSPT1	-0.071	-0.060	0.044
Q96A65	Exocyst complex component 4	EXOC4	0.076	0.093	0.044
P47914	60S ribosomal protein L29	RPL29	0.040	-0.211	0.044
O60443	Non-syndromic hearing impairment protein 5	DFNA5			0.044
Q14914-2	Isoform 2 of Prostaglandin reductase 1	PTGR1	-0.101	-0.067	0.043
P26358	DNA (cytosine-5)-methyltransferase 1	DNMT1	-0.146	-0.110	0.043
P51148	Ras-related protein Rab-5C	RAB5C	0.072	0.144	0.043
Q7L1Q6	Basic leucine zipper and W2 domain-containing protein 1	BZW1	-0.110	0.099	0.043
Q9NTJ5	Phosphatidylinositide phosphatase SAC1	SACM1L	-0.195	-0.117	0.042
P78346	Ribonuclease P protein subunit p30	RPP30	-0.066	-0.040	0.042
O43252	Bifunctional 3'-phosphoadenosine 5'-phosphosulfate synthase 1	PAPSS1	0.118	0.099	0.042
P54578-2	Isoform 2 of Ubiquitin carboxyl-terminal hydrolase 14	USP14	-0.072	-0.052	0.042
O43242	26S proteasome non-ATPase regulatory subunit 3	PSMD3	-0.138	-0.111	0.041
P59998	Actin-related protein 2/3 complex subunit 4	ARPC4	0.051	0.012	0.041
P61247	40S ribosomal protein S3a	RPS3A	-0.077	-0.099	0.041
Q9HC38-2	Isoform 2 of Glyoxalase domain-containing protein 4	GLOD4	0.004	0.011	0.041
Q9UGP8	Translocation protein SEC63 homolog	SEC63	0.072	0.113	0.041
O76021	Ribosomal L1 domain-containing protein 1	RSL1D1	0.005	-0.270	0.041
P20042	Eukaryotic translation initiation factor 2 subunit 2	EIF2S2	-0.009	-0.087	0.040
Q9NQ66	1-phosphatidylinositol 4,5-bisphosphate phosphodiesterase beta-1	PLCB1	-0.226	0.012	0.040
Q6P4A7-3	Isoform 3 of Sideroflexin-4	SFXN4		0.101	0.040
H3BU16	Hematological and neurological-expressed 1-like protein (Fragment)	HN1L	0.031	-0.082	0.040
Q9BV57	1,2-dihydroxy-3-keto-5-methylthiopentene dioxygenase	ADI1	-0.115	-0.049	0.040
O15372	Eukaryotic translation initiation factor 3 subunit H	EIF3H	-0.093	0.014	0.040
Q9Y3C6	Peptidyl-prolyl cis-trans isomerase-like 1	PPIL1	-0.019	-0.295	0.040
O76003	Glutaredoxin-3	GLRX3	-0.085	-0.003	0.040
Q9Y3Q3	Transmembrane emp24 domain-containing protein 3	TMED3	-0.088	0.237	0.040
Q96RS6	NudC domain-containing protein 1	NUDCD1	0.028	-0.076	0.039
C9JQQ2	3-oxoacyl-[acyl-carrier-protein] synthase, mitochondrial (Fragment)	OXSM	-0.066	0.027	0.039
P63173	60S ribosomal protein L38	RPL38	-0.103	-0.080	0.039
Q86V81	THO complex subunit 4	ALYREF	-0.038	-0.159	0.039
Q9Y678	Coatomer subunit gamma-1	COPG1	-0.063	0.062	0.039
Q15942	Zyxin	ZYX	0.058	-0.013	0.039
Q15008	26S proteasome non-ATPase regulatory subunit 6	PSMD6	-0.118	-0.127	0.039
P62851	40S ribosomal protein S25	RPS25	-0.141	-0.110	0.039
P36578	60S ribosomal protein L4	RPL4	-0.124	-0.102	0.039
Q8IYV9-2	Isoform 2 of Izumo sperm-egg fusion protein 1	IZUMO1			0.039
P30050	60S ribosomal protein L12	RPL12	-0.062	-0.093	0.038
P62140	Serine/threonine-protein phosphatase PP1-beta catalytic subunit	PPP1CB	0.181	0.034	0.038
E7ES10	DNA-directed RNA polymerase I subunit RPA34 (Fragment)	CAST	0.142	-0.252	0.037
Q9P260	LisH domain and HEAT repeat-containing protein KIAA1468	KIAA1468		-0.498	0.037
K7ELL7	Glucosidase 2 subunit beta	PRKCSH	0.136	0.101	0.037
P36405	ADP-ribosylation factor-like protein 3	ARL3	0.032	-0.198	0.036
Q14738-2	Isoform Delta-2 of Serine/threonine-protein phosphatase 2A 56 kDa regulatory su	PPP2R5D	-0.101	0.161	0.036
P62263	40S ribosomal protein S14	RPS14	-0.043	-0.045	0.036
O00515	Ladinin-1	LAD1	-0.014		0.036
J3KQU0	Nefastin-1	NUCB2		0.152	0.036
Q92665	28S ribosomal protein S31, mitochondrial	MRPS31	-0.017	0.190	0.035
P04843	Dolichyl-diphosphooligosaccharide--protein glycosyltransferase subunit 1	RPN1	0.064	0.077	0.035
Q6L8Q7-2	Isoform 2 of 2',5'-phosphodiesterase 12	PDE12	-0.038	-0.001	0.035
Q6IBS0	Twinfilin-2	TWF2	0.046	0.049	0.035
Q96N66	Lysophospholipid acyltransferase 7	MBOAT7	0.028	-0.061	0.035
Q16890-4	Isoform 4 of Tumor protein D53	TPD52L1	-0.083		0.035
P19338	Nucleolin	NCL	-0.035	-0.044	0.035
P00558	Phosphoglycerate kinase 1	PGK1	-0.008	0.059	0.035
Q9BVK6	Transmembrane emp24 domain-containing protein 9	TMED9	0.012	0.110	0.035
P30049	ATP synthase subunit delta, mitochondrial	ATP5D	-0.091	0.014	0.034
E9PNW4	CD59 glycoprotein	CD59	-0.013	0.047	0.034
P09110	3-ketoacyl-CoA thiolase, peroxisomal	ACAA1	0.289	0.175	0.034
Q99575	Ribonucleases P/MRP protein subunit POP1	POP1	0.018	0.113	0.034
Q96HY7	Probable 2-oxoglutarate dehydrogenase E1 component DHKTD1, mitochondrial	DHTKD1	0.068	0.019	0.034
P67936	Tropomyosin alpha-4 chain	TPM4	0.137	0.147	0.034
P30046	D-dopachrome decarboxylase	DDT	-0.052	-0.168	0.034
P52597	Heterogeneous nuclear ribonucleoprotein F	HNRNPF	0.056	-0.124	0.033
Q15363	Transmembrane emp24 domain-containing protein 2	TMED2	0.015	0.091	0.033
P30153	Serine/threonine-protein phosphatase 2A 65 kDa regulatory subunit A alpha isofor	PPP2R1A	-0.025	0.039	0.033
F5GX33	Calcium-binding mitochondrial carrier protein Aralar2	SLC25A13	0.090	-0.021	0.033
Q86UY0	TXNDC5 protein	TXNDC5	-0.227	-0.067	0.033
P41091	Eukaryotic translation initiation factor 2 subunit 3	EIF2S3	0.009	0.044	0.033
Q99714	3-hydroxyacyl-CoA dehydrogenase type-2	HSD17B10	0.138	0.029	0.033
P07954-2	Isoform Cytoplasmic of Fumarate hydratase, mitochondrial	FH	-0.009	0.110	0.033
Q99643-5	Isoform 5 of Succinate dehydrogenase cytochrome b560 subunit, mitochondrial	SDHC	0.052	0.215	0.033

Q9NZM1-6	Isoform 6 of Myoferlin	MYOF	0.060	0.133	0.033
Q9NPD3	Exosome complex component RRP41	EXOSC4	-0.121	-0.214	0.032
P62241	40S ribosomal protein S8	RPS8	-0.086	-0.201	0.032
Q13435	Splicing factor 3B subunit 2	SF3B2	0.027	-0.046	0.032
Q99470	Stromal cell-derived factor 2	SDF2	0.281	-0.040	0.032
H7BXW7	Mitochondrial pyruvate carrier 1	MPC1			0.032
E7ES08	High mobility group protein B3 (Fragment)	HMGB3	-0.201	-0.106	0.032
P61353	60S ribosomal protein L27	RPL27	-0.134	-0.109	0.032
P27708	CAD protein	CAD	0.003	0.056	0.032
Q92597	Protein NDRG1	NDRG1	-0.167	-0.187	0.032
P56192	Methionine--tRNA ligase, cytoplasmic	MARS	0.229	0.038	0.032
Q00839	Heterogeneous nuclear ribonucleoprotein U	HNRNPU	-0.025	-0.108	0.032
P52788	Spermine synthase	SMS	0.053	0.010	0.031
O60568	Procollagen-lysine,2-oxoglutarate 5-dioxygenase 3	PLOD3	0.090	-0.027	0.031
H0YM11	Small kinetochore-associated protein (Fragment)	KNSTRN	-0.188	-0.020	0.031
Q96RS0	Trimethylguanosine synthase	TGS1	0.132		0.031
O00244	Copper transport protein ATOX1	ATOX1	-0.052	-0.219	0.031
Q9BPX5	Actin-related protein 2/3 complex subunit 5-like protein	ARPC5L	-0.024	-0.014	0.031
Q9H2M9	Rab3 GTPase-activating protein non-catalytic subunit	RAB3GAP2	0.055	-0.010	0.031
P13804	Electron transfer flavoprotein subunit alpha, mitochondrial	ETFA	-0.037	0.176	0.031
Q9Y673-2	Isoform 2 of Dolichyl-phosphate beta-glucosyltransferase	ALG5	0.121	0.166	0.031
Q9NQR4	Omega-amidase NIT2	NIT2	0.062	0.118	0.031
Q9H223	EH domain-containing protein 4	EHD4	-0.032		0.031
Q6NXG1-2	Isoform 2 of Epithelial splicing regulatory protein 1	ESRP1	-0.094		0.031
P29401	Transketolase	TKT	-0.161	0.009	0.031
Q13765-2	Isoform 2 of Nascent polypeptide-associated complex subunit alpha	NACA	-0.167	-0.134	0.031
Q6DKK2	Tetratricopeptide repeat protein 19, mitochondrial	TTC19	0.018	-0.080	0.030
Q13242	Serine/arginine-rich splicing factor 9	SRSF9	-0.062		0.030
H3BUX2	Cytochrome b5 type B	CYB5B	0.128	0.122	0.030
Q09666	Neuroblast differentiation-associated protein AHNAK	AHNAK	-0.107	0.206	0.030
Q08257	Quinone oxidoreductase	CRYZ	0.395	0.065	0.030
E9PBU3	Phosphoribosylaminoimidazolecarboxamide formyltransferase	ATIC	-0.013	0.037	0.030
Q13057	Bifunctional coenzyme A synthase	COASY	-0.097		0.030
P39019	40S ribosomal protein S19	RPS19	-0.085	-0.087	0.030
O76094	Signal recognition particle subunit SRP72	SRP72	-0.135	0.000	0.029
P35998	26S protease regulatory subunit 7	PSMC2	-0.079	-0.127	0.029
Q16836	Hydroxyacyl-coenzyme A dehydrogenase, mitochondrial	HADH	0.172	0.051	0.029
Q02750	Dual specificity mitogen-activated protein kinase kinase 1	MAP2K1	0.066	0.052	0.029
Q92905	COP9 signalosome complex subunit 5	COPS5	-0.071	0.003	0.029
Q06830	Peroxiredoxin-1	PRDX1	-0.110	-0.005	0.029
Q9UI10-3	Isoform 3 of Translation initiation factor eIF-2B subunit delta	EIF2B4	0.050	0.120	0.028
Q9Y2Q3-3	Isoform 3 of Glutathione S-transferase kappa 1	GSTK1	-0.034	0.176	0.028
P30086	Phosphatidylethanolamine-binding protein 1	PEBP1	-0.293	-0.146	0.028
O00429-4	Isoform 3 of Dynamin-1-like protein	DNM1L	0.045	0.046	0.028
E9PD53	Structural maintenance of chromosomes protein	SMC4	-0.086	-0.007	0.028
Q9BUL8	Programmed cell death protein 10	PDCD10	-0.035	0.061	0.028
O95433	Activator of 90 kDa heat shock protein ATPase homolog 1	AHSA1	-0.276	-0.174	0.028
K7EN06	GTP-binding protein Di-Ras1 (Fragment)	DIRAS1	-0.145		0.028
O75351	Vacuolar protein sorting-associated protein 4B	VPS4B	0.123	-0.041	0.027
O75934	Pre-mRNA-splicing factor SPF27	BCAS2	-0.018	-0.522	0.027
K7EPB9	Tropomyosin alpha-4 chain (Fragment)	TPM4			0.027
Q06136	3-ketodihydrosphingosine reductase	KDSR		0.120	0.027
Q9UNF0-2	Isoform 2 of Protein kinase C and casein kinase substrate in neurons protein 2	PACSIN2	-0.051	-0.054	0.027
Q96CS3	FAS-associated factor 2	FAF2	-0.009	-0.103	0.027
Q15181	Inorganic pyrophosphatase	PPA1	-0.076	-0.147	0.027
P67870	Casein kinase II subunit beta	CSNK2B	-0.022	0.000	0.027
Q9NYL2-3	Isoform 3 of Mitogen-activated protein kinase kinase kinase MLT	MLTK	-0.064	0.425	0.027
Q9H173	Nucleotide exchange factor SIL1	SIL1	-0.197		0.026
Q6PIU2	Neutral cholesterol ester hydrolase 1	NCEH1	-0.533		0.026
Q99614	Tetratricopeptide repeat protein 1	TTC1	0.014	-0.108	0.026
Q86UP2-2	Isoform 2 of Kinectin	KTN1	-0.074	0.035	0.026
P16949	Stathmin	STMN1	-0.094	-0.135	0.026
H0YMT0	RAS guanyl-releasing protein 1 (Fragment)	RASGRP1			0.026
E7EX44	Caldesmon	CALD1	0.198	0.711	0.026
P07384	Calpain-1 catalytic subunit	CAPN1	0.091	0.189	0.025
Q15382	GTP-binding protein Rheb	RHEB	0.019	0.045	0.025
P0CB43	Protein FAM203B	FAM203B	-0.095	-0.002	0.025
P17152	Transmembrane protein 11, mitochondrial	TMEM11	-0.002	0.270	0.025
Q9GZM7-3	Isoform 3 of Tubulointerstitial nephritis antigen-like	TINAGL1			0.024
P54727	UV excision repair protein RAD23 homolog B	RAD23B	0.036	0.011	0.024
P63244	Guanine nucleotide-binding protein subunit beta-2-like 1	GNB2L1	-0.046	-0.149	0.024
P48651	Phosphatidylserine synthase 1	PTDSS1	0.010	0.020	0.024
P43243	Matrin-3	MATR3	0.014	-0.047	0.024
P60953	Cell division control protein 42 homolog	CDC42	-0.002	0.057	0.024
Q9UQ80	Proliferation-associated protein 2G4	PA2G4	0.002	-0.060	0.023
P08581	Hepatocyte growth factor receptor	MET	0.070		0.023
I3L2B0	Clustered mitochondria protein homolog	CLUH		-0.152	0.023
Q16629-3	Isoform 3 of Serine/arginine-rich splicing factor 7	SRSF7	-0.032	-0.324	0.023
P63010	AP-2 complex subunit beta	AP2B1	0.005	-0.014	0.023
P12081-4	Isoform 4 of Histidine--tRNA ligase, cytoplasmic	HARS	0.002	-0.020	0.022
Q9Y2D4	Exocyst complex component 6B	EXOC6B	0.074	0.031	0.022
E9PMR4	CD151 antigen	CD151	-0.152	0.122	0.022
P08238	Heat shock protein HSP 90-beta	HSP90AB1	-0.166	-0.166	0.022
P30041	Peroxiredoxin-6	PRDX6	-0.003	0.083	0.022
Q5VW32	BRO1 domain-containing protein BROX	BROX	0.141	0.315	0.022
P60468	Protein transport protein Sec61 subunit beta	SEC61B	-0.074	0.045	0.022
O15067	Phosphoribosylformylglycinamidine synthase	PFAS	-0.224	-0.039	0.022

Q96BJ3-3	Isoform 3 of Axin interactor, dorsalization-associated protein	AIDA	0.011	0.134	0.021
Q9NZB2	Constitutive coactivator of PPAR-gamma-like protein 1	FAM120A	0.110	-0.010	0.021
Q9NW13-2	Isoform 2 of RNA-binding protein 28	RBM28	0.014	0.002	0.021
Q8N1F7	Nuclear pore complex protein Nup93	NUP93	-0.035	-0.068	0.021
P26583	High mobility group protein B2	HMGB2	0.017	-0.067	0.021
P62277	40S ribosomal protein S13	RPS13	-0.088	-0.053	0.021
Q9BT78	COP9 signalosome complex subunit 4	COPS4	-0.088	0.012	0.021
P49792	E3 SUMO-protein ligase RanBP2	RANBP2	0.040	-0.009	0.021
P50502	Hsc70-interacting protein	ST13	-0.207	-0.166	0.021
P60842	Eukaryotic initiation factor 4A-I	EIF4A1	-0.146	-0.238	0.021
P07737	Profilin-1	PFN1	-0.091	-0.090	0.020
P53396-2	Isoform 2 of ATP-citrate synthase	ACLY	0.166	0.020	0.020
P62750	60S ribosomal protein L23a	RPL23A	-0.068	-0.075	0.020
G5E9R5	Acid phosphatase 1, soluble, isoform CRA_d	ACP1	0.117	-0.203	0.020
O75131	Copine-3	CPNE3	-0.075	0.049	0.020
J3KTL7	Cyclin-dependent kinase 11B	CDK11B	0.001	0.054	0.020
P50914	60S ribosomal protein L14	RPL14	-0.076	-0.063	0.020
P07942	Laminin subunit beta-1	LAMB1		-0.109	0.020
P62333	26S protease regulatory subunit 10B	PSMC6	-0.085	-0.107	0.020
Q13308-3	Isoform 3 of Inactive tyrosine-protein kinase 7	PTK7	0.065		0.020
Q9BSD7	Cancer-related nucleoside-triphosphatase	NTPCR	-0.014	-0.112	0.020
Q6ZN66-2	Isoform 2 of Guanylate-binding protein 6	GBP6			0.020
Q9Y2V2	Calcium-regulated heat stable protein 1	CARHSP1	0.085	0.057	0.020
Q69YU5	Uncharacterized protein C12orf73	C12orf73	-0.446	-0.279	0.020
O75340	Programmed cell death protein 6	PDCD6	0.040	0.079	0.019
D6R9H7	ATP synthase lipid-binding protein, mitochondrial	ATP5G1	0.320	0.244	0.019
P11310	Medium-chain specific acyl-CoA dehydrogenase, mitochondrial	ACADM	0.166	-0.055	0.019
P15927	Replication protein A 32 kDa subunit	RPA2	0.151	-0.030	0.019
P27635	60S ribosomal protein L10	RPL10	0.001	-0.063	0.019
P05455	Lupus La protein	SSB	0.022	-0.014	0.019
P50552	Vasodilator-stimulated phosphoprotein	VASP	-0.005	0.058	0.019
P60900	Proteasome subunit alpha type-6	PSMA6	-0.097	-0.068	0.019
Q16540	39S ribosomal protein L23, mitochondrial	MRPL23	0.003	-0.080	0.019
B4DEK4	Sorting nexin-2	SNX2	0.001	-0.045	0.019
P00441	Superoxide dismutase [Cu-Zn]	SOD1	0.001	-0.104	0.019
Q8WWI1-3	Isoform 3 of LIM domain only protein 7	LMO7	0.359	-0.034	0.019
P13797	Plastin-3	PLS3	0.149	0.013	0.019
P46781	40S ribosomal protein S9	RPS9	-0.096	-0.062	0.018
Q9H3K6	BolA-like protein 2	BOLA2	-0.003	-0.175	0.018
Q13126	S-methyl-5'-thioadenosine phosphorylase	MTAP	-0.152	-0.075	0.018
P25398	40S ribosomal protein S12	RPS12	-0.114	-0.219	0.018
P22061	Protein-L-isoaspartate(D-aspartate) O-methyltransferase	PCMT1	0.015	0.012	0.018
F5H5Y3	Signal recognition particle subunit SRP68	SRP68	-0.111	-0.027	0.018
P34932	Heat shock 70 kDa protein 4	HSPA4	-0.009	-0.042	0.018
F5GXW0	Adenosine deaminase	ADA	0.239		0.018
P51149	Ras-related protein Rab-7a	RAB7A	-0.027	0.050	0.017
Q9UHY1	Nuclear receptor-binding protein	NRBP1	-0.156	-0.034	0.017
Q7Z460-2	Isoform 2 of CLIP-associating protein 1	CLASP1	0.174		0.017
P62072	Mitochondrial import inner membrane translocase subunit Tim10	TIMM10	-0.174	-0.172	0.017
O96019	Actin-like protein 6A	ACTL6A	-0.028	-0.052	0.017
F5GZU3	Scaffold attachment factor B1	SAFB	-0.051	-0.373	0.017
A8MU28	NEDD8-activating enzyme E1 regulatory subunit	NAE1	0.112	0.056	0.016
Q8WWM7	Ataxin-2-like protein	ATXN2L	-0.092	-0.080	0.016
Q8TCJ2	Dolichyl-diphosphooligosaccharide--protein glycosyltransferase subunit STT3B	STT3B	0.086	0.013	0.016
K7EKE6	Lon protease homolog, mitochondrial	LONP1	0.097	0.022	0.016
P15151-3	Isoform Gamma of Poliovirus receptor	PVR			0.016
Q9P2E9	Ribosome-binding protein 1	RRBP1	0.028	0.131	0.016
Q9BPW8	Protein NipSnap homolog 1	NIPSNAP1	0.155	0.058	0.016
Q9Y624	Junctional adhesion molecule A	F11R	-0.272	-0.010	0.016
Q9Y2S6	Translation machinery-associated protein 7	TMA7	-0.281	-0.260	0.016
Q04941	Proteolipid protein 2	PLP2	0.043	0.109	0.015
P23588	Eukaryotic translation initiation factor 4B	EIF4B	-0.011	0.006	0.015
Q13033-2	Isoform Alpha of Striatin-3	STRN3	0.217		0.015
Q5QPP3	UDP-glucose 4-epimerase (Fragment)	GALE	-0.097		0.015
O60547-2	Isoform 2 of GDP-mannose 4,6 dehydratase	GMDS		0.114	0.015
Q9NTZ6	RNA-binding protein 12	RBM12	-0.082	0.056	0.015
M0R208	ATP-dependent Clp protease proteolytic subunit	CLPP	0.186	0.124	0.015
P68036-2	Isoform 2 of Ubiquitin-conjugating enzyme E2 L3	UBE2L3	-0.331	-0.213	0.015
P25788-2	Isoform 2 of Proteasome subunit alpha type-3	PSMA3	-0.104	-0.032	0.014
P22528	Cornifin-B	SPRR1B	-0.094		0.014
Q14126	Desmoglein-2	DSG2	-0.042	0.081	0.014
P48739	Phosphatidylinositol transfer protein beta isoform	PITPNB	-0.114	0.009	0.014
P60228	Eukaryotic translation initiation factor 3 subunit E	EIF3E	-0.102	-0.093	0.014
P54886-2	Isoform Short of Delta-1-pyrroline-5-carboxylate synthase	ALDH18A1	0.005	-0.098	0.014
Q9ULC4-2	Isoform 2 of Malignant T-cell-amplified sequence 1	MCTS1	-0.059	-0.092	0.014
P31150	Rab GDP dissociation inhibitor alpha	GDI1	-0.029	-0.100	0.013
Q13206	Probable ATP-dependent RNA helicase DDX10	DDX10	-0.010	1.149	0.013
P26373	60S ribosomal protein L13	RPL13	-0.060	-0.139	0.013
P08237	6-phosphofructokinase, muscle type	PFKM	0.083	0.123	0.013
J3QKT5	RNA-binding protein Musashi homolog 2	MSI2		0.132	0.013
O43615	Mitochondrial import inner membrane translocase subunit TIM44	TIMM44	0.021	0.102	0.013
Q9NXH9	tRNA (guanine(26)-N(2))-dimethyltransferase	TRMT1	0.064	-0.002	0.013
Q8WUF5	RelA-associated inhibitor	PPP1R13L	0.019	0.033	0.012
F5GYN4	Ubiquitin thioesterase OTUB1	OTUB1	-0.057	0.024	0.012
P13010	X-ray repair cross-complementing protein 5	XRCC5	-0.042	-0.093	0.012
P22102	Trifunctional purine biosynthetic protein adenosine-3	GART	0.000	0.013	0.012
Q6UW63	KDEL motif-containing protein 1	KDELC1		0.057	0.012

Q8NCN5	Pyruvate dehydrogenase phosphatase regulatory subunit, mitochondrial	PDPR	0.319	0.186	0.012
Q96QK1	Vacuolar protein sorting-associated protein 35	VPS35	-0.088	0.028	0.012
O95248	Myotubularin-related protein 5	SBF1			0.011
P62424	60S ribosomal protein L7a	RPL7A	-0.062	-0.081	0.011
E9PBY7	Zinc finger CCCH domain-containing protein 11A (Fragment)	ZC3H11A	-0.038	1.329	0.011
Q659C4	La-related protein 1B	LARP1B	-0.213		0.011
P45877	Peptidyl-prolyl cis-trans isomerase C	PPIC			0.011
P17301	Integrin alpha-2	ITGA2	-0.073		0.011
P61088	Ubiquitin-conjugating enzyme E2 N	UBE2N	-0.019	-0.142	0.011
O76054	SEC14-like protein 2	SEC14L2			0.011
P61006	Ras-related protein Rab-8A	RAB8A	-0.248	-0.114	0.011
J3QR09	Ribosomal protein L19	RPL19	-0.016	-0.103	0.011
O95747	Serine/threonine-protein kinaseR1	OXSR1	0.451	0.262	0.011
Q9NUJ3	T-complex protein 11-like protein 1	TCP11L1			0.011
Q9H1E5	Thioredoxin-related transmembrane protein 4	TMX4		0.085	0.011
B4E0J1	Max dimerization protein 3	MXD3	-0.164	-0.062	0.011
P42704	Leucine-rich PPR motif-containing protein, mitochondrial	LRPPRC	-0.050	0.013	0.011
B3KRS5	Histone deacetylase	HDAC2	-0.041	-0.255	0.011
Q6MZX6	Putative uncharacterized protein DKFZp686J06205	DKFZp686J06205	0.015	-0.120	0.011
P22531	Small proline-rich protein 2E	SPRR2E			0.010
P04792	Heat shock protein beta-1	HSPB1	0.026	-0.064	0.010
B4DT77	Annexin	ANXA7	0.048	0.085	0.010
Q9UJX3-2	Isoform 2 of Anaphase-promoting complex subunit 7	ANAPC7	-0.055	0.098	0.010
P11279	Lysosome-associated membrane glycoprotein 1	LAMP1	-0.014	0.069	0.010
Q96A33	Coiled-coil domain-containing protein 47	CCDC47	0.063	0.047	0.010
P49321	Nuclear autoantigenic sperm protein	NASP	-0.034	-0.063	0.010
Q9HCC0-2	Isoform 2 of Methylcrotonoyl-CoA carboxylase beta chain, mitochondrial	MCCC2	-0.043	0.006	0.010
P53985	Monocarboxylate transporter 1	SLC16A1	-0.104	-0.035	0.009
Q8NC56-2	Isoform 2 of LEM domain-containing protein 2	LEMD2	0.087	0.071	0.009
P49721	Proteasome subunit beta type-2	PSMB2	-0.183	0.019	0.009
Q8N5N7	39S ribosomal protein L50, mitochondrial	MRPL50	0.063	-0.006	0.009
Q5TAQ9	DDB1- and CUL4-associated factor 8	DCAF8	0.305	0.027	0.009
P46783	40S ribosomal protein S10	RPS10	-0.121	-0.062	0.009
K7EM09	Transmembrane protein 205 (Fragment)	TMEM205	-0.038	-0.108	0.009
P08708	40S ribosomal protein S17	RPS17	-0.038	0.019	0.009
Q14152	Eukaryotic translation initiation factor 3 subunit A	EIF3A	0.010	0.038	0.009
P09661	U2 small nuclear ribonucleoprotein A'	SNRPA1	0.061	-0.162	0.008
O75683	Surfeit locus protein 6	SURF6	-0.141	0.013	0.008
Q9BUJ2-2	Isoform 2 of Heterogeneous nuclear ribonucleoprotein U-like protein 1	HNRNPUL1	0.045	-0.051	0.008
P55039	Developmentally-regulated GTP-binding protein 2	DRG2	-0.163	-0.001	0.008
Q96DI7	U5 small nuclear ribonucleoprotein 40 kDa protein	SNRNP40	0.068	-0.209	0.008
Q02878	60S ribosomal protein L6	RPL6	-0.149	-0.124	0.008
Q5T4S7-3	Isoform 3 of E3 ubiquitin-protein ligase UBR4	UBR4	0.069	0.114	0.007
Q9P2J5	Leucine--tRNA ligase, cytoplasmic	LARS	0.056	0.034	0.007
P61970	Nuclear transport factor 2	NUTF2	-0.085	-0.128	0.007
E9PCY5	DNA topoisomerase 2 (Fragment)	TOP2B		-1.559	0.007
Q9BR61	Acyl-CoA-binding domain-containing protein 6	ACBD6	0.032	-0.031	0.007
Q9UQB8-3	Isoform 3 of Brain-specific angiogenesis inhibitor 1-associated protein 2	BAIAP2	-0.032		0.007
P35268	60S ribosomal protein L22	RPL22	-0.104	-0.059	0.007
Q14498-2	Isoform 2 of RNA-binding protein 39	RBM39	-0.056	-0.056	0.007
B4E241	Serine/arginine-rich-splicing factor 3	SFRS3	-0.088	-0.292	0.007
Q01081	Splicing factor U2AF 35 kDa subunit	U2AF1	-0.014	-0.206	0.007
P25787	Proteasome subunit alpha type-2	PSMA2	-0.036	-0.059	0.006
P00505	Aspartate aminotransferase, mitochondrial	GOT2	-0.239	-0.046	0.006
O43324	Eukaryotic translation elongation factor 1 epsilon-1	EEF1E1	0.106	-0.023	0.006
Q6NUM9-2	Isoform 2 of All-trans-retinol 13,14-reductase	RETSAT			0.006
Q8NCA5-2	Isoform 2 of Protein FAM98A	FAM98A	0.036	0.035	0.006
Q8WUY1	Protein THEM6	THEM6	0.090	0.081	0.006
O14579	Coatomer subunit epsilon	COPE	-0.044	-0.055	0.006
O14980	Exportin-1	XPO1	-0.071	-0.010	0.006
H3BU49	ADP-ribosylation factor-like protein 2-binding protein	ARL2BP	-0.350	-0.347	0.006
Q9BVJ7	Dual specificity protein phosphatase 23	DUSP23	-0.138		0.006
E7EPK1	Septin-7	SEPT7	-0.007	-0.013	0.006
P46779	60S ribosomal protein L28	RPL28	-0.128	-0.072	0.006
Q9C0A0-2	Isoform 2 of Contactin-associated protein-like 4	CNTNAP4	-0.221	0.047	0.005
P62306	Small nuclear ribonucleoprotein F	SNRPF	-0.028	-0.252	0.005
P06737-2	Isoform 2 of Glycogen phosphorylase, liver form	PYGL	0.181		0.005
Q9HAV7	GrpE protein homolog 1, mitochondrial	GRPEL1	-0.016	-0.060	0.005
P62070-2	Isoform 2 of Ras-related protein R-Ras2	RRAS2	-0.049		0.005
Q9Y3E5	Peptidyl-tRNA hydrolase 2, mitochondrial	PTRH2	0.094	0.075	0.005
B4DP11	Prostaglandin E synthase 3	PTGES3	-0.090	-0.123	0.005
P50479	PDZ and LIM domain protein 4	PDLIM4	-0.079		0.005
Q00325-2	Isoform B of Phosphate carrier protein, mitochondrial	SLC25A3	-0.102	0.015	0.005
P60866	40S ribosomal protein S20	RPS20	-0.055	-0.027	0.004
Q92979	Ribosomal RNA small subunit methyltransferase NEP1	EMG1	-0.048	-0.063	0.004
P62314	Small nuclear ribonucleoprotein Sm D1	SNRPD1	0.023	0.002	0.004
P40227	T-complex protein 1 subunit zeta	CCT6A	-0.071	-0.049	0.004
P36543	V-type proton ATPase subunit E 1	ATP6V1E1	-0.030	0.243	0.004
P55957	BH3-interacting domain death agonist	BID	-0.148	0.125	0.004
Q12972	Nuclear inhibitor of protein phosphatase 1	PPP1R8	-0.124	0.050	0.003
Q06124-2	Isoform 2 of Tyrosine-protein phosphatase non-receptor type 11	PTPN11	-0.190	0.007	0.003
Q5SY16	Polynucleotide 5'-hydroxyl-kinase NOL9	NOL9	0.092	0.042	0.003
P32119	Peroxiredoxin-2	PRDX2	-0.111	-0.090	0.003
Q9Y3A5	Ribosome maturation protein SBDS	SBDS	-0.011	-0.055	0.003
O43809	Cleavage and polyadenylation specificity factor subunit 5	NUDT21	-0.157	-0.013	0.003
Q99832	T-complex protein 1 subunit eta	CCT7	-0.044	-0.119	0.003
E7EQB8	Isocitrate dehydrogenase [NAD] subunit gamma, mitochondrial	IDH3G		0.204	0.003

Q9H7B2	Ribosome production factor 2 homolog	RPF2	-0.097	-0.044	0.003
B7Z2L0	B-cell receptor-associated protein 29	BCAP29		0.045	0.003
H0Y6I0	Golgin subfamily A member 4 (Fragment)	GOLGA4	0.111	0.098	0.003
P14868	Aspartate--tRNA ligase, cytoplasmic	DARS	-0.064	-0.058	0.002
E5RIH8	Cyclin-C	CCNC			0.002
O75348	V-type proton ATPase subunit G 1	ATP6V1G1	-0.049	0.212	0.002
Q15293	Reticulocalbin-1	RCN1	0.004	-0.010	0.002
Q99733	Nucleosome assembly protein 1-like 4	NAP1L4	0.032	-0.051	0.002
P20936-2	Isoform 2 of Ras GTPase-activating protein 1	RASA1	0.150		0.002
P27816-6	Isoform 6 of Microtubule-associated protein 4	MAP4	0.101	0.028	0.002
Q6P1M0	Long-chain fatty acid transport protein 4	SLC27A4	0.008	-0.062	0.002
Q13188	Serine/threonine-protein kinase 3	STK3	-0.087	-0.079	0.002
G5E9W7	28S ribosomal protein S22, mitochondrial	MRPS22	-0.094	-0.036	0.002
M0R0F0	40S ribosomal protein S5 (Fragment)	RPS5	-0.122	0.003	0.002
P62249	40S ribosomal protein S16	RPS16	-0.065	-0.083	0.001
P28070	Proteasome subunit beta type-4	PSMB4	-0.085	-0.076	0.001
Q15813	Tubulin-specific chaperone E	TBCE	0.211	0.114	0.001
Q96NB3	Zinc finger protein 830	ZNF830		0.051	0.001
F5H1L4	Thioredoxin reductase 2, mitochondrial	TXNRD2	0.110	0.004	0.001
Q8N1Q1	Carbonic anhydrase 13	CA13			0.001
P18077	60S ribosomal protein L35a	RPL35A	-0.199	-0.111	0.001
Q9P287	BRCA2 and CDKN1A-interacting protein	BCCIP	-0.040	0.036	0.001
P05198	Eukaryotic translation initiation factor 2 subunit 1	EIF2S1	-0.105	-0.062	0.001
F5H0U5	Glycolipid transfer protein	GLTP	-0.156	0.081	0.000
Q562R1	Beta-actin-like protein 2	ACTBL2	-0.129	0.239	0.000
G3V203	60S ribosomal protein L18	RPL18	-0.239	-0.103	0.000
Q6UWE0-3	Isoform 3 of E3 ubiquitin-protein ligase LRSAM1	LRSAM1			0.000
Q9Y639-3	Isoform 3 of Neuroplastin	NPTN	-0.094		0.000
Q5JTV8	Torsin-1A-interacting protein 1	TOR1AIP1	0.014	0.101	0.000
Q9NVP1	ATP-dependent RNA helicase DDX18	DDX18	0.078	0.100	0.000
Q05DH4	Protein FAM160A1	FAM160A1			0.000
P16615	Sarcoplasmic/endoplasmic reticulum calcium ATPase 2	ATP2A2	-0.042	0.017	0.000
B4DI28	Polypyrimidine tract-binding protein 2	PTBP2	-0.178	-0.194	0.000
J3KP15	Serine/arginine-rich-splicing factor 2 (Fragment)	SRSF2	-0.078	0.067	0.000
P50990	T-complex protein 1 subunit theta	CCT8	-0.039	-0.100	0.000
B4DJ85	Glomulin	GLMN			0.000
Q9Y5L0-5	Isoform 4 of Transportin-3	TNPO3	0.065	0.117	0.000
Q9H3P7	Golgi resident protein GCP60	ACBD3			0.000
Q15126	Phosphomevalonate kinase	PMVK	-0.216	-0.089	-0.001
P00846	ATP synthase subunit a	MT-ATP6	-0.144	-0.064	-0.001
O43813	LanC-like protein 1	LANCL1	0.155	0.156	-0.001
Q9UNM6	26S proteasome non-ATPase regulatory subunit 13	PSMD13	-0.127	-0.065	-0.001
P09211	Glutathione S-transferase P	GSTP1		-0.001	-0.001
Q16543	Hsp90 co-chaperone Cdc37	CDC37	-0.184	-0.077	-0.001
C9JZI1	Replication factor C subunit 4	RFC4	0.031	-0.050	-0.001
E5RGS4	Prefoldin subunit 1	PFDN1	-0.128	-0.007	-0.002
B7Z5T0	Protein angel homolog 2	ANGEL2			-0.002
P07814	Bifunctional glutamate/proline--tRNA ligase	EPRS	0.132	0.020	-0.002
P62854	40S ribosomal protein S26	RPS26	0.271	-0.174	-0.002
P13489	Ribonuclease inhibitor	RNH1	0.001	0.006	-0.002
Q53EZ4	Centrosomal protein of 55 kDa	CEP55	-0.140	-0.068	-0.002
P23368	NAD-dependent malic enzyme, mitochondrial	ME2		-0.019	-0.002
Q12979-4	Isoform 4 of Active breakpoint cluster region-related protein	ABR		0.027	-0.002
P46776	60S ribosomal protein L27a	RPL27A	0.114	-0.143	-0.002
Q9Y277	Voltage-dependent anion-selective channel protein 3	VDAC3	-0.023	0.040	-0.003
P13929-3	Isoform 3 of Beta-enolase	ENO3	-0.091	-0.014	-0.003
P05388	60S acidic ribosomal protein P0	RPLP0	-0.086	-0.040	-0.003
P42765	3-ketoacyl-CoA thiolase, mitochondrial	ACAA2	0.080	0.156	-0.003
D6REN3	Proteasome assembly chaperone 4	PSMG4	-0.071	-0.044	-0.003
Q9UMX5	Neudesin	NENF	0.039	-0.105	-0.004
P00533	Epidermal growth factor receptor	EGFR	-0.585	-0.197	-0.004
P13667	Protein disulfide-isomerase A4	PDIA4	0.004	-0.043	-0.004
P26196	Probable ATP-dependent RNA helicase DDX6	DDX6	-0.099	-0.104	-0.004
O60502	Bifunctional protein NCOAT	MGEA5	0.222	0.253	-0.004
O14908	PDZ domain-containing protein GIPC1	GIPC1	0.014	0.047	-0.005
Q93008-1	Isoform 2 of Probable ubiquitin carboxyl-terminal hydrolase FAF-X	USP9X	-0.132	0.106	-0.005
P48729	Casein kinase I isoform alpha	CSNK1A1	-0.023	0.053	-0.005
Q96B97-3	Isoform 3 of SH3 domain-containing kinase-binding protein 1	SH3KBP1	0.195	0.164	-0.005
Q9H0D6	5'-3' exoribonuclease 2	XRN2	0.037	-0.076	-0.005
Q93052	Lipoma-preferred partner	LPP	0.190	-0.065	-0.005
P48163	NADP-dependent malic enzyme	ME1	-0.264		-0.005
B2RTQ9	FANCM protein	FANCM		-0.195	-0.005
P16070-8	Isoform 8 of CD44 antigen	CD44	-0.099	-0.282	-0.006
P05141	ADP/ATP translocase 2	SLC25A5	-0.087	0.053	-0.006
P82932	28S ribosomal protein S6, mitochondrial	MRPS6	-0.074	-0.066	-0.006
P82930	28S ribosomal protein S34, mitochondrial	MRPS34	-0.147	-0.004	-0.006
E7EQ69	N-alpha-acetyltransferase 50	NAA50	-0.071	-0.004	-0.006
A6H8Z3	RAB3GAP1 protein	RAB3GAP1	-0.037	0.055	-0.006
Q9HB71	Calcyclin-binding protein	CACYBP	-0.068	-0.185	-0.006
Q9ULT8	E3 ubiquitin-protein ligase HECTD1	HECTD1	-0.107		-0.006
Q14697	Neutral alpha-glucosidase AB	GANAB	0.029	0.092	-0.006
Q99848	Probable rRNA-processing protein EBP2	EBNA1BP2	-0.071	-0.193	-0.006
R4GNH3	26S protease regulatory subunit 6A	PSMC3	-0.077	-0.021	-0.006
P06733	Alpha-enolase	ENO1	-0.093	0.010	-0.006
Q3ZCQ8-2	Isoform 2 of Mitochondrial import inner membrane translocase subunit TIM50	TIMM50	0.200	0.111	-0.006
P21281	V-type proton ATPase subunit B, brain isoform	ATP6V1B2	0.071	0.261	-0.006
O43491	Band 4.1-like protein 2	EPB41L2	-0.063	-0.278	-0.006

P35221	Catenin alpha-1	CTNNA1	-0.040	-0.318	-0.007
Q96MW1	Coiled-coil domain-containing protein 43	CCDC43	-0.088		-0.007
Q9Y2L1	Exosome complex exonuclease RRP44	DIS3	-0.043	-0.041	-0.007
P15559-2	Isoform 2 of NAD(P)H dehydrogenase [quinone] 1	NQO1	-0.343	0.136	-0.007
P31040	Succinate dehydrogenase [ubiquinone] flavoprotein subunit, mitochondrial	SDHA	-0.008	0.144	-0.007
P98161-2	Isoform 2 of Polycystin-1	PKD1	0.015	-0.178	-0.007
P22314	Ubiquitin-like modifier-activating enzyme 1	UBA1	0.007	0.080	-0.007
P32969	60S ribosomal protein L9	RPL9	-0.133	-0.081	-0.007
P30622-2	Isoform 3 of CAP-Gly domain-containing linker protein 1	CLIP1	-0.018	0.163	-0.007
P61081	NEDD8-conjugating enzyme Ubc12	UBE2M	-0.050	-0.032	-0.007
Q9Y4W2-2	Isoform 2 of Ribosomal biogenesis protein LAS1L	LAS1L	-0.175	0.076	-0.007
O75533	Splicing factor 3B subunit 1	SF3B1	0.036	0.007	-0.007
P22695	Cytochrome b-c1 complex subunit 2, mitochondrial	UQCRC2	0.031	0.042	-0.007
P14625	Endoplasmin	HSP90B1	0.073	0.130	-0.007
P63151	Serine/threonine-protein phosphatase 2A 55 kDa regulatory subunit B alpha isofor	PPP2R2A	0.057	-0.138	-0.008
Q9NX58	Cell growth-regulating nucleolar protein	LYAR		-0.233	-0.008
P15924	Desmoplakin	DSP	-0.153	0.085	-0.008
Q06323	Proteasome activator complex subunit 1	PSME1	0.057	0.073	-0.008
O75937	DnaJ homolog subfamily C member 8	DNAJC8	-0.040	0.022	-0.008
Q01469	Fatty acid-binding protein, epidermal	FABP5	0.129	-0.097	-0.008
J3KN67	Tropomyosin alpha-3 chain	TPM3	0.184	-0.061	-0.008
F5GXC8	Succinyl-CoA ligase [ADP-forming] subunit beta, mitochondrial	SUCLA2	-0.027	-0.029	-0.008
P52907	F-actin-capping protein subunit alpha-1	CAPZA1	0.047	0.137	-0.008
Q8NBM8	Prenylcysteine oxidase-like	PCYOX1L	0.572	0.259	-0.008
Q9NTI5-2	Isoform 2 of Sister chromatid cohesion protein PDS5 homolog B	PDS5B	0.124	-0.114	-0.008
P62280	40S ribosomal protein S11	RPS11	-0.090	-0.076	-0.008
P82979	SAP domain-containing ribonucleoprotein	SARNP	0.125	-0.076	-0.009
P35637-2	Isoform Short of RNA-binding protein FUS	FUS	-0.136	-0.167	-0.009
A5YKK6-2	Isoform 2 of CCR4-NOT transcription complex subunit 1	CNOT1	-0.068	0.070	-0.009
Q13547	Histone deacetylase 1	HDAC1	0.147	-0.008	-0.009
Q27J81-2	Isoform 2 of Inverted formin-2	INF2	0.006	-0.252	-0.009
P49915	GMP synthase [glutamine-hydrolyzing]	GMPS	-0.037	-0.066	-0.009
Q9Y4L1	Hypoxia up-regulated protein 1	HYOU1	0.072	0.105	-0.009
Q9BYD3	39S ribosomal protein L4, mitochondrial	MRPL4	-0.047	0.295	-0.009
H7BXI1	Extended synaptotagmin-2 (Fragment)	ESYT2	0.034	0.192	-0.010
Q92820	Gamma-glutamyl hydrolase	GGH	0.299	0.204	-0.010
B4DGU4	Catenin beta-1	CTNNB1	-0.108	0.014	-0.010
P23396	40S ribosomal protein S3	RPS3	-0.119	-0.056	-0.010
E5RJ68	AP-3 complex subunit beta-1	AP3B1	0.130	0.170	-0.010
P17612	cAMP-dependent protein kinase catalytic subunit alpha	PRKACA	-0.116	0.073	-0.010
B4DUS9	3'(2'),5'-bisphosphate nucleotidase 1	BPNT1	0.097	0.102	-0.010
Q15155	Nodal modulator 1	NOMO1	-0.007	0.072	-0.010
P14735	Insulin-degrading enzyme	IDE	0.009	-0.023	-0.010
Q8N0X7	Spartin	SPG20		0.376	-0.010
P27824	Calnexin	CANX	0.022	0.144	-0.010
P17858	6-phosphofructokinase, liver type	PFKL	-0.027	0.025	-0.011
Q9UJX2	Cell division cycle protein 23 homolog	CDC23	0.051	-0.129	-0.011
P49368	T-complex protein 1 subunit gamma	CCT3	-0.039	-0.070	-0.011
P62857	40S ribosomal protein S28	RPS28	-0.200	-0.635	-0.011
P55263-2	Isoform 2 of Adenosine kinase	ADK	0.085	0.076	-0.011
Q9BW92	Threonine--tRNA ligase, mitochondrial	TARS2	0.186	0.015	-0.011
F5H8E5	Ubiquitin carboxyl-terminal hydrolase	USP7	-0.016	-0.053	-0.011
P49756	RNA-binding protein 25	RBM25	-0.118	-0.079	-0.011
P36957	Dihydrolipoyllysine-residue succinyltransferase component of 2-oxoglutarate dehy	DLST	0.083	0.009	-0.011
Q14008	Cytoskeleton-associated protein 5	CKAP5	0.072	0.004	-0.012
P42858	Huntingtin	HTT	0.027	0.040	-0.012
Q9BU61-2	Isoform b of NADH dehydrogenase [ubiquinone] 1 alpha subcomplex assembly fa	NDUFAF3			-0.012
Q8IXI1	Mitochondrial Rho GTPase 2	RHOT2	0.048	0.361	-0.012
P23497	Nuclear autoantigen Sp-100	SP100			-0.012
P00491	Purine nucleoside phosphorylase	PNP	-0.132	-0.100	-0.012
P62258	14-3-3 protein epsilon	YWHAE	-0.068	-0.049	-0.012
P10599-2	Isoform 2 of Thioredoxin	TXN	-0.095	-0.096	-0.013
P12268	Inosine-5'-monophosphate dehydrogenase 2	IMPDH2	-0.068	-0.128	-0.013
P22681	E3 ubiquitin-protein ligase CBL	CBL			-0.013
Q9UNN8	Endothelial protein C receptor	PROCR	0.036		-0.013
P06576	ATP synthase subunit beta, mitochondrial	ATP5B	-0.003	0.174	-0.013
P30084	Enoyl-CoA hydratase, mitochondrial	ECHS1	0.065	0.060	-0.013
Q12905	Interleukin enhancer-binding factor 2	ILF2	-0.046	-0.289	-0.013
A6NHR9	Structural maintenance of chromosomes flexible hinge domain-containing protein	SMCHD1	0.133	-0.090	-0.013
Q9BVG4	Protein PBDC1	PBDC1	0.035	0.117	-0.013
Q92598	Heat shock protein 105 kDa	HSPH1	-0.130	-0.166	-0.013
B4DZG7	ADP-ribosylation factor-like protein 1	ARL1	0.098	0.228	-0.013
P12956	X-ray repair cross-complementing protein 6	XRCC6	0.004	-0.060	-0.014
O95757	Heat shock 70 kDa protein 4L	HSPA4L	0.018	-0.098	-0.014
Q13724-2	Isoform 2 of Mannosyl-oligosaccharide glucosidase	MOGS	0.051	0.091	-0.014
Q9UMX0	Ubiquilin-1	UBQLN1	-0.021	-0.023	-0.014
Q9H2H9	Sodium-coupled neutral amino acid transporter 1	SLC38A1		-0.099	-0.014
P23246	Splicing factor, proline- and glutamine-rich	SFPQ	-0.073	-0.289	-0.014
Q8TEX9	Importin-4	IPO4	-0.315	-0.068	-0.014
O60884	DnaJ homolog subfamily A member 2	DNAJA2	-0.083	-0.020	-0.014
P24539	ATP synthase subunit b, mitochondrial	ATP5F1	0.012	0.198	-0.014
P63167	Dynein light chain 1, cytoplasmic	DYNLL1		-0.048	-0.014
B0QYW5	Peroxisomal membrane protein PMP34	SLC25A17			-0.014
K7ESE3	UV excision repair protein RAD23 homolog A	RAD23A	0.009	0.007	-0.014
P55010	Eukaryotic translation initiation factor 5	EIF5	-0.261	-0.084	-0.014
A8MYC1	Ribonuclease P protein subunit p29	POP4	-0.113		-0.014
P0DJ93	Small integral membrane protein 13	SMIM13	0.087	0.217	-0.015

B1AKV4	Ubiquinol-cytochrome c reductase complex chaperone CBP3 homolog	UQCC		0.077	-0.015
Q9UBR2	Cathepsin Z	CTSZ	-0.288		-0.015
A2A2V2	RNA-binding protein 34 (Fragment)	RBM34	0.032	-0.065	-0.015
O75323	Protein NipSnap homolog 2	GBAS	-0.085	0.006	-0.015
Q8WUM4	Programmed cell death 6-interacting protein	PDCD6IP	0.012	0.059	-0.015
Q9BXK5	Bcl-2-like protein 13	BCL2L13	0.097		-0.015
P54136	Arginine--tRNA ligase, cytoplasmic	RARS	0.008	-0.054	-0.016
P48643	T-complex protein 1 subunit epsilon	CCT5	-0.105	-0.124	-0.016
Q96F86	Enhancer of mRNA-decapping protein 3	EDC3	0.035	0.122	-0.016
Q9Y4K4	Mitogen-activated protein kinase kinase kinase kinase 5	MAP4K5	-0.187		-0.016
Q6ZXV5-2	Isoform 2 of Transmembrane and TPR repeat-containing protein 3	TMTC3			-0.016
P52306	Rap1 GTPase-GDP dissociation stimulator 1	RAP1GDS1	0.057	-0.043	-0.016
Q9H061-2	Isoform 2 of Transmembrane protein 126A	TMEM126A	-0.273		-0.016
P41214	Eukaryotic translation initiation factor 2D	EIF2D	-0.358	0.256	-0.017
P80723	Brain acid soluble protein 1	BASP1	-0.413	0.212	-0.017
Q8TCS8	Polyribonucleotide nucleotidyltransferase 1, mitochondrial	PNPT1	0.041	-0.064	-0.017
Q9NYL9	Tropomodulin-3	TMOD3	-0.006	0.447	-0.017
H0YDU8	Serine/threonine-protein phosphatase (Fragment)	PPP5C	0.017	-0.033	-0.017
P20962	Parathymosin	PTMS	0.049	0.102	-0.017
Q9NSE4	Isoleucine--tRNA ligase, mitochondrial	IARS2	0.036	0.017	-0.017
E7EQ01	Caspase-8 subunit p10 (Fragment)	CASP8	0.044	-0.171	-0.017
K7ERQ8	Uncharacterized protein (Fragment)	3			-0.017
P62269	40S ribosomal protein S18	RPS18	-0.074	-0.125	-0.017
P55072	Transitional endoplasmic reticulum ATPase	VCP	-0.102	-0.053	-0.017
Q86VP6	Cullin-associated NEDD8-dissociated protein 1	CAND1	0.046	0.022	-0.017
Q9NQW6-2	Isoform 2 of Actin-binding protein anillin	ANLN	0.131	0.063	-0.017
P16930	Fumarylacetoacetase	FAH	0.083	-0.060	-0.018
Q9NXR7-4	Isoform 4 of BRCA1-A complex subunit BRE	BRE	0.030	0.105	-0.018
B3KW71	Dihydropteridine reductase	QDPR	-0.032	1.017	-0.018
Q9Y263	Phospholipase A-2-activating protein	PLAA	0.025	-0.024	-0.018
P62753	40S ribosomal protein S6	RPS6	-0.079	-0.059	-0.018
Q9BUB1	PRKAR2A protein	PRKAR2A	0.116	0.101	-0.018
O43681	ATPase ASNA1	ASNA1	0.075	0.238	-0.018
Q9UBU9	Nuclear RNA export factor 1	NXF1	0.098	-0.018	-0.018
Q86V48-2	Isoform 2 of Leucine zipper protein 1	LUZP1	0.114	0.480	-0.018
H3BRE8	RNA polymerase II-associated protein 1	RPAP1	-0.094		-0.019
F5H4J2	Peroxisomal membrane protein PEX14	PEX14	-0.016	-0.007	-0.019
Q9NS69	Mitochondrial import receptor subunit TOM22 homolog	TOMM22	0.005	0.105	-0.019
P30040	Endoplasmic reticulum resident protein 29	ERP29	0.050	0.077	-0.019
P28066	Proteasome subunit alpha type-5	PSMA5	-0.086	-0.023	-0.019
P48449-2	Isoform 2 of Lanosterol synthase	LSS	0.236	0.348	-0.019
Q9H3N1	Thioredoxin-related transmembrane protein 1	TMX1	0.074	0.061	-0.019
Q68E01-2	Isoform 2 of Integrator complex subunit 3	INTS3	0.288	0.087	-0.020
P17812	CTP synthase 1	CTPS1	0.068	0.068	-0.020
Q8IXU6	Solute carrier family 35 member F2	SLC35F2			-0.020
P53367-2	Isoform A of Arfaptin-1	ARFIP1	-0.091	0.005	-0.020
Q9NPQ8-4	Isoform 4 of Synembryn-A	RIC8A	-0.032	0.010	-0.020
Q9H4A6	Golgi phosphoprotein 3	GOLPH3	-0.250	-0.028	-0.020
Q5JW30	Double-stranded RNA-binding protein Staufen homolog 1	STAU1	-0.134	-0.221	-0.020
P00395	Cytochrome c oxidase subunit 1	MT-CO1	-0.139		-0.020
P20674	Cytochrome c oxidase subunit 5A, mitochondrial	COX5A	0.000	-0.049	-0.020
Q8WVM8	Sec1 family domain-containing protein 1	SCFD1	0.248	0.220	-0.020
Q9NX20	39S ribosomal protein L16, mitochondrial	MRPL16	-0.024	0.238	-0.020
M0R1K2	Nitric oxide synthase-interacting protein (Fragment)	NOSIP		-0.208	-0.020
J3KPX7	Prohibitin-2	PHB2	-0.113	-0.004	-0.021
P21796	Voltage-dependent anion-selective channel protein 1	VDAC1	0.024	0.063	-0.021
P61956	Small ubiquitin-related modifier 2	SUMO2	-0.162	-0.067	-0.021
Q96I99	Succinyl-CoA ligase [GDP-forming] subunit beta, mitochondrial	SUCLG2	0.131	0.326	-0.021
Q6P2Q9	Pre-mRNA-processing-splicing factor 8	PRPF8	0.008	-0.135	-0.022
E9PH18	DnaJ homolog subfamily B member 6	DNAJB6	-0.112		-0.022
Q9H078-2	Isoform 2 of Caseinolytic peptidase B protein homolog	CLPB	-0.014	0.173	-0.022
P51665	26S proteasome non-ATPase regulatory subunit 7	PSMD7	-0.101	-0.088	-0.022
Q13263	Transcription intermediary factor 1-beta	TRIM28	0.192	0.006	-0.022
O94776	Metastasis-associated protein MTA2	MTA2	0.056	-0.099	-0.023
P85037	Forkhead box protein K1	FOXK1	-0.001	0.148	-0.023
F8W9X7	Coiled-coil domain-containing protein 93	CCDC93	-0.072		-0.023
P61086	Ubiquitin-conjugating enzyme E2 K	UBE2K	0.013	0.021	-0.023
Q92538	Golgi-specific brefeldin A-resistance guanine nucleotide exchange factor 1	GBF1	-0.043	-0.114	-0.023
Q9UFN0	Protein NipSnap homolog 3A	NIPSNAP3A	0.213	0.051	-0.023
P19174	1-phosphatidylinositol 4,5-bisphosphate phosphodiesterase gamma-1	PLCG1	0.045	0.185	-0.023
Q9BY32	Inosine triphosphate pyrophosphatase	ITPA	-0.081	-0.009	-0.024
P50897	Palmitoyl-protein thioesterase 1	PPT1	0.110	0.118	-0.024
Q9Y5K5-2	Isoform 2 of Ubiquitin carboxyl-terminal hydrolase isozyme L5	UCHL5	-0.122	-0.120	-0.024
Q8WYA6	Beta-catenin-like protein 1	CTNNBL1	0.078	0.071	-0.024
Q9UMS4	Pre-mRNA-processing factor 19	PRPF19	-0.055	-0.238	-0.025
I3L2C7	Gem-associated protein 4	GEMIN4	-0.131	0.062	-0.025
Q14692	Ribosome biogenesis protein BMS1 homolog	BMS1	-0.027	-0.151	-0.025
Q9Y6I8-2	Isoform 2 of Peroxisomal membrane protein 4	PXMP4			-0.025
P11586	C-1-tetrahydrofolate synthase, cytoplasmic	MTHFD1	0.001		-0.025
Q9Y696	Chloride intracellular channel protein 4	CLIC4	-0.161	0.072	-0.025
P20618	Proteasome subunit beta type-1	PSMB1	-0.080	0.027	-0.025
Q9Y320-2	Isoform 2 of Thioredoxin-related transmembrane protein 2	TMX2	0.022	0.212	-0.025
B4DM50	MOB-like protein phocein	MOB4	0.021	-0.146	-0.025
O60306	Intron-binding protein aquarius	AQR	0.065	0.043	-0.025
P62701	40S ribosomal protein S4, X isoform	RPS4X	-0.031	-0.058	-0.025
Q7Z333-3	Isoform 3 of Probable helicase senataxin	SETX	-0.482	-0.537	-0.026
Q9BXJ9	N-alpha-acetyltransferase 15, NatA auxiliary subunit	NAA15	0.066	-0.043	-0.026

Q8NFV4-4	Isoform 4 of Alpha/beta hydrolase domain-containing protein 11	ABHD11	0.102	0.082	-0.026
Q9UI30	tRNA methyltransferase 112 homolog	TRMT112	-0.037	-0.007	-0.026
P51648	Fatty aldehyde dehydrogenase	ALDH3A2	-0.023		-0.026
E9PE17	28S ribosomal protein S17, mitochondrial (Fragment)	MRPS17	-0.019	-0.132	-0.026
Q1KMD3	Heterogeneous nuclear ribonucleoprotein U-like protein 2	HNRNPUL2	0.078	-0.099	-0.026
P50213	Isocitrate dehydrogenase [NAD] subunit alpha, mitochondrial	IDH3A	-0.060	0.066	-0.026
P35244	Replication protein A 14 kDa subunit	RPA3	0.098	-0.238	-0.026
A6NJ11	Ubiquitin fusion degradation protein 1 homolog	UFD1L	-0.401	-0.245	-0.027
C9JC63	Mitochondrial inner membrane protein OXA1L (Fragment)	OXA1L	-0.290		-0.027
Q9NZN4	EH domain-containing protein 2	EHD2		0.090	-0.027
P50991	T-complex protein 1 subunit delta	CCT4	-0.064	-0.084	-0.027
G3V5X4	Nesprin-2	SYNE2	0.105	-0.013	-0.027
Q5VW38-2	Isoform 2 of Protein GPR107	GPR107	-0.238	-0.015	-0.027
P62826	GTP-binding nuclear protein Ran	RAN	0.000	-0.053	-0.027
P08559	Pyruvate dehydrogenase E1 component subunit alpha, somatic form, mitochondria	PDHA1	0.083	0.006	-0.028
E9PG40	Gamma-secretase C-terminal fragment 59	APP	-0.092	0.033	-0.028
P35232	Prohibitin	PHB	-0.116	-0.061	-0.028
Q15907	Ras-related protein Rab-11B	RAB11B	0.009	0.115	-0.028
Q07817-2	Isoform Bcl-X(S) of Bcl-2-like protein 1	BCL2L1			-0.028
P28072	Proteasome subunit beta type-6	PSMB6	-0.236	-0.358	-0.028
P61313	60S ribosomal protein L15	RPL15	-0.071	-0.030	-0.028
Q15365	Poly(rC)-binding protein 1	PCBP1	-0.004	0.012	-0.028
Q9Y394-2	Isoform 2 of Dehydrogenase/reductase SDR family member 7	DHRS7	0.270	0.246	-0.029
P39656	Dolichyl-diphosphooligosaccharide--protein glycosyltransferase 48 kDa subunit	DDOST	0.132	0.093	-0.029
Q9UGI8-2	Isoform 2 of Testin	TES	-0.161		-0.029
B4DVY1	Eukaryotic translation initiation factor 3 subunit D	EIF3D	0.011	0.113	-0.029
P07203	Glutathione peroxidase 1	GPX1	0.097	0.173	-0.029
P52272-2	Isoform 2 of Heterogeneous nuclear ribonucleoprotein M	HNRNPM	-0.077	-0.065	-0.029
P20290-2	Isoform 2 of Transcription factor BTF3	BTF3	-0.238	-0.097	-0.029
Q96HC4	PDZ and LIM domain protein 5	PDLIM5	0.290	-0.195	-0.029
Q9UKR5	Probable ergosterol biosynthetic protein 28	C14orf1	0.014		-0.029
C9J2Y9	DNA-directed RNA polymerase	POLR2B	0.081	0.061	-0.029
P55265-5	Isoform 5 of Double-stranded RNA-specific adenosine deaminase	ADAR	0.035	0.110	-0.029
O43143	Putative pre-mRNA-splicing factor ATP-dependent RNA helicase DHX15	DHX15	-0.056	-0.083	-0.029
P41227	N-alpha-acetyltransferase 10	NAA10	0.005	-0.067	-0.029
Q9Y230	RuvB-like 2	RUVBL2	-0.022	-0.085	-0.029
Q15542-2	Isoform Short of Transcription initiation factor TFIID subunit 5	TAF5			-0.030
E7EU96	Casein kinase II subunit alpha	CSNK2A1	-0.089	-0.088	-0.030
P43686	26S protease regulatory subunit 6B	PSMC4	-0.046	-0.023	-0.030
P61224-2	Isoform 2 of Ras-related protein Rap-1b	RAP1B	0.035	0.122	-0.030
P09471-2	Isoform Alpha-2 of Guanine nucleotide-binding protein G(o) subunit alpha	GNAO1		0.031	-0.030
Q07812-5	Isoform Epsilon of Apoptosis regulator BAX	BAX		0.161	-0.030
Q71RC2-5	Isoform 5 of La-related protein 4	LARP4	-0.267	-0.284	-0.030
O75717	WD repeat and HMG-box DNA-binding protein 1	WDHD1	-0.053	0.061	-0.030
P0DJI8	Serum amyloid A-1 protein	SAA1			-0.031
Q96A49	Synapse-associated protein 1	SYAP1	0.086	0.073	-0.031
P58511	Small integral membrane protein 11	SMIM11			-0.031
Q969Q0	60S ribosomal protein L36a-like	RPL36AL	-0.019		-0.031
P05109	Protein S100-A8	S100A8	0.461		-0.031
Q01813	6-phosphofructokinase type C	PFKP	0.046	-0.040	-0.031
P18065	Insulin-like growth factor-binding protein 2	IGFBP2			-0.032
C9JYQ9	60S ribosomal protein L22-like 1	RPL22L1			-0.032
P84090	Enhancer of rudimentary homolog	ERH	-0.046	-0.126	-0.032
P49137	MAP kinase-activated protein kinase 2	MAPKAPK2	0.119	0.042	-0.032
Q86U38	Nucleolar protein 9	NOP9	0.060	0.158	-0.032
Q6Y1H2	Very-long-chain (3R)-3-hydroxyacyl-[acyl-carrier protein] dehydratase 2	PTPLB	-0.034	0.110	-0.032
R4GMR5	26S proteasome non-ATPase regulatory subunit 8	PSMD8	-0.063	0.002	-0.032
P51659	Peroxisomal multifunctional enzyme type 2	HSD17B4	0.094	0.138	-0.032
Q9P258	Protein RCC2	RCC2	-0.082	-0.153	-0.033
P00367	Glutamate dehydrogenase 1, mitochondrial	GLUD1		0.009	-0.033
Q6NUK1	Calcium-binding mitochondrial carrier protein SCaMC-1	SLC25A24	0.066	0.069	-0.033
Q14CX7	N-alpha-acetyltransferase 25, NatB auxiliary subunit	NAA25	-0.025	0.006	-0.033
P28074	Proteasome subunit beta type-5	PSMB5	-0.212	-0.140	-0.033
B4DJV2	Citrate synthase	CS	-0.252	-0.043	-0.033
P46063	ATP-dependent DNA helicase Q1	RECQL	0.087	-0.096	-0.033
Q07065	Cytoskeleton-associated protein 4	CKAP4	-0.060	0.010	-0.033
B4E1A0	Protein CHURC1-FNTB	CHURC1-FNTB	-0.135	-0.147	-0.034
Q86VN1	Vacuolar protein-sorting-associated protein 36	VPS36	0.161	0.254	-0.034
Q9BQ39	ATP-dependent RNA helicase DDX50	DDX50	0.082	-0.041	-0.034
P23258	Tubulin gamma-1 chain	TUBG1	-0.084	-0.049	-0.034
Q9Y5S9	RNA-binding protein 8A	RBM8A	-0.094	-0.159	-0.034
Q9NYY8-2	Isoform 2 of FAST kinase domain-containing protein 2	FASTKD2	-0.195	0.104	-0.034
P42766	60S ribosomal protein L35	RPL35	0.097	-0.111	-0.034
P17987	T-complex protein 1 subunit alpha	TCP1	-0.050	-0.134	-0.035
C9J0K6	Sorcin	SRI	0.239	-0.076	-0.035
O43172-2	Isoform 2 of U4/U6 small nuclear ribonucleoprotein Prp4	PRPF4	0.061	0.011	-0.035
Q9H6Y2	WD repeat-containing protein 55	WDR55	0.013	0.098	-0.035
F8WBK5	39S ribosomal protein L40, mitochondrial	MRPL40	0.049	0.101	-0.035
P62330	ADP-ribosylation factor 6	ARF6	-0.047		-0.035
P17655	Calpain-2 catalytic subunit	CAPN2	-0.029	-0.019	-0.035
P56385	ATP synthase subunit e, mitochondrial	ATP5I	-0.265	-0.381	-0.035
Q6P2I3	Fumarylacetoacetate hydrolase domain-containing protein 2B	FAHD2B	-0.076	0.078	-0.035
Q92973-2	Isoform 2 of Transportin-1	TNPO1	-0.045	0.009	-0.035
P07195	L-lactate dehydrogenase B chain	LDHB	-0.121	0.069	-0.036
Q9Y3T9	Nucleolar complex protein 2 homolog	NOC2L	0.050	0.088	-0.036
D6RBV2	Vesicular integral-membrane protein VIP36	LMAN2	0.074	0.096	-0.036
Q01844-6	Isoform 6 of RNA-binding protein EWS	EWSR1	-0.001	0.023	-0.036

P19388	DNA-directed RNA polymerases I, II, and III subunit RPABC1	POLR2E	0.007	0.051	-0.036
P31350	Ribonucleoside-diphosphate reductase subunit M2	RRM2	0.247	-0.047	-0.036
P11142	Heat shock cognate 71 kDa protein	HSPA8	-0.077	-0.052	-0.036
B4DI16	Fructose-2,6-bisphosphatase	PFKFB2	0.019	0.095	-0.036
P13073	Cytochrome c oxidase subunit 4 isoform 1, mitochondrial	COX4I1	-0.103	0.012	-0.037
O95487-2	Isoform 2 of Protein transport protein Sec24B	SEC24B	-0.218	-0.116	-0.037
Q92922	SWI/SNF complex subunit SMARCC1	SMARCC1	0.101	-0.158	-0.037
J3QRY5	CDK5 and ABL1 enzyme substrate 1	CABLES1			-0.037
F8VYY9	5'-AMP-activated protein kinase subunit gamma-1	PRKAG1	-0.192	0.097	-0.037
P39748	Flap endonuclease 1	FEN1	-0.018	0.028	-0.037
Q13148	TAR DNA-binding protein 43	TARDBP	0.000	-0.067	-0.037
Q14739	Lamin-B receptor	LBR	-0.358	-0.186	-0.037
P24390	ER lumen protein retaining receptor 1	KDELR1	0.135	-0.105	-0.037
Q9BXP5-4	Isoform 4 of Serrate RNA effector molecule homolog	SRRT	-0.060	-0.100	-0.037
Q03405-3	Isoform 3 of Urokinase plasminogen activator surface receptor	PLAUR	0.155		-0.038
P05023	Sodium/potassium-transporting ATPase subunit alpha-1	ATP1A1	-0.123	0.050	-0.038
Q9NUJ1	Mycophenolic acid acyl-glucuronide esterase, mitochondrial	ABHD10	0.081	0.101	-0.038
E7EUI5	Disintegrin and metalloproteinase domain-containing protein 17	ADAM17	-0.098		-0.038
F5H702	39S ribosomal protein L48, mitochondrial	MRPL48		0.025	-0.038
Q9UBE0	SUMO-activating enzyme subunit 1	SAE1	0.071	0.046	-0.038
P62906	60S ribosomal protein L10a	RPL10A	-0.080	-0.070	-0.038
Q6UB35	Monofunctional C1-tetrahydrofolate synthase, mitochondrial	MTHFD1L	-0.134	-0.209	-0.038
Q14974	Importin subunit beta-1	KPNB1	-0.040	0.021	-0.038
Q92945	Far upstream element-binding protein 2	KHSRP	-0.366	-0.207	-0.038
P04632	Calpain small subunit 1	CAPNS1	-0.035	-0.086	-0.038
Q9Y3Y2-4	Isoform 3 of Chromatin target of PRMT1 protein	CHTOP	0.038	-0.047	-0.038
O95881	Thioredoxin domain-containing protein 12	TXNDC12	0.075	-0.060	-0.038
P46778	60S ribosomal protein L21	RPL21	-0.115	-0.110	-0.038
Q5JVF3-3	Isoform 3 of PCI domain-containing protein 2	PCID2	-0.083	0.001	-0.039
P25786	Proteasome subunit alpha type-1	PSMA1	-0.075	0.017	-0.039
P50452	Serpin B8	SERPINB8			-0.039
Q9H501	ESF1 homolog	ESF1	0.000		-0.039
Q8N766-4	Isoform 4 of ER membrane protein complex subunit 1	EMC1	0.089	0.143	-0.039
O00743-2	Isoform 2 of Serine/threonine-protein phosphatase 6 catalytic subunit	PPP6C	-0.022	-0.046	-0.039
G3V198	Nuclear pore complex protein Nup160	NUP160	0.070	-0.044	-0.039
J3KN29	26S proteasome non-ATPase regulatory subunit 9	PSMD9	-0.056	-0.060	-0.039
Q00535	Cyclin-dependent kinase 5	CDK5	-0.148	0.026	-0.039
P38919	Eukaryotic initiation factor 4A-III	EIF4A3	0.017	-0.076	-0.039
Q02338	D-beta-hydroxybutyrate dehydrogenase, mitochondrial	BDH1	-0.109	0.000	-0.039
F5H2J1	Myosin-IIIb (Fragment)	MYO3B	-0.315		-0.039
Q93096	Protein tyrosine phosphatase type IVA 1	PTP4A1		0.039	-0.040
Q9UPT5-5	Isoform 5 of Exocyst complex component 7	EXOC7	-0.029		-0.040
Q9NZ01	Very-long-chain enoyl-CoA reductase	TECR	-0.013	0.065	-0.040
G5EA52	Protein disulfide isomerase family A, member 3, isoform CRA_b	PDIA3	0.039	0.106	-0.040
P14324-2	Isoform 2 of Farnesyl pyrophosphate synthase	FDPS	0.145	0.121	-0.040
Q03154-2	Isoform 2 of Aminoacylase-1	ACY1	0.099		-0.040
Q9H4A4	Aminopeptidase B	RNPEP	0.087	0.064	-0.040
Q9HB40	Retinoid-inducible serine carboxypeptidase	SCPEP1		0.240	-0.041
P05387	60S acidic ribosomal protein P2	RPLP2	-0.118	-0.160	-0.041
P60903	Protein S100-A10	S100A10	-0.065	0.072	-0.041
O15160-2	Isoform 2 of DNA-directed RNA polymerases I and III subunit RPAC1	POLR1C	-0.075	0.059	-0.041
Q99426	Tubulin-folding cofactor B	TBCB	0.343	-0.007	-0.041
Q4KMQ2-3	Isoform 3 of Anoctamin-6	ANO6		0.000	-0.041
O60762	Dolichol-phosphate mannosyltransferase	DPM1	-0.021	-0.013	-0.041
P50402	Emerin	EMD	0.004	-0.126	-0.041
P11172	Uridine 5'-monophosphate synthase	UMPS	0.021	-0.010	-0.041
Q5T4U8	Geranylgeranyl transferase type-2 subunit beta	RABGGTB	0.082	0.004	-0.041
E9PL10	Transcription factor BTF3 homolog 4	BTF3L4	0.061	-0.046	-0.041
C9J8U2	Nicotinate phosphoribosyltransferase	NAPRT1	0.310		-0.041
P48047	ATP synthase subunit O, mitochondrial	ATP5O	0.052	0.200	-0.041
Q9NUU7	ATP-dependent RNA helicase DDX19A	DDX19A	-0.159	-0.017	-0.041
Q13185	Chromobox protein homolog 3	CBX3	-0.111	-0.163	-0.041
P52815	39S ribosomal protein L12, mitochondrial	MRPL12	-0.193	0.099	-0.042
Q5QJE6	Deoxynucleotidyltransferase terminal-interacting protein 2	DNTTIP2	1.536	-0.609	-0.042
H3BTV1	CDP-diacylglycerol--inositol 3-phosphatidyltransferase	CDIPT	0.043	0.162	-0.042
H3BPJ9	NADH dehydrogenase [ubiquinone] 1 beta subcomplex subunit 10	NDUFB10	0.044	-0.044	-0.042
Q8N684-2	Isoform 2 of Cleavage and polyadenylation specificity factor subunit 7	CPSF7	-0.052	-0.019	-0.042
P43490	Nicotinamide phosphoribosyltransferase	NAMPT	-0.007	0.063	-0.042
P40926	Malate dehydrogenase, mitochondrial	MDH2	-0.036	0.063	-0.042
Q16718	NADH dehydrogenase [ubiquinone] 1 alpha subcomplex subunit 5	NDUFA5	-0.001	0.029	-0.042
Q92900-2	Isoform 2 of Regulator of nonsense transcripts 1	UPF1	-0.059	0.082	-0.042
B4DQ51	Short/branched chain-specific acyl-CoA dehydrogenase, mitochondrial	ACADSB	0.004		-0.042
P62266	40S ribosomal protein S23	RPS23	0.089	-0.126	-0.043
P36639-4	Isoform p18 of 7,8-dihydro-8-oxoguanine triphosphatase	NUDT1	0.046	-0.166	-0.043
P78406	mRNA export factor	RAE1	0.041	-0.103	-0.043
E9PC26	Mitotic checkpoint serine/threonine-protein kinase BUB1	BUB1	0.039	-0.713	-0.043
Q5VYK3	Proteasome-associated protein ECM29 homolog	ECM29	-0.050	0.055	-0.043
Q96EN8	Molybdenum cofactor sulfurase	MOCOS			-0.043
P35659	Protein DEK	DEK	0.042	-0.124	-0.044
P62244	40S ribosomal protein S15a	RPS15A	-0.054	-0.081	-0.044
Q9BYN8	28S ribosomal protein S26, mitochondrial	MRPS26	0.070	0.039	-0.044
Q9Y3D3	28S ribosomal protein S16, mitochondrial	MRPS16	-0.100	0.048	-0.044
Q9NQG5	Regulation of nuclear pre-mRNA domain-containing protein 1B	RPRD1B	-0.264	-0.266	-0.044
E7ET15	U2 snRNP-associated SURP motif-containing protein	U2SURP	-0.086	0.041	-0.044
Q06787-8	Isoform 8 of Fragile X mental retardation protein 1	FMR1	0.118	-0.028	-0.044
O43598	2'-deoxynucleoside 5'-phosphate N-hydrolase 1	DNPH1	0.055	-0.032	-0.044
P09758	Tumor-associated calcium signal transducer 2	TACSTD2	0.001	0.287	-0.044

Q15393	Splicing factor 3B subunit 3	SF3B3	0.063	0.122	-0.044
B4DL14	ATP synthase subunit gamma	ATP5C1	-0.043	0.119	-0.044
P62917	60S ribosomal protein L8	RPL8	-0.058	-0.085	-0.045
Q16401-2	Isoform 2 of 26S proteasome non-ATPase regulatory subunit 5	PSMD5	-0.081	-0.093	-0.045
Q6PHR2-2	Isoform 2 of Serine/threonine-protein kinase ULK3	ULK3			-0.045
P62316	Small nuclear ribonucleoprotein Sm D2	SNRPD2	-0.018	-0.093	-0.045
Q9Y3B7-2	Isoform 2 of 39S ribosomal protein L11, mitochondrial	MRPL11			-0.045
P29966	Myristoylated alanine-rich C-kinase substrate	MARCKS	0.216	0.209	-0.045
O75477	Erlin-1	ERLIN1		0.132	-0.045
G5E9W3	Cleavage and polyadenylation specific factor 3, 73kDa, isoform CRA_b	CPSF3	-0.013	0.048	-0.045
Q9H840	Gem-associated protein 7	GEMIN7	-0.183	-0.203	-0.045
P43487	Ran-specific GTPase-activating protein	RANBP1	-0.103	-0.158	-0.045
Q9Y6C9	Mitochondrial carrier homolog 2	MTCH2	0.088	0.127	-0.046
Q92599-2	Isoform 2 of Septin-8	SEPT8	0.013	0.149	-0.046
P06748	Nucleophosmin	NPM1	-0.017	0.035	-0.046
P35241	Radixin	RDX	-0.130	-0.112	-0.046
F8WBJ6	RNA-binding protein PNO1	PNO1	0.100	0.149	-0.046
O95633-2	Isoform 2 of Follistatin-related protein 3	FSTL3			-0.046
P42695	Condensin-2 complex subunit D3	NCAPD3	0.177	0.127	-0.046
Q9Y6A4	UPF0468 protein C16orf80	C16orf80	-0.125	-0.030	-0.046
Q9H5K3	Probable inactive protein kinase-like protein SgK196	SGK196	0.117	0.185	-0.046
Q9Y512	Sorting and assembly machinery component 50 homolog	SAMM50	-0.001	0.053	-0.046
Q8TDN6	Ribosome biogenesis protein BRX1 homolog	BRIX1	-0.066	-0.161	-0.047
D7R525	Mitogen-activated protein kinase 9	MAPK9	-0.031	0.117	-0.047
P38606	V-type proton ATPase catalytic subunit A	ATP6V1A	0.104	0.116	-0.047
Q92797	Symplekin	SYMPK	0.134	0.082	-0.047
P46736-4	Isoform 4 of Lys-63-specific deubiquitinase BRCC36	BRCC3	0.044	0.261	-0.047
Q9H0L4	Cleavage stimulation factor subunit 2 tau variant	CSTF2T	-0.044	0.175	-0.047
P82673	28S ribosomal protein S35, mitochondrial	MRPS35	-0.017	-0.028	-0.047
Q96M86	Dynein heavy chain domain-containing protein 1	DNHD1		-0.126	-0.047
Q7Z6Z7-2	Isoform 2 of E3 ubiquitin-protein ligase HUWE1	HUWE1	-0.069	-0.017	-0.047
Q9BQA9-2	Isoform 2 of Uncharacterized protein C17orf62	C17orf62	0.132		-0.048
Q14690	Protein RRP5 homolog	PDCD11	0.046	-0.007	-0.048
Q2T9J0-2	Isoform 2 of Peroxisomal leader peptide-processing protease	TYSND1			-0.048
Q53LP3	Ankyrin repeat domain-containing protein SOWAHC	SOWAHC	-0.072		-0.048
C9JQ41	Coiled-coil domain-containing protein 58	CCDC58	-0.010	-0.147	-0.048
Q01085	Nucleolysin TIAR	TIAL1	-0.007	0.008	-0.048
Q9Y5B9	FACT complex subunit SPT16	SUPT16H	-0.165	-0.417	-0.048
Q8IYJ9	DUSP3 protein	DUSP3	0.081	0.056	-0.049
P20645	Cation-dependent mannose-6-phosphate receptor	M6PR	0.168	-0.063	-0.049
Q96BN8	Protein FAM105B	FAM105B	0.027	-0.140	-0.049
Q9H0C8	Integrin-linked kinase-associated serine/threonine phosphatase 2C	ILKAP	0.188	-0.028	-0.049
P84077	ADP-ribosylation factor 1	ARF1	0.033	0.109	-0.049
Q9BQA1	Methylosome protein 50	WDR77	-0.061	-0.069	-0.049
D6R8Y9	Zinc finger protein 330 (Fragment)	ZNF330	-0.039	0.134	-0.049
H7BZ50	Mitotic-spindle organizing protein 2B (Fragment)	MZT2B	0.018	0.243	-0.050
Q15366-4	Isoform 4 of Poly(rC)-binding protein 2	PCBP2	-0.084	-0.099	-0.050
Q14103-3	Isoform 3 of Heterogeneous nuclear ribonucleoprotein D0	HNRNPD	-0.053	-0.248	-0.050
Q9NUQ9	Protein FAM49B	FAM49B	0.100	0.055	-0.050
Q96KA5-2	Isoform 2 of Cleft lip and palate transmembrane protein 1-like protein	CLPTM1L	0.016	0.168	-0.050
P61106	Ras-related protein Rab-14	RAB14	-0.019	0.101	-0.050
O43837	Isocitrate dehydrogenase [NAD] subunit beta, mitochondrial	IDH3B	-0.011	0.047	-0.050
F5H315	Pre-mRNA-splicing factor SYF1	XAB2	0.065	-0.130	-0.050
A6NML8	Diaphanous homolog 2 (Drosophila), isoform CRA_c	DIAPH2	0.022		-0.050
Q15758	Neutral amino acid transporter B(0)	SLC1A5	-0.367	0.023	-0.051
Q02790	Peptidyl-prolyl cis-trans isomerase FKBP4	FKBP4	-0.061	-0.057	-0.051
Q03252	Lamin-B2	LMNB2	0.084	-0.148	-0.051
Q07021	Complement component 1 Q subcomponent-binding protein, mitochondrial	C1QBP	-0.085	-0.060	-0.051
F5H5I6	G-rich sequence factor 1	GRSF1	0.013	-0.001	-0.051
Q9H488	GDP-fucose protein O-fucosyltransferase 1	POFUT1	-0.180	0.064	-0.052
Q9UKD2	mRNA turnover protein 4 homolog	MRTO4	0.057	0.048	-0.052
Q14677	Clathrin interactor 1	CLINT1	-0.157	0.008	-0.052
P62714	Serine/threonine-protein phosphatase 2A catalytic subunit beta isoform	PPP2CB			-0.052
P30085	UMP-CMP kinase	CMPK1	0.110	0.048	-0.052
Q14558	Phosphoribosyl pyrophosphate synthase-associated protein 1	PRPSAP1	0.002	0.215	-0.052
P04179	Superoxide dismutase [Mn], mitochondrial	SOD2	-0.250	0.271	-0.052
Q04837	Single-stranded DNA-binding protein, mitochondrial	SSBP1	-0.018	-0.218	-0.052
Q9Y3U8	60S ribosomal protein L36	RPL36	0.088	-0.107	-0.052
Q96EY1-2	Isoform 2 of DnaJ homolog subfamily A member 3, mitochondrial	DNAJA3	0.018	0.022	-0.052
Q96HE7	ERO1-like protein alpha	ERO1L	0.130	0.034	-0.052
C9J4G9	Beta-galactosidase (Fragment)	GLB1		-0.059	-0.052
Q12774	Rho guanine nucleotide exchange factor 5	ARHGEF5	-0.033		-0.052
K7ES30	Zinc finger protein 180	ZNF180			-0.052
Q8NDF8-2	Isoform 2 of PAP-associated domain-containing protein 5	PAPD5	-0.055		-0.052
A3KFL4	Exosome complex component RRP4	EXOSC2	-0.106	-0.001	-0.052
A8MX75	TFIIH basal transcription factor complex helicase XPD subunit (Fragment)	ERCC2	0.023	-0.216	-0.053
P36871	Phosphoglucomutase-1	PGM1	-0.080	0.187	-0.053
Q96PZ0	Pseudouridylate synthase 7 homolog	PUS7	-0.126	0.011	-0.053
P27695	DNA-(apurinic or apyrimidinic site) lyase	APEX1	-0.106	-0.005	-0.053
Q9H773	dCTP pyrophosphatase 1	DCTPP1	0.024	-0.166	-0.053
Q8IY81	pre-rRNA processing protein FTSJ3	FTSJ3	0.066	-0.074	-0.053
P07900	Heat shock protein HSP 90-alpha	HSP90AA1	-0.109	-0.115	-0.053
Q92688-2	Isoform 2 of Acidic leucine-rich nuclear phosphoprotein 32 family member B	ANP32B	-0.002	-0.035	-0.053
Q14157-4	Isoform 4 of Ubiquitin-associated protein 2-like	UBAP2L	-0.045	-0.026	-0.053
Q9HC07	Transmembrane protein 165	TMEM165	0.163	0.081	-0.053
Q7L014	Probable ATP-dependent RNA helicase DDX46	DDX46	-0.081	-0.002	-0.054
Q01105	Protein SET	SET	-0.088	-0.196	-0.054

Q92879-5	Isoform 5 of CUGBP Elav-like family member 1	CELF1	-0.081	-0.072	-0.054
Q9Y2P4	Long-chain fatty acid transport protein 6	SLC27A6			-0.055
Q8WW59	SPRY domain-containing protein 4	SPRYD4	0.194	0.125	-0.055
P19367-4	Isoform 4 of Hexokinase-1	HK1	0.021	0.082	-0.055
P62136	Serine/threonine-protein phosphatase PP1-alpha catalytic subunit	PPP1CA	-0.068	-0.153	-0.055
P10620	Microsomal glutathione S-transferase 1	MGST1	-0.032	0.077	-0.055
Q16658	Fascin	FSCN1	-0.120	-0.366	-0.055
O00299	Chloride intracellular channel protein 1	CLIC1	-0.010	-0.007	-0.055
Q96ST3	Paired amphipathic helix protein Sin3a	SIN3A	0.120	0.228	-0.056
Q9C0C2	182 kDa tankyrase-1-binding protein	TNKS1BP1	0.075	0.065	-0.056
P78371	T-complex protein 1 subunit beta	CCT2	-0.065	-0.161	-0.056
Q9Y5X3	Sorting nexin-5	SNX5	0.162	0.057	-0.056
O00442	RNA 3'-terminal phosphate cyclase	RTCA	-0.066	-0.047	-0.056
P37837	Transaldolase	TALDO1	-0.048	-0.019	-0.056
E7EWE1	Ubiquitin-like modifier-activating enzyme 5	UBA5	-0.040	0.102	-0.056
O00411	DNA-directed RNA polymerase, mitochondrial	POLRMT	-0.028		-0.056
P63000	Ras-related C3 botulinum toxin substrate 1	RAC1	-0.054	-0.020	-0.057
Q9H307	Pinin	PNN	-0.228	-0.657	-0.057
P62873	Guanine nucleotide-binding protein G(I)/G(S)/G(T) subunit beta-1	GNB1	0.070	0.016	-0.057
Q96KP4	Cytosolic non-specific dipeptidase	CNDP2	0.037	0.335	-0.057
P10155-3	Isoform 3 of 60 kDa SS-A/Ro ribonucleoprotein	TROVE2	-0.012	0.124	-0.057
P09382	Galectin-1	LGALS1	0.150	0.053	-0.057
O75419-2	Isoform 2 of Cell division control protein 45 homolog	CDC45	0.056	-0.047	-0.057
O95361	Tripartite motif-containing protein 16	TRIM16	0.009		-0.057
O15091-4	Isoform 4 of Mitochondrial ribonuclease P protein 3	KIAA0391		0.121	-0.057
Q5VWZ2-2	Isoform 2 of Lysophospholipase-like protein 1	LYPLAL1	0.014		-0.057
P53680	AP-2 complex subunit sigma	AP2S1	0.059	-0.074	-0.057
P46934-4	Isoform 4 of E3 ubiquitin-protein ligase NEDD4	NEDD4	0.143		-0.057
Q6I9Y2	THO complex subunit 7 homolog	THOC7	0.098	-0.160	-0.058
P51151	Ras-related protein Rab-9A	RAB9A	0.063	0.331	-0.058
A6NLH6	Protein cornichon homolog 4	CNIH4	0.204	0.058	-0.058
Q9C005	Protein dpy-30 homolog	DPY30	0.031	-0.061	-0.058
Q12933-3	Isoform 3 of TNF receptor-associated factor 2	TRAF2	-0.154	-0.592	-0.058
Q12874	Splicing factor 3A subunit 3	SF3A3	0.033	-0.094	-0.058
O00571	ATP-dependent RNA helicase DDX3X	DDX3X	-0.086	-0.043	-0.058
Q96CW1-2	Isoform 2 of AP-2 complex subunit mu	AP2M1	0.090	0.070	-0.058
O15357-2	Isoform 2 of Phosphatidylinositol 3,4,5-trisphosphate 5-phosphatase 2	INPPL1	-0.116	0.058	-0.058
P35573-2	Isoform 5 of Glycogen debranching enzyme	AGL	-0.062		-0.058
Q99570	Phosphoinositide 3-kinase regulatory subunit 4	PIK3R4	-0.389	-0.004	-0.058
H7C5G1	Isoamyl acetate-hydrolyzing esterase 1 homolog (Fragment)	IAH1		0.049	-0.058
Q6IAA8	Ragulator complex protein LAMTOR1	LAMTOR1	-0.443	-0.342	-0.058
P62910	60S ribosomal protein L32	RPL32	-0.102	-0.095	-0.059
Q86XA6	B4GALT1 protein	B4GALT1	-0.088	-0.101	-0.059
H7C515	Protein CMSS1 (Fragment)	CMSS1	-0.100	0.073	-0.059
P0C0S5	Histone H2A.Z	H2AFZ	0.154	-0.215	-0.059
P78527	DNA-dependent protein kinase catalytic subunit	PRKDC	-0.047	-0.029	-0.059
P38117	Electron transfer flavoprotein subunit beta	ETFB	0.027	0.082	-0.059
Q9Y265	RuvB-like 1	RUVBL1	-0.029	-0.106	-0.059
P28331	NADH-ubiquinone oxidoreductase 75 kDa subunit, mitochondrial	NDUFS1	0.137	0.043	-0.060
P62820	Ras-related protein Rab-1A	RAB1A	0.075	0.089	-0.060
P61758	Prefoldin subunit 3	VBP1	-0.123	-0.108	-0.060
Q96L92-2	Isoform 3 of Sorting nexin-27	SNX27		0.052	-0.060
Q9NY93-2	Isoform 2 of Probable ATP-dependent RNA helicase DDX56	DDX56	0.047	-0.052	-0.060
Q9NRX2	39S ribosomal protein L17, mitochondrial	MRPL17	0.034	0.172	-0.060
Q8NCW5	NAD(P)H-hydrate epimerase	APOA1BP	0.015	0.158	-0.060
D3DQV9	Eukaryotic translation initiation factor 4 gamma 2 (Fragment)	EIF4G2	-0.182	0.018	-0.060
C9JX92	Afadin	MLLT4	0.133	0.014	-0.060
F2Z2X4	Exportin-4	XPO4	0.018	0.021	-0.060
Q9BZK7	F-box-like/WD repeat-containing protein TBL1XR1	TBL1XR1	-0.016	-0.015	-0.060
B1AH59	Activating signal cointegrator 1 complex subunit 2 (Fragment)	ASCC2	-0.107		-0.060
P52701	DNA mismatch repair protein Msh6	MSH6	-0.085	-0.079	-0.060
P29144	Tripeptidyl-peptidase 2	TPP2	-0.005	-0.009	-0.061
Q99497	Protein DJ-1	PARK7	-0.035	-0.015	-0.061
P82663	28S ribosomal protein S25, mitochondrial	MRPS25	-0.180	-0.036	-0.061
P25789	Proteasome subunit alpha type-4	PSMA4	-0.126	-0.069	-0.061
Q9NZL4	Hsp70-binding protein 1	HSPBP1	-0.069	0.030	-0.062
P06396-2	Isoform 2 of Gelsolin	GSN	0.232	0.185	-0.062
Q9P0J0	NADH dehydrogenase [ubiquinone] 1 alpha subcomplex subunit 13	NDUFA13	0.030	0.099	-0.062
Q14160	Protein scribble homolog	SCRIB	-0.021	-0.038	-0.062
P62847-2	Isoform 2 of 40S ribosomal protein S24	RPS24	0.004	0.117	-0.062
P53597	Succinyl-CoA ligase [ADP/GDP-forming] subunit alpha, mitochondrial	SUCLG1	0.029	0.069	-0.062
Q9P0L0	Vesicle-associated membrane protein-associated protein A	VAPA	-0.033	0.038	-0.062
P46926	Glucosamine-6-phosphate isomerase 1	GNPDA1	-0.142	0.117	-0.063
Q9Y4R8	Telomere length regulation protein TEL2 homolog	TELO2	-0.222	-0.114	-0.063
O43795-2	Isoform 2 of Unconventional myosin-Ib	MYO1B	-0.098		-0.063
O75947	ATP synthase subunit d, mitochondrial	ATP5H	-0.026	0.091	-0.063
Q9UNX3	60S ribosomal protein L26-like 1	RPL26L1	-0.039	0.028	-0.063
Q9UBQ0	Vacuolar protein sorting-associated protein 29	VPS29	-0.120	0.012	-0.063
E7ERV9	Acid ceramidase	ASAH1			-0.063
Q4G0N4	NAD kinase domain-containing protein 1, mitochondrial	NADKD1	-0.153	-0.086	-0.063
Q96AE4-2	Isoform 2 of Far upstream element-binding protein 1	FUBP1	0.236	0.205	-0.063
Q8WUP2	Filamin-binding LIM protein 1	FBLIM1	0.205		-0.063
P61221	ATP-binding cassette sub-family E member 1	ABCE1	-0.377	-0.133	-0.064
Q9Y6M1-1	Isoform 2 of Insulin-like growth factor 2 mRNA-binding protein 2	IGF2BP2	-0.514	-0.127	-0.064
Q9HD45	Transmembrane 9 superfamily member 3	TM9SF3	0.009	0.094	-0.064
Q96C36	Pyrroline-5-carboxylate reductase 2	PYCR2	0.079	0.110	-0.064
Q66K14-2	Isoform 2 of TBC1 domain family member 9B	TBC1D9B	0.040	-0.120	-0.064

P06730	Eukaryotic translation initiation factor 4E	EIF4E	0.000	-0.073	-0.064
O75663	TIP41-like protein	TIPRL	-0.168	-0.009	-0.064
H7C1N7	Selenocysteine lyase (Fragment)	SCLY			-0.064
P62081	40S ribosomal protein S7	RPS7	-0.134	-0.063	-0.065
Q13428-3	Isoform 3 of Treacle protein	TCOF1	0.084	0.029	-0.065
M0QY97	Zinc finger CCCH domain-containing protein 4 (Fragment)	ZC3H4	-0.038	-0.054	-0.065
Q99797	Mitochondrial intermediate peptidase	MIPEP			-0.065
P31948	Stress-induced-phosphoprotein 1	STIP1	-0.069	-0.032	-0.065
Q9Y4E8-2	Isoform 2 of Ubiquitin carboxyl-terminal hydrolase 15	USP15	0.060	0.040	-0.065
Q9NV31	U3 small nucleolar ribonucleoprotein protein IMP3	IMP3	0.003	-0.189	-0.066
Q5VZK9-2	Isoform 2 of Leucine-rich repeat-containing protein 16A	LRRC16A	0.097		-0.066
Q5TZP9	Cyclin B1	CCNB1	-0.015		-0.066
Q86SQ0-2	Isoform 2 of Pleckstrin homology-like domain family B member 2	PHLDB2	0.226		-0.066
O75608-2	Isoform 2 of Acyl-protein thioesterase 1	LYPLA1	-0.004	0.071	-0.067
P36969-2	Isoform Cytoplasmic of Phospholipid hydroperoxide glutathione peroxidase, mitocl	GPX4		0.083	-0.067
Q96AT1	Uncharacterized protein KIAA1143	KIAA1143	-0.194	-0.085	-0.067
Q08378-2	Isoform 2 of Golgin subfamily A member 3	GOLGA3	0.199		-0.067
H0YIV9	Uncharacterized protein (Fragment)	3	-0.014		-0.067
Q92621	Nuclear pore complex protein Nup205	NUP205	0.053	-0.005	-0.067
Q5T8P6-5	Isoform 5 of RNA-binding protein 26	RBM26	0.100	-0.600	-0.067
Q9UDW1-2	Isoform 2 of Cytochrome b-c1 complex subunit 9	UQCR10	0.157	0.073	-0.067
Q08AM6	Protein VAC14 homolog	VAC14	-0.292	-0.079	-0.068
Q02543	60S ribosomal protein L18a	RPL18A	-0.121	-0.145	-0.068
P62328	Thymosin beta-4	TMSB4X	-0.244	-0.499	-0.068
Q96EK6	Glucosamine 6-phosphate N-acetyltransferase	GNPNAT1	-0.182	-0.089	-0.068
P09429	High mobility group protein B1	HMGB1	-0.141	-0.006	-0.068
O75643	U5 small nuclear ribonucleoprotein 200 kDa helicase	SNRNP200	0.026	-0.146	-0.069
P28288	ATP-binding cassette sub-family D member 3	ABCD3	-0.056	-0.081	-0.069
Q9NVH1-3	Isoform 3 of DnaJ homolog subfamily C member 11	DNAJC11	-0.146	-0.042	-0.069
Q96P70	Importin-9	IPO9	0.007	-0.076	-0.069
P62841	40S ribosomal protein S15	RPS15	-0.091	-0.080	-0.069
P16403	Histone H1.2	HIST1H1C	-0.201	-0.019	-0.069
P38646	Stress-70 protein, mitochondrial	HSPA9	-0.016	-0.023	-0.069
P52948-6	Isoform 6 of Nuclear pore complex protein Nup98-Nup96	NUP98	0.065	-0.023	-0.069
Q13620-3	Isoform 3 of Cullin-4B	CUL4B	0.021	0.119	-0.070
E9PM92	Small acidic protein (Fragment)	C11orf58	-0.009	-0.130	-0.070
Q9HBL7	Plasminogen receptor (KT)	PLGRKT			-0.070
Q8TEA8	D-tyrosyl-tRNA(Tyr) deacylase 1	DTD1	0.177	-0.224	-0.070
Q9UG63	ATP-binding cassette sub-family F member 2	ABCF2	-0.057	-0.002	-0.070
O95292	Vesicle-associated membrane protein-associated protein B/C	VAPB	0.196	0.122	-0.070
H3BRK1	Poly(A)-specific ribonuclease PARN (Fragment)	PARN	-0.101	0.069	-0.070
B4DZ67	Nuclear pore complex protein Nup107	NUP107	0.048	-0.034	-0.070
P45974-2	Isoform Short of Ubiquitin carboxyl-terminal hydrolase 5	USP5	0.011	-0.069	-0.070
P29992	Guanine nucleotide-binding protein subunit alpha-11	GNA11	0.027	-0.034	-0.070
P54920	Alpha-soluble NSF attachment protein	NAPA	-0.019	0.070	-0.071
Q8NBJ4-2	Isoform 2 of Golgi membrane protein 1	GOLM1	0.121	0.108	-0.071
H7C022	WD repeat-containing protein 60 (Fragment)	WDR60			-0.071
Q92504	Zinc transporter SLC39A7	SLC39A7	0.036	0.268	-0.071
Q53GQ0	Estradiol 17-beta-dehydrogenase 12	HSD17B12	-0.111	0.026	-0.071
O60832	H/ACA ribonucleoprotein complex subunit 4	DKC1	-0.004	-0.009	-0.072
P27449	V-type proton ATPase 16 kDa proteolipid subunit	ATP6V0C	0.017	0.003	-0.072
Q92616	Translational activator GCN1	GCN1L1	0.010	0.101	-0.072
Q00653-4	Isoform 4 of Nuclear factor NF-kappa-B p100 subunit	NFKB2		0.131	-0.072
Q8IZ83	Aldehyde dehydrogenase family 16 member A1	ALDH16A1	0.164	0.097	-0.072
Q8TEQ6	Gem-associated protein 5	GEMIN5	-0.027	-0.079	-0.073
P25815	Protein S100-P	S100P	0.797		-0.073
O95140	Mitofusin-2	MFN2	0.088	0.122	-0.073
Q13867	Bleomycin hydrolase	BLMH	-0.133	-0.235	-0.073
P55884	Eukaryotic translation initiation factor 3 subunit B	EIF3B	-0.152	-0.032	-0.073
O95817	BAG family molecular chaperone regulator 3	BAG3	-0.077	-1.119	-0.073
Q29RF7	Sister chromatid cohesion protein PDS5 homolog A	PDS5A	-0.023	-0.076	-0.073
P56537	Eukaryotic translation initiation factor 6	EIF6	-0.009	-0.296	-0.074
Q8N5L8	Ribonuclease P protein subunit p25-like protein	RPP25L	-0.044		-0.074
P49023-2	Isoform Alpha of Paxillin	PXN	-0.038		-0.074
P10809	60 kDa heat shock protein, mitochondrial	HSPD1	-0.044	-0.038	-0.074
J3QLE5	Small nuclear ribonucleoprotein-associated protein N (Fragment)	SNRPN	-0.040	-0.055	-0.074
Q8N1G4	Leucine-rich repeat-containing protein 47	LRRC47	0.010	0.040	-0.074
P67936-2	Isoform 2 of Tropomyosin alpha-4 chain	TPM4	0.264		-0.075
Q9H0A0	N-acetyltransferase 10	NAT10	0.056	-0.086	-0.075
P06753-5	Isoform 5 of Tropomyosin alpha-3 chain	TPM3	0.256	-0.027	-0.075
P49750	YLP motif-containing protein 1	YLPM1	0.008	-0.024	-0.075
P03886	NADH-ubiquinone oxidoreductase chain 1	MT-ND1	-0.002	-0.182	-0.075
P50336	Protoporphyrinogen oxidase	PPOX	0.222		-0.075
P62829	60S ribosomal protein L23	RPL23	-0.022	-0.077	-0.075
Q9BS26	Endoplasmic reticulum resident protein 44	ERP44	0.214	0.159	-0.076
Q8IWR0	Zinc finger CCCH domain-containing protein 7A	ZC3H7A		0.321	-0.076
O14818	Proteasome subunit alpha type-7	PSMA7	-0.051	-0.008	-0.076
Q08379	Golgin subfamily A member 2	GOLGA2	-0.016		-0.076
E9PFK5	Nucleolar protein 14	NOP14		0.044	-0.076
P41252	Isoleucine--tRNA ligase, cytoplasmic	IARS	0.001	-0.030	-0.076
Q9UKY7	Protein CDV3 homolog	CDV3	-0.131	-0.038	-0.076
P51398-2	Isoform 2 of 28S ribosomal protein S29, mitochondrial	DAP3	-0.108	0.040	-0.076
D6RAD4	Cyclin-dependent kinase 7	CDK7			-0.077
Q15075	Early endosome antigen 1	EEA1	-0.208	-0.034	-0.077
O60264	SWI/SNF-related matrix-associated actin-dependent regulator of chromatin subfar	SMARCA5	0.092	-0.431	-0.077
Q9UHV9	Prefoldin subunit 2	PFDN2	-0.102	-0.051	-0.077
P03915	NADH-ubiquinone oxidoreductase chain 5	MT-ND5		0.358	-0.078

Q9BRJ2	39S ribosomal protein L45, mitochondrial	MRPL45	0.014	-0.079	-0.078
P25705	ATP synthase subunit alpha, mitochondrial	ATP5A1	0.027	0.167	-0.078
P11766	Alcohol dehydrogenase class-3	ADH5	0.042	0.095	-0.078
Q9BTW9	Tubulin-specific chaperone D	TBCD	0.135	0.073	-0.078
P62310	U6 snRNA-associated Sm-like protein LSm3	LSM3	-0.055		-0.078
P12270	Nucleoprotein TPR	TPR	0.096	0.004	-0.078
O95155-2	Isoform 2 of Ubiquitin conjugation factor E4 B	UBE4B		-0.095	-0.078
O95295	SNARE-associated protein Snapin	SNAPIN			-0.078
P23284	Peptidyl-prolyl cis-trans isomerase B	PPIB	-0.017	-0.035	-0.079
P14923	Junction plakoglobin	JUP	0.213	0.149	-0.079
O96008	Mitochondrial import receptor subunit TOM40 homolog	TOMM40	-0.005	0.056	-0.079
Q9Y333	U6 snRNA-associated Sm-like protein LSm2	LSM2	-0.173	-0.230	-0.079
O15235	28S ribosomal protein S12, mitochondrial	MRPS12	0.054	0.240	-0.079
C9JG87	39S ribosomal protein L39, mitochondrial (Fragment)	MRPL39	-0.024	-0.193	-0.080
P61326	Protein mago nashi homolog	MAGOH	-0.052	-0.210	-0.080
O43776	Asparagine--tRNA ligase, cytoplasmic	NARS	0.032	-0.019	-0.080
P30042	ES1 protein homolog, mitochondrial	C21orf33	-0.047	0.104	-0.080
O75694	Nuclear pore complex protein Nup155	NUP155	-0.001	-0.006	-0.080
B4DLH2	Chromosome 2 open reading frame 18, isoform CRA_c	C2orf18	0.067	0.006	-0.080
P47813	Eukaryotic translation initiation factor 1A, X-chromosomal	EIF1AX	-0.111	-0.193	-0.080
Q9Y5U9	Immediate early response 3-interacting protein 1	IER3IP1		0.130	-0.080
P34897-3	Isoform 3 of Serine hydroxymethyltransferase, mitochondrial	SHMT2	0.035	0.044	-0.080
Q15386	Ubiquitin-protein ligase E3C	UBE3C	0.357		-0.080
Q7Z7F7	39S ribosomal protein L55, mitochondrial	MRPL55			-0.080
Q8IXM3	39S ribosomal protein L41, mitochondrial	MRPL41	-0.039	0.097	-0.080
F5H7B0	Helicase SKI2W	SKIV2L		0.243	-0.081
H7C2M7	Armadillo repeat-containing protein 10 (Fragment)	ARMC10			-0.081
P11387	DNA topoisomerase 1	TOP1	0.002	-0.051	-0.081
P56589	Peroxisomal biogenesis factor 3	PEX3		-0.069	-0.082
O94826	Mitochondrial import receptor subunit TOM70	TOMM70A	-0.043	0.018	-0.082
Q9Y446	Plakophilin-3	PKP3	-0.237	-0.065	-0.082
Q9NUQ3	Gamma-taxilin	TXLNG	0.040	-0.061	-0.082
P48506	Glutamate--cysteine ligase catalytic subunit	GCLC		0.033	-0.082
H0Y588	EF-hand calcium-binding domain-containing protein 2 (Fragment)	EFCAB2	0.162	0.029	-0.082
D6RBJ9	Deoxycytidylate deaminase (Fragment)	DCTD	-0.103	0.085	-0.083
Q9BUK6-7	Isoform 7 of Protein misato homolog 1	MSTO1		0.014	-0.083
F8VZN8	Protein phosphatase 1 regulatory subunit 12A (Fragment)	PPP1R12A	-0.011	-0.187	-0.083
P39687	Acidic leucine-rich nuclear phosphoprotein 32 family member A	ANP32A	-0.254		-0.083
O00170	AH receptor-interacting protein	AIP	0.089	-0.122	-0.083
B4DXV1	Elongator complex protein 3	ELP3	-0.044		-0.083
P09001	39S ribosomal protein L3, mitochondrial	MRPL3	0.065	-0.279	-0.083
Q6ZRP7	Sulfhydryl oxidase 2	QSOX2		0.074	-0.084
B4DVJ1	Sn1-specific diacylglycerol lipase beta	DAGLB	0.003	0.200	-0.084
Q69YN4-2	Isoform 2 of Protein virilizer homolog	KIAA1429	-0.138		-0.084
P41250	Glycine--tRNA ligase	GARS	0.089	-0.016	-0.084
H0YN81	WD repeat-containing protein 61 (Fragment)	WDR61	0.020	-0.022	-0.085
Q9Y266	Nuclear migration protein nudC	NUDC	-0.151	-0.078	-0.085
O00487	26S proteasome non-ATPase regulatory subunit 14	PSMD14	-0.112	-0.139	-0.085
Q8NC51	Plasminogen activator inhibitor 1 RNA-binding protein	SERBP1		0.077	-0.085
O15173	Membrane-associated progesterone receptor component 2	PGRMC2	0.354	0.048	-0.085
Q12904	Aminoacyl tRNA synthase complex-interacting multifunctional protein 1	AIMP1	0.053	0.032	-0.085
B4E2X3	Methyltransferase-like protein 13	METTL13	-0.085	0.135	-0.086
Q9UK59	Lariat debranching enzyme	DBR1	0.168	0.106	-0.086
Q9BV38	WD repeat-containing protein 18	WDR18	-0.002	-0.079	-0.086
B3KSI3	Branched-chain-amino-acid aminotransferase	BCAT2	0.144	0.177	-0.086
P50454	Serpin H1	SERPINH1	0.084	0.172	-0.087
F5H897	Heat shock protein 75 kDa, mitochondrial	TRAP1	-0.016	-0.065	-0.087
E7ERS5	Serine/threonine-protein kinase VRK2 (Fragment)	VRK2	-0.035		-0.087
F5H5N1	NADH dehydrogenase [ubiquinone] iron-sulfur protein 7, mitochondrial	NDUFS7		0.100	-0.087
H0Y614	Ubiquitin-fold modifier 1 (Fragment)	UFM1	-0.211	-0.211	-0.087
Q9NRN7	L-aminoadipate-semialdehyde dehydrogenase-phosphopantetheinyl transferase	AASDHPPT	-0.158	-0.248	-0.087
Q9UKL0	REST corepressor 1	RCOR1	-0.010	-0.186	-0.087
P18206-2	Isoform 1 of Vinculin	VCL	0.013	-0.015	-0.087
Q6ZS74	Ras-related protein Ral-B	RALB	0.034		-0.087
Q7L2E3-3	Isoform 3 of Putative ATP-dependent RNA helicase DHX30	DHX30	-0.081	-0.001	-0.088
O14561	Acyl carrier protein, mitochondrial	NDUFAB1	-0.029	0.059	-0.088
G3V159	Hermansky-Pudlak syndrome 5 protein	HPS5			-0.088
A3KMH1-2	Isoform 2 of von Willebrand factor A domain-containing protein 8	VWA8	-0.126		-0.088
P16152	Carbonyl reductase [NADPH] 1	CBR1	-0.089		-0.088
Q9Y3C8	Ubiquitin-fold modifier-conjugating enzyme 1	UFC1	-0.024	-0.079	-0.088
Q13492-3	Isoform 3 of Phosphatidylinositol-binding clathrin assembly protein	PICALM	0.012	-0.188	-0.088
Q9NZT2-2	Isoform 2 of Opioid growth factor receptor	OGFR	-0.047	0.018	-0.088
E9PB14	Pyruvate dehydrogenase protein X component, mitochondrial	PDHX	0.051	0.247	-0.089
A0AVT1	Ubiquitin-like modifier-activating enzyme 6	UBA6	-0.046	0.053	-0.089
G5EA06	28S ribosomal protein S27, mitochondrial	MRPS27	0.038	0.223	-0.089
E9PHT6	Pantothenate kinase 4	PANK4	0.700	-0.074	-0.089
Q9P265	Disco-interacting protein 2 homolog B	DIP2B	0.274	-0.052	-0.089
P09622	Dihydrolipoyl dehydrogenase, mitochondrial	DLD	0.006	0.133	-0.089
E7EM64	COP9 signalosome complex subunit 6	COPS6	-0.033	0.042	-0.089
Q9BXW7-2	Isoform 1 of Cat eye syndrome critical region protein 5	CECR5	-0.021	0.052	-0.089
Q13442	28 kDa heat- and acid-stable phosphoprotein	PDAP1	-0.043	-0.186	-0.089
Q13011	Delta(3,5)-Delta(2,4)-dienoyl-CoA isomerase, mitochondrial	ECH1	0.318	0.064	-0.089
Q9HB90	Ras-related GTP-binding protein C	RRAGC	0.086	0.001	-0.089
Q5BKT4	Dol-P-Glc:Glc(2)Man(9)GlcNAc(2)-PP-Dol alpha-1,2-glucosyltransferase	ALG10			-0.090
P14174	Macrophage migration inhibitory factor	MIF	-0.139	-0.308	-0.090
O00273	DNA fragmentation factor subunit alpha	DFFA	0.107	0.025	-0.090
Q8WXI9	Transcriptional repressor p66-beta	GATAD2B		0.034	-0.090

Q9BRG1	Vacuolar protein-sorting-associated protein 25	VPS25	0.026	0.173	-0.090
Q9NRP0	Oligosaccharyltransferase complex subunitTC	OSTC	0.071		-0.091
Q9H0P0-3	Isoform 4 of Cytosolic 5'-nucleotidase 3A	NT5C3A	-0.047		-0.091
P21399	Cytoplasmic aconitate hydratase	ACO1	-0.193	0.267	-0.092
O75489	NADH dehydrogenase [ubiquinone] iron-sulfur protein 3, mitochondrial	NDUFS3	0.012	-0.014	-0.092
Q9UQ35	Serine/arginine repetitive matrix protein 2	SRRM2	-0.016	-0.015	-0.092
Q9UPT9-2	Isoform 2 of Ubiquitin carboxyl-terminal hydrolase 22	USP22	-0.058	-0.055	-0.092
P15514	Amphiregulin	AREG			-0.093
Q15819	Ubiquitin-conjugating enzyme E2 variant 2	UBE2V2	-0.094	-0.055	-0.093
P82675	28S ribosomal protein S5, mitochondrial	MRPS5	0.012	-0.070	-0.093
Q71UM5	40S ribosomal protein S27-like	RPS27L	0.295	0.317	-0.093
O60716-21	Isoform 3A of Catenin delta-1	CTNND1	0.007	0.029	-0.093
E7EQT4	Apoptotic chromatin condensation inducer in the nucleus	ACIN1	-0.084	-0.341	-0.093
Q15019	Septin-2	SEPT2	0.137	0.017	-0.094
Q9NR28-2	Isoform 2 of Diablo homolog, mitochondrial	DIABLO	0.088	0.063	-0.094
B4DJP7	Small nuclear ribonucleoprotein Sm D3	SNRPD3	-0.052	-0.232	-0.094
P05165-3	Isoform 3 of Propionyl-CoA carboxylase alpha chain, mitochondrial	PCCA			-0.094
F5H4C6	N-acetylglucosamine-6-sulfatase (Fragment)	GNS		0.250	-0.094
Q9BUB7	Transmembrane protein 70, mitochondrial	TMEM70		-0.015	-0.095
P49406	39S ribosomal protein L19, mitochondrial	MRPL19	-0.121	-0.053	-0.095
P19623	Spermidine synthase	SRM	-0.099	-0.141	-0.095
Q6NXR4	TELO2-interacting protein 2	TTI2	-0.078	-0.089	-0.095
B4DXI5	2-hydroxyacyl-CoA lyase 1	HACL1	0.102	0.149	-0.095
Q15437	Protein transport protein Sec23B	SEC23B	-0.065	0.074	-0.095
H3BRV0	Eukaryotic translation initiation factor 3 subunit C	EIF3C	-0.036	-0.038	-0.095
O14545	TRAF-type zinc finger domain-containing protein 1	TRAFD1	0.040	0.000	-0.095
Q6FI81	Anamorsin	CIAPIN1	-0.015	0.012	-0.095
Q15427	Splicing factor 3B subunit 4	SF3B4	0.121	0.021	-0.096
Q6PI48	Aspartate--tRNA ligase, mitochondrial	DARS2	0.096	-0.023	-0.096
Q96IU4	Alpha/beta hydrolase domain-containing protein 14B	ABHD14B	0.007	0.001	-0.096
P11177-2	Isoform 2 of Pyruvate dehydrogenase E1 component subunit beta, mitochondrial	PDHB	0.019	0.061	-0.096
Q9Y2A7	Nck-associated protein 1	NCKAP1	0.020	0.040	-0.096
O15226	NF-kappa-B-repressing factor	NKRF	0.031	-0.180	-0.096
Q8WVJ2	NudC domain-containing protein 2	NUDCD2	0.025	-0.099	-0.096
G3XAH6	Poly(A) polymerase alpha	PAPOLA	0.002	0.082	-0.097
Q92841	Probable ATP-dependent RNA helicase DDX17	DDX17	-0.127	-0.109	-0.097
O94906	Pre-mRNA-processing factor 6	PRPF6	0.002	0.016	-0.097
Q15149	Plectin	PLEC			-0.097
E9PN51	NADH dehydrogenase [ubiquinone] iron-sulfur protein 8, mitochondrial (Fragment	NDUFS8	-0.298	0.215	-0.097
Q6R327	Rapamycin-insensitive companion of mTOR	RICTOR	0.159		-0.097
O75915	PRA1 family protein 3	ARL6IP5	0.219	0.131	-0.098
O00748-2	Isoform 2 of Cocaine esterase	CES2			-0.098
Q8IYS1	Peptidase M20 domain-containing protein 2	PM20D2	0.027		-0.098
Q5T655	Coiled-coil domain-containing protein 147	CCDC147			-0.098
C9JNK6	Metaxin-2	MTX2	0.019	0.185	-0.098
H7C1F0	Interleukin-1 receptor-associated kinase 1 (Fragment)	IRAK1	0.258		-0.098
F5GXJ9	CD166 antigen	ALCAM		-0.070	-0.098
Q9H2U2	Inorganic pyrophosphatase 2, mitochondrial	PPA2	0.180	0.084	-0.099
P22033	Methylmalonyl-CoA mutase, mitochondrial	MUT	0.138	0.059	-0.099
Q9UHX1-2	Isoform 2 of Poly(U)-binding-splicing factor PUF60	PUF60	-0.072	0.057	-0.099
B7ZAM9	Eukaryotic translation initiation factor 3 subunit K	EIF3K	-0.072	-0.072	-0.099
Q96QC0	Serine/threonine-protein phosphatase 1 regulatory subunit 10	PPP1R10	0.076	0.260	-0.099
E5RGP3	Pleckstrin homology domain-containing family A member 2 (Fragment)	PLEKHA2			-0.099
J3KSI8	28S ribosomal protein S7, mitochondrial (Fragment)	MRPS7	-0.043	-0.155	-0.100
P25325	3-mercaptopyruvate sulfurtransferase	MPST	0.113	0.058	-0.100
E7EV59	V-type proton ATPase subunit C 1	ATP6V1C1	0.023	0.234	-0.100
C9JNW5	60S ribosomal protein L24	RPL24	0.178	0.142	-0.100
R4GN43	NADH dehydrogenase [ubiquinone] 1 alpha subcomplex subunit 6	NDUFA6	0.032	0.042	-0.100
Q969X5-2	Isoform 2 of Endoplasmic reticulum-Golgi intermediate compartment protein 1	ERGIC1	-0.114	-0.185	-0.101
E9PRV2	Yorkie homolog	YAP1	0.056		-0.101
Q96GQ7	Probable ATP-dependent RNA helicase DDX27	DDX27	0.030	-0.185	-0.101
Q9UK45	U6 snRNA-associated Sm-like protein LSm7	LSM7	-0.042	-0.252	-0.101
Q8WY22	BRI3-binding protein	BRI3BP	-0.304	0.052	-0.101
O14684	Prostaglandin E synthase	PTGES	0.253	0.525	-0.101
Q9H788	SH2 domain-containing protein 4A	SH2D4A	-0.026		-0.102
P55145	Mesencephalic astrocyte-derived neurotrophic factor	MANF	0.176	-0.077	-0.102
H7C4E5	Cytochrome c oxidase copper chaperone (Fragment)	COX17	-0.134	-0.190	-0.102
P08579	U2 small nuclear ribonucleoprotein B''	SNRPB2	0.286	-0.022	-0.102
Q8WTS6	Histone-lysine N-methyltransferase SETD7	SETD7	0.018		-0.102
H0YNG3	Signal peptidase complex catalytic subunit SEC11A	SEC11A	0.097	0.182	-0.102
Q9UIQ6-3	Isoform 3 of Leucyl-cystinyl aminopeptidase	LNPEP		0.086	-0.103
P46108-2	Isoform Crk-I of Adapter molecule crk	CRK	0.024	-0.251	-0.103
Q08211	ATP-dependent RNA helicase A	DHX9	-0.004	-0.242	-0.103
Q5RKV6	Exosome complex component MTR3	EXOSC6	-0.122	0.032	-0.103
Q9Y4W6	AFG3-like protein 2	AFG3L2	0.069	0.163	-0.103
Q2NL82	Pre-rRNA-processing protein TSR1 homolog	TSR1	-0.033	-0.046	-0.103
C9IZ04	Serine/threonine-protein phosphatase 4 regulatory subunit 2 (Fragment)	PPP4R2	-0.062	0.016	-0.103
P36915	Guanine nucleotide-binding protein-like 1	GNL1	-0.029	0.103	-0.104
Q5JR04	Mov10, Moloney leukemia virus 10, homolog (Mouse), isoform CRA_a	MOV10	-0.074		-0.104
P17931	Galectin-3	LGALS3	0.283	0.731	-0.104
E7EQG2	Eukaryotic initiation factor 4A-II	EIF4A2	-0.042	-0.131	-0.104
Q8N5M9	Protein jagunal homolog 1	JAGN1	-0.248	-0.050	-0.104
Q13838	Spliceosome RNA helicase DDX39B	DDX39B	-0.049	-0.121	-0.105
Q5UCC4-2	Isoform 2 of ER membrane protein complex subunit 10	EMC10	0.165	0.248	-0.105
P53999	Activated RNA polymerase II transcriptional coactivator p15	SUB1	-0.065	0.195	-0.105
Q6ZSZ5-2	Isoform 2 of Rho guanine nucleotide exchange factor 18	ARHGEF18	0.029	0.178	-0.105
Q5VZB4	SLIT-ROBO Rho GTPase activating protein 2 (Fragment)	SRGAP2		-0.267	-0.105

O14907	Tax1-binding protein 3	TAX1BP3			-0.106
Q92878	DNA repair protein RAD50	RAD50	0.073	0.078	-0.106
O75394	39S ribosomal protein L33, mitochondrial	MRPL33	0.024		-0.106
H3BQ52	cAMP-regulated phosphoprotein 19	ARPP19	-0.047	-0.086	-0.106
Q6Y7W6-4	Isoform 3 of PERQ amino acid-rich with GYF domain-containing protein 2	GIGYF2	0.038	0.049	-0.106
Q8N163	DBIRD complex subunit KIAA1967	KIAA1967	-0.074	-0.097	-0.106
E5RGZ1	Probable tRNA threonylcarbamoyladenosine biosynthesis proteinGEPL1 (Fragment	OSGEPL1	-0.166		-0.106
Q6YHK3	CD109 antigen	CD109	-0.096	0.071	-0.106
P08107	Heat shock 70 kDa protein 1A/1B	HSPA1A	-0.103	-0.022	-0.107
Q92947	Glutaryl-CoA dehydrogenase, mitochondrial	GCDH	-0.139	-0.122	-0.107
Q02978	Mitochondrial 2-oxoglutarate/malate carrier protein	SLC25A11	0.000	0.047	-0.107
O60888-3	Isoform C of Protein CutA	CUTA	0.039	0.073	-0.107
Q6RW13	Type-1 angiotensin II receptor-associated protein	AGTRAP	-0.065	0.059	-0.107
Q08170	Serine/arginine-rich splicing factor 4	SRSF4	-0.036	-0.150	-0.107
Q9UKZ1	CCR4-NOT transcription complex subunit 11	CNOT11	-0.306	-0.243	-0.108
Q8N0U8	Vitamin K epoxide reductase complex subunit 1-like protein 1	VKORC1L1	-0.081	-0.074	-0.108
Q9NRR4-3	Isoform 3 of Ribonuclease 3	DROSHA			-0.108
P48730-2	Isoform 2 of Casein kinase I isoform delta	CSNK1D	-0.046	0.199	-0.108
Q9Y6D6	Brefeldin A-inhibited guanine nucleotide-exchange protein 1	ARFGEF1	-0.001	0.035	-0.108
Q03701	CCAAT/enhancer-binding protein zeta	CEBPZ	0.101	-0.034	-0.108
P30419	Glycylpeptide N-tetradecanoyltransferase 1	NMT1	0.039	-0.025	-0.108
Q53GS9	U4/U6.U5 tri-snRNP-associated protein 2	USP39	0.097	-0.083	-0.108
B0V043	Valine--tRNA ligase	VARS	-0.090	0.025	-0.109
Q9BYG3	MKI67 FHA domain-interacting nucleolar phosphoprotein	MKI67IP	0.140	0.095	-0.109
P11441	Ubiquitin-like protein 4A	UBL4A	0.111	0.338	-0.109
P21912	Succinate dehydrogenase [ubiquinone] iron-sulfur subunit, mitochondrial	SDHB	0.031	0.172	-0.109
Q9NZU0	Leucine-rich repeat transmembrane protein FLRT3	FLRT3			-0.109
P46109	Crk-like protein	CRKL	-0.056	-0.157	-0.109
P47985	Cytochrome b-c1 complex subunit Rieske, mitochondrial	UQCRFS1	-0.019	-0.033	-0.110
G3V529	ATP-dependent RNA helicase DDX24	DDX24	-0.115	-0.015	-0.110
Q05209	Tyrosine-protein phosphatase non-receptor type 12	PTPN12	-0.022	-0.159	-0.110
E9PPY3	Ribosomal RNA-processing protein 8	RRP8			-0.110
P21266	Glutathione S-transferase Mu 3	GSTM3	-0.120	0.060	-0.111
P49207	60S ribosomal protein L34	RPL34	-0.136	-0.075	-0.112
P14854	Cytochrome c oxidase subunit 6B1	COX6B1	-0.066	-0.258	-0.112
Q9BTE3-2	Isoform 2 of Mini-chromosome maintenance complex-binding protein	MCMBP	0.006	-0.120	-0.112
P31937	3-hydroxyisobutyrate dehydrogenase, mitochondrial	HIBADH	0.147	0.125	-0.112
B4DMQ3	Nuclear pore complex protein Nup85	NUP85	0.070	-0.128	-0.112
O43823	A-kinase anchor protein 8	AKAP8	-0.178	-0.042	-0.113
B0UX83	HLA-B associated transcript 3, isoform CRA_a	BAG6	-0.044	-0.009	-0.113
Q9Y5Y0	Feline leukemia virus subgroup C receptor-related protein 1	FLVCR1	0.180		-0.113
P17568	NADH dehydrogenase [ubiquinone] 1 beta subcomplex subunit 7	NDUFB7	0.006	0.134	-0.113
Q8TAT6	Nuclear protein localization protein 4 homolog	NPLOC4	-0.445	-0.265	-0.113
Q15029-2	Isoform 2 of 116 kDa U5 small nuclear ribonucleoprotein component	EFTUD2	0.002	-0.126	-0.113
O14737	Programmed cell death protein 5	PDCD5	-0.175	-0.145	-0.113
Q9Y4Z0	U6 snRNA-associated Sm-like protein LSm4	LSM4	0.002	0.053	-0.114
Q92882	Osteoclast-stimulating factor 1	OSTF1	0.104	0.052	-0.114
P62879	Guanine nucleotide-binding protein G(I)/G(S)/G(T) subunit beta-2	GNB2	0.070	0.011	-0.114
O75818-2	Isoform 2 of Ribonuclease P protein subunit p40	RPP40	-0.002	0.042	-0.114
P03905	NADH-ubiquinone oxidoreductase chain 4	MT-ND4		-0.272	-0.115
P62304	Small nuclear ribonucleoprotein E	SNRPE	0.040	-0.183	-0.115
Q6UXV4	Apolipoprotein O-like	APOOL		-0.123	-0.115
O14949	Cytochrome b-c1 complex subunit 8	UQCRQ	0.093	0.166	-0.115
P00403	Cytochrome c oxidase subunit 2	MT-CO2	0.008	-0.005	-0.116
P49247	Ribose-5-phosphate isomerase	RPIA	-0.085		-0.116
G8JLF3	Rab-like protein 6	RABL6	0.726		-0.116
P24752	Acetyl-CoA acetyltransferase, mitochondrial	ACAT1	-0.063	0.058	-0.116
Q7Z7L1	Schlafen family member 11	SLFN11		-0.144	-0.116
Q9NXF1-2	Isoform 2 of Testis-expressed sequence 10 protein	TEX10	0.001	0.065	-0.116
E9PDU5	WD repeat-containing protein 6	WDR6	-0.245	-0.085	-0.117
Q6PKG0	La-related protein 1	LARP1	-0.084	0.042	-0.117
P26368-2	Isoform 2 of Splicing factor U2AF 65 kDa subunit	U2AF2	-0.062	-0.045	-0.117
Q15417	Calponin-3	CNN3	-0.059	0.047	-0.117
Q9H0R4-2	Isoform 2 of Haloacid dehalogenase-like hydrolase domain-containing protein 2	HDHD2		0.051	-0.117
P23921	Ribonucleoside-diphosphate reductase large subunit	RRM1	0.045	-0.117	-0.117
K7EPV0	Cytochrome c oxidase assembly protein 3 homolog, mitochondrial	COA3		0.167	-0.117
Q9Y6N5	Sulfide:quinone oxidoreductase, mitochondrial	SQRDL	-0.065		-0.118
F5H5X1	Splicing factor, suppressor of white-apricot homolog (Fragment)	SFSWAP	-0.335	-0.104	-0.118
Q86W92-4	Isoform 4 of Liprin-beta-1	PPFIBP1	-0.205		-0.118
O95478	Ribosome biogenesis protein NSA2 homolog	NSA2	0.117	-0.129	-0.118
E9PJK1	CD81 antigen	CD81	-0.004	-0.079	-0.119
P57764	Gasdermin-D	GSDMD	-0.026	0.059	-0.119
Q6NYC8-2	Isoform 2 of Phostensin	PPP1R18	0.210		-0.119
P57737-3	Isoform 3 of Coronin-7	CORO7	-0.072	0.021	-0.119
P30043	Flavin reductase (NADPH)	BLVRB	-0.054	-0.055	-0.119
Q96HS1-2	Isoform 2 of Serine/threonine-protein phosphatase PGAM5, mitochondrial	PGAM5	-0.012	-0.007	-0.120
Q15428	Splicing factor 3A subunit 2	SF3A2	0.011	-0.064	-0.120
Q9H4M3	F-box only protein 44	FBXO44			-0.121
E9PJN1	Ribosomal protein S6 kinase	RPS6KA4	0.129		-0.121
Q02809	Procollagen-lysine,2-oxoglutarate 5-dioxygenase 1	PLOD1	-0.068	-0.040	-0.121
O00165-5	Isoform 5 of HCLS1-associated protein X-1	HAX1	-0.011	0.056	-0.121
Q7Z434	Mitochondrial antiviral-signaling protein	MAVS	-0.050	0.030	-0.121
P48436	Transcription factor SOX-9	SOX9	0.035	0.050	-0.121
K7EMM8	Putative oxidoreductase GLYR1 (Fragment)	GLYR1	-0.202	0.078	-0.121
A6NJZ9	Nucleolar complex protein 3 homolog	NOC3L	-0.086	0.023	-0.122
Q9BVK2-2	Isoform 2 of Probable dolichyl pyrophosphate Glc1Man9GlcNAc2 alpha-1,3-glucosy	ALG8	0.008		-0.122
Q9Y6Y0	Influenza virus NS1A-binding protein	IVNS1ABP		0.186	-0.122

Q5RHS7	Protein S100-A2	S100A2			-0.122
Q96ST2-2	Isoform 2 of Protein IWS1 homolog	IWS1	0.050		-0.122
Q9H2V7-3	Isoform 3 of Protein spinster homolog 1	SPNS1	-0.105	0.112	-0.122
Q96CN7	Isochorismatase domain-containing protein 1	ISOC1			-0.122
Q01780-2	Isoform 2 of Exosome component 10	EXOSC10	0.019	-0.050	-0.122
B3KMK8	Starch-binding domain-containing protein 1	STBD1	0.501		-0.122
Q96PK6	RNA-binding protein 14	RBM14	-0.016	-0.016	-0.122
O75531	Barrier-to-autointegration factor	BANF1	-0.305	-0.275	-0.123
B4DUX5	Methionine aminopeptidase	METAP2	0.074	0.052	-0.123
Q9BYT8	Neurolysin, mitochondrial	NLN	-0.010	-0.014	-0.123
Q02818	Nucleobindin-1	NUCB1	0.049	0.013	-0.123
H3BS09	Ubiquitin carboxyl-terminal hydrolase CYLD	CYLD			-0.124
O75815-3	Isoform 3 of Breast cancer anti-estrogen resistance protein 3	BCAR3	-0.068		-0.124
K7EIJ0	WW domain-binding protein 2 (Fragment)	WBP2	0.448	0.082	-0.124
P06702	Protein S100-A9	S100A9	0.398		-0.125
Q9H2U1	Probable ATP-dependent RNA helicase DHX36	DHX36	-0.053	-0.086	-0.125
P42226-3	Isoform 3 of Signal transducer and activator of transcription 6	STAT6	-0.120		-0.125
Q8TC07-2	Isoform 2 of TBC1 domain family member 15	TBC1D15	0.072	0.155	-0.125
P31930	Cytochrome b-c1 complex subunit 1, mitochondrial	UQCRC1	0.020	0.015	-0.125
Q9Y520-6	Isoform 6 of Protein PRRC2C	PRRC2C	-0.009	0.057	-0.125
Q7KZ85	Transcription elongation factor SPT6	SUPT6H	0.147	0.010	-0.125
Q8NE71	ATP-binding cassette sub-family F member 1	ABCF1	-0.087	-0.101	-0.126
Q9NZL9	Methionine adenosyltransferase 2 subunit beta	MAT2B	0.097	0.027	-0.126
Q5TCU3	Tropomyosin beta chain	TPM2			-0.127
Q8WUA2	Peptidyl-prolyl cis-trans isomerase-like 4	PPIL4	0.074	-0.022	-0.127
Q13616	Cullin-1	CUL1	-0.001	0.078	-0.127
E7ETZ4	Basic leucine zipper and W2 domain-containing protein 2 (Fragment)	BZW2	-0.200	-0.276	-0.127
G5E9D5	ElaC homolog 2 (E. coli), isoform CRA_a	ELAC2	0.031	0.028	-0.127
Q13541	Eukaryotic translation initiation factor 4E-binding protein 1	EIF4EBP1		-0.072	-0.127
H7BXS3	Phosphatidylinositol 5-phosphate 4-kinase type-2 alpha	PIP4K2A	-0.053	0.217	-0.127
F8WD00	U3 small nucleolar RNA-associated protein 14 homolog A	UTP14A	0.057	-0.042	-0.128
E7EMB1	Switch-associated protein 70	SWAP70	0.096	-0.008	-0.128
Q8IZL8	Proline-, glutamic acid- and leucine-rich protein 1	PELP1	0.066	-0.069	-0.129
P18510-4	Isoform 4 of Interleukin-1 receptor antagonist protein	IL1RN			-0.129
Q6YN16	Hydroxysteroid dehydrogenase-like protein 2	HSDL2	0.000	-0.028	-0.129
Q14191	Werner syndrome ATP-dependent helicase	WRN			-0.129
Q8N2K0	Monoacylglycerol lipase ABHD12	ABHD12	0.134	0.066	-0.130
H0YLA4	Sorbitol dehydrogenase	SORD	-0.009	0.077	-0.130
Q9NPJ3-2	Isoform 2 of Acyl-coenzyme A thioesterase 13	ACOT13	0.058		-0.130
P16401	Histone H1.5	HIST1H1B	-0.372	-0.082	-0.130
C9JGR5	Rab GTPase-activating protein 1 (Fragment)	RABGAP1	-0.236	-0.085	-0.130
Q7Z2W4	Zinc finger CCCH-type antiviral protein 1	ZC3HAV1	0.211	0.053	-0.131
A2IDC7	39S ribosomal protein L30, mitochondrial (Fragment)	MRPL28	0.138	-0.014	-0.131
P19971	Thymidine phosphorylase	TYMP		0.247	-0.131
Q9UNI6	Dual specificity protein phosphatase 12	DUSP12	-0.040		-0.132
F8WJN3	Cleavage and polyadenylation-specificity factor subunit 6	CPSF6	-0.022	0.053	-0.132
Q09161	Nuclear cap-binding protein subunit 1	NCBP1	0.013	-0.090	-0.132
P08670	Vimentin	VIM	0.666	-0.791	-0.133
P18827	Syndecan-1	SDC1			-0.133
Q13177	Serine/threonine-protein kinase PAK 2	PAK2	-0.118	-0.114	-0.134
Q13363-2	Isoform 2 of C-terminal-binding protein 1	CTBP1	0.097	0.353	-0.134
O60244	Mediator of RNA polymerase II transcription subunit 14	MED14	0.020		-0.135
Q10570	Cleavage and polyadenylation specificity factor subunit 1	CPSF1	0.091	-0.016	-0.135
H3BQQ9	SUMO-conjugating enzyme UBC9 (Fragment)	UBE2I	-0.065	-0.107	-0.135
Q9BZE1	39S ribosomal protein L37, mitochondrial	MRPL37	-0.011	0.167	-0.135
P18859	ATP synthase-coupling factor 6, mitochondrial	ATP5J	0.160	0.048	-0.136
F5H1W8	Mitochondrial enolase superfamily member 1	ENOSF1	-0.024	0.294	-0.136
Q9UBP6	tRNA (guanine-N(7)-)-methyltransferase	METTL1	0.022	0.257	-0.136
Q9NPL8	Translocase of inner mitochondrial membrane domain-containing protein 1	TIMMDC1	-0.348	-0.268	-0.136
P10316	HLA class I histocompatibility antigen, A-69 alpha chain	HLA-A		-1.336	-0.136
P08397-2	Isoform 2 of Porphobilinogen deaminase	HMBS	-0.050	0.128	-0.136
B7Z588	Tumor necrosis factor receptor superfamily member 10B	TNFRSF10B			-0.136
Q9H8H0	Nucleolar protein 11	NOL11		-0.308	-0.136
O75380	NADH dehydrogenase [ubiquinone] iron-sulfur protein 6, mitochondrial	NDUFS6	-0.167	-0.197	-0.136
Q9Y4A5-2	Isoform 2 of Transformation/transcription domain-associated protein	TRRAP	0.002	0.128	-0.137
P24941-2	Isoform 2 of Cyclin-dependent kinase 2	CDK2	0.281	0.003	-0.137
P49459	Ubiquitin-conjugating enzyme E2 A	UBE2A	-0.106	0.130	-0.137
Q13155	Aminoacyl tRNA synthase complex-interacting multifunctional protein 2	AIMP2	-0.094	0.055	-0.137
Q6P1N0-2	Isoform 2 of Coiled-coil and C2 domain-containing protein 1A	CC2D1A	0.153	0.131	-0.138
Q13630	GDP-L-fucose synthase	TSTA3	-0.086	-0.039	-0.138
Q15637-4	Isoform 4 of Splicing factor 1	SF1	-0.045	0.017	-0.138
O43768-2	Isoform 2 of Alpha-endosulfine	ENSA	-0.041	-0.026	-0.138
Q8NBF2	NHL repeat-containing protein 2	NHLRC2	-0.058	-0.116	-0.138
P61604	10 kDa heat shock protein, mitochondrial	HSPE1	-0.104	-0.246	-0.138
Q9NX40	OCIA domain-containing protein 1	OCIAD1	0.046	-0.040	-0.139
Q9BZZ5-2	Isoform 2 of Apoptosis inhibitor 5	API5	0.117	-0.003	-0.139
Q9NRG0	Chromatin accessibility complex protein 1	CHRAC1	0.102	0.058	-0.139
P07741	Adenine phosphoribosyltransferase	APRT	-0.046	-0.081	-0.139
O43929	Origin recognition complex subunit 4	ORC4		0.058	-0.139
Q8NBJ5	Procollagen galactosyltransferase 1	COLGALT1	0.122	0.018	-0.140
O15078-2	Isoform 2 of Centrosomal protein of 290 kDa	CEP290		-0.061	-0.140
Q9P2B2	Prostaglandin F2 receptor negative regulator	PTGFRN	-0.008		-0.140
P43897	Elongation factor Ts, mitochondrial	TSFM	0.290	0.176	-0.140
F8VSC5	SCY1-like protein 2 (Fragment)	SCYL2	-0.001		-0.140
B4DUF5	Acyl-CoA synthetase family member 2, mitochondrial	ACSF2	0.033		-0.140
G5E972	Thymopentin	TMPO			-0.141
Q96BR5	Sel1 repeat-containing protein 1	SELRC1	0.147	0.326	-0.141

Q9P0J1	[Pyruvate dehydrogenase [acetyl-transferring]]-phosphatase 1, mitochondrial	PDP1	0.095	-0.054	-0.142
Q99720	Sigma non-opioid intracellular receptor 1	SIGMAR1	-0.021	0.255	-0.142
F8WAH1	Lysine ketoglutarate reductase	AASS			-0.142
O76031	ATP-dependent Clp protease ATP-binding subunit clpX-like, mitochondrial	CLPX	-0.004	-0.050	-0.142
Q9UL46	Proteasome activator complex subunit 2	PSME2	0.111	0.149	-0.142
Q9HA77	Probable cysteine--tRNA ligase, mitochondrial	CARS2		0.056	-0.142
Q9UIA9	Exportin-7	XPO7	0.027	0.056	-0.142
O94925	Glutaminase kidney isoform, mitochondrial	GLS		0.114	-0.142
Q9UHD9	Ubiquilin-2	UBQLN2	0.113	-0.042	-0.142
Q96T37-2	Isoform 2 of Putative RNA-binding protein 15	RBM15	0.084	-0.020	-0.143
Q9C0B1	Alpha-ketoglutarate-dependent dioxygenase FTO	FTO	-0.041	-0.023	-0.143
Q9H9B4	Sideroflexin-1	SFXN1	0.012	0.090	-0.143
Q9NVI7-2	Isoform 2 of ATPase family AAA domain-containing protein 3A	ATAD3A	-0.039	-0.076	-0.143
Q6KC79-2	Isoform 2 of Nipped-B-like protein	NIPBL	0.080	-0.127	-0.143
Q13123	Protein Red	IK	0.072	0.072	-0.143
Q96FQ6	Protein S100-A16	S100A16	-0.091		-0.143
P06400	Retinoblastoma-associated protein	RB1			-0.144
Q9H0S4	Probable ATP-dependent RNA helicase DDX47	DDX47	0.152	0.165	-0.144
O75438	NADH dehydrogenase [ubiquinone] 1 beta subcomplex subunit 1	NDUFB1	0.002	-0.097	-0.145
Q7Z4Q2	HEAT repeat-containing protein 3	HEATR3	-0.004	-0.109	-0.146
Q99627-2	Isoform 2 of COP9 signalosome complex subunit 8	COPS8	-0.047	-0.233	-0.146
Q8NFW8	N-acylneuraminate cytidylyltransferase	CMAS	0.280	-0.224	-0.147
Q96HR8-2	Isoform 2 of H/ACA ribonucleoprotein complex non-core subunit NAF1	NAF1	0.016	-0.032	-0.147
Q9H299	SH3 domain-binding glutamic acid-rich-like protein 3	SH3BGRL3	0.066	-0.199	-0.148
Q13191-3	Isoform Truncated 2 of E3 ubiquitin-protein ligase CBL-B	CBLB	-0.221	0.137	-0.148
F5H7E2	Superkiller viralicidic activity 2-like 2	SKIV2L2	-0.068	-0.055	-0.148
Q969Z0	Protein TBRG4	TBRG4	-0.293	-0.131	-0.149
O95336	6-phosphogluconolactonase	PGLS	0.054	0.089	-0.149
Q9UBB6-2	Isoform 2 of Neurochondrin	NCDN	-0.101	-0.029	-0.150
Q9BYX4	Interferon-induced helicase C domain-containing protein 1	IFIH1			-0.150
E9PAU2	Ribonucleoprotein PTB-binding 1	RAVER1	-0.028	-0.026	-0.151
Q9H444	Charged multivesicular body protein 4b	CHMP4B	0.060	0.059	-0.151
Q7Z5K2	Wings apart-like protein homolog	WAPAL	-0.119	0.088	-0.151
C9JVN9	L-2-hydroxyglutarate dehydrogenase, mitochondrial	L2HGDH	-0.010		-0.151
Q96C90	Protein phosphatase 1 regulatory subunit 14B	PPP1R14B	-0.069	-0.025	-0.152
Q99988	Growth/differentiation factor 15	GDF15			-0.152
Q9Y399	28S ribosomal protein S2, mitochondrial	MRPS2	-0.089	-0.005	-0.152
H3BS02	[3-methyl-2-oxobutanoate dehydrogenase [lipoamide]] kinase, mitochondrial	BCKDK	-0.147		-0.152
Q8NEZ5	F-box only protein 22	FBXO22	0.079		-0.153
Q16637-3	Isoform SMN-delta7 of Survival motor neuron protein	SMN1			-0.153
Q9BY77-2	Isoform 2 of Polymerase delta-interacting protein 3	POLDIP3	0.103	0.008	-0.153
J3KMY5	Epididymal secretory protein E1	NPC2	-0.247	0.157	-0.153
Q06203	Amidophosphoribosyltransferase	PPAT	-0.070	0.076	-0.153
O96013	Serine/threonine-protein kinase PAK 4	PAK4	0.025	0.092	-0.154
A8DPD7	Protein zyg-11 homolog B	ZYG11B	-0.096	-0.121	-0.154
Q9UKK9	ADP-sugar pyrophosphatase	NUDT5	-0.100	-0.121	-0.154
Q8WW12	PEST proteolytic signal-containing nuclear protein	PCNP	0.047	-0.019	-0.154
Q15654	Thyroid receptor-interacting protein 6	TRIP6	0.200	0.097	-0.154
Q96GX9	Methylthioribulose-1-phosphate dehydratase	APIP	-0.097	-0.084	-0.154
F5GX82	Protein furry homolog-like	FRYL			-0.154
P49720	Proteasome subunit beta type-3	PSMB3	-0.003	-0.035	-0.155
Q14696	LDLR chaperone MESD	MESDC2	-0.072	-0.197	-0.155
Q6PJG6	BRCA1-associated ATM activator 1	BRAT1	-0.071	-0.135	-0.155
P34896-2	Isoform 2 of Serine hydroxymethyltransferase, cytosolic	SHMT1	0.070	0.281	-0.156
Q05932-3	Isoform 3 of Folylpolyglutamate synthase, mitochondrial	FPGS	0.149		-0.156
O95777	N-alpha-acetyltransferase 38, NatC auxiliary subunit	NAA38	0.073	-0.116	-0.156
P35237	Serpin B6	SERPINB6	0.062	0.304	-0.156
Q7L9L4	MOB kinase activator 1B	MOB1B	-0.054	-0.007	-0.157
B5MDQ4	Cell differentiation protein RCD1 homolog	RQCD1	-0.164	-0.035	-0.158
Q86U42-2	Isoform 2 of Polyadenylate-binding protein 2	PABPN1	0.052	-0.146	-0.158
Q03468	DNA excision repair protein ERCC-6	ERCC6	-0.063		-0.158
P49458	Signal recognition particle 9 kDa protein	SRP9	-0.118	-0.230	-0.159
O43464-2	Isoform 2 of Serine protease HTRA2, mitochondrial	HTRA2	0.185		-0.159
Q9H1E1	Ribonuclease 7	RNASE7			-0.160
Q9NUL7	Probable ATP-dependent RNA helicase DDX28	DDX28	0.040		-0.160
Q14108	Lysosome membrane protein 2	SCARB2	-0.173	0.136	-0.160
P57076	Uncharacterized protein C21orf59	C21orf59	-0.166	-0.102	-0.161
Q16774	Guanylate kinase	GUK1		0.268	-0.162
Q9Y6G9	Cytoplasmic dynein 1 light intermediate chain 1	DYNC1LI1	0.122	-0.051	-0.162
P00390-5	Isoform 4 of Glutathione reductase, mitochondrial	GSR	-0.416	0.003	-0.163
Q13404-1	Isoform 1 of Ubiquitin-conjugating enzyme E2 variant 1	UBE2V1	-0.014	-0.015	-0.163
B7Z806	Glycogen [starch] synthase, muscle	GYS1	0.064	0.323	-0.163
Q86TU7	Histone-lysine N-methyltransferase setd3	SETD3	-0.034	0.020	-0.163
Q8IYM9-2	Isoform 2 of E3 ubiquitin-protein ligase TRIM22	TRIM22			-0.164
Q8NFF5-3	Isoform 3 of FAD synthase	FLAD1	0.022	-0.090	-0.164
Q9H0U6	39S ribosomal protein L18, mitochondrial	MRPL18		-0.012	-0.164
O15031	Plexin-B2	PLXNB2	-0.004		-0.164
G3V325	Pentatricopeptide repeat-containing protein 1, mitochondrial	ATP5J2-PTCD1	0.013	0.161	-0.165
C9JQV0	Uncharacterized protein C7orf50 (Fragment)	C7orf50	-0.043	0.215	-0.165
P20700	Lamin-B1	LMNB1	0.001	-0.170	-0.165
Q6P179-3	Isoform 3 of Endoplasmic reticulum aminopeptidase 2	ERAP2	0.428		-0.165
Q9UEW8	STE20/SPS1-related proline-alanine-rich protein kinase	STK39	-0.293		-0.166
F5H855	Breast cancer anti-estrogen resistance protein 1	BCAR1	-0.079	-0.030	-0.166
B4DQJ8	6-phosphogluconate dehydrogenase, decarboxylating	PGD	0.056	0.011	-0.166
P37198	Nuclear pore glycoprotein p62	NUP62	0.136	-0.008	-0.166
Q9Y2Z4	Tyrosine--tRNA ligase, mitochondrial	YARS2	-0.089		-0.167
P42345	Serine/threonine-protein kinase mTOR	MTOR	0.079	0.024	-0.167

Q04760-2	Isoform 2 of Lactoylglutathione lyase	GLO1	0.278	-0.005	-0.167
Q8NDI1-3	Isoform 3 of EH domain-binding protein 1	EHBP1		-0.122	-0.167
Q9P2E3	NFX1-type zinc finger-containing protein 1	ZNFX1	-0.068		-0.168
Q96JB5	CDK5 regulatory subunit-associated protein 3	CDK5RAP3	0.099	0.210	-0.168
Q9BQG0	Myb-binding protein 1A	MYBBP1A	-0.197	-0.106	-0.168
Q9Y2B0	Protein canopy homolog 2	CNPY2	0.153	-0.001	-0.169
Q8N4H5	Mitochondrial import receptor subunit TOM5 homolog	TOMM5			-0.169
Q9BRP8-2	Isoform 2 of Partner of Y14 and mago	WIBG	0.063	0.140	-0.170
Q7Z2W9-2	Isoform 2 of 39S ribosomal protein L21, mitochondrial	MRPL21			-0.170
P49591	Serine--tRNA ligase, cytoplasmic	SARS	0.022	-0.010	-0.170
E9PS44	Interferon-induced transmembrane protein 3	IFITM3	0.378	0.219	-0.171
Q5VZE5	N-alpha-acetyltransferase 35, NatC auxiliary subunit	NAA35	-0.075		-0.172
J3KT51	Hematological and neurological-expressed 1 protein	HN1	0.085	-0.012	-0.172
O14776-2	Isoform 2 of Transcription elongation regulator 1	TCERG1	0.117	0.001	-0.172
P78347-2	Isoform 2 of General transcription factor II-I	GTF2I	-0.007	0.053	-0.172
Q99615	DnaJ homolog subfamily C member 7	DNAJC7	-0.142	-0.098	-0.172
O95810	Serum deprivation-response protein	SDPR			-0.173
P48637	Glutathione synthetase	GSS	0.059	0.140	-0.173
Q9Y6B6	GTP-binding protein SAR1b	SAR1B		0.234	-0.173
F5H721	WW domain-binding protein 11	WBP11	0.062	0.017	-0.173
Q14166	Tubulin--tyrosine ligase-like protein 12	TTLL12	-0.122	0.035	-0.173
Q9UK73	Protein fem-1 homolog B	FEM1B			-0.173
C9J0A7	Charged multivesicular body protein 2b	CHMP2B	-0.136		-0.174
Q9NWV4	UPF0587 protein C1orf123	C1orf123	0.064	0.018	-0.174
K7EKW4	Isochorismatase domain-containing protein 2, mitochondrial (Fragment)	ISOC2	-0.130	0.084	-0.174
B8ZZU8	Transcription elongation factor B (SIII), polypeptide 2 (18kDa, elongin B), isoform	TCEB2	0.194	-0.108	-0.174
Q5JRA6-2	Isoform 2 of Melanoma inhibitory activity protein 3	MIA3	0.009	-0.033	-0.175
P27707	Deoxycytidine kinase	DCK	0.135	-0.108	-0.176
P42224	Signal transducer and activator of transcription 1-alpha/beta	STAT1	0.116	0.147	-0.176
O95571	Protein ETHE1, mitochondrial	ETHE1	0.167		-0.176
Q96DV4	39S ribosomal protein L38, mitochondrial	MRPL38	0.014	-0.025	-0.176
Q08752	Peptidyl-prolyl cis-trans isomerase D	PPID	-0.164	-0.012	-0.177
P60602	Reactive oxygen species modulator 1	ROMO1			-0.177
Q9HD33-2	Isoform 2 of 39S ribosomal protein L47, mitochondrial	MRPL47	-0.053	0.038	-0.177
Q6P1J9	Parafibromin	CDC73	0.014	0.056	-0.178
Q13310	Polyadenylate-binding protein 4	PABPC4			-0.178
Q92974-3	Isoform 3 of Rho guanine nucleotide exchange factor 2	ARHGEF2	0.173	0.024	-0.178
O95602	DNA-directed RNA polymerase I subunit RPA1	POLR1A	0.149	-0.090	-0.178
Q9BTV4	Transmembrane protein 43	TMEM43	0.097	0.061	-0.178
B4E2J1	ATPase family AAA domain-containing protein 1	ATAD1		0.109	-0.179
Q9HCY8	Protein S100-A14	S100A14	-0.210		-0.179
O75223	Gamma-glutamylcyclotransferase	GGCT	-0.033	-0.073	-0.179
P14406	Cytochrome c oxidase subunit 7A2, mitochondrial	COX7A2			-0.179
P61026	Ras-related protein Rab-10	RAB10	0.138	0.015	-0.180
Q9UJZ1	Stomatin-like protein 2, mitochondrial	STOML2	0.024	0.034	-0.180
Q15648	Mediator of RNA polymerase II transcription subunit 1	MED1	-0.012	0.061	-0.180
P06280	Alpha-galactosidase A	GLA	0.107	0.228	-0.180
O75051	Plexin-A2	PLXNA2			-0.180
Q9Y6D5	Brefeldin A-inhibited guanine nucleotide-exchange protein 2	ARFGEF2	0.337		-0.181
O43237	Cytoplasmic dynein 1 light intermediate chain 2	DYNC1LI2	-0.033	-0.042	-0.181
Q8NI36	WD repeat-containing protein 36	WDR36		-0.103	-0.182
Q8N5K1	CDGSH iron-sulfur domain-containing protein 2	CISD2	-0.050	0.061	-0.182
H7C0M4	ALS2 C-terminal-like protein (Fragment)	ALS2CL			-0.182
Q9NX63	Coiled-coil-helix-coiled-coil-helix domain-containing protein 3, mitochondrial	CHCHD3	0.024	-0.030	-0.183
Q9HAV4	Exportin-5	XPO5	-0.070	0.006	-0.183
Q9UEE5	Serine/threonine-protein kinase 17A	STK17A			-0.183
Q9Y305	Acyl-coenzyme A thioesterase 9, mitochondrial	ACOT9	0.124		-0.183
F5H0N1	Anaphase-promoting complex subunit 5	ANAPC5		0.176	-0.183
Q5TDH0-2	Isoform 2 of Protein DDI1 homolog 2	DDI2			-0.183
Q9NVS2	28S ribosomal protein S18a, mitochondrial	MRPS18A	0.127		-0.183
Q9NVU7	Protein SDA1 homolog	SDAD1	-0.124	-0.014	-0.183
Q7L592-2	Isoform 2 of NADH dehydrogenase [ubiquinone] complex I, assembly factor 7	NDUFAF7	-0.242		-0.185
A8MTP9	Probable ATP-dependent RNA helicase DDX52	DDX52	0.088		-0.185
O75886	Signal transducing adapter molecule 2	STAM2			-0.185
Q9UPN9	E3 ubiquitin-protein ligase TRIM33	TRIM33	0.043	-0.155	-0.185
C9J5G4	Follistatin-related protein 1 (Fragment)	FSTL1	-0.228		-0.186
Q14137	Ribosome biogenesis protein BOP1	BOP1	-0.127	-0.198	-0.186
Q9H7E9	UPF0488 protein C8orf33	C8orf33	-0.059	0.006	-0.186
P40222	Alpha-taxilin	TXLNA	-0.017	-0.029	-0.187
Q8NBN7	Retinol dehydrogenase 13	RDH13	-0.067	0.053	-0.187
H3BLU7	Aflatoxin B1 aldehyde reductase member 2 (Fragment)	AKR7A2	0.154	-0.040	-0.188
P35250-2	Isoform 2 of Replication factor C subunit 2	RFC2	0.048	0.037	-0.189
O60493	Sorting nexin-3	SNX3	-0.034	-0.059	-0.189
Q9P0I2	ER membrane protein complex subunit 3	EMC3	0.096	0.113	-0.190
O94874	E3 UFM1-protein ligase 1	UFL1	0.081	0.179	-0.190
Q5JTH9	RRP12-like protein	RRP12	-0.081	-0.211	-0.190
Q9H6S3	Epidermal growth factor receptor kinase substrate 8-like protein 2	EPS8L2	0.222	0.109	-0.191
B7ZAB3	Galactosylgalactosylxylosylprotein 3-beta-glucuronosyltransferase 3	B3GAT3		0.180	-0.191
H7C1G1	Transmembrane protein 87B (Fragment)	TMEM87B	0.178		-0.191
O15296-2	Isoform B of Arachidonate 15-lipoxygenase B	ALOX15B			-0.192
Q7L8L6	FAST kinase domain-containing protein 5	FASTKD5			-0.193
B4E3S0	Coronin	CORO1C	-0.164	0.323	-0.193
B3KT28	FAS-associated factor 1	FAF1	-0.012	-0.128	-0.194
C9J0J7	Profilin-2	PFN2	0.488	-0.367	-0.194
Q92575	UBX domain-containing protein 4	UBXN4	-0.025	-0.107	-0.194
P29508	Serpin B3	SERPINB3			-0.195
P61964	WD repeat-containing protein 5	WDR5	0.001	-0.173	-0.195

Q92614-4	Isoform 4 of Unconventional myosin-XVIIIa	MYO18A	0.049	0.036	-0.196
Q6P9B9	Integrator complex subunit 5	INTS5	-0.087	-0.072	-0.196
H0Y9E6	Centromere protein H (Fragment)	CENPH			-0.196
Q7Z5L9-2	Isoform 2 of Interferon regulatory factor 2-binding protein 2	IRF2BP2	-0.011	0.179	-0.197
P49189	4-trimethylaminobutyraldehyde dehydrogenase	ALDH9A1	0.122	-0.027	-0.197
O75165	DnaJ homolog subfamily C member 13	DNAJC13	0.024		-0.199
Q9HD26-2	Isoform 2 of Golgi-associated PDZ and coiled-coil motif-containing protein	GOPC	0.187		-0.200
P28340	DNA polymerase delta catalytic subunit	POLD1	-0.082	-0.008	-0.200
P55809	Succinyl-CoA:3-ketoacid coenzyme A transferase 1, mitochondrial	OXCT1	-0.102	-0.013	-0.200
P63208	S-phase kinase-associated protein 1	SKP1	-0.069	0.052	-0.201
O43617	Trafficking protein particle complex subunit 3	TRAPPC3		0.184	-0.201
Q9UBV2	Protein sel-1 homolog 1	SEL1L	0.058	0.096	-0.201
Q8N4Q1	Mitochondrial intermembrane space import and assembly protein 40	CHCHD4	-0.050	-0.054	-0.202
P35270	Sepiapterin reductase	SPR	0.017	0.236	-0.202
Q92625	Ankyrin repeat and SAM domain-containing protein 1A	ANKS1A	-0.052		-0.202
Q13895	Bystin	BYSL	0.100	-0.103	-0.203
P26232-2	Isoform 2 of Catenin alpha-2	CTNNA2	0.112		-0.203
O00178	GTP-binding protein 1	GTPBP1	-0.036	0.187	-0.204
Q8IZ73	RNA pseudouridylate synthase domain-containing protein 2	RPUSD2	0.045	-0.131	-0.204
P54577	Tyrosine--tRNA ligase, cytoplasmic	YARS	-0.001	0.058	-0.204
Q15642-2	Isoform 2 of Cdc42-interacting protein 4	TRIP10	0.228	0.230	-0.205
Q7Z4H3-2	Isoform 2 of HD domain-containing protein 2	HDDC2	0.044		-0.205
Q9Y6M7-6	Isoform 6 of Sodium bicarbonate cotransporter 3	SLC4A7	0.220	0.059	-0.205
Q3MHD2	Protein LSM12 homolog	LSM12	-0.213	-0.086	-0.206
Q8N878	FERM domain-containing protein 1	FRMD1	0.052	-0.009	-0.206
Q9NRW7	Vacuolar protein sorting-associated protein 45	VPS45	0.045	0.016	-0.206
Q6PJW8-3	Isoform 3 of Consortin	CNST			-0.206
H7C492	Mitochondrial chaperone BCS1 (Fragment)	BCS1L			-0.206
Q14232	Translation initiation factor eIF-2B subunit alpha	EIF2B1	0.099	0.058	-0.207
P29353-5	Isoform 5 of SHC-transforming protein 1	SHC1	-0.258		-0.207
P09543-2	Isoform CNPI of 2',3'-cyclic-nucleotide 3'-phosphodiesterase	CNP	0.095	0.072	-0.207
Q8IWZ3	Ankyrin repeat and KH domain-containing protein 1	ANKHD1	0.182	0.127	-0.207
P10412	Histone H1.4	HIST1H1E	-0.118	0.056	-0.208
P62937	Peptidyl-prolyl cis-trans isomerase A	PPIA	0.008	-0.005	-0.208
H0YCN4	DCN1-like protein 5 (Fragment)	DCUN1D5	-0.106	-0.058	-0.209
Q16643	Drebrin	DBN1	-0.064	0.318	-0.209
P42574	Caspase-3	CASP3	-0.058	-0.075	-0.210
Q58FG1	Putative heat shock protein HSP 90-alpha A4	HSP90AA4P	0.534	-0.109	-0.210
K7ELV2	Nucleoporin SEH1 (Fragment)	SEH1L	0.019	-0.056	-0.211
P23381	Tryptophan--tRNA ligase, cytoplasmic	WARS	0.141	0.019	-0.211
F8W785	Golgi integral membrane protein 4	GOLIM4	-0.091	0.079	-0.212
Q8IXH7-4	Isoform NELF-D of Negative elongation factor C/D	NELFCD	0.073	0.028	-0.212
O43684-2	Isoform 2 of Mitotic checkpoint protein BUB3	BUB3	-0.085	-0.151	-0.212
O43913	Origin recognition complex subunit 5	ORC5	0.011	0.321	-0.212
P28062-2	Isoform 2 of Proteasome subunit beta type-8	PSMB8	0.304	0.216	-0.213
Q5VIR6	Vacuolar protein sorting-associated protein 53 homolog	VPS53	0.153	0.094	-0.214
P37108	Signal recognition particle 14 kDa protein	SRP14	-0.022	0.142	-0.214
Q96DG6	Carboxymethylenebutenolidase homolog	CMBL	-0.214		-0.215
P20340-4	Isoform 4 of Ras-related protein Rab-6A	RAB6A	0.133	0.080	-0.215
M0R2R2	Serine/threonine-protein kinase D2	PRKD2			-0.216
Q9H4L5-2	Isoform 1b of Oxysterol-binding protein-related protein 3	OSBPL3	0.054		-0.217
B4DT35	Nucleoporin p54	NUP54	0.032	-0.008	-0.217
O43670-2	Isoform 2 of Zinc finger protein 207	ZNF207	0.073	-0.057	-0.217
I3L3V5	Trafficking protein particle complex subunit 1 (Fragment)	TRAPPC1	-0.080	0.040	-0.217
P04899	Guanine nucleotide-binding protein G(i) subunit alpha-2	GNAI2	0.159	0.229	-0.218
Q16795	NADH dehydrogenase [ubiquinone] 1 alpha subcomplex subunit 9, mitochondrial	NDUFA9	-0.175	0.325	-0.219
Q9NP92	28S ribosomal protein S30, mitochondrial	MRPS30	0.026	0.022	-0.219
Q9HCD5	Nuclear receptor coactivator 5	NCOA5	0.018	0.082	-0.220
P31942-2	Isoform 2 of Heterogeneous nuclear ribonucleoprotein H3	HNRNPH3	-0.054	-0.604	-0.220
Q5JR08	Rho-related GTP-binding protein RhoC (Fragment)	RHOC	0.569	0.593	-0.221
B4DJJ3	Transmembrane anterior posterior transformation protein 1 homolog	TAPT1			-0.221
Q86X10-2	Isoform 2 of Ral GTPase-activating protein subunit beta	RALGAPB	0.085	0.059	-0.221
Q9BVC6	Transmembrane protein 109	TMEM109	0.047	0.109	-0.221
Q5VTL8	Pre-mRNA-splicing factor 38B	PRPF38B	-0.078	-0.054	-0.222
J3KNH7	Sentrin-specific protease 3	SENP3	-0.152	0.140	-0.222
F5H1S9	tRNA pseudouridine synthase	PUS1		0.019	-0.223
H0YA80	Ubiquitin-conjugating enzyme E2 B (Fragment)	UBE2B	0.137	-0.107	-0.223
Q99805	Transmembrane 9 superfamily member 2	TM9SF2	0.048	0.132	-0.224
O95831-3	Isoform 3 of Apoptosis-inducing factor 1, mitochondrial	AIFM1	0.073	0.137	-0.225
P21589	5'-nucleotidase	NT5E	0.014		-0.225
Q9NUQ2	1-acyl-sn-glycerol-3-phosphate acyltransferase epsilon	AGPAT5	-0.022		-0.225
Q96JJ7	Protein disulfide-isomerase TMX3	TMX3		-0.352	-0.225
P61769	Beta-2-microglobulin	B2M	0.449		-0.225
O60220	Mitochondrial import inner membrane translocase subunit Tim8 A	TIMM8A	0.032	-0.116	-0.226
Q9UDY2-3	Isoform C1 of Tight junction protein ZO-2	TJP2	0.158	0.077	-0.226
H0YEL9	Probable asparagine--tRNA ligase, mitochondrial (Fragment)	NARS2			-0.227
Q9P032	NADH dehydrogenase [ubiquinone] 1 alpha subcomplex assembly factor 4	NDUFAF4	-0.020	0.120	-0.227
Q5JRX3	Presequence protease, mitochondrial	PITRM1	0.051	0.082	-0.228
P81274	G-protein-signaling modulator 2	GPSM2			-0.228
Q9Y5V0	Zinc finger protein 706	ZNF706	-0.267	-0.260	-0.229
Q01970-2	Isoform 2 of 1-phosphatidylinositol 4,5-bisphosphate phosphodiesterase beta-3	PLCB3	0.138	-0.049	-0.229
Q16186	Proteasomal ubiquitin receptor ADRM1	ADRM1	-0.124	-0.102	-0.229
P14550	Alcohol dehydrogenase [NADP(+)]	AKR1A1	0.075	0.187	-0.230
Q86VM9	Zinc finger CCCH domain-containing protein 18	ZC3H18	0.011	-0.039	-0.230
Q2TAY7	WD40 repeat-containing protein SMU1	SMU1	0.067	0.013	-0.233
Q4G0J3	La-related protein 7	LARP7	-0.033	0.172	-0.233
Q9BYV8-3	Isoform 3 of Centrosomal protein of 41 kDa	CEP41		0.215	-0.234

P55265-3	Isoform 3 of Double-stranded RNA-specific adenosine deaminase	ADAR			-0.234
P29083	General transcription factor IIE subunit 1	GTF2E1	0.223	-0.033	-0.234
Q05086-2	Isoform I of Ubiquitin-protein ligase E3A	UBE3A	-0.034	0.041	-0.234
B5TY33	Mitogen-activated protein kinase 14	MAPK14	-0.108	0.165	-0.235
P52566	Rho GDP-dissociation inhibitor 2	ARHGDIB	0.185		-0.235
K7EQE7	Transmembrane protein 161A (Fragment)	TMEM161A		0.373	-0.237
Q9UNW1	Multiple inositol polyphosphate phosphatase 1	MINPP1			-0.237
G3V4W0	Heterogeneous nuclear ribonucleoproteins C1/C2 (Fragment)	HNRNPC	0.246	0.585	-0.237
Q8TCY9-3	Isoform 3 of Up-regulator of cell proliferation	URGCP	0.112		-0.238
A2BED6	Histone-lysine N-methyltransferase EHMT2 (Fragment)	EHMT2	0.075	0.044	-0.239
M0QY61	Protein Smaug homolog 2 (Fragment)	SAMD4B	-0.058		-0.239
Q13451	Peptidyl-prolyl cis-trans isomerase FKBP5	FKBP5	0.061	-0.009	-0.242
Q9UNZ2	NSFL1 cofactor p47	NSFL1C	0.008	0.118	-0.242
Q9BV79	Trans-2-enoyl-CoA reductase, mitochondrial	MECR	0.089		-0.242
P40261	Nicotinamide N-methyltransferase	NNMT			-0.242
P28838-2	Isoform 2 of Cytosol aminopeptidase	LAP3	0.001	-0.079	-0.243
B4DP57	Nucleoporin NUP53	NUP35			-0.244
B1AH87	Putative peripheral benzodiazepine receptor-related protein (Fragment)	TSPO		0.129	-0.245
E9PJW9	Oxysterol-binding protein	OSBPL9	-0.006		-0.245
B1AHC2	Rho GTPase-activating protein 8 (Fragment)	ARHGAP8			-0.246
Q3ZE25	Diacylglycerol kinase alpha	DGKA			-0.246
Q8N3F8	MICAL-like protein 1	MICALL1	0.042	0.098	-0.246
O15270	Serine palmitoyltransferase 2	SPTLC2	0.189	0.392	-0.246
B4E1S6	Syndecan	SDC4	-0.011		-0.246
Q9P0K7-4	Isoform 4 of Ankycorbin	RAI14		0.117	-0.248
Q6ZRV2	Protein FAM83H	FAM83H	-0.007	-0.022	-0.249
Q9Y217-2	Isoform 2 of Myotubularin-related protein 6	MTMR6			-0.249
H3BLV0	Complement decay-accelerating factor (Fragment)	CD55			-0.250
H9KV91	Proline-rich AKT1 substrate 1 (Fragment)	AKT1S1		0.142	-0.250
O94822	E3 ubiquitin-protein ligase listerin	LTN1		0.131	-0.250
Q5QPM7	Proteasome inhibitor PI31 subunit	PSMF1	0.029	0.065	-0.250
Q14258	E3 ubiquitin/ISG15 ligase TRIM25	TRIM25	-0.074	-0.064	-0.252
C9JRJ5	LIM domain-containing protein 1	LIMD1	0.083	0.029	-0.252
Q9NX47	E3 ubiquitin-protein ligase MARCH5	MARC5			-0.252
Q9NQH7-3	Isoform 3 of Probable Xaa-Pro aminopeptidase 3	XPNPEP3	-0.299	-0.186	-0.252
Q8WU90	Zinc finger CCCH domain-containing protein 15	ZC3H15	-0.082	0.021	-0.253
Q8IX12-2	Isoform 2 of Cell division cycle and apoptosis regulator protein 1	CCAR1	-0.045	-0.060	-0.254
Q86SX6	Glutaredoxin-related protein 5, mitochondrial	GLRX5	-0.110	-0.046	-0.255
Q9NQ55-2	Isoform 2 of Suppressor of SWI4 1 homolog	PPAN	0.035	0.108	-0.255
P62993	Growth factor receptor-bound protein 2	GRB2	-0.186	-0.077	-0.256
Q9NXV6	CDKN2A-interacting protein	CDKN2AIP	-0.003	0.028	-0.256
F8W1A4	Adenylate kinase 2, mitochondrial	AK2	-0.010	0.133	-0.257
Q8NBY1	Serine/threonine-protein kinase MST4	MST4	0.221		-0.258
Q5T8U9	UPF0586 protein C9orf41	C9orf41			-0.258
P13674-2	Isoform 2 of Prolyl 4-hydroxylase subunit alpha-1	P4HA1	0.071	0.064	-0.260
Q6IN85	Serine/threonine-protein phosphatase 4 regulatory subunit 3A	SMEK1		0.030	-0.261
Q9BYD1	39S ribosomal protein L13, mitochondrial	MRPL13	0.018		-0.261
Q8TAA9-2	Isoform 2 of Vang-like protein 1	VANGL1	0.032	-0.266	-0.262
Q8N983-4	Isoform 4 of 39S ribosomal protein L43, mitochondrial	MRPL43	0.123	-0.001	-0.262
Q96EY7	Pentatricopeptide repeat domain-containing protein 3, mitochondrial	PTCD3	-0.016	0.036	-0.262
P42356	Phosphatidylinositol 4-kinase alpha	PI4KA	0.139	0.169	-0.263
Q9UM22	Mammalian ependymin-related protein 1	EPDR1			-0.263
P40937-2	Isoform 2 of Replication factor C subunit 5	RFC5	0.022	0.018	-0.264
P11047	Laminin subunit gamma-1	LAMC1	0.188	-0.080	-0.264
P14927	Cytochrome b-c1 complex subunit 7	UQCRB	0.010	-0.045	-0.266
P53990-2	Isoform 2 of IST1 homolog	IST1	-0.010	0.082	-0.266
Q9ULW0	Targeting protein for Xklp2	TPX2	0.199	-0.206	-0.266
Q14669	E3 ubiquitin-protein ligase TRIP12	TRIP12	-0.190	-0.348	-0.267
Q9Y221-2	Isoform 2 of 60S ribosome subunit biogenesis protein NIP7 homolog	NIP7	0.107	-0.211	-0.267
Q969S9-4	Isoform 4 of Ribosome-releasing factor 2, mitochondrial	GFM2			-0.268
A6NH27	ZW10 interactor	ZWINT			-0.270
Q13554-7	Isoform 7 of Calcium/calmodulin-dependent protein kinase type II subunit beta	CAMK2B	0.011	0.122	-0.270
Q9Y6M5	Zinc transporter 1	SLC30A1	0.034		-0.271
O94888	UBX domain-containing protein 7	UBXN7	0.098	-0.185	-0.271
P41223	Protein BUD31 homolog	BUD31	-0.065	-0.243	-0.271
P53384-2	Isoform 2 of Cytosolic Fe-S cluster assembly factor NUBP1	NUBP1		1.085	-0.272
Q13464	Rho-associated protein kinase 1	ROCK1	0.005		-0.273
Q68CQ4	Digestive organ expansion factor homolog	DIEXF		0.038	-0.274
P49588	Alanine--tRNA ligase, cytoplasmic	AARS	0.166	0.098	-0.274
Q5VT52-2	Isoform 2 of Regulation of nuclear pre-mRNA domain-containing protein 2	RPRD2	0.216	0.123	-0.274
O43318-4	Isoform 1D of Mitogen-activated protein kinase kinase kinase 7	MAP3K7	-0.156		-0.275
Q5T2E7	UPF0668 protein C10orf76	C10orf76			-0.276
P45985	Dual specificity mitogen-activated protein kinase kinase 4	MAP2K4		-0.107	-0.276
Q9P2D3-3	Isoform 3 of HEAT repeat-containing protein 5B	HEATR5B			-0.278
Q7Z721	RPS6KB1 protein	RPS6KB1			-0.278
Q16666-3	Isoform 3 of Gamma-interferon-inducible protein 16	IFI16			-0.278
Q08AF3	Schlafen family member 5	SLFN5	0.403		-0.279
Q12797-10	Isoform 10 of Aspartyl/asparaginyl beta-hydroxylase	ASPH	-0.171	0.096	-0.282
P10606	Cytochrome c oxidase subunit 5B, mitochondrial	COX5B	-0.046	-0.101	-0.283
P10253	Lysosomal alpha-glucosidase	GAA		0.174	-0.285
O43592	Exportin-T	XPOT	0.042	-0.071	-0.286
F8W1R7	Myosin light polypeptide 6	MYL6	0.231	-0.032	-0.288
O00560-2	Isoform 2 of Syntenin-1	SDCBP	-0.047	-0.032	-0.289
O15198-2	Isoform B of Mothers against decapentaplegic homolog 9	SMAD9			-0.291
Q9Y6D9	Mitotic spindle assembly checkpoint protein MAD1	MAD1L1	0.146	-0.042	-0.296
Q14146	Unhealthy ribosome biogenesis protein 2 homolog	URB2	-0.003	-0.106	-0.297
B7ZKM0	SART3 protein	SART3	0.044	-0.103	-0.297

P15529-16	Isoform 3 of Membrane cofactor protein	CD46	-0.372	-0.121	-0.297
P17676	CCAAT/enhancer-binding protein beta	CEBPB			-0.298
H0YEB6	Sjoegren syndrome/scleroderma autoantigen 1 (Fragment)	SSSCA1	0.050	0.157	-0.299
O75691	Small subunit processome component 20 homolog	UTP20	0.130	-0.227	-0.301
Q8TEQ8	GPI ethanolamine phosphate transferase 3	PIGO	0.065		-0.302
Q9H6R0	Putative ATP-dependent RNA helicase DHX33	DHX33		0.882	-0.304
E9PK95	Retinoic acid receptor RXR-beta	RXRB	-0.048	0.006	-0.304
P23786	Carnitine O-palmitoyltransferase 2, mitochondrial	CPT2	0.004	0.236	-0.306
P11182	Lipoamide acyltransferase component of branched-chain alpha-keto acid dehydrog	DBT	0.057		-0.309
M0QZQ8	Trafficking protein particle complex subunit 5	TRAPPC5		-0.055	-0.310
Q96S55-2	Isoform 2 of ATPase WRNIP1	WRNIP1	0.117	-0.051	-0.311
Q9BX95	Sphingosine-1-phosphate phosphatase 1	SGPP1	-0.014		-0.312
Q8N9M5	Transmembrane protein 102	TMEM102	-0.162		-0.312
Q9BQ69	O-acetyl-ADP-ribose deacetylase MACROD1	MACROD1	0.248	-0.051	-0.312
G5E9V7	Armadillo repeat containing 8, isoform CRA_d	ARMC8	0.126	0.293	-0.314
Q15345-3	Isoform 3 of Leucine-rich repeat-containing protein 41	LRRC41	0.520	0.190	-0.315
P35579	Myosin-9	MYH9	0.289		-0.315
B4E0Q3	Dynamin-binding protein	DNMBP			-0.315
H0YDJ1	Terminal uridylyltransferase 4 (Fragment)	ZCCHC11			-0.315
O95139-2	Isoform 2 of NADH dehydrogenase [ubiquinone] 1 beta subcomplex subunit 6	NDUFB6	-0.229	0.302	-0.315
O43920	NADH dehydrogenase [ubiquinone] iron-sulfur protein 5	NDUFS5		0.151	-0.318
P19525-2	Isoform 2 of Interferon-induced, double-stranded RNA-activated protein kinase	EIF2AK2	0.108	-0.166	-0.318
Q92522	Histone H1x	H1FX	-0.004	-0.064	-0.319
E7EMH5	Pyridoxal-dependent decarboxylase domain-containing protein 1	PDXDC1	-0.040	0.076	-0.320
I3L400	Serine/threonine-protein kinase SMG1 (Fragment)	SMG1			-0.322
Q9NWX6	Probable tRNA(His) guanylyltransferase	THG1L			-0.323
E7EVX8	U4/U6 small nuclear ribonucleoprotein Prp31	PRPF31	0.019	0.078	-0.324
P49662-2	Isoform 2 of Caspase-4	CASP4	-0.069		-0.329
Q96CT7	Coiled-coil domain-containing protein 124	CCDC124	0.061	-0.181	-0.330
B4DUE9	Serine incorporator 3	SERINC3	0.163	0.160	-0.330
Q10589	Bone marrow stromal antigen 2	BST2			-0.331
Q8N392-2	Isoform 2 of Rho GTPase-activating protein 18	ARHGAP18			-0.331
Q9NZ08	Endoplasmic reticulum aminopeptidase 1	ERAP1	0.356	0.250	-0.332
P25685	DnaJ homolog subfamily B member 1	DNAJB1	0.014	-0.025	-0.332
K7EQ77	NADH dehydrogenase [ubiquinone] 1 alpha subcomplex subunit 11	NDUFA11		0.165	-0.334
B4DDR8	Mediator of RNA polymerase II transcription subunit 24	MED24	-0.041	-0.061	-0.335
C9J8E1	MAP kinase-activated protein kinase 3 (Fragment)	MAPKAPK3	-0.116	-0.051	-0.338
E9PGL4	Beta-hexosaminidase subunit alpha	HEXA	0.139	0.299	-0.338
B7Z1N3	GPI transamidase component PIG-T	PIGT	0.045	0.331	-0.339
P48634	Protein PRRC2A	PRRC2A	-0.103	-0.079	-0.339
P63096	Guanine nucleotide-binding protein G(i) subunit alpha-1	GNAI1			-0.339
Q13107-2	Isoform 2 of Ubiquitin carboxyl-terminal hydrolase 4	USP4	0.047		-0.339
P01034	Cystatin-C	CST3			-0.339
Q8IVD9	NudC domain-containing protein 3	NUDCD3	-0.090	-0.104	-0.340
Q92544	Transmembrane 9 superfamily member 4	TM9SF4	-0.273	-0.314	-0.342
Q5SRE5	Nucleoporin NUP188 homolog	NUP188	0.066	-0.024	-0.345
A6NN80	Annexin	ANXA6	0.179	0.143	-0.346
B4E1Q4	Serine/threonine-protein kinase RIO3	RIOK3	0.110		-0.347
Q96QD8-2	Isoform 2 of Sodium-coupled neutral amino acid transporter 2	SLC38A2			-0.348
P78556-2	Isoform 2 of C-C motif chemokine 20	CCL20			-0.349
Q86UK7-2	Isoform 2 of Zinc finger protein 598	ZNF598		-0.103	-0.351
O43175	D-3-phosphoglycerate dehydrogenase	PHGDH	0.310	0.035	-0.352
P29373	Cellular retinoic acid-binding protein 2	CRABP2	-0.132	0.378	-0.352
Q8TBC4-2	Isoform 2 of NEDD8-activating enzyme E1 catalytic subunit	UBA3	0.342	0.100	-0.354
F5H4T7	Mediator of RNA polymerase II transcription subunit 23	MED23	0.035	0.011	-0.357
P12532	Creatine kinase U-type, mitochondrial	CKMT1A	0.743	0.442	-0.359
H0YGT3	Steroid hormone receptor ERR1 (Fragment)	ESRRA	0.287		-0.360
Q9NX62	Inositol monophosphatase 3	IMPAD1	-0.392	0.026	-0.360
D6R9U8	3-hydroxybutyrate dehydrogenase type 2 (Fragment)	BDH2	0.055	0.291	-0.360
Q9UBF2	Coatomer subunit gamma-2	COPG2	-0.115	0.066	-0.361
P08243-2	Isoform 2 of Asparagine synthetase [glutamine-hydrolyzing]	ASNS	0.348	-0.446	-0.363
K7EP32	UBX domain-containing protein 6 (Fragment)	UBXN6			-0.364
Q8N474	Secreted frizzled-related protein 1	SFRP1	-0.303	-0.191	-0.364
Q9Y617	Phosphoserine aminotransferase	PSAT1	-0.253	-0.226	-0.366
P09012	U1 small nuclear ribonucleoprotein A	SNRPA	0.213	-0.053	-0.369
E7EMN6	Protein phosphatase inhibitor 2 (Fragment)	PPP1R2	-0.191	-0.320	-0.370
Q9NT62-2	Isoform 2 of Ubiquitin-like-conjugating enzyme ATG3	ATG3	-0.122	0.011	-0.373
D6RAN8	39S ribosomal protein L41, mitochondrial	MRPL27	0.090	0.057	-0.374
Q96AE4	Far upstream element-binding protein 1	FUBP1	0.232	0.083	-0.375
Q7Z2T5	TRMT1-like protein	TRMT1L	0.015		-0.377
Q9P0S9	Transmembrane protein 14C	TMEM14C	-0.040		-0.380
Q96TC7	Regulator of microtubule dynamics protein 3	RMDN3	-0.075	0.190	-0.382
Q9Y3L3-2	Isoform 2 of SH3 domain-binding protein 1	SH3BP1	-0.180	-0.065	-0.386
Q16831	Uridine phosphorylase 1	UPP1	0.381		-0.386
B4DS61	SFRS protein kinase 1, isoform CRA_f	SRPK1	-0.125	-0.225	-0.387
Q96JQ2	Calmin	CLMN	0.482	-0.048	-0.392
P08754	Guanine nucleotide-binding protein G(k) subunit alpha	GNAI3	0.102	-0.002	-0.393
Q9NR19	Acetyl-coenzyme A synthetase, cytoplasmic	ACSS2	0.056	-0.171	-0.394
E7EVD1	U4/U6 small nuclear ribonucleoprotein Prp3	PRPF3	0.032	-0.038	-0.395
Q13641	Trophoblast glycoprotein	TPBG	-0.178	-0.118	-0.396
C9JBI3	Phosphoserine phosphatase (Fragment)	PSPH	0.179	0.121	-0.396
P29466-4	Isoform Delta of Caspase-1	CASP1			-0.397
O00625	Pirin	PIR	-0.512	-0.084	-0.399
H7C0Q5	Uncharacterized protein C7orf26 (Fragment)	C7orf26			-0.400
Q5T7A2	Chaperone activity of bc1 complex-like, mitochondrial	ADCK3			-0.404
B4DK80	DNA topoisomerase	TOP3A	0.060	0.315	-0.406
Q9H0W8-2	Isoform 2 of Protein SMG9	SMG9	-0.105		-0.407

P09669	Cytochrome c oxidase subunit 6C	COX6C	-0.177	0.140	-0.409
Q96FX7	tRNA (adenine(58)-N(1))-methyltransferase catalytic subunit TRMT61A	TRMT61A	-0.089	-0.183	-0.415
Q5K651	Sterile alpha motif domain-containing protein 9	SAMD9			-0.422
B4DIT1	Arf-GAP with Rho-GAP domain, ANK repeat and PH domain-containing protein 3	ARAP3	-0.127		-0.422
C9JAW5	HIG1 domain family member 1A, mitochondrial	HIGD1A			-0.424
B4DR80	Serine/threonine-protein kinase 24 12 kDa subunit	STK24	-0.041	0.124	-0.425
P33121	Long-chain-fatty-acid--CoA ligase 1	ACSL1	0.016	0.402	-0.425
Q9UKX7-2	Isoform 2 of Nuclear pore complex protein Nup50	NUP50	-0.251	-0.019	-0.426
Q8NBM4	Ubiquitin-associated domain-containing protein 2	UBAC2	-0.094		-0.426
Q8NC51-3	Isoform 3 of Plasminogen activator inhibitor 1 RNA-binding protein	SERBP1			-0.432
Q9Y2Z2-6	Isoform 7 of Protein MTO1 homolog, mitochondrial	MTO1	0.114		-0.438
B4DLC8	Signal transducer and activator of transcription 2	STAT2			-0.448
P55210-4	Isoform 4 of Caspase-7	CASP7	0.056		-0.448
P49790	Nuclear pore complex protein Nup153	NUP153	-0.028	0.089	-0.452
Q9BUI4	DNA-directed RNA polymerase III subunit RPC3	POLR3C	0.022	0.136	-0.458
Q15646-3	Isoform 3 of 2'-5'-oligoadenylate synthase-like protein	OASL	0.440		-0.461
J3KS22	L-xylulose reductase (Fragment)	DCXR	0.069	0.157	-0.465
Q9BRZ2	E3 ubiquitin-protein ligase TRIM56	TRIM56	-0.092	0.000	-0.467
P04183	Thymidine kinase, cytosolic	TK1	-0.198	-0.035	-0.468
Q9UMR2	ATP-dependent RNA helicase DDX19B	DDX19B	0.015		-0.474
Q9P015	39S ribosomal protein L15, mitochondrial	MRPL15	-0.125	0.057	-0.474
Q8WVK2	U4/U6.U5 small nuclear ribonucleoprotein 27 kDa protein	SNRNP27	0.051	0.051	-0.475
Q8N573-3	Isoform 3 of Oxidation resistance protein 1	OXR1	-0.191	0.362	-0.476
E9PQT7	Ubiquitin/ISG15-conjugating enzyme E2 L6 (Fragment)	UBE2L6	0.242		-0.477
Q13423	NAD(P) transhydrogenase, mitochondrial	NNT	-0.639	-0.053	-0.477
Q12789-3	Isoform 2 of General transcription factor 3C polypeptide 1	GTF3C1	0.031	0.051	-0.478
E9PH64	NADH dehydrogenase [ubiquinone] 1 beta subcomplex subunit 9	NDUFB9	-0.009	0.037	-0.481
O76071	Probable cytosolic iron-sulfur protein assembly protein CIAO1	CIAO1	-0.026	-0.005	-0.481
O94966-6	Isoform 6 of Ubiquitin carboxyl-terminal hydrolase 19	USP19	-0.049		-0.482
F5GYS8	Homeobox protein Meis1	MEIS1			-0.483
Q96LD4	Tripartite motif-containing protein 47	TRIM47	0.140	-0.061	-0.485
Q9NRG7-2	Isoform 2 of Epimerase family protein SDR39U1	SDR39U1			-0.491
H6UMI1	GABARAP-a	GABARAP	0.212	0.068	-0.492
Q8WU76-2	Isoform 2 of Sec1 family domain-containing protein 2	SCFD2			-0.494
P11802	Cyclin-dependent kinase 4	CDK4	-0.071	-0.056	-0.495
P03973	Antileukoproteinase	SLPI			-0.498
D6RG34	Solute carrier family 35 member B1 (Fragment)	SLC35B1	0.030		-0.502
Q9NWU5	39S ribosomal protein L22, mitochondrial	MRPL22	0.128	0.056	-0.505
Q9NWT1	p21-activated protein kinase-interacting protein 1	PAK1IP1	0.033	-0.069	-0.509
Q8NI27	THO complex subunit 2	THOC2	0.037	-0.050	-0.512
Q9H4G0-4	Isoform 4 of Band 4.1-like protein 1	EPB41L1	-0.372	-0.424	-0.514
Q7Z6V2	Plasma alpha-L-fucosidase (Fragment)	FUCA2			-0.515
H0Y4P7	Collagen alpha-1(XII) chain (Fragment)	COL12A1			-0.520
Q9BZH6	WD repeat-containing protein 11	WDR11		0.044	-0.528
Q5TBH6	Dihydroxyacetone phosphate acyltransferase (Fragment)	GNPAT	0.143	0.449	-0.531
Q9NVM6	DnaJ homolog subfamily C member 17	DNAJC17	-0.011	-0.011	-0.532
Q53EL6-2	Isoform 2 of Programmed cell death protein 4	PDCD4	0.376	0.058	-0.539
P05161	Ubiquitin-like protein ISG15	ISG15	0.339	0.311	-0.543
Q6DKJ4	Nucleoredoxin	NXN		-0.059	-0.543
O43513	Mediator of RNA polymerase II transcription subunit 7	MED7			-0.545
Q9C002	Normal mucosa of esophagus-specific gene 1 protein	NMES1			-0.545
E9PPA0	Chitinase domain-containing protein 1 (Fragment)	CHID1			-0.547
F5H4C2	Ran-binding protein 3	RANBP3	0.061	0.032	-0.558
Q9NUD5	Zinc finger CCHC domain-containing protein 3	ZCCHC3	0.252	0.096	-0.562
Q9Y3Z3-3	Isoform 3 of Deoxynucleoside triphosphate triphosphohydrolase SAMHD1	SAMHD1	0.292		-0.564
Q14241	Transcription elongation factor B polypeptide 3	TCEB3	0.276	-0.193	-0.565
B4E1N1	Armadillo repeat-containing protein 6	ARMC6	0.012	-0.129	-0.567
F5GYC4	Ribosomal protein S6 kinase alpha-3	RPS6KA3	0.093	0.088	-0.571
O60333-3	Isoform 3 of Kinesin-like protein KIF1B	KIF1B		-0.144	-0.571
O15231-4	Isoform 4 of Zinc finger protein 185	ZNF185	0.089		-0.585
Q14554	Protein disulfide-isomerase A5	PDIA5	0.087	0.102	-0.596
Q9Y496	Kinesin-like protein KIF3A	KIF3A			-0.606
G3V5B2	Legumain (Fragment)	LGMN	0.094	-0.222	-0.612
Q08380	Galectin-3-binding protein	LGALS3BP	0.302		-0.613
B4E0V0	Pyridoxine-5'-phosphate oxidase	PNPO	-0.196		-0.613
A1A528	Centromere/kinetochore protein zw10 homolog	ZW10	-0.005	0.030	-0.623
Q9NV96	Cell cycle control protein 50A	TMEM30A	0.073	0.228	-0.624
Q9UEE9	Craniofacial development protein 1	CFDP1	-0.067	-0.061	-0.657
B4DDV1	Interferon-induced protein with tetratricopeptide repeats 5	IFIT5	-0.167	-0.261	-0.682
Q8NHU6-3	Isoform 3 of Tudor domain-containing protein 7	TDRD7	0.270		-0.686
C9JX83	U3 small nucleolar ribonucleoprotein protein MPP10	MPHOSPH10	-0.131	-0.114	-0.688
Q8WXA9-2	Isoform 2 of Splicing regulatory glutamine/lysine-rich protein 1	SREK1	-0.029	0.027	-0.699
B4DU10	N-alpha-acetyltransferase 40	NAA40	-0.584		-0.702
P14209-3	Isoform 3 of CD99 antigen	CD99		-0.045	-0.710
Q9UQ13	Leucine-rich repeat protein SHOC-2	SHOC2			-0.717
P35580	Myosin-10	MYH10	0.264	0.294	-0.731
Q00534	Cyclin-dependent kinase 6	CDK6	0.126	0.125	-0.766
Q14139	Ubiquitin conjugation factor E4 A	UBE4A	0.167		-0.767
Q96IR7	4-hydroxyphenylpyruvate dioxygenase-like protein	HPDL	-0.085		-0.770
Q2TAL8	Glutamine-rich protein 1	QRICH1	-0.160	-0.175	-0.799
O95479	GDH/6PGL endoplasmic bifunctional protein	H6PD	0.034		-0.818
Q8IY21	Probable ATP-dependent RNA helicase DDX60	DDX60	0.670		-0.840
Q9Y6K5	2'-5'-oligoadenylate synthase 3	OAS3	0.348		-0.852
A8MWD9	Small nuclear ribonucleoprotein G-like protein	2	-0.124	-0.424	-0.858
Q6IAK0	SELT protein	SELT	0.065	0.093	-0.910
P32320	Cytidine deaminase	CDA	-0.023		-0.920
B8ZZS4	Ankyrin repeat and zinc finger domain-containing protein 1	ANKZF1			-0.953

D3DR31	Interferon-induced protein with tetratricopeptide repeats 1	IFIT1	0.643		-0.964
H0Y8H0	Probable ATP-dependent RNA helicase DDX60-like (Fragment)	DDX60L	0.660		-1.073
H0YBY9	Arf-GAP with SH3 domain, ANK repeat and PH domain-containing protein 1 (Fragr	ASAP1			-1.085
P82970	High mobility group nucleosome-binding domain-containing protein 5	HMGN5		0.001	-1.123
F5H8D7	DNA repair protein XRCC1	XRCC1	0.046		-1.169
F5H5W6	Probable ATP-dependent RNA helicase DDX58	DDX58	0.800		-1.172
B1AZV3	Antigen peptide transporter 1	TAP1	0.467	0.313	-1.186
Q9BYX2-2	Isoform 2 of TBC1 domain family member 2A	TBC1D2	0.079		-1.256
J3KSZ0	Eukaryotic initiation factor 4A-I (Fragment)	EIF4A1			-1.473
P09913	Interferon-induced protein with tetratricopeptide repeats 2	IFIT2			-1.486
Q13144	Translation initiation factor eIF-2B subunit epsilon	EIF2B5	-0.011	0.102	-1.521
Q2TAM5	RELA protein	RELA	-0.150	0.085	-1.525
F8W8T1	Interferon-induced GTP-binding protein Mx1, N-terminally processed	MX1	1.664		-1.859
Q8N726-2	Isoform 6 of Cyclin-dependent kinase inhibitor 2A, isoform 4	CDKN2A	-0.633	0.046	
Q13642-3	Isoform 3 of Four and a half LIM domains protein 1	FHL1	-0.615		
H3BLV4	Sushi domain-containing protein 1 (Fragment)	SUSD1	-0.565		
M0QY25	Prostate tumor-overexpressed gene 1 protein	PTOV1	-0.558	0.000	
Q8N442	Translation factor GUF1, mitochondrial	GUF1	-0.544		
Q14938-5	Isoform 5 of Nuclear factor 1 X-type	NFIX	-0.522	-1.035	
Q8N6M0	OTU domain-containing protein 6B	OTUD6B	-0.521		
P31944	Caspase-14	CASP14	-0.520		
Q9Y3E7-4	Isoform 4 of Charged multivesicular body protein 3	CHMP3	-0.499		
Q5VT66	MOSC domain-containing protein 1, mitochondrial	MARC1	-0.464		
Q8WTV0-4	Isoform 4 of Scavenger receptor class B member 1	SCARB1	-0.438	0.327	
Q9UIL1-3	Isoform 3 of Short coiled-coil protein	SCOC	-0.429		
Q12882	Dihydropyrimidine dehydrogenase [NADP(+)]	DPYD	-0.415		
D6RA96	Protein RTEL1-TNFRSF6B	RTEL1-TNFRSF6B	-0.405		
F8WDG7	Inactive tyrosine-protein kinase 7	PTK7	-0.392		
H0YL98	DDB1- and CUL4-associated factor 11 (Fragment)	DCAF11	-0.381		
Q9BYG8	Gasdermin-C	GSDMC	-0.377		
D6R9X2	PHD finger protein 3	PHF3	-0.374		
A1A4S6	Rho GTPase-activating protein 10	ARHGAP10	-0.374		
Q52LW3	Rho GTPase-activating protein 29	ARHGAP29	-0.369	-0.254	
H0YC88	Synembryn-A (Fragment)	RIC8A	-0.368		
O14524	Transmembrane protein 194A	TMEM194A	-0.361		
H0Y6C3	Pyrroline-5-carboxylate reductase 3 (Fragment)	PYCRL	-0.349		
P56381	ATP synthase subunit epsilon, mitochondrial	ATP5E	-0.340	-0.639	
F5H5P4	Proto-oncogene vav	VAV1	-0.339	-0.140	
F5H2B0	Phosphatidylinositol 4-phosphate 3-kinase C2 domain-containing subunit alpha	PIK3C2A	-0.332		
P00966	Argininosuccinate synthase	ASS1	-0.326	-0.146	
O94925-3	Isoform 3 of Glutaminase kidney isoform, mitochondrial	GLS	-0.322	-0.451	
Q99808	Equilibrative nucleoside transporter 1	SLC29A1	-0.320	-0.138	
P16220-2	Isoform CREB-B of Cyclic AMP-responsive element-binding protein 1	CREB1	-0.318	-0.418	
Q4LDG9-2	Isoform 2 of Dynein light chain 1, axonemal	DNAL1	-0.313		
A8MYV2	LUC7-like (S. cerevisiae)	LUC7L	-0.311	0.008	
Q9H0U9	Testis-specific Y-encoded-like protein 1	TSPYL1	-0.308	-0.127	
Q01658	Protein Dr1	DR1	-0.293		
Q93100-4	Isoform 4 of Phosphorylase b kinase regulatory subunit beta	PHKB	-0.289		
Q99523	Sortilin	SORT1	-0.288	-0.449	
Q14151	Scaffold attachment factor B2	SAFB2	-0.284	-0.448	
Q5MNZ6	WD repeat domain phosphoinositide-interacting protein 3	WDR45B	-0.281	-0.028	
P24311	Cytochrome c oxidase subunit 7B, mitochondrial	COX7B	-0.280	-0.514	
H0Y3K7	Actin-binding LIM protein 1	ABLIM1	-0.279	0.036	
Q9UBX3	Mitochondrial dicarboxylate carrier	SLC25A10	-0.279	0.033	
Q9H8W4	Pleckstrin homology domain-containing family F member 2	PLEKHF2	-0.278	0.244	
P21980	Protein-glutamine gamma-glutamyltransferase 2	TGM2	-0.276		
H0YNA7	Signal peptide peptidase-like 2A (Fragment)	SPPL2A	-0.276	0.167	
H3BTY4	Ceroid-lipofuscinosis neuronal protein 6	CLN6	-0.275	-0.026	
O94788-4	Isoform 4 of Retinal dehydrogenase 2	ALDH1A2	-0.273		
P13051	Uracil-DNA glycosylase	UNG	-0.273	-0.096	
Q2KHT3-2	Isoform 2 of Protein CLEC16A	CLEC16A	-0.273		
A2TJX0	Krueppel-like factor 5	KLF5	-0.273		
Q00059	Transcription factor A, mitochondrial	TFAM	-0.273	-0.042	
Q8N6N3	UPF0690 protein C1orf52	C1orf52	-0.272	-0.059	
F5H729	BTB/POZ domain-containing adapter for CUL3-mediated RhoA degradation protein	KCTD10	-0.270	-0.727	
I3L2L5	Protein FAM195B	FAM195B	-0.270	-0.383	
O60662-2	Isoform Short of Kelch-like protein 41	KLHL41	-0.265		
H0YCF4	Gem-associated protein 2 (Fragment)	GEMIN2	-0.264	-0.050	
E9PFH7	C-Jun-amino-terminal kinase-interacting protein 3	MAPK8IP3	-0.261		
I3L3E8	Mediator of RNA polymerase II transcription subunit 11	MED11	-0.259		
P38935	DNA-binding protein SMUBP-2	IGHMBP2	-0.254		
Q9H7E2-2	Isoform 2 of Tudor domain-containing protein 3	TDRD3	-0.250	-0.174	
O95716	Ras-related protein Rab-3D	RAB3D	-0.248	-0.403	
Q9UP95-4	Isoform 4 of Solute carrier family 12 member 4	SLC12A4	-0.239		
K7ELF8	Inositol monophosphatase 2	IMPA2	-0.237	-0.022	
Q6GMV3	Putative peptidyl-tRNA hydrolase PTRHD1	PTRHD1	-0.235	-0.305	
Q8WWI5-3	Isoform 3 of Choline transporter-like protein 1	SLC44A1	-0.234		
Q7Z6M1-2	Isoform 2 of Rab9 effector protein with kelch motifs	RABEPK	-0.232		
Q9H8Y8	Golgi reassembly-stacking protein 2	GORASP2	-0.231	-0.263	
P40306	Proteasome subunit beta type-10	PSMB10	-0.227	-0.330	
Q9H3H3	UPF0696 protein C11orf68	C11orf68	-0.227	-0.117	
D6R9B3	Multidrug resistance-associated protein 7 (Fragment)	ABCC10	-0.227		
P0DI83	Ras-related protein Rab-34, isoform NARR	RAB34	-0.226	-0.375	
P15735-2	Isoform 2 of Phosphorylase b kinase gamma catalytic chain, liver/testis isoform	PHKG2	-0.224	-0.268	
Q93034	Cullin-5	CUL5	-0.224		
P00338-2	Isoform 2 of L-lactate dehydrogenase A chain	LDHA	-0.223	-0.007	
P16402	Histone H1.3	HIST1H1D	-0.223	-0.030	

Q12765	Secernin-1	SCRN1	-0.222	-0.022	
M0QY45	Zinc finger protein 729	ZNF729	-0.222		
Q92667	A-kinase anchor protein 1, mitochondrial	AKAP1	-0.221	-0.046	
E9PHV4	DNA-directed RNA polymerase II subunit RPB4	POLR2D	-0.221		
Q8TCG1	Protein CIP2A	KIAA1524	-0.220	0.077	
Q96S44	TP53-regulating kinase	TP53RK	-0.219		
Q15043-2	Isoform 2 of Zinc transporter ZIP14	SLC39A14	-0.217	0.189	
Q32P41	tRNA (guanine(37)-N1)-methyltransferase	TRMT5	-0.214		
H0YES2	DEP domain-containing protein 1A (Fragment)	DEPDC1	-0.212		
Q0VGL1	Ragulator complex protein LAMTOR4	LAMTOR4	-0.212	-0.212	
Q9H009	Nascent polypeptide-associated complex subunit alpha-2	NACA2	-0.211		
P09884	DNA polymerase alpha catalytic subunit	POLA1	-0.208	-0.116	
K7ENJ3	Kinesin light chain 3 (Fragment)	KLC3	-0.208		
F5H1R7	Protein FLJ22184	FLJ22184	-0.207		
O75969	A-kinase anchor protein 3	AKAP3	-0.206		
Q8NBN3-3	Isoform 3 of Transmembrane protein 87A	TMEM87A	-0.205	-0.149	
Q02539	Histone H1.1	HIST1H1A	-0.205	-0.084	
Q06265	Exosome complex component RRP45	EXOSC9	-0.204	-0.051	
P12109	Collagen alpha-1(VI) chain	COL6A1	-0.203	0.135	
B1AHQ6	Centromere protein M	CENPM	-0.201	0.207	
Q8NF37	Lysophosphatidylcholine acyltransferase 1	LPCAT1	-0.200	-0.379	
F5GX88	Uncharacterized protein	ENG	-0.200	0.391	
Q13526	Peptidyl-prolyl cis-trans isomerase NIMA-interacting 1	PIN1	-0.200	-0.207	
Q9Y6Q5	AP-1 complex subunit mu-2	AP1M2	-0.200		
P61024	Cyclin-dependent kinases regulatory subunit 1	CKS1B	-0.198	-0.659	
Q9H6F5	Coiled-coil domain-containing protein 86	CCDC86	-0.197		
A0AV96-2	Isoform 2 of RNA-binding protein 47	RBM47	-0.197		
O15116	U6 snRNA-associated Sm-like protein LSm1	LSM1	-0.196	-0.204	
O15020-2	Isoform 2 of Spectrin beta chain, non-erythrocytic 2	SPTBN2	-0.195		
Q14315-2	Isoform 2 of Filamin-C	FLNC	-0.193		
P51397	Death-associated protein 1	DAP	-0.192	-0.064	
Q9Y3C4	TP53RK-binding protein	TPRKB	-0.192	-0.066	
Q9NWB7	Intraflagellar transport protein 57 homolog	IFT57	-0.192		
P61978-3	Isoform 3 of Heterogeneous nuclear ribonucleoprotein K	HNRNPK	-0.191	-0.345	
H7C4B1	EF-hand domain-containing protein 1	EFHC1	-0.191		
Q92783-2	Isoform 2 of Signal transducing adapter molecule 1	STAM	-0.190	-0.050	
M0QYT9	Interferon regulatory factor 3	IRF3	-0.187		
J3QLD9	Flotillin-2	FLOT2	-0.187		
Q7L5D6	Golgi to ER traffic protein 4 homolog	GET4	-0.186	-0.723	
O43633	Charged multivesicular body protein 2a	CHMP2A	-0.186	-0.050	
Q9P000-2	Isoform 2 of COMM domain-containing protein 9	COMMD9	-0.185		
P61978	Heterogeneous nuclear ribonucleoprotein K	HNRNPK	-0.185	-0.131	
Q8N5M4	Tetratricopeptide repeat protein 9C	TTC9C	-0.185	-0.126	
Q16512	Serine/threonine-protein kinase N1	PKN1	-0.184	-0.091	
Q5T0Z8	Uncharacterized protein C6orf132	C6orf132	-0.183		
P40123	Adenylyl cyclase-associated protein 2	CAP2	-0.182		
D6REK3	Peptidyl-prolyl cis-trans isomerase CWC27 homolog	CWC27	-0.180	0.032	
Q9Y4F1	FERM, RhoGEF and pleckstrin domain-containing protein 1	FARP1	-0.179	0.057	
P54753	Ephrin type-B receptor 3	EPHB3	-0.179	-0.178	
Q7Z2K8-2	Isoform 2 of G protein-regulated inducer of neurite outgrowth 1	GPRIN1	-0.177		
Q8WWT9-4	Isoform 4 of Solute carrier family 13 member 3	SLC13A3	-0.176		
R4GMX0	YTH domain family protein 3	YTHDF3	-0.176	-0.037	
Q5TFQ8	Signal-regulatory protein beta-1 isoform 3	SIRPB1	-0.176		
Q6P1K2-4	Isoform 4 of Polyamine-modulated factor 1	PMF1	-0.176	-0.050	
Q96ST8-2	Isoform 2 of Centrosomal protein of 89 kDa	CEP89	-0.175	0.180	
H7C5V3	28S ribosomal protein S35, mitochondrial (Fragment)	MRPS28	-0.175	-0.203	
Q9Y4K1	Absent in melanoma 1 protein	AIM1	-0.173	0.051	
C9JMQ4	NCK-interacting protein with SH3 domain (Fragment)	NCKIPSD	-0.173		
Q9BTM9	Ubiquitin-related modifier 1 homolog	URM1	-0.171		
Q5TID7	Coiled-coil domain-containing protein 181	CCDC181	-0.169	-0.015	
Q9NRD9-2	Isoform 2 of Dual oxidase 1	DUOX1	-0.169	-0.118	
Q6ZRS2-3	Isoform 3 of Helicase SRCAP	SRCAP	-0.169		
O14777	Kinetochore protein NDC80 homolog	NDC80	-0.168	0.122	
J3QLD5	tRNA-dihydrouridine(20) synthase [NAD(P)+]-like (Fragment)	DUS2L	-0.168		
C9JJX6	Armadillo repeat protein deleted in velo-cardio-facial syndrome	ARVCF	-0.167		
O95239	Chromosome-associated kinesin KIF4A	KIF4A	-0.165	0.005	
E9PCN5	CCR4-NOT transcription complex subunit 10	CNOT10	-0.165	-0.042	
B2RCS5	Actinin, alpha 2, isoform CRA_b	ACTN2	-0.164	0.328	
Q9BYB4	Guanine nucleotide-binding protein subunit beta-like protein 1	GNB1L	-0.164		
Q13112	Chromatin assembly factor 1 subunit B	CHAF1B	-0.164		
O15027-2	Isoform 2 of Protein transport protein Sec16A	SEC16A	-0.161	0.104	
D6RBG4	Signal recognition particle 19 kDa protein	SRP19	-0.160	0.116	
Q14145	Kelch-like ECH-associated protein 1	KEAP1	-0.160	-0.181	
O95721	Synaptosomal-associated protein 29	SNAP29	-0.160		
Q9BZL1	Ubiquitin-like protein 5	UBL5	-0.159	-0.559	
E7EX70	DNA-directed RNA polymerase I subunit RPA49	POLR1E	-0.158		
P16989-2	Isoform 2 of Y-box-binding protein 3	YBX3	-0.158		
Q96R06	Sperm-associated antigen 5	SPAG5	-0.158		
Q99541	Perilipin-2	PLIN2	-0.157	-0.520	
F8VYE8	Serine/threonine-protein phosphatase	PPP1CC	-0.157	-0.327	
H7C252	Intraflagellar transport protein 172 homolog (Fragment)	IFT172	-0.156	-0.139	
P19838	Nuclear factor NF-kappa-B p105 subunit	NFKB1	-0.156	-0.108	
G3XAB3	Tetratricopeptide repeat domain 17, isoform CRA_c	TTC17	-0.154	0.142	
Q9UBI6	Guanine nucleotide-binding protein G(I)/G(S)/G(O) subunit gamma-12	GNG12	-0.154		
E2QRB3	Pyrroline-5-carboxylate reductase 1, isoform CRA_c	PYCR1	-0.154	0.067	
Q9P0V9-3	Isoform 3 of Septin-10	SEPT10	-0.154	0.082	
Q0VDF9	Heat shock 70 kDa protein 14	HSPA14	-0.152	-0.187	

O60293-2	Isoform 2 of Zinc finger C3H1 domain-containing protein	ZFC3H1	-0.151		
A1A5D9	Bicaudal D-related protein 2	CCDC64B	-0.151		
Q96HY6-2	Isoform 2 of DDRGK domain-containing protein 1	DDRGK1	-0.150	0.231	
P16422	Epithelial cell adhesion molecule	EPCAM	-0.150	-0.098	
E9PS71	Peroxisomal biogenesis factor 19	PEX19	-0.150	-0.026	
Q9NR09	Baculoviral IAP repeat-containing protein 6	BIRC6	-0.149	0.583	
D6RBN9	RELT-like protein 1 (Fragment)	RELL1	-0.149	-0.814	
D6RBS5	ELMO domain-containing protein 2 (Fragment)	ELMOD2	-0.149	0.054	
Q9H5V8-3	Isoform 3 of CUB domain-containing protein 1	CDCP1	-0.148		
F5H845	Amyloid-like protein 2	APLP2	-0.147		
Q8WZ42-12	Isoform 12 of Titin	TTN	-0.147	-0.205	
J3KRZ8	Testis-expressed sequence 2 protein (Fragment)	TEX2	-0.147		
K7EP22	Synergin gamma (Fragment)	SYNRG	-0.146		
Q96B26	Exosome complex component RRP43	EXOSC8	-0.146		
P05026-2	Isoform 2 of Sodium/potassium-transporting ATPase subunit beta-1	ATP1B1	-0.146		
P08651-4	Isoform 3 of Nuclear factor 1 C-type	NFIC	-0.146	-0.567	
P49795	Regulator of G-protein signaling 19	RGS19	-0.145		
P09038-2	Isoform 3 of Fibroblast growth factor 2	FGF2	-0.145		
Q9NRC1-7	Isoform 7 of Suppressor of tumorigenicity 7 protein	ST7	-0.145	-0.453	
H0Y8X9	Calcium-transporting ATPase type 2C member 1 (Fragment)	ATP2C1	-0.144		
I3L3W7	Protein spinster homolog 3	SPNS3	-0.144		
O76061	Stanniocalcin-2	STC2	-0.144		
E7EUI8	Glucose-6-phosphate 1-dehydrogenase (Fragment)	G6PD	-0.143		
F5H1E4	Probable G-protein-coupled receptor 113	GPR113	-0.143		
Q5JYT7	Uncharacterized protein KIAA1755	KIAA1755	-0.142	0.095	
J3KP22	Receptor-type tyrosine-protein phosphatase alpha	PTPRA	-0.141	-0.368	
B7Z1U0	Metal transporter CNNM4	CNNM4	-0.140	-0.112	
B7WPG3	Heterogeneous nuclear ribonucleoprotein L-like	HNRPLL	-0.140	-0.023	
Q8IWA4	Mitofusin-1	MFN1	-0.140	0.023	
Q9Y2S0	DNA-directed RNA polymerases I and III subunit RPAC2	POLR1D	-0.140	-0.190	
Q12959-5	Isoform 5 of Disks large homolog 1	DLG1	-0.139	-0.078	
O43824	Putative GTP-binding protein 6	GTPBP6	-0.139	-0.027	
H0YDF9	Protein scribble homolog (Fragment)	SCRIB	-0.138		
Q9BUV8-4	Isoform 4 of Uncharacterized protein C20orf24	C20orf24	-0.138	0.017	
E9PQC4	Kinetochore protein Nuf2	NUF2	-0.138		
Q9HB65-2	Isoform 2 of RNA polymerase II elongation factor ELL3	ELL3	-0.138		
Q7Z7F0-3	Isoform 3 of UPF0469 protein KIAA0907	KIAA0907	-0.137	-0.084	
B7Z6P0	Cytosolic Fe-S cluster assembly factor NUBP2	NUBP2	-0.137	0.033	
Q86TG7-2	Isoform RF1 of Retrotransposon-derived protein PEG10	PEG10	-0.137	0.034	
I3L2R3	Nuclear distribution protein nudE homolog 1 (Fragment)	NDE1	-0.137	0.127	
P33552	Cyclin-dependent kinases regulatory subunit 2	CKS2	-0.136	-0.848	
Q9BT73	Proteasome assembly chaperone 3	PSMG3	-0.136	0.137	
Q9UC07	Zinc finger protein 69	ZNF69	-0.136	-0.035	
E9PIH6	EH domain-binding protein 1-like protein 1 (Fragment)	EHBP1L1	-0.136		
E7ETA6	Methyl-CpG-binding domain protein 1	PCM1	-0.135	-0.121	
K7EQ85	Alanyl-tRNA-editing protein Aarsd1 (Fragment)	AARSD1	-0.134	-0.136	
O95218-2	Isoform 2 of Zinc finger Ran-binding domain-containing protein 2	ZRANB2	-0.134	0.141	
Q9BXR0	Queuine tRNA-ribosyltransferase	QTRT1	-0.134		
Q96S66-2	Isoform 2 of Chloride channel CLIC-like protein 1	CLCC1	-0.133	-0.054	
Q9NZN8-3	Isoform 3 of CCR4-NOT transcription complex subunit 2	CNOT2	-0.133	-0.056	
Q9Y371	Endophilin-B1	SH3GLB1	-0.132	-0.126	
Q16864	V-type proton ATPase subunit F	ATP6V1F	-0.131		
B4DKA8	Ubiquitin carboxyl-terminal hydrolase	USP8	-0.131	0.071	
Q9NPF4	Probable tRNA threonylcarbamoyladenosine biosynthesis proteinGEP	OSGEP	-0.130	0.093	
P55854	Small ubiquitin-related modifier 3	SUMO3	-0.129	-0.157	
Q05639	Elongation factor 1-alpha 2	EEF1A2	-0.129	0.393	
P63092-3	Isoform 3 of Guanine nucleotide-binding protein G(s) subunit alpha isoforms short	GNAS	-0.129	0.031	
Q86WB0-3	Isoform 3 of Nuclear-interacting partner of ALK	ZC3HC1	-0.129	0.105	
C9J3F6	TBC1 domain family member 5	TBC1D5	-0.128	0.032	
Q13243	Serine/arginine-rich splicing factor 5	SRSF5	-0.127	-0.168	
Q8WYQ0	Peroxin Pex6p	PEX6	-0.127		
O00622	Protein CYR61	CYR61	-0.127	-0.385	
Q9UBZ9-2	Isoform 2 of DNA repair protein REV1	REV1	-0.126	-0.161	
Q86YV6	Myosin light chain kinase family member 4	MYLK4	-0.125		
Q9UK61-2	Isoform 2 of Protein FAM208A	FAM208A	-0.125	-0.206	
A0MZ66-4	Isoform 4 of Shootin-1	KIAA1598	-0.125	0.374	
P04818	Thymidylate synthase	TYMS	-0.124	-0.132	
Q9Y4D1-3	Isoform 3 of Disheveled-associated activator of morphogenesis 1	DAAM1	-0.124		
Q9BW60-2	Isoform 2 of Elongation of very long chain fatty acids protein 1	ELOVL1	-0.124	-0.003	
Q8TF42	Ubiquitin-associated and SH3 domain-containing protein B	UBASH3B	-0.123	0.010	
Q9BV44	THUMP domain-containing protein 3	THUMPD3	-0.122	0.031	
Q9BQ67	Glutamate-rich WD repeat-containing protein 1	GRWD1	-0.122	0.065	
Q9UKB1-2	Isoform A of F-box/WD repeat-containing protein 11	FBXW11	-0.122		
O15438	Canalicular multispecific organic anion transporter 2	ABCC3	-0.122		
D6RCL3	Probable dimethyladenosine transferase	DIMT1	-0.121	0.055	
P21580	Tumor necrosis factor alpha-induced protein 3	TNFAIP3	-0.121		
B8ZZ56	AN1-type zinc finger protein 2B	ZFAND2B	-0.120		
Q13158	Protein FADD	FADD	-0.119		
F8W840	YTH domain family protein 1	YTHDF1	-0.119	0.191	
Q16891-4	Isoform 4 of Mitochondrial inner membrane protein	IMMT	-0.118	-0.127	
P41247-2	Isoform 2 of Patatin-like phospholipase domain-containing protein 4	PNPLA4	-0.118		
Q8WUA8	Tsukushin	TSKU	-0.118		
Q96P48-3	Isoform 3 of Arf-GAP with Rho-GAP domain, ANK repeat and PH domain-containin	ARAP1	-0.117		
K7ENR6	Proteasome assembly chaperone 2	PSMG2	-0.116	0.074	
C9JM79	Peptidyl-prolyl cis-trans isomerase G (Fragment)	PPIG	-0.116		
Q6ZUS5-2	Isoform 2 of Coiled-coil domain-containing protein 121	CCDC121	-0.116	-0.234	
Q8WUX9	Charged multivesicular body protein 7	CHMP7	-0.115		

B4E2P0	Mediator of RNA polymerase II transcription subunit 6	MED6	-0.115	-0.050	
Q86SZ2	Trafficking protein particle complex subunit 6B	TRAPPC6B	-0.114		
A6NFE2	Uncharacterized protein C12orf70	C12orf70	-0.114		
E9PB51	RNA-binding protein 4 (Fragment)	RBM4	-0.112	-0.063	
Q5JXX4	Mortality factor 4-like protein 2	MORF4L2	-0.111	-0.128	
H0Y7P8	Vacuolar protein sorting-associated protein 13A (Fragment)	VPS13A	-0.111		
Q86WJ1-4	Isoform 4 of Chromodomain-helicase-DNA-binding protein 1-like	CHD1L	-0.110	0.061	
Q5SWK9	Triadin	TRDN	-0.110	-0.167	
H3BSB3	M-phase phosphoprotein 6	MPHOSPH6	-0.110	-0.181	
Q8IVM0-2	Isoform 2 of Coiled-coil domain-containing protein 50	CCDC50	-0.110	-0.090	
C9JAX1	Frataxin, mitochondrial	FXN	-0.109	0.038	
O75794	Cell division cycle protein 123 homolog	CDC123	-0.109	-0.310	
P51808	Dynein light chain Tctex-type 3	DYNLT3	-0.108	0.217	
B4DH53	MAP1S light chain	MAP1S	-0.108	-0.254	
Q9UKK3	Poly [ADP-ribose] polymerase 4	PARP4	-0.108		
F5GY93	Cob(I)yrinic acid a,c-diamide adenosyltransferase, mitochondrial (Fragment)	MMAB	-0.108		
Q6PL18	ATPase family AAA domain-containing protein 2	ATAD2	-0.108	-0.239	
Q53H96	Pyrroline-5-carboxylate reductase 3	PYCRL	-0.105		
P04040	Catalase	CAT	-0.104	0.079	
O95400	CD2 antigen cytoplasmic tail-binding protein 2	CD2BP2	-0.104	-0.170	
Q86UT6-2	Isoform 2 of NLR family member X1	NLRX1	-0.104		
C9JG97	Angio-associated migratory cell protein	AAMP	-0.104		
Q96L93-5	Isoform 4 of Kinesin-like protein KIF16B	KIF16B	-0.104	-0.363	
C9JXF9	Insulin-like growth factor-binding protein 1	IGFBP1	-0.103		
A6NDJ8	Putative Rab-43-like protein ENSP00000330714	5	-0.103		
Q6ZRR9	Doublecortin domain-containing protein 5	DCDC5	-0.103	0.019	
Q7RTP6	Protein-methionine sulfoxide oxidase MICAL3	MICAL3	-0.102		
Q9Y4C8	Probable RNA-binding protein 19	RBM19	-0.102	-0.147	
B4DS52	Thioredoxin domain-containing protein 15	TXNDC15	-0.102		
Q8WVH0	Complexin-3	CPLX3	-0.102	0.207	
Q13610	Periodic tryptophan protein 1 homolog	PWP1	-0.101	0.030	
E7ETU6	Protein unc-45 homolog A	SMAP1	-0.101	-0.053	
K7ENW0	Gametogenetin-binding protein 2 (Fragment)	GGNBP2	-0.100		
O75882-3	Isoform 3 of Attractin	ATRN	-0.099		
A6NDG6	Phosphoglycolate phosphatase	PGP	-0.098		
P10109	Adrenodoxin, mitochondrial	FDX1	-0.097	-0.129	
Q96SB3	Neurabin-2	PPP1R9B	-0.097		
G5EA48	SLIT-ROBO Rho GTPase activating protein 1, isoform CRA_a	SRGAP1	-0.097		
F5GXP1	Alpha-2-macroglobulin-like protein 1 (Fragment)	A2ML1	-0.096		
H7BYN7	Target of Myb protein 1	TOM1	-0.096	-0.271	
C9JVY5	A-kinase anchor protein 2 (Fragment)	AKAP2	-0.095		
O60341	Lysine-specific histone demethylase 1A	KDM1A	-0.095	0.027	
E9PJV3	RNA pseudouridylate synthase domain-containing protein 4 (Fragment)	RPUSD4	-0.093		
O75843	AP-1 complex subunit gamma-like 2	AP1G2	-0.093		
Q9NWU2	Glucose-induced degradation protein 8 homolog	GID8	-0.093	-0.021	
F6W009	DNA-directed RNA polymerase II subunit RPB11-b1	POLR2J2	-0.092		
Q15773	Myeloid leukemia factor 2	MLF2	-0.092		
Q7Z6L1-3	Isoform 3 of Tectonin beta-propeller repeat-containing protein 1	TECPR1	-0.092	0.079	
Q15527	Surfeit locus protein 2	SURF2	-0.092	0.096	
O15287	Fanconi anemia group G protein	FANCG	-0.092		
B4DKB2	Endothelin-converting enzyme 1	ECE1	-0.091	0.006	
Q9NQT5	Exosome complex component RRP40	EXOSC3	-0.090	-0.141	
B1AH58	Intraflagellar transport protein 27 homolog (Fragment)	IFT27	-0.088		
Q15561-2	Isoform 2 of Transcriptional enhancer factor TEF-3	TEAD4	-0.088		
F5H6D8	N6-adenosine-methyltransferase 70 kDa subunit	METTL3	-0.088	0.057	
E7EUL7	Sperm-specific antigen 2	SSFA2	-0.087		
F5H7A2	Podocalyxin	PODXL	-0.087	0.693	
Q5VSL9-3	Isoform 3 of Striatin-interacting protein 1	STRIP1	-0.087	-0.174	
Q9BVC3	Sister chromatid cohesion protein DCC1	DSCC1	-0.087		
A8MU44	Protein Hook homolog 1	HOOK1	-0.087	-0.022	
Q9NWB6	Arginine and glutamate-rich protein 1	ARGLU1	-0.086	0.015	
Q9NV35	Probable 8-oxo-dGTP diphosphatase NUDT15	NUDT15	-0.086	-0.106	
Q9UHA4-2	Isoform 2 of Ragulator complex protein LAMTOR3	LAMTOR3	-0.085	0.121	
Q5SZX1	Protein SMG5	SMG5	-0.085		
Q96AY4	Tetratricopeptide repeat protein 28	TTC28	-0.085		
Q7LGA3-2	Isoform 2 of Heparan sulfate 2-O-sulfotransferase 1	HS2ST1	-0.084	0.010	
J3KTE1	Abhydrolase domain-containing protein 3	ABHD3	-0.084		
H0YA18	Ufm1-specific protease 2 (Fragment)	UFSP2	-0.084	0.115	
Q99543	DnaJ homolog subfamily C member 2	DNAJC2	-0.083	-0.060	
Q6IPX3-2	Isoform 2 of Transcription elongation factor A protein-like 6	TCEAL6	-0.083		
Q969X6-3	Isoform 3 of Cirhin	CIRH1A	-0.083	-0.313	
P51570	Galactokinase	GALK1	-0.083	0.063	
Q8IWC1-3	Isoform 3 of MAP7 domain-containing protein 3	MAP7D3	-0.082	-0.195	
C9JUN5	Coiled-coil domain-containing protein 12 (Fragment)	CCDC12	-0.082	-0.157	
J3KND1	Protein SAAL1	SAAL1	-0.081	-0.157	
O00754-2	Isoform 2 of Lysosomal alpha-mannosidase	MAN2B1	-0.081	-0.124	
Q9Y3D6	Mitochondrial fission 1 protein	FIS1	-0.080	0.149	
Q9NVV4	Poly(A) RNA polymerase, mitochondrial	MTPAP	-0.080		
Q9H553-2	Isoform 2 of Alpha-1,3/1,6-mannosyltransferase ALG2	ALG2	-0.080	0.100	
Q15050	Ribosome biogenesis regulatory protein homolog	RRS1	-0.079	-0.140	
E7EVM5	DDB1- and CUL4-associated factor 13	DCAF13	-0.079	-0.090	
P26440	Isovaleryl-CoA dehydrogenase, mitochondrial	IVD	-0.078		
B4DPR4	Makorin, ring finger protein, 2, isoform CRA_b	MKRN2	-0.078	-0.231	
P09234	U1 small nuclear ribonucleoprotein C	SNRPC	-0.078	-0.244	
G3V500	Echinoderm microtubule-associated protein-like 1 (Fragment)	EML1	-0.078		
H0YLG3	Gamma-tubulin complex component 5 (Fragment)	TUBGCP5	-0.077		
Q7Z6E9-2	Isoform 2 of E3 ubiquitin-protein ligase RBBP6	RBBP6	-0.077	-0.007	

Q99622	Protein C10	C12orf57	-0.077		
Q9GZT8-3	Isoform 3 of NIF3-like protein 1	NIF3L1	-0.077	0.013	
Q9Y237	Peptidyl-prolyl cis-trans isomerase NIMA-interacting 4	PIN4	-0.075	-0.260	
J3QRG6	Cyclin-dependent kinase inhibitor 2A, isoforms 1/2/3	CDKN2A	-0.075	-0.265	
P09467	Fructose-1,6-bisphosphatase 1	FBP1	-0.075		
H7C1M5	Deoxynucleotidyltransferase terminal-interacting protein 1 (Fragment)	DNTTIP1	-0.075	-0.226	
Q8TD26	Chromodomain-helicase-DNA-binding protein 6	CHD6	-0.074	-0.153	
E9PAV9	G patch domain-containing protein 4	GPATCH4	-0.074	-0.111	
O14647-2	Isoform 2 of Chromodomain-helicase-DNA-binding protein 2	CHD2	-0.074		
Q86W34	Archaemetzincin-2	AMZ2	-0.074	0.400	
H3BPR3	Sorting nexin-33 (Fragment)	SNX33	-0.074		
Q9H9E3-2	Isoform 2 of Conserved oligomeric Golgi complex subunit 4	COG4	-0.073		
P49643	DNA primase large subunit	PRIM2	-0.073	-0.035	
Q9Y6H1	Coiled-coil-helix-coiled-coil-helix domain-containing protein 2, mitochondrial	CHCHD2	-0.073	0.123	
O75828	Carbonyl reductase [NADPH] 3	CBR3	-0.072	-0.199	
O00308-2	Isoform 2 of NEDD4-like E3 ubiquitin-protein ligase WWP2	WWP2	-0.071		
P33981-2	Isoform 2 of Dual specificity protein kinase TTK	TTK	-0.071		
Q9NPI6	mRNA-decapping enzyme 1A	DCP1A	-0.071	0.277	
E5RFJ3	DBIRD complex subunit KIAA1967 (Fragment)	KIAA1967	-0.070		
K7EQP1	Zinc finger HIT domain-containing protein 3	ZNHIT3	-0.070		
O43674-2	Isoform 2 of NADH dehydrogenase [ubiquinone] 1 beta subcomplex subunit 5, mi	NDUFB5	-0.070	0.078	
Q8IWA0	WD repeat-containing protein 75	WDR75	-0.069		
M0R0H0	IgG receptor FcRn large subunit p51 (Fragment)	FCGRT	-0.069		
G3V4M9	Inositol 1,3,4-triphosphate 5/6 kinase, isoform CRA_c	ITPK1	-0.069	-0.073	
P82914	28S ribosomal protein S15, mitochondrial	MRPS15	-0.069	-0.127	
Q9Y244	Proteasome maturation protein	POMP	-0.068		
H3BUM8	Ubiquitin domain-containing protein UBFD1	UBFD1	-0.068	0.204	
E9PPA5	Serine/threonine-protein kinase Chk1 (Fragment)	CHEK1	-0.068		
O15397	Importin-8	IPO8	-0.068	0.298	
Q96EL2	28S ribosomal protein S24, mitochondrial	MRPS24	-0.068		
Q96I25	Splicing factor 45	RBM17	-0.067	-0.016	
Q9NVH2-4	Isoform 4 of Integrator complex subunit 7	INTS7	-0.067	0.042	
Q96DZ1-2	Isoform 2 of Endoplasmic reticulum lectin 1	ERLEC1	-0.067		
O75340-2	Isoform 2 of Programmed cell death protein 6	PDCD6	-0.067		
K7ES22	Histone chaperone ASF1B	ASF1B	-0.067	0.476	
Q9Y6W5	Wiskott-Aldrich syndrome protein family member 2	WASF2	-0.067		
P50224	Sulfotransferase 1A3/1A4	SULT1A3	-0.065	0.204	
J3QL56	Protein SCO1 homolog, mitochondrial	SCO1	-0.064	-0.003	
Q99611	Selenide, water dikinase 2	SEPHS2	-0.064	0.255	
Q96SI9-2	Isoform 2 of Spermatid perinuclear RNA-binding protein	STRBP	-0.063	-0.346	
Q9NXG2	THUMP domain-containing protein 1	THUMPD1	-0.063	-0.006	
Q7L2J0	7SK snRNA methylphosphate capping enzyme	MEPCE	-0.063	-0.067	
H3BPS1	Trafficking protein particle complex subunit 2-like protein	TRAPPC2L	-0.063		
G3V3Y4	Zinc finger CCCH domain-containing protein 14 (Fragment)	ZC3H14	-0.062	-0.132	
O95297-4	Isoform 4 of Myelin protein zero-like protein 1	MPZL1	-0.062	0.190	
P46939	Utrophin	UTRN	-0.062	0.270	
Q5VV41-2	Isoform 2 of Rho guanine nucleotide exchange factor 16	ARHGEF16	-0.062		
H0YJL9	LIM domain-containing protein ajuba (Fragment)	AJUBA	-0.062		
Q13555-10	Isoform 10 of Calcium/calmodulin-dependent protein kinase type II subunit gamm	CAMK2G	-0.062	-0.068	
G5E9C8	Son of sevenless homolog 1	SOS1	-0.062		
Q9ULZ3-2	Isoform 2 of Apoptosis-associated speck-like protein containing a CARD	PYCARD	-0.061		
K7EQL6	Ubiquitin carboxyl-terminal hydrolase	USP32	-0.061		
Q9H0B6-2	Isoform 2 of Kinesin light chain 2	KLC2	-0.061		
Q9UHK0	Nuclear fragile X mental retardation-interacting protein 1	NUFIP1	-0.061		
E9PGM4	1,4-alpha-glucan-branching enzyme	GBE1	-0.061	0.053	
Q9H4A3-4	Isoform 3 of Serine/threonine-protein kinase WNK1	WNK1	-0.061	-0.175	
Q9NR33	DNA polymerase epsilon subunit 4	POLE4	-0.060		
Q96KR1	Zinc finger RNA-binding protein	ZFR	-0.060	-0.067	
Q9UEU0	Vesicle transport through interaction with t-SNAREs homolog 1B	VTI1B	-0.060		
Q03169	Tumor necrosis factor alpha-induced protein 2	TNFAIP2	-0.060	0.123	
O00425	Insulin-like growth factor 2 mRNA-binding protein 3	IGF2BP3	-0.060	0.047	
P09936	Ubiquitin carboxyl-terminal hydrolase isozyme L1	UCHL1	-0.059		
H0YH87	Ataxin-2 (Fragment)	ATXN2	-0.058	-0.321	
Q9UKV5	E3 ubiquitin-protein ligase AMFR	AMFR	-0.058		
Q9H9J2	39S ribosomal protein L44, mitochondrial	MRPL44	-0.057	0.179	
A6NG79	Proline-rich protein PRCC	PRCC	-0.057		
Q96KG9-5	Isoform 5 of N-terminal kinase-like protein	SCYL1	-0.057		
P07902-2	Isoform 2 of Galactose-1-phosphate uridylyltransferase	GALT	-0.057		
P49006	MARCKS-related protein	MARCKSL1	-0.057	0.062	
H0YM82	Uncharacterized protein C15orf52 (Fragment)	C15orf52	-0.056		
P84101-4	Isoform 4 of Small EDRK-rich factor 2	SERF2	-0.056	0.079	
O95104-2	Isoform 2 of Splicing factor, arginine/serine-rich 15	SCAF4	-0.055	0.024	
P16219	Short-chain specific acyl-CoA dehydrogenase, mitochondrial	ACADS	-0.055		
Q9H2P9-6	Isoform 6 of Diphthine synthase	DPH5	-0.053		
F8WAR6	Kinesin-like protein KIF3C	KIF3C	-0.053	-0.091	
Q86X95	Corepressor interacting with RBPJ 1	CIR1	-0.053		
Q96NE9-2	Isoform 2 of FERM domain-containing protein 6	FRMD6	-0.053		
H0Y564	Anaphase-promoting complex subunit 1 (Fragment)	ANAPC1	-0.053	0.062	
B4DQH9	Ran-binding protein 10	RANBP10	-0.053		
Q8TD19	Serine/threonine-protein kinase Nek9	NEK9	-0.053		
P16455	Methylated-DNA--protein-cysteine methyltransferase	MGMT	-0.053	0.145	
G5EA36	Cell division cycle 27, isoform CRA_c	CDC27	-0.052		
H0YMJ4	Kinesin-like protein KIF23	KIF23	-0.052	-0.184	
P23193	Transcription elongation factor A protein 1	TCEA1	-0.052	0.049	
Q9NVX2	Notchless protein homolog 1	NLE1	-0.051		
P52294	Importin subunit alpha-1	KPNA1	-0.051		
Q9NS86	LanC-like protein 2	LANCL2	-0.050	0.197	

C9J8Q5	Succinate-semialdehyde dehydrogenase, mitochondrial	ALDH5A1	-0.050		
A6PVS8	Leucine-rich repeat and IQ domain-containing protein 3	LRRIQ3	-0.050		
B4DSR5	Kinesin-like protein KIF3B	KIF3B	-0.050		
Q53HL2	Borealin	CDCA8	-0.050	-0.154	
B4DS55	Coiled-coil domain-containing protein 132	CCDC132	-0.050		
Q96G46	tRNA-dihydrouridine(47) synthase [NAD(P)(+)]-like	DUS3L	-0.050	-0.021	
Q8N9T8	Protein KRI1 homolog	KRI1	-0.050	-0.100	
O96007	Molybdopterin synthase catalytic subunit	MOCS2	-0.050		
E9PQD0	Probable RNA-binding protein EIF1AD	EIF1AD	-0.050	0.024	
Q86VS8	Protein Hook homolog 3	HOOK3	-0.049	0.059	
G5EA42	Tropomodulin 2 (Neuronal), isoform CRA_a	TMOD2	-0.048	-0.357	
Q6ZN55	Zinc finger protein 574	ZNF574	-0.048	-0.206	
O60504-2	Isoform Beta of Vinexin	SORBS3	-0.048	0.587	
Q06587	E3 ubiquitin-protein ligase RING1	RING1	-0.047	-0.086	
Q9H2K8	Serine/threonine-protein kinase TAO3	TAOK3	-0.047	0.190	
O75616	GTPase Era, mitochondrial	ERAL1	-0.047		
C9J4K0	Ashwin	C2orf49	-0.046		
Q5T1M5	FK506-binding protein 15	FKBP15	-0.046	0.175	
O95989	Diphosphoinositol polyphosphate phosphohydrolase 1	NUDT3	-0.046	-0.110	
H0Y5N2	Sphingomyelin phosphodiesterase 2 (Fragment)	SMPD2	-0.045	-0.311	
Q9NR50-3	Isoform 3 of Translation initiation factor eIF-2B subunit gamma	EIF2B3	-0.045	0.025	
K7EPT6	TATA-binding protein-associated factor 2N	TAF15	-0.045	-0.136	
D6R9W2	Glutathione S-transferase C-terminal domain-containing protein	GSTCD	-0.045		
Q9Y5J9	Mitochondrial import inner membrane translocase subunit Tim8 B	TIMM8B	-0.044		
Q9UBW8	COP9 signalosome complex subunit 7a	COPS7A	-0.044	0.104	
O95759-2	Isoform 2 of TBC1 domain family member 8	TBC1D8	-0.044		
Q8ND04-3	Isoform 3 of Protein SMG8	SMG8	-0.044	-0.051	
P18084	Integrin beta-5	ITGB5	-0.044	-0.032	
Q8IVH2-3	Isoform 3 of Forkhead box protein P4	FOXP4	-0.044		
Q9H8H2-4	Isoform 4 of Probable ATP-dependent RNA helicase DDX31	DDX31	-0.044		
Q8TB03-3	Isoform 3 of Uncharacterized protein CXorf38	CXorf38	-0.044		
Q9NVR5-2	Isoform 2 of Protein kintoun	DNAAF2	-0.044		
B3KQZ9	Tumor necrosis factor receptor type 1-associated DEATH domain protein	TRADD	-0.043		
Q92636	Protein FAN	NSMAF	-0.043	0.066	
H3BMM5	Uncharacterized protein	4	-0.043		
Q8NEP3-3	Isoform 3 of Dynein assembly factor 1, axonemal	DNAAF1	-0.043		
Q9Y6K0	Choline/ethanolaminephosphotransferase 1	CEPT1	-0.043	0.078	
Q15124-2	Isoform 2 of Phosphoglucomutase-like protein 5	PGM5	-0.043	0.031	
E9PHK0	Tetranectin	CLEC3B	-0.042		
J3QS48	Mannose-P-dolichol utilization defect 1 protein	MPDU1	-0.042		
P49770	Translation initiation factor eIF-2B subunit beta	EIF2B2	-0.042	0.062	
H3BTB7	Probable glutamate--tRNA ligase, mitochondrial	EARS2	-0.042		
Q6P1X5	Transcription initiation factor TFIID subunit 2	TAF2	-0.042		
H0YNN4	Complex I intermediate-associated protein 30, mitochondrial (Fragment)	NDUFAF1	-0.042		
P84085	ADP-ribosylation factor 5	ARF5	-0.041	0.111	
E9PKT9	Molybdopterin synthase sulfur carrier subunit	MOCS2	-0.041		
Q6PD62	RNA polymerase-associated protein CTR9 homolog	CTR9	-0.041	-0.044	
P01116-2	Isoform 2B of GTPase KRas	KRAS	-0.040		
Q5SWX8-3	Isoform 3 of Protein odr-4 homolog	ODR4	-0.040	0.295	
B4DL79	Kinesin-like protein KIF20A	KIF20A	-0.040		
Q9UIG0-2	Isoform 2 of Tyrosine-protein kinase BAZ1B	BAZ1B	-0.040	-0.277	
P18583-7	Isoform G of Protein SON	SON	-0.040	-0.192	
H3BNT2	Ubiquinone biosynthesis protein COQ9, mitochondrial (Fragment)	COQ9	-0.039	0.219	
Q9GZL7	Ribosome biogenesis protein WDR12	WDR12	-0.039	-0.173	
Q9UJU6	Drebrin-like protein	DBNL	-0.039	0.024	
J3QS03	Twisted gastrulation protein homolog 1	TWSG1	-0.039		
F5H2B9	Uveal autoantigen with coiled-coil domains and ankyrin repeats	UACA	-0.039	-0.007	
P55212	Caspase-6	CASP6	-0.039	-0.126	
Q9H3H5-3	Isoform 3 of UDP-N-acetylglucosamine--dolichyl-phosphate N-acetylglucosaminepl	DPAGT1	-0.038	0.273	
Q9Y294	Histone chaperone ASF1A	ASF1A	-0.038	-0.340	
Q6P1X6	UPF0598 protein C8orf82	C8orf82	-0.038	0.054	
Q9UJW0	Dynactin subunit 4	DCTN4	-0.037	0.064	
Q96KM6	Zinc finger protein 512B	ZNF512B	-0.037	0.034	
P46100-2	Isoform 1 of Transcriptional regulator ATRX	ATRX	-0.037	-0.128	
C9J1G2	DnaJ homolog subfamily B member 2 (Fragment)	DNAJB2	-0.037	0.008	
Q8IYB3-2	Isoform 2 of Serine/arginine repetitive matrix protein 1	SRRM1	-0.037	0.023	
F8VRZ8	Sentrin-specific protease 1 (Fragment)	SENP1	-0.036		
H0Y9P4	Androgen-dependent TFPI-regulating protein (Fragment)	ADTRP	-0.035		
E7EMP6	UPF0361 protein C3orf37	C3orf37	-0.034	0.008	
P50895	Basal cell adhesion molecule	BCAM	-0.034		
Q5VTR2	E3 ubiquitin-protein ligase BRE1A	RNF20	-0.033	0.081	
Q8N556	Actin filament-associated protein 1	AFAP1	-0.033		
P02545	Prelamin-A/C	LMNA	-0.033	0.041	
Q14344	Guanine nucleotide-binding protein subunit alpha-13	GNA13	-0.032	0.051	
Q9HB21	Pleckstrin homology domain-containing family A member 1	PLEKHA1	-0.032		
Q11201	CMP-N-acetylneuraminate-beta-galactosamide-alpha-2,3-sialyltransferase 1	ST3GAL1	-0.032		
Q9Y5Y6	Suppressor of tumorigenicity 14 protein	ST14	-0.032		
P51970	NADH dehydrogenase [ubiquinone] 1 alpha subcomplex subunit 8	NDUFA8	-0.032	0.015	
O60930	Ribonuclease H1	RNASEH1	-0.032		
Q99653	Calcineurin B homologous protein 1	CHP1	-0.031	0.003	
Q9NRF8	CTP synthase 2	CTPS2	-0.031	-0.052	
M0QY77	KxDL motif-containing protein 1	KXD1	-0.031		
P42575	Caspase-2	CASP2	-0.031	0.522	
E7ERH3	Tensin-3	TNS3	-0.031	0.118	
Q12899	Tripartite motif-containing protein 26	TRIM26	-0.031	-0.147	
Q9NRV9	Heme-binding protein 1	HEBP1	-0.030		
Q96C01	Protein FAM136A	FAM136A	-0.030	-0.196	

O43240	Kallikrein-10	KLK10	-0.030		
Q9NWH9	SAFB-like transcription modulator	SLTM	-0.029	-0.245	
Q6P1L8	39S ribosomal protein L14, mitochondrial	MRPL14	-0.029	-0.233	
Q9UNE7	E3 ubiquitin-protein ligase CHIP	STUB1	-0.029	-0.039	
Q9H089	Large subunit GTPase 1 homolog	LSG1	-0.029		
Q9NP72	Ras-related protein Rab-18	RAB18	-0.029	0.151	
B4DJ23	Myotubularin-related protein 14	MTMR14	-0.028	0.005	
Q6P6C2-3	Isoform 3 of RNA demethylase ALKBH5	ALKBH5	-0.028	0.102	
Q9BY89	Uncharacterized protein KIAA1671	KIAA1671	-0.028		
Q8TB36-2	Isoform 2 of Ganglioside-induced differentiation-associated protein 1	GDAP1	-0.028		
Q8NEZ4-2	Isoform 2 of Histone-lysine N-methyltransferase MLL3	MLL3	-0.028		
P10586-2	Isoform 2 of Receptor-type tyrosine-protein phosphatase F	PTPRF	-0.028		
A8MWR2	WAS/WASL-interacting protein family member 2	WIPF2	-0.028		
O43402	ER membrane protein complex subunit 8	EMC8	-0.027		
Q9P2X0	Dolichol-phosphate mannosyltransferase subunit 3	DPM3	-0.027	0.130	
Q96AC5	ETS1 protein	ETS1	-0.027	0.020	
Q9BSH5	Haloacid dehalogenase-like hydrolase domain-containing protein 3	HDHD3	-0.027	-0.191	
P16383-2	Isoform 2 of GC-rich sequence DNA-binding factor 2	GCFC2	-0.027		
O60826	Coiled-coil domain-containing protein 22	CCDC22	-0.027	-0.037	
E7EW18	DNA polymerase beta (Fragment)	POLB	-0.027	0.052	
Q16513-3	Isoform 3 of Serine/threonine-protein kinase N2	PKN2	-0.026	-0.211	
Q96QR8	Transcriptional activator protein Pur-beta	PURB	-0.026	0.019	
Q86VP1-3	Isoform 3 of Tax1-binding protein 1	TAX1BP1	-0.026		
Q8IZ07	Ankyrin repeat domain-containing protein 13A	ANKRD13A	-0.026		
Q9NXC5	WD repeat-containing protein mio	MIOS	-0.026		
Q13823	Nucleolar GTP-binding protein 2	GNL2	-0.025	-0.511	
B7WPF4	Ubiquitin carboxyl-terminal hydrolase	USP24	-0.025	0.127	
Q9Y5L4	Mitochondrial import inner membrane translocase subunit Tim13	TIMM13	-0.025	0.058	
Q14320	Protein FAM50A	FAM50A	-0.025	0.040	
Q9HB07	UPF0160 protein MYG1, mitochondrial	C12orf10	-0.025	0.021	
H0YBW3	Integrator complex subunit 10 (Fragment)	INTS10	-0.025	0.172	
P62987	Ubiquitin-60S ribosomal protein L40	UBA52	-0.024	0.353	
A8MZH8	Pituitary tumor-transforming gene 1 protein-interacting protein	PTTG1IP	-0.024	0.304	
O43708-3	Isoform 3 of Maleylacetoacetate isomerase	GSTZ1	-0.023	0.211	
Q9NUQ6-2	Isoform 2 of SPATS2-like protein	SPATS2L	-0.023		
F5GZ06	Maestro heat-like repeat-containing protein family member 2B	MROH2B	-0.023	0.171	
Q8NDV3-2	Isoform 2 of Structural maintenance of chromosomes protein 1B	SMC1B	-0.023	0.201	
Q8IVS2	Malonyl-CoA-acyl carrier protein transacylase, mitochondrial	MCAT	-0.023		
Q15814	Tubulin-specific chaperone C	TBCC	-0.022		
O95865	N(G),N(G)-dimethylarginine dimethylaminohydrolase 2	DDAH2	-0.022	-0.023	
Q5T0N5-3	Isoform 3 of Formin-binding protein 1-like	FNBP1L	-0.022		
Q9BS40	Latexin	LXN	-0.021		
E9PQF6	Ribosomal protein S6 kinase beta-2 (Fragment)	RPS6KB2	-0.021		
O60231	Putative pre-mRNA-splicing factor ATP-dependent RNA helicase DHX16	DHX16	-0.021	-0.041	
Q6IN84	rRNA methyltransferase 1, mitochondrial	MRM1	-0.021		
H0Y9L8	Bifunctional lysine-specific demethylase and histidyl-hydroxylase MINA (Fragment)	MINA	-0.021		
K7ERN1	E3 ubiquitin-protein ligase NEDD4-like (Fragment)	NEDD4L	-0.020		
Q9GZR2-2	Isoform 2 of RNA exonuclease 4	REXO4	-0.020		
Q8WXI4-2	Isoform 2 of Acyl-coenzyme A thioesterase 11	ACOT11	-0.019		
O60518	Ran-binding protein 6	RANBP6	-0.018	0.013	
H0YGV7	Endoplasmic reticulum mannosyl-oligosaccharide 1,2-alpha-mannosidase (Fragme	MAN1B1	-0.018		
Q9H0H5	Rac GTPase-activating protein 1	RACGAP1	-0.017	-0.124	
Q8N6N7	Acyl-CoA-binding domain-containing protein 7	ACBD7	-0.017	-0.376	
Q9H967	WD repeat-containing protein 76	WDR76	-0.017	-0.002	
O00204-2	Isoform 2 of Sulfotransferase family cytosolic 2B member 1	SULT2B1	-0.017		
Q13049	E3 ubiquitin-protein ligase TRIM32	TRIM32	-0.017		
Q8NEY1-5	Isoform 5 of Neuron navigator 1	NAV1	-0.017		
Q8TCU4-3	Isoform 3 of Alstrom syndrome protein 1	ALMS1	-0.017	0.138	
K7EIF9	Signal transducer and activator of transcription 5A (Fragment)	STAT5A	-0.017		
Q96CV9-3	Isoform 3 of Optineurin	OPTN	-0.017	0.162	
Q13131	5'-AMP-activated protein kinase catalytic subunit alpha-1	PRKAA1	-0.016		
E9PND3	Receptor-binding cancer antigen-expressed on SiSo cells	EBAG9	-0.016	0.235	
Q9H0V9	VIP36-like protein	LMAN2L	-0.015		
Q06546	GA-binding protein alpha chain	GABPA	-0.015	-0.089	
Q96EU6-2	Isoform 2 of Ribosomal RNA processing protein 36 homolog	RRP36	-0.015		
F8W6A4	Kelch domain-containing protein 3	KLHDC3	-0.015		
Q92536	Y+L amino acid transporter 2	SLC7A6	-0.014		
Q96C24	Synaptotagmin-like protein 4	SYTL4	-0.014		
Q04637-5	Isoform D of Eukaryotic translation initiation factor 4 gamma 1	EIF4G1	-0.014		
F5H2J3	Vacuole membrane protein 1	VMP1	-0.014	-0.049	
P36507	Dual specificity mitogen-activated protein kinase kinase 2	MAP2K2	-0.013	0.088	
C9JQD4	Peptidyl-prolyl cis-trans isomerase (Fragment)	PPIH	-0.013	-0.163	
Q9H7D0	Dedicator of cytokinesis protein 5	DOCK5	-0.013		
Q86X76-2	Isoform 1 of Nitrilase homolog 1	NIT1	-0.013	0.099	
K7ELS8	Synaptogyrin-2 (Fragment)	SYNGR2	-0.013	0.170	
Q5VV42	Threonylcarbamoyladenosine tRNA methylthiotransferase	CDKAL1	-0.013		
Q5T0B9	Zinc finger protein 362	ZNF362	-0.012		
H3BNU0	Prickle-like protein 2	PRICKLE2	-0.012	0.007	
Q8NI35-4	Isoform 4 of InaD-like protein	INADL	-0.012	-0.006	
G8JLD3	ELKS/Rab6-interacting/CAST family member 1	ERC1	-0.012		
C9J0I5	Solute carrier family 12 member 9 (Fragment)	SLC12A9	-0.012		
H0YL91	Activating signal cointegrator 1	TRIP4	-0.011		
O75400-2	Isoform 2 of Pre-mRNA-processing factor 40 homolog A	PRPF40A	-0.011	-0.001	
C9J8Q1	NFU1 iron-sulfur cluster scaffold homolog, mitochondrial (Fragment)	NFU1	-0.011	-0.017	
Q5BKZ1	DBIRD complex subunit ZNF326	ZNF326	-0.011	-0.018	
Q9Y5J1	U3 small nucleolar RNA-associated protein 18 homolog	UTP18	-0.010	-0.030	
Q7L775	EPM2A-interacting protein 1	EPM2AIP1	-0.010		

E9PKE4	Solute carrier family 52, riboflavin transporter, member 2 (Fragment)	SLC52A2	-0.010		
B7ZC39	Endophilin-B2	SH3GLB2	-0.010	-0.156	
Q8ND24	RING finger protein 214	RNF214	-0.010		
P25490	Transcriptional repressor protein YY1	YY1	-0.009	-1.337	
B4DQA8	Golgi SNAP receptor complex member 1	GOSR1	-0.009		
E7EM96	Ras and Rab interactor 1	RIN1	-0.008		
O60885	Bromodomain-containing protein 4	BRD4	-0.008	0.014	
Q9NQZ5	StAR-related lipid transfer protein 7, mitochondrial	STARD7	-0.008	0.003	
Q7Z5G4-3	Isoform 2 of Golgin subfamily A member 7	GOLGA7	-0.007		
P17480-2	Isoform UBF2 of Nucleolar transcription factor 1	UBTF	-0.007	-0.319	
Q99735-2	Isoform 2 of Microsomal glutathione S-transferase 2	MGST2	-0.007		
E9PM91	DNA polymerase delta subunit 3 (Fragment)	POLD3	-0.007		
Q8NBX0	Saccharopine dehydrogenase-like oxidoreductase	SCCPDH	-0.006	-0.093	
B4DG44	Alpha- and gamma-adaptin-binding protein p34	AAGAB	-0.006		
D3DRR9	Chromosome 10 open reading frame 47, isoform CRA_b	C10orf47	-0.006		
Q96EI5	Transcription elongation factor A protein-like 4	TCEAL4	-0.006	0.131	
E9PI87	Oxidoreductase HTATIP2	HTATIP2	-0.006	0.312	
Q8WYQ3	Coiled-coil-helix-coiled-coil-helix domain-containing protein 10, mitochondrial	CHCHD10	-0.006		
K7EK00	Protein FAM210A (Fragment)	FAM210A	-0.006		
Q9BVR0	Putative HERC2-like protein 3	HERC2P3	-0.005		
Q96FJ2	Dynein light chain 2, cytoplasmic	DYNLL2	-0.005	0.041	
H0YDP9	Receptor-type tyrosine-protein phosphatase kappa (Fragment)	PTPRK	-0.005		
Q9UBD5-3	Isoform 3 of Origin recognition complex subunit 3	ORC3	-0.005		
Q14BN4	Sarcolemmal membrane-associated protein	SLMAP	-0.005	0.114	
Q15149-4	Isoform 4 of Plectin	PLEC	-0.004		
P14618-2	Isoform M1 of Pyruvate kinase isozymes M1/M2	PKM	-0.003	0.067	
I3L459	Phosphatidylinositol transfer protein alpha isoform (Fragment)	PITPNA	-0.003	-0.010	
E9PPW6	Mitochondrial fission factor	MFF	-0.002	0.050	
Q8TEM1	Nuclear pore membrane glycoprotein 210	NUP210	-0.002	0.007	
C9JDZ2	Fas apoptotic inhibitory molecule 1 (Fragment)	FAIM	-0.002		
J3KSY7	Protein CASC3 (Fragment)	CASC3	-0.002	-0.140	
Q9NX46	Poly(ADP-ribose) glycohydrolase ARH3	ADPRHL2	-0.002		
Q9H7Z6	Histone acetyltransferase KAT8	KAT8	-0.002	0.041	
Q9GZU8	Protein FAM192A	FAM192A	-0.002	-0.052	
P49821-2	Isoform 2 of NADH dehydrogenase [ubiquinone] flavoprotein 1, mitochondrial	NDUFV1	-0.001	0.075	
Q8NAF0	Zinc finger protein 579	ZNF579	-0.001	-0.290	
C9JZR4	ADP-ribosylation factor GTPase-activating protein 3	ARFGAP3	-0.001	0.124	
H0YA52	Pterin-4-alpha-carbinolamine dehydratase 2 (Fragment)	PCBD2	-0.001	-0.210	
Q9P2I0	Cleavage and polyadenylation specificity factor subunit 2	CPSF2	-0.001	0.008	
Q9H0R6	Glutamyl-tRNA(Gln) amidotransferase subunit A, mitochondrial	QRSL1	-0.001	-0.146	
E5RFJ1	E3 SUMO-protein ligase NSE2	NSMCE2	-0.001		
Q9NYV4-2	Isoform 2 of Cyclin-dependent kinase 12	CDK12	-0.001		
Q13217	DnaJ homolog subfamily C member 3	DNAJC3	-0.001	0.081	
Q9NWY4	UPF0609 protein C4orf27	C4orf27	0.000	0.214	
E7EX41	ATP-dependent RNA helicase DDX55	DDX55	0.000		
H0Y8N7	39S ribosomal protein L1, mitochondrial (Fragment)	MRPL1	0.000	-0.025	
Q8TC12-2	Isoform 2 of Retinol dehydrogenase 11	RDH11	0.000	-0.296	
E9PE10	Ribonucleoprotein PTB-binding 2	RAVER2	0.001		
O00221	NF-kappa-B inhibitor epsilon	NFKBIE	0.001	-0.156	
E7EX87	Serine--tRNA ligase, mitochondrial	SARS2	0.001	0.046	
Q5T200-2	Isoform 2 of Zinc finger CCCH domain-containing protein 13	ZC3H13	0.001	-0.097	
Q9H3P2-7	Isoform 2 of Negative elongation factor A	NELFA	0.002		
Q9BX68	Histidine triad nucleotide-binding protein 2, mitochondrial	HINT2	0.002	-0.135	
Q8WWA1-2	Isoform 2 of Transmembrane protein 40	TMEM40	0.002		
O95801	Tetratricopeptide repeat protein 4	TTC4	0.002	0.029	
C9JEI7	DNA topoisomerase 3-beta-1 (Fragment)	TOP3B	0.002		
Q9BTZ2-8	Isoform 8 of Dehydrogenase/reductase SDR family member 4	DHRS4	0.002		
Q8IYN9	Focal adhesion kinase 1	PTK2	0.003		
K7EKW3	Transmembrane and ubiquitin-like domain-containing protein 2 (Fragment)	TMUB2	0.003		
Q86XI2	Condensin-2 complex subunit G2	NCAPG2	0.003	-0.012	
I3L3Z1	SHC SH2 domain-binding protein 1 (Fragment)	SHCBP1	0.004		
Q8IYB8	ATP-dependent RNA helicase SUPV3L1, mitochondrial	SUPV3L1	0.004		
Q9ULR0	Pre-mRNA-splicing factor ISY1 homolog	ISY1	0.004	-0.031	
Q9BRT8-4	Isoform 4 of COBW domain-containing protein 1	CBWD1	0.005	0.071	
Q8N3K9	Cardiomyopathy-associated protein 5	CMYA5	0.005	0.150	
E9PM62	Protein wntless homolog (Fragment)	WLS	0.005		
D6RFZ2	TBC1 domain family member 8B	TBC1D8B	0.005	-0.158	
Q8IVT2	Uncharacterized protein C19orf21	C19orf21	0.006		
Q9Y5X2	Sorting nexin-8	SNX8	0.006		
O60318	80 kDa MCM3-associated protein	MCM3AP	0.006	0.039	
Q8TEW0-9	Isoform 9 of Partitioning defective 3 homolog	PARD3	0.006		
Q9P253	Vacuolar protein sorting-associated protein 18 homolog	VPS18	0.006	-0.066	
P45973	Chromobox protein homolog 5	CBX5	0.007	-0.336	
Q9NV70-2	Isoform 2 of Exocyst complex component 1	EXOC1	0.007		
Q9Y487	V-type proton ATPase 116 kDa subunit a isoform 2	ATP6V0A2	0.008	0.133	
Q9BU23-2	Isoform 2 of Lipase maturation factor 2	LMF2	0.008	-0.010	
Q3MIX3	Uncharacterized aarF domain-containing protein kinase 5	ADCK5	0.008		
F2Z2T2	DNA repair protein-complementing XP-A cells	XPA	0.008	-0.126	
Q9UHR6	Zinc finger HIT domain-containing protein 2	ZNHIT2	0.008		
O75629	Protein CREG1	CREG1	0.009	0.035	
B4DFL2	Isocitrate dehydrogenase [NADP]	IDH2	0.009	0.018	
P35610	Sterol O-acyltransferase 1	SOAT1	0.010	0.172	
Q92805	Golgin subfamily A member 1	GOLGA1	0.010		
Q9BZ23-2	Isoform 3 of Pantothenate kinase 2, mitochondrial	PANK2	0.010		
F5GYV5	ADP-ribosylation factor-like protein 6-interacting protein 4 (Fragment)	ARL6IP4	0.010	0.358	
Q9BXB4	Oxysterol-binding protein-related protein 11	OSBPL11	0.010	-0.007	
F5H4R4	mRNA-decapping enzyme 1B	DCP1B	0.011	0.008	

Q9BZ29-3	Isoform 3 of Dedicator of cytokinesis protein 9	DOCK9	0.012		
A6NNK5	Tumor suppressor p53-binding protein 1	TP53BP1	0.012		
Q8WUD1	Ras-related protein Rab-2B	RAB2B	0.012	0.052	
Q9NVH6-5	Isoform 5 of Trimethyllysine dioxygenase, mitochondrial	TMLHE	0.012	0.070	
Q3KQU3-4	Isoform 4 of MAP7 domain-containing protein 1	MAP7D1	0.012		
Q9BQ61	Uncharacterized protein C19orf43	C19orf43	0.012	-0.099	
H3BTU3	Transcription factor 25 (Fragment)	TCF25	0.012	0.309	
F8WF45	TATA element modulatory factor	TMF1	0.012		
I3L4X3	NF-kappa-B inhibitor beta (Fragment)	NFKBIB	0.013		
H7C3G7	CWF19-like protein 2 (Fragment)	CWF19L2	0.013	-0.328	
F8W0R1	Endoplasmic reticulum-Golgi intermediate compartment protein 2 (Fragment)	ERGIC2	0.013		
B7Z3K3	Inositol-3-phosphate synthase 1	ISYNA1	0.013	0.081	
E9PQP3	ADP-ribosylation factor GTPase-activating protein 2 (Fragment)	ARFGAP2	0.013	-0.027	
F8W9B8	Exocyst complex component 5	EXOC5	0.013		
B4DKC7	GPI ethanolamine phosphate transferase 2	PIGG	0.014		
Q6ZUM4-2	Isoform 2 of Rho GTPase-activating protein 27	ARHGAP27	0.014		
Q92615	La-related protein 4B	LARP4B	0.014	0.058	
H7C155	RAF proto-oncogene serine/threonine-protein kinase (Fragment)	RAF1	0.015		
Q9NVU0-3	Isoform 3 of DNA-directed RNA polymerase III subunit RPC5	POLR3E	0.015		
Q8NBT2	Kinetochore protein Spc24	SPC24	0.015	-0.390	
P11388	DNA topoisomerase 2-alpha	TOP2A	0.015	-1.317	
Q53HC9	Protein TSSC1	TSSC1	0.016		
C9JEX3	Myotubularin-related protein 2 (Fragment)	MTMR2	0.016		
G3V2R9	Uncharacterized protein	2	0.016		
O14646-2	Isoform 2 of Chromodomain-helicase-DNA-binding protein 1	CHD1	0.016		
H3BS73	UPF0585 protein C16orf13	C16orf13	0.017		
Q16514-2	Isoform TAFII15 of Transcription initiation factor TFIID subunit 12	TAF12	0.017	-0.145	
Q9NX70	Mediator of RNA polymerase II transcription subunit 29	MED29	0.017		
O14802	DNA-directed RNA polymerase III subunit RPC1	POLR3A	0.017	-0.171	
Q9NZD8-2	Isoform 2 of Maspardin	SPG21	0.017	0.171	
Q9UL15	BAG family molecular chaperone regulator 5	BAG5	0.017	0.095	
Q96T51-2	Isoform 2 of RUN and FYVE domain-containing protein 1	RUFY1	0.017	-0.033	
P49841	Glycogen synthase kinase-3 beta	GSK3B	0.017	-0.022	
E7EP00	Protein transport protein Sec24C	SEC24C	0.017	0.004	
B7Z821	DNA mismatch repair protein Mlh1	MLH1	0.018		
Q9H4L7	SWI/SNF-related matrix-associated actin-dependent regulator of chromatin subfar	SMARCAD1	0.019	0.203	
I3L0N3	Vesicle-fusing ATPase	NSF	0.019	0.047	
Q96BW5-2	Isoform 2 of Phosphotriesterase-related protein	PTER	0.019		
M0QXH0	Thioredoxin, mitochondrial	TXN2	0.019	0.129	
P16989	Y-box-binding protein 3	YBX3	0.020	-0.147	
Q9UPN7	Serine/threonine-protein phosphatase 6 regulatory subunit 1	PPP6R1	0.020	-0.109	
P62312	U6 snRNA-associated Sm-like protein LSm6	LSM6	0.021	-0.259	
H7C4M1	Kinesin light chain 4 (Fragment)	KLC4	0.021		
O95298-2	Isoform 4 of NADH dehydrogenase [ubiquinone] 1 subunit C2	NDUFC2	0.021	0.089	
Q9Y2U5	Mitogen-activated protein kinase kinase kinase 2	MAP3K2	0.021	0.046	
I3L1K7	Golgi SNAP receptor complex member 2	GOSR2	0.021		
Q12929	Epidermal growth factor receptor kinase substrate 8	EPS8	0.021		
P52298	Nuclear cap-binding protein subunit 2	NCBP2	0.021	-0.569	
O95302	Peptidyl-prolyl cis-trans isomerase FKBP9	FKBP9	0.022	0.178	
B4DIL8	Tuberin	TSC2	0.022	-0.156	
Q8WYP5	Protein ELYS	AHCTF1	0.022	0.053	
F6U1T9	Calcineurin subunit B type 1	PPP3R1	0.022	-0.145	
J3KS15	Peptidyl-tRNA hydrolase ICT1, mitochondrial (Fragment)	ICT1	0.022	-0.123	
F5H2G6	Acyl-CoA synthetase family member 3, mitochondrial	ACSF3	0.022		
C9J8U1	Cytospin-A (Fragment)	SPECC1L	0.022	0.058	
Q9BU89	Deoxyhypusine hydroxylase	DOHH	0.023	-0.104	
Q8TCC3-3	Isoform 3 of 39S ribosomal protein L30, mitochondrial	MRPL30	0.023	0.102	
Q15398-1	Isoform 2 of Disks large-associated protein 5	DLGAP5	0.023		
M0R1I5	Deoxyhypusine synthase	DHPS	0.023	-0.002	
Q96Q11-2	Isoform 2 of CCA tRNA nucleotidyltransferase 1, mitochondrial	TRNT1	0.023		
P49711	Transcriptional repressor CTCF	CTCF	0.023	-0.280	
Q9HBH5	Retinol dehydrogenase 14	RDH14	0.024	-0.001	
Q10469	Alpha-1,6-mannosyl-glycoprotein 2-beta-N-acetylglucosaminyltransferase	MGAT2	0.024		
M0R3C8	Bifunctional polynucleotide phosphatase/kinase	PNKP	0.024		
Q6GMV2	SET and MYND domain-containing protein 5	SMYD5	0.024		
F5GZ28	DNA ligase	LIG1	0.025	-0.044	
Q9BRP1	Programmed cell death protein 2-like	PDCD2L	0.025	0.006	
Q8IWV8-2	Isoform 2 of E3 ubiquitin-protein ligase UBR2	UBR2	0.025		
P51116	Fragile X mental retardation syndrome-related protein 2	FXR2	0.025	0.002	
Q8IVQ6	Palmitoyltransferase ZDHHC21	ZDHHC21	0.025		
Q9HBM1	Kinetochore protein Spc25	SPC25	0.026	0.100	
O43719	HIV Tat-specific factor 1	HTATSF1	0.026	0.093	
Q9NRR5	Ubiquilin-4	UBQLN4	0.026	-0.058	
H0YBE0	Protein LYRIC (Fragment)	MTDH	0.027	-0.035	
H0Y5G7	Neuroblastoma-amplified sequence (Fragment)	NBAS	0.027	-0.268	
Q14802	FXYD domain-containing ion transport regulator 3	FXYD3	0.028		
P04920-2	Isoform B1 of Anion exchange protein 2	SLC4A2	0.028	-0.156	
P23634-7	Isoform ZB of Plasma membrane calcium-transporting ATPase 4	ATP2B4	0.028	0.065	
Q9H270	Vacuolar protein sorting-associated protein 11 homolog	VPS11	0.028	-0.146	
Q9Y4X0-4	Isoform 4 of AMME syndrome candidate gene 1 protein	AMMECR1	0.029		
C9JQ42	Glycogenin-1 (Fragment)	GYG1	0.029	0.115	
H9KVC6	UAP56-interacting factor (Fragment)	FYTTD1	0.029		
Q9NPJ6-2	Isoform 2 of Mediator of RNA polymerase II transcription subunit 4	MED4	0.029	0.050	
Q9H814	Phosphorylated adapter RNA export protein	PHAX	0.030	-0.079	
P62699	Protein yippee-like 5	YPEL5	0.030		
P25098	Beta-adrenergic receptor kinase 1	ADRBK1	0.030	0.112	
Q8IXT5	RNA-binding protein 12B	RBM12B	0.030	0.274	

Q14332	Frizzled-2	FZD2	0.031		
Q14318	Peptidyl-prolyl cis-trans isomerase FKBP8	FKBP8	0.031	0.073	
E7ERU0	Dystonin	DST	0.031		
C9J2Q2	ATP synthase mitochondrial F1 complex assembly factor 2 (Fragment)	ATPAF2	0.031		
P53350	Serine/threonine-protein kinase PLK1	PLK1	0.031	-0.071	
P50748	Kinetochore-associated protein 1	KNTC1	0.031	0.215	
F5H2U1	Cystathionine beta-synthase	CBS	0.032	-0.047	
E9PJU8	Ester hydrolase C11orf54 (Fragment)	C11orf54	0.032	-0.204	
Q9P206	Uncharacterized protein KIAA1522	KIAA1522	0.032		
O75312	Zinc finger protein ZPR1	ZNF259	0.032		
B7Z7A3	Nucleolar GTP-binding protein 1	GTPBP4	0.032	-0.129	
Q9NRF9	DNA polymerase epsilon subunit 3	POLE3	0.033	0.088	
Q13136-2	Isoform 2 of Liprin-alpha-1	PPFIA1	0.033	0.145	
Q9P0U4	CpG-binding protein	CXXC1	0.034	-0.087	
P52735-3	Isoform 3 of Guanine nucleotide exchange factor VAV2	VAV2	0.034		
B7ZM82	Protein Wiz	WIZ	0.034	0.015	
Q9NUB1	Acetyl-coenzyme A synthetase 2-like, mitochondrial	ACSS1	0.035		
J3KNC2	N-alpha-acetyltransferase 30 (Fragment)	NAA30	0.036		
C9JAB9	Cytoplasmic protein NCK1 (Fragment)	NCK1	0.036		
F5H2V5	Fatty acid desaturase 3	FADS3	0.036		
E9PMG4	Telomerase Cajal body protein 1	WRAP53	0.036	-0.025	
K7EJQ7	Signal peptidase complex catalytic subunit SEC11C	SEC11C	0.037	-0.139	
Q96HW7	Integrator complex subunit 4	INTS4	0.037	-0.011	
P57735	Ras-related protein Rab-25	RAB25	0.037		
K7ENL9	Uncharacterized protein C18orf8	C18orf8	0.038		
P12830	Cadherin-1	CDH1	0.038		
O15127	Secretory carrier-associated membrane protein 2	SCAMP2	0.038	0.270	
Q8WUW1	Protein BRICK1	BRK1	0.038		
F5GX07	Oligoribonuclease, mitochondrial	REXO2	0.039	0.174	
O94804	Serine/threonine-protein kinase 10	STK10	0.039	0.119	
Q9NVM9	Protein asunder homolog	ASUN	0.039	-0.122	
Q8WVC0	RNA polymerase-associated protein LEO1	LEO1	0.039		
Q9H9A6	Leucine-rich repeat-containing protein 40	LRRC40	0.039	-0.197	
O60524-3	Isoform 3 of Nuclear export mediator factor NEMF	NEMF	0.039	-0.241	
E9PPG8	Protein Hikeshi	C11orf73	0.039		
O43290	U4/U6.U5 tri-snRNP-associated protein 1	SART1	0.040	0.088	
Q7Z4H7-2	Isoform 2 of HAUS augmin-like complex subunit 6	HAUS6	0.040	0.263	
O75369-2	Isoform 2 of Filamin-B	FLNB	0.040		
D3DNV8	Leprecan-like 1, isoform CRA_b	LEPREL1	0.040	0.243	
Q13617	Cullin-2	CUL2	0.040	0.019	
A2ABK4	Negative elongation factor E (Fragment)	NELFE	0.040		
Q9BXW6	Oxysterol-binding protein-related protein 1	OSBPL1A	0.041		
Q9UJF2	Ras GTPase-activating protein nGAP	RASAL2	0.041	0.201	
Q9Y281	Cofilin-2	CFL2	0.041		
O14578-3	Isoform 3 of Citron Rho-interacting kinase	CIT	0.041	-0.045	
O76024	Wolframin	WFS1	0.041		
E7EX73	Eukaryotic translation initiation factor 4 gamma 1	EIF4G1	0.041	-0.042	
Q5T7U1	General transcription factor 3C polypeptide 5	GTF3C5	0.041	-0.047	
Q56P03	E2F-associated phosphoprotein	EAPP	0.042		
Q96D71-2	Isoform 2 of RalBP1-associated Eps domain-containing protein 1	REPS1	0.042	0.002	
Q5T1W1	Probable ATP-dependent RNA helicase DDX59 (Fragment)	DDX59	0.042	-0.098	
Q9NW64	Pre-mRNA-splicing factor RBM22	RBM22	0.042	-0.266	
E9PE01	Unconventional myosin-Vc	MYO5C	0.043		
P49757-3	Isoform 3 of Protein numb homolog	NUMB	0.043	-0.057	
O95714	E3 ubiquitin-protein ligase HERC2	HERC2	0.043		
Q14155-1	Isoform 1 of Rho guanine nucleotide exchange factor 7	ARHGEF7	0.043	-0.111	
E9PS41	WD repeat-containing protein 74	WDR74	0.043	0.023	
Q96BH1	E3 ubiquitin-protein ligase RNF25	RNF25	0.044		
Q9Y4C2	Protein FAM115A	FAM115A	0.044		
O95466	Formin-like protein 1	FMNL1	0.044		
B7ZAS0	Ethanolamine-phosphate cytidylyltransferase	PCYT2	0.044	0.147	
Q14118	Dystroglycan	DAG1	0.045	0.133	
Q96T88	E3 ubiquitin-protein ligase UHRF1	UHRF1	0.046	-0.120	
Q7LBR1	Charged multivesicular body protein 1b	CHMP1B	0.046	0.040	
Q9P2N5	RNA-binding protein 27	RBM27	0.046	0.061	
A3KN83-3	Isoform 3 of Protein strawberry notch homolog 1	SBNO1	0.046	-0.032	
K7EIQ7	Mitochondrial Rho GTPase 1 (Fragment)	RHOT1	0.046		
Q5T8C6	Cell division cycle protein 16 homolog	CDC16	0.046	-0.001	
Q9H4H8	Protein FAM83D	FAM83D	0.046		
Q96EK9	Protein KTI12 homolog	KTI12	0.047	-0.068	
Q99590-2	Isoform 2 of Protein SCAF11	SCAF11	0.047	-0.059	
H0Y6J1	M-phase phosphoprotein 8 (Fragment)	MPHOSPH8	0.048	-0.058	
E9PSH0	CUGBP Elav-like family member 1 (Fragment)	CELF1	0.048		
O15258	Protein RER1	RER1	0.048		
Q5JW57	Kinetochore-associated protein DSN1 homolog (Fragment)	DSN1	0.049	0.094	
B7ZLW7	MTR protein	MTR	0.049		
O75150	E3 ubiquitin-protein ligase BRE1B	RNF40	0.049	-0.012	
Q96CW5-2	Isoform 2 of Gamma-tubulin complex component 3	TUBGCP3	0.050	0.043	
Q7Z417	Nuclear fragile X mental retardation-interacting protein 2	NUFIP2	0.050	-0.179	
Q9Y3A6	Transmembrane emp24 domain-containing protein 5	TMED5	0.050	0.041	
E9PG35	Methylcrotonoyl-CoA carboxylase subunit alpha, mitochondrial (Fragment)	MCCC1	0.050	0.153	
H3BPK3	Hydroxyacylglutathione hydrolase, mitochondrial (Fragment)	HAGH	0.051	-0.346	
Q9BSF4	Uncharacterized protein C19orf52	C19orf52	0.051	-0.052	
E9PNW8	Fatty acyl-CoA reductase 1 (Fragment)	FAR1	0.051		
K7EKZ2	Uncharacterized protein (Fragment)	4	0.051		
O43149	Zinc finger ZZ-type and EF-hand domain-containing protein 1	ZZEF1	0.051	0.136	
Q9UL54-2	Isoform 2 of Serine/threonine-protein kinase TAO2	TAOK2	0.052	-0.128	

P05549-2	Isoform 4 of Transcription factor AP-2-alpha	TFAP2A	0.052		
Q9BTL3	RNMT-activating mini protein	FAM103A1	0.054	0.233	
Q96KN1	Protein FAM84B	FAM84B	0.054		
Q9NP77	RNA polymerase II subunit A C-terminal domain phosphatase SSU72	SSU72	0.054	-0.163	
H0YJ97	Thyroid receptor-interacting protein 11 (Fragment)	TRIP11	0.055	0.160	
K7EQ71	Periplakin (Fragment)	PPL	0.055	0.033	
H0YL76	Protein CASP	CUX1	0.055	0.084	
Q5TC82-2	Isoform 2 of Probable E3 ubiquitin-protein ligase Roquin	RC3H1	0.055	0.012	
Q99747	Gamma-soluble NSF attachment protein	NAPG	0.056		
Q14691	DNA replication complex GINS protein PSF1	GINS1	0.056	-0.329	
Q5T6V5	UPF0553 protein C9orf64	C9orf64	0.056	0.126	
P24385	G1/S-specific cyclin-D1	CCND1	0.057		
Q9HCK8-2	Isoform 2 of Chromodomain-helicase-DNA-binding protein 8	CHD8	0.057		
Q8NB46	Serine/threonine-protein phosphatase 6 regulatory ankyrin repeat subunit C	ANKRD52	0.057	0.171	
J9JIC5	Protein Njmu-R1	C17orf75	0.057		
Q8NB90-3	Isoform 3 of Spermatogenesis-associated protein 5	SPATA5	0.058	-0.032	
O43819	Protein SCO2 homolog, mitochondrial	SCO2	0.058	0.165	
Q96A44	SPRY domain-containing SOCS box protein 4	SPSB4	0.058		
Q86V21	Acetoacetyl-CoA synthetase	AACS	0.058	0.078	
Q13573	SNW domain-containing protein 1	SNW1	0.058	-0.271	
F8W6A0	Periphilin-1	PPHLN1	0.059	0.108	
E5RIY9	Protein N-terminal glutamine amidohydrolase	WDYHV1	0.060	0.306	
M0QXH1	Prenylated Rab acceptor protein 1 (Fragment)	RABAC1	0.060	0.248	
Q6IQ22	Ras-related protein Rab-12	RAB12	0.061		
F8W651	Coatomer subunit zeta-1	COPZ1	0.061	0.046	
P10515	Dihydrolipoyllysine-residue acetyltransferase component of pyruvate dehydrogena	DLAT	0.061	0.079	
J3KNU8	Methylmalonate-semialdehyde dehydrogenase [acylating], mitochondrial	ALDH6A1	0.061	0.154	
C9JWG9	Williams-Beuren syndrome chromosomal region 16 protein (Fragment)	WBSCR16	0.061	0.081	
Q05193-3	Isoform 3 of Dynamin-1	DNM1	0.061	0.007	
O14672	Disintegrin and metalloproteinase domain-containing protein 10	ADAM10	0.061		
R4GNF9	Thiosulfate sulfurtransferase/rhodanese-like domain-containing protein 1	TSTD1	0.062		
Q9BY42	Protein RTF2 homolog	RTFDC1	0.062	0.018	
Q9BXS6-4	Isoform 4 of Nucleolar and spindle-associated protein 1	NUSAP1	0.062		
J3QQW9	Polycomb protein SUZ12	SUZ12	0.062	0.024	
Q9H900-2	Isoform 2 of Protein zwilch homolog	ZWILCH	0.062	-0.021	
Q14165	Malectin	MLEC	0.062	0.104	
Q16204	Coiled-coil domain-containing protein 6	CCDC6	0.062	0.125	
Q9NZC7-2	Isoform 2 of WW domain-containing oxidoreductase	WWOX	0.063		
Q8NBK3-2	Isoform 2 of Sulfatase-modifying factor 1	SUMF1	0.063		
P07311	Acylphosphatase-1	ACYP1	0.063	-0.302	
O14841	5-oxoprolinase	OPLAH	0.064		
P83111	Serine beta-lactamase-like protein LACTB, mitochondrial	LACTB	0.065		
P50226	Sulfotransferase 1A2	SULT1A2	0.065	-0.628	
Q9UQR1	Zinc finger protein 148	ZNF148	0.065		
E7EPJ7	Triple functional domain protein (Fragment)	TRIO	0.065	-0.077	
Q7Z2Z1-2	Isoform 2 of Treslin	TICRR	0.065		
K7ESE6	Glucose-6-phosphatase 3 (Fragment)	G6PC3	0.065	0.236	
Q96BQ5	Coiled-coil domain-containing protein 127	CCDC127	0.066		
Q9Y3D0	Mitotic spindle-associated MMXD complex subunit MIP18	FAM96B	0.066	0.232	
Q8TAQ2-2	Isoform 2 of SWI/SNF complex subunit SMARCC2	SMARCC2	0.066	0.061	
E9PRN7	3 beta-hydroxysteroid dehydrogenase/Delta 5-->4-isomerase type 1 (Fragment)	HSD3B1	0.066	0.129	
E7ET92	Oxysterol-binding protein	OSBPL2	0.067	0.181	
O00220	Tumor necrosis factor receptor superfamily member 10A	TNFRSF10A	0.067		
Q15059	Bromodomain-containing protein 3	BRD3	0.067		
Q9H8V3-2	Isoform 2 of Protein ECT2	ECT2	0.067	0.017	
P35251-2	Isoform 2 of Replication factor C subunit 1	RFC1	0.068		
C9JWC4	Holliday junction recognition protein (Fragment)	HJURP	0.068		
Q13425	Beta-2-syntrophin	SNTB2	0.068	0.053	
Q6P2E9	Enhancer of mRNA-decapping protein 4	EDC4	0.068	-0.008	
Q16891-2	Isoform 2 of Mitochondrial inner membrane protein	IMMT	0.068	0.072	
H0Y720	Trinucleotide repeat-containing gene 6B protein (Fragment)	TNRC6B	0.068		
J3QQM0	Cytospin-B	SPECC1	0.068		
Q9Y450	HBS1-like protein	HBS1L	0.069	0.182	
Q4KMP7	TBC1 domain family member 10B	TBC1D10B	0.069		
P20248	Cyclin-A2	CCNA2	0.069	0.053	
Q9BWU0	Kanadaptin	SLC4A1AP	0.069	0.046	
Q9Y6W3	Calpain-7	CAPN7	0.070	0.093	
E3W978	Hyaluronan mediated motility receptor	HMMR	0.070	0.036	
Q9BVG9	Phosphatidylserine synthase 2	PTDSS2	0.070	-0.132	
P07305-2	Isoform 2 of Histone H1.0	H1F0	0.070	-0.085	
B4DDZ0	Vacuolar fusion protein MON1 homolog B	MON1B	0.070		
Q96IY1	Kinetochore-associated protein NSL1 homolog	NSL1	0.070	-0.165	
H3BR29	UPF0420 protein C16orf58	C16orf58	0.071		
Q13445	Transmembrane emp24 domain-containing protein 1	TMED1	0.071	0.160	
K7EIH3	Protein unc-13 homolog D (Fragment)	UNC13D	0.071		
Q15291-2	Isoform 2 of Retinoblastoma-binding protein 5	RBBP5	0.071	-0.061	
J3QRU8	ARF GTPase-activating protein GIT1	GIT1	0.072	0.068	
Q9Y4K0	Lysyl oxidase homolog 2	LOXL2	0.072		
Q9Y5M8	Signal recognition particle receptor subunit beta	SRPRB	0.073	0.117	
E9PHY8	Maestro heat-like repeat-containing protein family member 1	MROH1	0.073		
P30530-2	Isoform Short of Tyrosine-protein kinase receptor UFO	AXL	0.073		
O75446	Histone deacetylase complex subunit SAP30	SAP30	0.073	0.016	
I3L139	Ketosamine-3-kinase	FN3KRP	0.074		
Q2VIQ3	Chromosome-associated kinesin KIF4B	KIF4B	0.074		
Q96IQ9-2	Isoform 2 of Zinc finger protein 414	ZNF414	0.074	-0.273	
Q86TB9-2	Isoform 2 of Protein PAT1 homolog 1	PATL1	0.074		
D6RAW2	ADP-ribose pyrophosphatase, mitochondrial (Fragment)	NUDT9	0.074	-0.016	

H7C2J2	Sphingomyelin phosphodiesterase 4 (Fragment)	SMPD4	0.075	0.292	
Q14683	Structural maintenance of chromosomes protein 1A	SMC1A	0.075	-0.032	
P11274-2	Isoform 2 of Breakpoint cluster region protein	BCR	0.075		
Q63HN8	E3 ubiquitin-protein ligase RNF213	RNF213	0.076	-0.063	
P09496-2	Isoform Non-brain of Clathrin light chain A	CLTA	0.076	-0.219	
Q08357	Sodium-dependent phosphate transporter 2	SLC20A2	0.076		
Q9UNY4	Transcription termination factor 2	TTF2	0.076		
Q14527-2	Isoform 2 of Helicase-like transcription factor	HLTF	0.078	0.033	
P27694	Replication protein A 70 kDa DNA-binding subunit	RPA1	0.078	-0.158	
Q969S3	Zinc finger protein 622	ZNF622	0.078		
F8WEW4	Protein O-glucosyltransferase 1	POGLUT1	0.079	-0.030	
E7ESP4	Integrin alpha-2	ITGA2	0.079		
H0YC06	PWWP domain-containing protein 2A (Fragment)	PWWP2A	0.079		
P04156-2	Isoform 2 of Major prion protein	PRNP	0.080		
Q9H0G5	Nuclear speckle splicing regulatory protein 1	NSRP1	0.080	-0.082	
J3KT35	Protein FAM104A	FAM104A	0.080	-0.001	
O75935	Dynactin subunit 3	DCTN3	0.080	-0.002	
P16435	NADPH--cytochrome P450 reductase	POR	0.080	0.066	
Q14244-6	Isoform 6 of Ensconsin	MAP7	0.081		
H7C377	COMM domain-containing protein 1 (Fragment)	COMMD1	0.082	0.033	
O75530-3	Isoform 3 of Polycomb protein EED	EED	0.082		
Q96DA6-2	Isoform 2 of Mitochondrial import inner membrane translocase subunit TIM14	DNAJC19	0.082	0.076	
P50583	Bis(5'-nucleosyl)-tetraphosphatase [asymmetrical]	NUDT2	0.082		
Q6DD87	Zinc finger protein 787	ZNF787	0.083	-0.308	
F8WBE0	Cytoskeleton-associated protein 2-like	CKAP2L	0.083		
E9PGW7	Mediator of RNA polymerase II transcription subunit 22 (Fragment)	MED22	0.083		
Q9BSV6	tRNA-splicing endonuclease subunit Sen34	TSEN34	0.084	-0.513	
K7ES51	WD repeat-containing protein 7 (Fragment)	WDR7	0.084		
Q9NZT1	Calmodulin-like protein 5	CALML5	0.084		
Q8IZP0-10	Isoform 10 of Abl interactor 1	ABI1	0.085	0.078	
Q96EU7	C1GALT1-specific chaperone 1	C1GALT1C1	0.085		
J3QRG1	2-oxoglutarate and iron-dependent oxygenase domain-containing protein 3 (Fragr	OGFOD3	0.086		
Q96P11-5	Isoform 5 of Putative methyltransferase NSUN5	NSUN5	0.087	-0.065	
Q9BZJ0-2	Isoform 2 of Crooked neck-like protein 1	CRNKL1	0.087	-0.203	
F2Z329	Transmembrane protein 237	TMEM237	0.088		
Q9UPN3	Microtubule-actin cross-linking factor 1, isoforms 1/2/3/5	MACF1	0.088	0.124	
Q9Y3B9	RRP15-like protein	RRP15	0.089		
F5GXF5	Nucleosome-remodeling factor subunit BPTF (Fragment)	BPTF	0.089	-0.385	
Q7Z350	Kelch-like protein 9	DKFZp686L0695	0.090		
Q6P2C8-2	Isoform 2 of Mediator of RNA polymerase II transcription subunit 27	MED27	0.090	-0.185	
B3KSA0	Acyl-coenzyme A thioesterase 2, mitochondrial	ACOT2	0.090	0.230	
Q16762	Thiosulfate sulfurtransferase	TST	0.091		
Q96JC1-2	Isoform 2 of Vam6/Vps39-like protein	VPS39	0.091		
F5GZH2	Inversin	INVS	0.092		
P56181-2	Isoform 2 of NADH dehydrogenase [ubiquinone] flavoprotein 3, mitochondrial	NDUFV3	0.093	0.040	
A6ZKI3	Protein FAM127A	FAM127A	0.093	0.108	
B4DP08	Chitobiosyldiphosphodolichol beta-mannosyltransferase	ALG1	0.093	0.158	
B4DR92	UPF0489 protein C5orf22	C5orf22	0.093	-0.034	
B4DNC9	Iron-sulfur cluster assembly enzyme ISCU, mitochondrial	ISCU	0.093		
F8VV56	CD63 antigen	CD63	0.094	0.235	
Q9C0D5-2	Isoform 2 of Protein TANC1	TANC1	0.094		
Q86WR0	Coiled-coil domain-containing protein 25	CCDC25	0.094		
O94992	Protein HEXIM1	HEXIM1	0.094	-0.169	
Q969G5	Protein kinase C delta-binding protein	PRKCDBP	0.095		
P41440-3	Isoform 3 of Folate transporter 1	SLC19A1	0.095		
O43291	Kunitz-type protease inhibitor 2	SPINT2	0.095		
F8WFB5	Leucine-rich repeat and calponin homology domain-containing protein 3	LRCH3	0.096		
A6NIH7	Protein unc-119 homolog B	UNC119B	0.096		
Q96EC8	Protein YIPF6	YIPF6	0.096		
Q9UKM9-2	Isoform 1 of RNA-binding protein Raly	RALY	0.096	-0.388	
P13284	Gamma-interferon-inducible lysosomal thiol reductase	IFI30	0.096	0.280	
Q5VUA4	Zinc finger protein 318	ZNF318	0.096		
Q9H3G5	Probable serine carboxypeptidase CPVL	CPVL	0.096	0.235	
H0YKX0	E3 ubiquitin-protein ligase RNF31 (Fragment)	RNF31	0.097		
P78318	Immunoglobulin-binding protein 1	IGBP1	0.097		
Q9GZP4-2	Isoform 2 of PITH domain-containing protein 1	PITHD1	0.097	0.049	
C9JPH1	Golgi apparatus membrane protein TVP23 homolog C	FAM18B2	0.098		
O95149	Snurportin-1	SNUPN	0.099	-0.286	
Q9Y388	RNA-binding motif protein, X-linked 2	RBMX2	0.099		
P17706-3	Isoform 3 of Tyrosine-protein phosphatase non-receptor type 2	PTPN2	0.099	0.001	
Q8IVF7-2	Isoform 2 of Formin-like protein 3	FMNL3	0.100		
O00139-2	Isoform 2 of Kinesin-like protein KIF2A	KIF2A	0.101	-0.523	
C9JYM0	Ribonuclease P protein subunit p20 (Fragment)	POP7	0.101	-0.007	
Q9BRT9	DNA replication complex GINS protein SLD5	GINS4	0.101	0.142	
Q96JP5-2	Isoform 2 of E3 ubiquitin-protein ligase ZFP91	ZFP91	0.101	0.203	
Q8TDB6	E3 ubiquitin-protein ligase DTX3L	DTX3L	0.101	-0.451	
Q7L5N7	Lysophosphatidylcholine acyltransferase 2	LPCAT2	0.102		
P15144	Aminopeptidase N	ANPEP	0.102	0.647	
H7C5B8	NADH dehydrogenase [ubiquinone] 1 beta subcomplex subunit 2, mitochondrial (I	NDUFB2	0.102		
Q5STR5	Death domain-associated protein 6 (Fragment)	DAXX	0.103	0.094	
Q5T280	Uncharacterized protein C9orf114	C9orf114	0.103		
O95376	E3 ubiquitin-protein ligase ARIH2	ARIH2	0.103	0.067	
O95163	Elongator complex protein 1	IKBKAP	0.103	0.058	
H7C5J3	Ubiquitin carboxyl-terminal hydrolase 13 (Fragment)	USP13	0.104		
H7C0N4	Steroidogenic factor 1 (Fragment)	SF1	0.104	0.209	
C9JP01	Protein RFT1 homolog (Fragment)	RFT1	0.104	0.002	
F5GZU5	Mediator of RNA polymerase II transcription subunit 15	MED15	0.105	-0.139	

F8VUA2	Charged multivesicular body protein 1a	CHMP1A	0.105	0.038	
H7C5E4	5'-3' exoribonuclease 1 (Fragment)	XRN1	0.105		
Q9UMY1	Nucleolar protein 7	NOL7	0.105	-0.085	
Q5T6H7	Xaa-Pro aminopeptidase 1	XPNPEP1	0.105	0.036	
Q8WWV3-3	Isoform 3 of Reticulon-4-interacting protein 1, mitochondrial	RTN4IP1	0.105		
B4DNX9	Zinc finger protein 460	ZNF460	0.105	0.091	
O43639	Cytoplasmic protein NCK2	NCK2	0.105	-0.885	
Q9BW71-2	Isoform 2 of HIRA-interacting protein 3	HIRIP3	0.106	0.191	
P12235	ADP/ATP translocase 1	SLC25A4	0.106	-0.086	
O15213	WD repeat-containing protein 46	WDR46	0.107	0.271	
Q92506	Estradiol 17-beta-dehydrogenase 8	HSD17B8	0.107		
Q9H0R1-2	Isoform 2 of AP-5 complex subunit mu-1	AP5M1	0.107		
Q9C0J8	pre-mRNA 3' end processing protein WDR33	WDR33	0.108	0.075	
Q8N8J7	Uncharacterized protein C4orf32	C4orf32	0.108		
F8WBW1	COMM domain-containing protein 2	COMMD2	0.108	-0.045	
Q9ULC3	Ras-related protein Rab-23	RAB23	0.109	0.486	
E9PLM7	Serine/threonine-protein kinase BRSK2	BRSK2	0.109		
Q9NTJ4	Alpha-mannosidase 2C1	MAN2C1	0.109	0.160	
Q15833-2	Isoform 2 of Syntaxin-binding protein 2	STXBP2	0.109	0.134	
O95416	Transcription factor SOX-14	SOX14	0.110		
D6RF48	Syntaxin-18	STX18	0.111	0.067	
H7BYI7	3-hydroxyisobutyryl-CoA hydrolase, mitochondrial (Fragment)	HIBCH	0.111		
Q15057	Arf-GAP with coiled-coil, ANK repeat and PH domain-containing protein 2	ACAP2	0.111		
Q5W0Q3	Zinc finger MYM-type protein 2	ZMYM2	0.111		
E7ERL6	Microtubule-associated serine/threonine-protein kinase 2	MAST2	0.112	0.162	
Q96MW5	Conserved oligomeric Golgi complex subunit 8	COG8	0.113	0.093	
Q9BUT9	Protein FAM195A	FAM195A	0.113	0.052	
Q6NSJ5	Leucine-rich repeat-containing protein 8E	LRRC8E	0.113		
Q15120	[Pyruvate dehydrogenase [lipoamide]] kinase isozyme 3, mitochondrial	PDK3	0.113	0.060	
E7ESJ7	Protein FAM114A2	FAM114A2	0.114	0.005	
Q96KP1	Exocyst complex component 2	EXOC2	0.114		
Q76FK4-2	Isoform 2 of Nucleolar protein 8	NOL8	0.114	-0.067	
Q9H269-2	Isoform 2 of Vacuolar protein sorting-associated protein 16 homolog	VPS16	0.115	0.236	
P60059	Protein transport protein Sec61 subunit gamma	SEC61G	0.116	0.151	
A3KFJ0	Aurora kinase A	AURKA	0.116	-0.165	
Q96BP3	Peptidylprolyl isomerase domain and WD repeat-containing protein 1	PPWD1	0.116	-0.025	
Q6Y288	Beta-1,3-glucosyltransferase	B3GALTL	0.117		
Q96EY4	Translation machinery-associated protein 16	TMA16	0.118	-0.110	
O15084	Serine/threonine-protein phosphatase 6 regulatory ankyrin repeat subunit A	ANKRD28	0.120	0.130	
F8VUA7	Oxysterol-binding protein (Fragment)	OSBPL8	0.120	0.053	
P07910	Heterogeneous nuclear ribonucleoproteins C1/C2	HNRNPC	0.120	0.195	
B5MCL2	Glutathione S-transferase theta-2B	GSTT2	0.122		
Q99567	Nuclear pore complex protein Nup88	NUP88	0.122	-0.043	
Q9NPA0	ER membrane protein complex subunit 7	EMC7	0.122	0.276	
Q96AC1	Fermitin family homolog 2	FERMT2	0.123	0.138	
Q8WUA4	General transcription factor 3C polypeptide 2	GTF3C2	0.123	0.068	
Q9HBI1-3	Isoform 3 of Beta-parvin	PARVB	0.123		
E9PJV6	Syntaxin-17	STX17	0.124		
Q8N8R5	UPF0565 protein C2orf69	C2orf69	0.124		
P05549-5	Isoform 2 of Transcription factor AP-2-alpha	TFAP2A	0.124		
J3KSY6	5'(3')-deoxyribonucleotidase, cytosolic type	NT5C	0.125	-0.881	
Q15554-2	Isoform 2 of Telomeric repeat-binding factor 2	TERF2	0.125	0.001	
P61764	Syntaxin-binding protein 1	STXBP1	0.125		
C9J2P9	E3 ubiquitin-protein ligase Hakai (Fragment)	CBLL1	0.125	-0.122	
G3V158	2-deoxyribose-5-phosphate aldolase homolog (C. elegans), isoform CRA_a	DERA	0.125	0.149	
Q9NXA8-3	Isoform 3 of NAD-dependent protein deacylase sirtuin-5, mitochondrial	SIRT5	0.126		
H0YAS1	Fatty acid-binding protein 12 (Fragment)	FABP12	0.126		
O75179-6	Isoform 6 of Ankyrin repeat domain-containing protein 17	ANKRD17	0.126		
H0YB09	UPF0317 protein C14orf159, mitochondrial (Fragment)	C14orf159	0.128	0.284	
Q9NVJ2	ADP-ribosylation factor-like protein 8B	ARL8B	0.128	0.087	
M0QZP8	Immunity-related GTPase family Q protein (Fragment)	IRGQ	0.129	0.204	
Q9HBH0-2	Isoform 2 of Rho-related GTP-binding protein RhoF	RHOF	0.130		
B0QYR8	ADP-ribosylation factor-binding protein GGA1 (Fragment)	GGA1	0.130	0.088	
Q5VYS8-4	Isoform 4 of Terminal uridylyltransferase 7	ZCCHC6	0.130	-0.004	
Q9BRQ6	Coiled-coil-helix-coiled-coil-helix domain-containing protein 6, mitochondrial	CHCHD6	0.130	-0.010	
C9JCI8	Cyclic AMP-dependent transcription factor ATF-2 (Fragment)	ATF2	0.130		
E5RIA1	Glycerol-3-phosphate acyltransferase 4 (Fragment)	AGPAT6	0.131		
H7C0G1	Transmembrane protein 245 (Fragment)	TMEM245	0.131	0.226	
Q96S59	Ran-binding protein 9	RANBP9	0.131	0.187	
Q92541	RNA polymerase-associated protein RTF1 homolog	RTF1	0.132	0.088	
G5E9J4	TBCC domain containing 1, isoform CRA_a	TBCCD1	0.132		
Q9H832	Ubiquitin-conjugating enzyme E2 Z	UBE2Z	0.132	0.148	
F5H241	Glucosylceramidase	GBA	0.132	0.340	
Q9NVC6	Mediator of RNA polymerase II transcription subunit 17	MED17	0.132		
Q9NTG7-2	Isoform 2 of NAD-dependent protein deacetylase sirtuin-3, mitochondrial	SIRT3	0.133		
Q8NC96	Adaptin ear-binding coat-associated protein 1	NECAP1	0.133		
Q9Y3X0	Coiled-coil domain-containing protein 9	CCDC9	0.134	-0.028	
F8W108	AT-rich interactive domain-containing protein 2	ARID2	0.134	0.117	
B4DGL8	ATP-binding cassette sub-family B member 7, mitochondrial (Fragment)	ABCB7	0.135	0.216	
Q16822	Phosphoenolpyruvate carboxykinase [GTP], mitochondrial	PCK2	0.135		
Q9NPA8-2	Isoform 2 of Enhancer of yellow 2 transcription factor homolog	ENY2	0.135	-0.055	
P50416	Carnitine O-palmitoyltransferase 1, liver isoform	CPT1A	0.136		
P21359-2	Isoform 1 of Neurofibromin	NF1	0.136		
B7Z6Q6	Kinesin-like protein KIF2C	KIF2C	0.136	0.000	
Q4G148-2	Isoform 2 of Glucoside xylosyltransferase 1	GXYLT1	0.136		
J3QRQ3	Dihydroorotate dehydrogenase (quinone), mitochondrial (Fragment)	DHODH	0.136		
E9PG46	AP2-associated protein kinase 1	AAK1	0.137		

O60828-2	Isoform 2 of Polyglutamine-binding protein 1	PQBP1	0.137	-0.061	
Q9HD20-2	Isoform B of Probable cation-transporting ATPase 13A1	ATP13A1	0.138	0.120	
Q9Y3C1	Nucleolar protein 16	NOP16	0.138	-0.155	
Q9GZV1	Ankyrin repeat domain-containing protein 2	ANKRD2	0.138		
Q8WVB6	Chromosome transmission fidelity protein 18 homolog	CHTF18	0.139		
O95674	Phosphatidate cytidylyltransferase 2	CDS2	0.139	0.001	
E9PKE9	RING finger protein 121	RNF121	0.140		
Q6ZNB6	NF-X1-type zinc finger protein NFXL1	NFXL1	0.140	0.153	
P18440	Arylamine N-acetyltransferase 1	NAT1	0.140		
H0Y6T7	Nicastrin (Fragment)	NCSTN	0.140	0.305	
Q9HCN8	Stromal cell-derived factor 2-like protein 1	SDF2L1	0.140	-0.110	
P49454	Centromere protein F	CENPF	0.140	0.090	
B8ZZF0	Protein phosphatase 1B (Fragment)	PPM1B	0.141	0.014	
Q4V328-3	Isoform 3 of GRIP1-associated protein 1	GRIPAP1	0.142		
J3KMX2	SWI/SNF-related matrix-associated actin-dependent regulator of chromatin subfar	SMARCD2	0.142	-0.203	
Q24JP5-3	Isoform 3 of Transmembrane protein 132A	TMEM132A	0.142		
H7C2J7	Serine/threonine-protein kinase 11-interacting protein (Fragment)	STK11IP	0.143	-0.093	
K7EIX2	HEAT repeat-containing protein 6	HEATR6	0.143		
Q15334	Lethal(2) giant larvae protein homolog 1	LLGL1	0.143		
D6RJC3	Type II inositol 3,4-bisphosphate 4-phosphatase (Fragment)	INPP4B	0.144		
F5H8F7	Set1/Ash2 histone methyltransferase complex subunit ASH2	ASH2L	0.145	-0.003	
F8WF55	CDK5 regulatory subunit-associated protein 2	CDK5RAP2	0.145		
Q8NBJ7-2	Isoform 2 of Sulfatase-modifying factor 2	SUMF2	0.145	0.221	
O75976	Carboxypeptidase D	CPD	0.146	-0.258	
Q08426-2	Isoform 2 of Peroxisomal bifunctional enzyme	EHHADH	0.146		
O00468-2	Isoform 2 of Agrin	AGRN	0.146	0.245	
O43292-2	Isoform 2 of Glycosylphosphatidylinositol anchor attachment 1 protein	GPAA1	0.146		
H3BPF2	Probable ribosome biogenesis protein RLP24	RSL24D1	0.147		
Q9NZ45	CDGSH iron-sulfur domain-containing protein 1	CISD1	0.147	0.283	
O94760	N(G),N(G)-dimethylarginine dimethylaminohydrolase 1	DDAH1	0.147	-0.007	
E7EVQ6	Squalene monooxygenase	SQLE	0.148	0.166	
Q8TBP6	Solute carrier family 25 member 40	SLC25A40	0.148	0.286	
P54652	Heat shock-related 70 kDa protein 2	HSPA2	0.149	-0.153	
O15431	High affinity copper uptake protein 1	SLC31A1	0.149		
Q15208	Serine/threonine-protein kinase 38	STK38	0.150		
Q9C0B5-2	Isoform 2 of Palmitoyltransferase ZDHHC5	ZDHHC5	0.150		
Q12846	Syntaxin-4	STX4	0.151	0.225	
B7Z6X7	S1 RNA-binding domain-containing protein 1	SRBD1	0.151		
Q12996	Cleavage stimulation factor subunit 3	CSTF3	0.151		
Q9Y6I3-3	Isoform 3 of Epsin-1	EPN1	0.151	0.072	
P63165	Small ubiquitin-related modifier 1	SUMO1	0.152	-0.126	
Q8TD30-2	Isoform 2 of Alanine aminotransferase 2	GPT2	0.152	0.418	
Q13618-2	Isoform 2 of Cullin-3	CUL3	0.153	0.025	
Q8IW45	ATP-dependent (S)-NAD(P)H-hydrate dehydratase	CARKD	0.153	0.285	
P40121-2	Isoform 2 of Macrophage-capping protein	CAPG	0.154	0.125	
P05362	Intercellular adhesion molecule 1	ICAM1	0.154		
P04150-6	Isoform Beta-2 of Glucocorticoid receptor	NR3C1	0.154		
Q96FV9	THO complex subunit 1	THOC1	0.154	-0.135	
Q96ER9	Coiled-coil domain-containing protein 51	CCDC51	0.155	0.189	
P41212	Transcription factor ETV6	ETV6	0.155	0.272	
G3V4G1	Neuroguidin (Fragment)	NGDN	0.155	-0.005	
O15344-2	Isoform 2 of Midline-1	MID1	0.156	-0.472	
P53675-2	Isoform 2 of Clathrin heavy chain 2	CLTCL1	0.156		
Q5BJF2	Transmembrane protein 97	TMEM97	0.156	0.148	
Q9NQS7-2	Isoform 2 of Inner centromere protein	INCENP	0.156	-0.397	
Q15031	Probable leucine--tRNA ligase, mitochondrial	LARS2	0.156		
Q86Y79	Probable peptidyl-tRNA hydrolase	PTRH1	0.157		
H0YFT9	Ubiquitin carboxyl-terminal hydrolase 28 (Fragment)	USP28	0.157		
O43353-2	Isoform 2 of Receptor-interacting serine/threonine-protein kinase 2	RIPK2	0.157	0.437	
F8WBE2	Cytochrome P450 20A1	CYP20A1	0.158		
E9PD90	Cytoskeleton-associated protein 2	CKAP2	0.158	-0.191	
O75151	Lysine-specific demethylase PHF2	PHF2	0.158	-0.092	
Q86YV9	Hermansky-Pudlak syndrome 6 protein	HPS6	0.159	0.385	
Q9H7C9-3	Isoform 3 of Mth938 domain-containing protein	AAMDC	0.160	0.001	
H0Y8I7	DNA topoisomerase 2-binding protein 1 (Fragment)	TOPBP1	0.160		
Q14331	Protein FRG1	FRG1	0.160	0.168	
Q9H1Y0-2	Isoform Short of Autophagy protein 5	ATG5	0.160		
O94761	ATP-dependent DNA helicase Q4	RECQL4	0.160	0.067	
Q99963-4	Isoform 4 of Endophilin-A3	SH3GL3	0.160		
Q8N1G2	Cap-specific mRNA (nucleoside-2'-O-)-methyltransferase 1	FTSJD2	0.161	0.042	
D6RB24	Adaptin ear-binding coat-associated protein 2	NECAP2	0.162	-0.079	
M0QYC5	Paired amphipathic helix protein Sin3b	SIN3B	0.162		
E5RGN0	Cyclin-dependent kinase 16 (Fragment)	CDK16	0.163		
Q8IZ21-3	Isoform 3 of Phosphatase and actin regulator 4	PHACTR4	0.163	0.146	
Q9H2H8	Peptidyl-prolyl cis-trans isomerase-like 3	PPIL3	0.163		
Q15172-2	Isoform 2 of Serine/threonine-protein phosphatase 2A 56 kDa regulatory subunit	PPP2R5A	0.164	0.010	
P15407	Fos-related antigen 1	FOSL1	0.165		
Q8IXQ4-3	Isoform 3 of Uncharacterized protein KIAA1704	KIAA1704	0.166		
Q96EK5	KIF1-binding protein	KIAA1279	0.167	-0.035	
Q13330-3	Isoform 3 of Metastasis-associated protein MTA1	MTA1	0.169	-0.005	
H3BN64	Zinc finger FYVE domain-containing protein 19 (Fragment)	ZFYVE19	0.169	0.162	
H7C3S0	Quinone oxidoreductase-like protein 1 (Fragment)	CRYZL1	0.169		
H0Y412	Protein PRRC2B (Fragment)	PRRC2B	0.169	-0.129	
Q9NXV2	BTB/POZ domain-containing protein KCTD5	KCTD5	0.169	-0.021	
P48448	Aldehyde dehydrogenase family 3 member B2	ALDH3B2	0.170		
E7ER97	N-acetylserotonin O-methyltransferase-like protein	ASMTL	0.170	-0.003	
Q9Y672	Dolichyl pyrophosphate Man9GlcNAc2 alpha-1,3-glucosyltransferase	ALG6	0.170	0.126	

Q9UMY4-2	Isoform 2 of Sorting nexin-12	SNX12	0.171	0.090	
O60942-3	Isoform 3 of mRNA-capping enzyme	RNGTT	0.171		
E9PSG7	Phosphofurin acidic cluster sorting protein 1	PACS1	0.171		
Q8TDD1	ATP-dependent RNA helicase DDX54	DDX54	0.171	-0.209	
Q14571	Inositol 1,4,5-trisphosphate receptor type 2	ITPR2	0.174	-0.079	
Q9NRZ9-2	Isoform 2 of Lymphoid-specific helicase	HELLS	0.175		
P21953	2-oxoisovalerate dehydrogenase subunit beta, mitochondrial	BCKDHB	0.175		
E9PQ01	Pre-mRNA cleavage complex 2 protein Pcf11 (Fragment)	PCF11	0.175	0.400	
Q9BTA9-3	Isoform 3 of WW domain-containing adapter protein with coiled-coil	WAC	0.176	0.380	
Q16647	Prostacyclin synthase	PTGIS	0.176		
E9PKY5	Peptidyl-prolyl cis-trans isomerase (Fragment)	PPIE	0.177		
O60645-3	Isoform 3 of Exocyst complex component 3	EXOC3	0.178	0.122	
Q86VI3	Ras GTPase-activating-like protein IQGAP3	IQGAP3	0.179	0.057	
Q9P2K5-4	Isoform 4 of Myelin expression factor 2	MYEF2	0.179	-0.310	
Q92876	Kallikrein-6	KLK6	0.179		
Q9BW19	Kinesin-like protein KIFC1	KIFC1	0.180	-0.264	
P32970	CD70 antigen	CD70	0.180		
O15169-2	Isoform 2 of Axin-1	AXIN1	0.180		
Q8WX93-8	Isoform 8 of Palladin	PALLD	0.180	0.322	
O60739	Eukaryotic translation initiation factor 1b	EIF1B	0.181	0.176	
D6RG19	Ribosomal protein L37	RPL37	0.181	-0.070	
O95630	STAM-binding protein	STAMBP	0.182	0.219	
P36404	ADP-ribosylation factor-like protein 2	ARL2	0.182	0.133	
Q3ZK31	Serine racemase (Fragment)	SRR	0.182		
H3BNY4	WD repeat-containing protein 59 (Fragment)	WDR59	0.182		
O95352-2	Isoform 2 of Ubiquitin-like modifier-activating enzyme ATG7	ATG7	0.183		
M0QX73	Glioma tumor suppressor candidate region gene 2 protein (Fragment)	GLTSCR2	0.183	-0.095	
C9JYP1	Protein FAM107B (Fragment)	FAM107B	0.183		
H0YEP3	Probable aminopeptidase NPEPL1 (Fragment)	NPEPL1	0.183		
Q9NVH0-2	Isoform 2 of Exonuclease 3'-5' domain-containing protein 2	EXD2	0.183		
Q8N3R9-2	Isoform 2 of MAGUK p55 subfamily member 5	MPP5	0.184		
Q96MX3	Zinc finger protein 48	ZNF48	0.184		
O95081-2	Isoform 2 of Arf-GAP domain and FG repeat-containing protein 2	AGFG2	0.185		
E9PNT5	DNA repair protein RAD51 homolog 1 (Fragment)	RAD51	0.186		
K7ESB7	Dedicator of cytokinesis protein 6 (Fragment)	DOCK6	0.186		
Q9UJX6-2	Isoform 2 of Anaphase-promoting complex subunit 2	ANAPC2	0.187		
Q8N2A8	Mitochondrial cardiolipin hydrolase	PLD6	0.187		
O60522-2	Isoform 2 of Tudor domain-containing protein 6	TDRD6	0.188	-0.001	
Q9Y4E1-5	Isoform 5 of WASH complex subunit FAM21C	FAM21C	0.190	-0.049	
Q6YP21	Kynurenine--oxoglutarate transaminase 3	CCBL2	0.190	-0.057	
Q9BWT1-3	Isoform 3 of Cell division cycle-associated protein 7	CDCA7	0.191		
P26447	Protein S100-A4	S100A4	0.191	0.110	
C9JNA8	Inositol hexakisphosphate kinase 1	IP6K1	0.191		
P10301	Ras-related protein R-Ras	RRAS	0.191		
J3KPD3	RNA binding motif protein 7, isoform CRA_c	RBM7	0.192	0.073	
Q14676-4	Isoform 4 of Mediator of DNA damage checkpoint protein 1	MDC1	0.192	-0.381	
Q9BRY0	Zinc transporter ZIP3	SLC39A3	0.192	0.320	
Q9BRU9	rRNA-processing protein UTP23 homolog	UTP23	0.193		
I3L1D4	RNA-binding protein fox-1 homolog 1 (Fragment)	RBFOX1	0.193		
Q9UPY3-2	Isoform 2 of Endoribonuclease Dicer	DICER1	0.193		
Q9NW82	WD repeat-containing protein 70	WDR70	0.193	0.065	
J3QQM4	E3 ubiquitin-protein ligase SMURF2 (Fragment)	SMURF2	0.194		
Q96CF2	Charged multivesicular body protein 4c	CHMP4C	0.194		
Q9Y597-2	Isoform 2 of BTB/POZ domain-containing protein KCTD3	KCTD3	0.196		
F8WE49	E3 ubiquitin-protein ligase RAD18	RAD18	0.197		
B7Z1J9	Parkinson disease 7 domain-containing protein 1	PDDC1	0.197	-0.391	
P29350-2	Isoform 3 of Tyrosine-protein phosphatase non-receptor type 6	PTPN6	0.197		
H0YAH3	N-acetylgalactosaminyltransferase 7 (Fragment)	GALNT7	0.198		
Q8NE01-2	Isoform 2 of Metal transporter CNNM3	CNNM3	0.198		
Q96GW9	Methionine--tRNA ligase, mitochondrial	MARS2	0.199		
F5H6G7	Nucleolar protein 10	NOL10	0.199	-0.240	
Q5T0W9	Protein FAM83B	FAM83B	0.202		
E5RJ24	TBC1 domain family member 3 (Fragment)	TBC1D3	0.203		
O95425-2	Isoform 2 of Supervillin	SVIL	0.203		
Q9NQG1	Protein MANBAL	MANBAL	0.205		
Q7L3T8	Probable proline--tRNA ligase, mitochondrial	PARS2	0.205		
E7EVG2	Protein polybromo-1 (Fragment)	PBRM1	0.205	0.033	
Q9P2X3-2	Isoform 2 of Protein IMPACT	IMPACT	0.206		
H0YBX3	Ribonuclease UK114 (Fragment)	HRSP12	0.206	0.127	
O60292	Signal-induced proliferation-associated 1-like protein 3	SIPA1L3	0.207		
Q9UKN8	General transcription factor 3C polypeptide 4	GTF3C4	0.207	-0.034	
R4GN94	Ankyrin repeat and SOCS box protein 9 (Fragment)	ASB9	0.207	-0.009	
Q5JTZ9	Alanine--tRNA ligase, mitochondrial	AARS2	0.208		
H3BSJ6	G-protein-coupled receptor 56 (Fragment)	GPR56	0.208		
Q92626	Peroxidasin homolog	PXDN	0.209		
H0YL53	Protein regulator of cytokinesis 1 (Fragment)	PRC1	0.210		
Q9BSY4	Coiled-coil-helix-coiled-coil-helix domain-containing protein 5	CHCHD5	0.211		
H0YNJ7	Cytochrome P450 19A1 (Fragment)	CYP19A1	0.213		
Q9BTU6	Phosphatidylinositol 4-kinase type 2-alpha	PI4K2A	0.213	0.639	
Q15910-3	Isoform 3 of Histone-lysine N-methyltransferase EZH2	EZH2	0.215	-0.188	
Q53RE8	Ankyrin repeat domain-containing protein 39	ANKRD39	0.217		
Q86W42-2	Isoform 2 of THO complex subunit 6 homolog	THOC6	0.217	-0.148	
E9PHI4	SUN domain-containing protein 1	SUN1	0.218	0.056	
E9PKW1	Latent-transforming growth factor beta-binding protein 3 (Fragment)	LTBP3	0.219		
P36954	DNA-directed RNA polymerase II subunit RPB9	POLR2I	0.220	-0.182	
B4DTG6	Protein LSM14 homolog A	LSM14A	0.221	0.141	
Q99685-2	Isoform 2 of Monoglyceride lipase	MGLL	0.221		

Q9BY43	Charged multivesicular body protein 4a	CHMP4A	0.221		
Q01831-2	Isoform 2 of DNA repair protein complementing XP-C cells	XPC	0.222	-0.213	
P39060-2	Isoform 3 of Collagen alpha-1(XVIII) chain	COL18A1	0.222		
Q15836	Vesicle-associated membrane protein 3	VAMP3	0.223	0.231	
O75528-2	Isoform 2 of Transcriptional adapter 3	TADA3	0.223		
Q13951	Core-binding factor subunit beta	CBFB	0.224	-0.076	
Q9BXS6-3	Isoform 3 of Nucleolar and spindle-associated protein 1	NUSAP1	0.227	0.183	
E9PJN3	Protein phosphatase 1A (Fragment)	PPM1A	0.228	-0.049	
Q6JQN1-3	Isoform 3 of Acyl-CoA dehydrogenase family member 10	ACAD10	0.228		
Q13542	Eukaryotic translation initiation factor 4E-binding protein 2	EIF4EBP2	0.231	-0.076	
E5RH41	Transcription initiation factor IIE subunit beta (Fragment)	GTF2E2	0.232		
F5GYF8	Signal-induced proliferation-associated 1-like protein 1	SIPA1L1	0.232		
Q9H2G2-2	Isoform 2 of STE20-like serine/threonine-protein kinase	SLK	0.233	0.056	
P02462-2	Isoform 2 of Collagen alpha-1(IV) chain	COL4A1	0.235	-0.472	
Q6P1M3-2	Isoform A of Lethal(2) giant larvae protein homolog 2	LLGL2	0.235	0.359	
Q9NVN8	Guanine nucleotide-binding protein-like 3-like protein	GNL3L	0.235	0.119	
H3BNB9	E3 ubiquitin-protein ligase ARIH1 (Fragment)	ARIH1	0.235	0.083	
O60287	Nucleolar pre-ribosomal-associated protein 1	URB1	0.235	0.042	
B4DKL4	Lipolysis-stimulated lipoprotein receptor	LSR	0.237	-0.139	
O75884	Putative hydrolase RBBP9	RBBP9	0.238		
Q96JH7	Deubiquitinating protein VCIP135	VCPIP1	0.240	0.071	
P38571-2	Isoform 2 of Lysosomal acid lipase/cholesteryl ester hydrolase	LIPA	0.240		
Q14807-2	Isoform 2 of Kinesin-like protein KIF22	KIF22	0.242		
Q86VU5	Catechol O-methyltransferase domain-containing protein 1	COMTD1	0.243		
Q8IWA5-3	Isoform 3 of Choline transporter-like protein 2	SLC44A2	0.244	0.157	
A8MU21	Transmembrane protein 147	TMEM147	0.244	0.258	
I3L0V5	A-kinase anchor protein 1, mitochondrial (Fragment)	AKAP1	0.245		
Q96G25-3	Isoform 3 of Mediator of RNA polymerase II transcription subunit 8	MED8	0.245	0.169	
Q96C00	Zinc finger and BTB domain-containing protein 9	ZBTB9	0.248		
P63218	Guanine nucleotide-binding protein G(I)/G(S)/G(O) subunit gamma-5	GNG5	0.249	0.196	
E7EMW7	E3 ubiquitin-protein ligase UBR5	UBR5	0.250	-0.015	
Q9UBP9-2	Isoform 2 of PTB domain-containing engulfment adapter protein 1	GULP1	0.251		
P50440-2	Isoform 2 of Glycine amidinotransferase, mitochondrial	GATM	0.251		
H7C3T2	Autophagy-related protein 2 homolog A (Fragment)	ATG2A	0.252		
P35240-4	Isoform 4 of Merlin	NF2	0.252	-0.248	
Q8N531-2	Isoform 2 of F-box/LRR-repeat protein 6	FBXL6	0.253		
P04053-2	Isoform 2 of DNA nucleotidylexotransferase	DNTT	0.255		
Q9UHJ6	Sedoheptulokinase	SHPK	0.256	0.122	
Q9BRK5-4	Isoform 4 of 45 kDa calcium-binding protein	SDF4	0.256	0.021	
Q9ULD0	2-oxoglutarate dehydrogenase-like, mitochondrial	OGDHL	0.258		
Q7L5L3-1	Isoform 2 of Glycerophosphodiester phosphodiesterase domain-containing protein	GDPD3	0.261		
O75907	Diacylglycerol O-acyltransferase 1	DGAT1	0.264	0.050	
P09455	Retinol-binding protein 1	RBP1	0.265		
O94919	Endonuclease domain-containing 1 protein	ENDOD1	0.266	0.134	
P05534	HLA class I histocompatibility antigen, A-24 alpha chain	HLA-A	0.267		
B0QYI3	TBC1 domain family member 22A	TBC1D22A	0.269		
E9PM95	Apolipoprotein L2 (Fragment)	APOL2	0.271		
Q86VY4	Testis-specific Y-encoded-like protein 5	TSPYL5	0.272	-0.005	
F6RY50	Signal-induced proliferation-associated protein 1	SIPA1	0.274		
Q8TEA7-3	Isoform 3 of TBC domain-containing protein kinase-like protein	TBCK	0.275		
Q9C0K1-2	Isoform 2 of Zinc transporter ZIP8	SLC39A8	0.277		
P58107	Epiplakin	EPPK1	0.277		
Q9P016	Thymocyte nuclear protein 1	THYN1	0.278		
O75792	Ribonuclease H2 subunit A	RNASEH2A	0.278	-0.060	
P08240	Signal recognition particle receptor subunit alpha	SRPR	0.280	-0.023	
P09104	Gamma-enolase	ENO2	0.282	-0.051	
P80217	Interferon-induced 35 kDa protein	IFI35	0.285		
O43657	Tetraspanin-6	TSPAN6	0.289		
Q9BZI7-2	Isoform 2 of Regulator of nonsense transcripts 3B	UPF3B	0.290		
P29762	Cellular retinoic acid-binding protein 1	CRABP1	0.293		
Q01415	N-acetylgalactosamine kinase	GALK2	0.299		
Q16352	Alpha-internexin	INA	0.300		
Q86SK9-2	Isoform 2 of Stearoyl-CoA desaturase 5	SCD5	0.302	-0.110	
Q96DE0-3	Isoform 3 of U8 snoRNA-decapping enzyme	NUDT16	0.303		
Q8IXZ2-2	Isoform 2 of Zinc finger CCCH domain-containing protein 3	ZC3H3	0.304		
Q8TED9-3	Isoform 3 of Actin filament-associated protein 1-like 1	AFAP1L1	0.306		
G3V235	Cyclin-K (Fragment)	CCNK	0.307		
P08582	Melanotransferrin	MFI2	0.308	0.053	
O95274	Ly6/PLAUR domain-containing protein 3	LYPD3	0.310	0.906	
Q6UW56	All-trans retinoic acid-induced differentiation factor	ATRAID	0.313	0.175	
P30038-2	Isoform 2 of Delta-1-pyrroline-5-carboxylate dehydrogenase, mitochondrial	ALDH4A1	0.314	0.336	
E9PH32	Mucin-20	MUC20	0.315		
Q13287	N-myc-interactor	NMI	0.315		
Q9Y223	Bifunctional UDP-N-acetylglucosamine 2-epimerase/N-acetylmannosamine kinase	GNE	0.316	0.186	
Q5JVS0-2	Isoform 2 of Intracellular hyaluronan-binding protein 4	HABP4	0.327		
B4DRC4	Ankyrin repeat and SOCS box protein 6	ASB6	0.327		
D6RE80	Macrophage erythroblast attacher (Fragment)	MAEA	0.327		
P57105	Synaptojanin-2-binding protein	SYNJ2BP	0.330		
P10321	HLA class I histocompatibility antigen, Cw-7 alpha chain	HLA-C	0.332		
P61011	Signal recognition particle 54 kDa protein	SRP54	0.333		
Q8N5P1	Zinc finger CCCH domain-containing protein 8	ZC3H8	0.336		
A2ACR1	Proteasome subunit beta type	PSMB9	0.338	0.513	
Q96HR9	Receptor expression-enhancing protein 6	REEP6	0.338		
O95377	Gap junction beta-5 protein	GJB5	0.340		
K7EJ48	Protein Hook homolog 2 (Fragment)	HOOK2	0.346		
G5E9A6	Ubiquitin carboxyl-terminal hydrolase	USP11	0.348	0.006	
Q7L4S7	Protein ARMCX6	ARMCX6	0.349	-0.010	

O95870-2	Isoform 2 of Abhydrolase domain-containing protein 16A	ABHD16A	0.350	0.216	
Q13586	Stromal interaction molecule 1	STIM1	0.356	0.351	
K7EMY9	Cold-inducible RNA-binding protein (Fragment)	CIRBP	0.359	0.081	
H0YCB1	Nuclear receptor-binding protein 2 (Fragment)	NRBP2	0.361		
H3BNG6	Synaptosomal-associated protein 23	SNAP23	0.362		
E9PFC1	Centrosomal protein of 170 kDa protein B	CEP170B	0.369		
Q8TAE8	Growth arrest and DNA damage-inducible proteins-interacting protein 1	GADD45GIP1	0.370	-0.119	
P32004-3	Isoform 3 of Neural cell adhesion molecule L1	L1CAM	0.373		
Q7Z406	Myosin-14	MYH14	0.379	0.344	
Q92743	Serine protease HTRA1	HTRA1	0.381		
Q5JWV1	Uridine-cytidine kinase-like 1 (Fragment)	UCKL1	0.383	-0.077	
Q8WUM9	Sodium-dependent phosphate transporter 1	SLC20A1	0.384		
Q9Y337	Kallikrein-5	KLK5	0.386		
Q03519	Antigen peptide transporter 2	TAP2	0.394	0.180	
Q5VW52	Glycerol-3-phosphate acyltransferase 1, mitochondrial	GPAM	0.400		
P29590-11	Isoform PML-11 of Protein PML	PML	0.402		
Q96PV6-2	Isoform 2 of Leukocyte receptor cluster member 8	LENG8	0.403	0.177	
Q86VM6	MBNL1 protein	MBNL1	0.404		
Q96MU7-2	Isoform 2 of YTH domain-containing protein 1	YTHDC1	0.410		
B7Z3B9	GRB2-associated-binding protein 1	GAB1	0.415		
P24821-4	Isoform 4 of Tenascin	TNC	0.417		
Q9Y4I1-2	Isoform 2 of Unconventional myosin-Va	MYO5A	0.419	0.158	
Q9H2C0	Gigaxonin	GAN	0.424		
H0Y7P1	Mediator of RNA polymerase II transcription subunit 12 (Fragment)	MED12	0.429		
P50750	Cyclin-dependent kinase 9	CDK9	0.434	-0.069	
Q9UHF7	Zinc finger transcription factor Trps1	TRPS1	0.445	0.029	
H0YER2	Activating signal cointegrator 1 complex subunit 1 (Fragment)	ASCC1	0.446		
H3BN14	Calretinin (Fragment)	CALB2	0.449		
H0YAK0	N-acetylgalactosaminyltransferase 7 (Fragment)	GALNT7	0.453		
Q9C0C9	Ubiquitin-conjugating enzyme E2 O	UBE2O	0.460	-0.080	
P58397-3	Isoform 3 of A disintegrin and metalloproteinase with thrombospondin motifs 12	ADAMTS12	0.461		
O75923-3	Isoform 3 of Dysferlin	DYSF	0.465		
C9J712	Profilin-2	PFN2	0.479	-0.242	
Q5SS58	MHC class I polypeptide-related sequence A	MICA	0.490		
B3KX27	Rhophilin-2	RHPN2	0.520	-0.349	
G3V291	Iron-sulfur cluster assembly 2 homolog, mitochondrial	ISCA2	0.521		
Q14149	MORC family CW-type zinc finger protein 3	MORC3	0.521	-0.219	
P01889	HLA class I histocompatibility antigen, B-7 alpha chain	HLA-B	0.523		
P84157-2	Isoform 2 of Matrix-remodeling-associated protein 7	MXRA7	0.524		
P30486	HLA class I histocompatibility antigen, B-48 alpha chain	HLA-B	0.528		
H7C5A7	Acyl-coenzyme A thioesterase 8 (Fragment)	ACOT8	0.530	0.102	
D6RB85	Calnexin (Fragment)	CANX	0.533		
O95833	Chloride intracellular channel protein 3	CLIC3	0.536		
P30447	HLA class I histocompatibility antigen, A-23 alpha chain	HLA-A	0.538		
Q969L2	Protein MAL2	MAL2	0.543		
Q9BYG5	Partitioning defective 6 homolog beta	PARD6B	0.545	0.117	
B4DIU3	Protein-arginine deiminase type-2	PADI2	0.548	0.346	
K7EQF2	1-phosphatidylinositol 4,5-bisphosphate phosphodiesterase delta-3 (Fragment)	PLCD3	0.555		
P30481	HLA class I histocompatibility antigen, B-44 alpha chain	HLA-B	0.558		
Q9Y4B6-3	Isoform 3 of Protein VPRBP	VPRBP	0.687		
M0QZ93	Galectin-4 (Fragment)	LGALS4	0.695	-0.720	
F8VPW7	2'-5'-oligoadenylate synthase 1 (Fragment)	OAS1	0.700		
Q587J7	Tudor domain-containing protein 12	TDRD12	0.723	0.058	
Q86YZ3	Hornerin	HRNR	0.740	1.923	
I3L443	RNA methyltransferase-like protein 1 (Fragment)	RNMTL1	0.768	0.454	
Q96GX5-2	Isoform 2 of Serine/threonine-protein kinase greatwall	MASTL	0.869	0.819	
H7C528	Polyhomeotic-like protein 3 (Fragment)	PHC3	1.037		
Q5TBQ1	Protein LSM14 homolog B (Fragment)	LSM14B	1.217		
H0YH01	A-kinase anchor protein 7 isoform gamma (Fragment)	AKAP7	1.349	0.813	
P08493	Matrix Gla protein	MGP	1.540		
O14772-2	Isoform 5 of Fucose-1-phosphate guanylyltransferase	FPGT		0.323	
O43148	mRNA cap guanine-N7 methyltransferase	RNMT		0.324	
P05204	Non-histone chromosomal protein HMG-17	HMGN2		-1.052	
Q6P087-2	Isoform 2 of RNA pseudouridylate synthase domain-containing protein 3	RPUSD3		-0.996	
B5MCB4	Methyl-CpG-binding protein 2	MECP2		-0.973	
C0H5Y7	BAK1 protein	BAK1		-0.854	
P16104	Histone H2A.x	H2AFX		-0.804	
F6TQG2	Probable global transcription activator SNF2L1 (Fragment)	SMARCA1		-0.731	
P42167-2	Isoform Gamma of Lamina-associated polypeptide 2, isoforms beta/gamma	TMPO		-0.666	
A6NCS9	Serologically defined colon cancer antigen 8	SDCCAG8		-0.627	
O15061	Synemin	SYNM		-0.500	
J3KRL6	Nuclear factor of-activated T-cells 5 (Fragment)	NFAT5		-0.481	
E7ESK6	Syndecan	SDC2		-0.453	
I3L305	Syntaxin-8 (Fragment)	STX8		-0.443	
I3L1H3	Lipopolysaccharide-induced tumor necrosis factor-alpha factor	LITAF		-0.436	
H7C2Q2	H/ACA ribonucleoprotein complex subunit 4 (Fragment)	DKC1		-0.433	
Q63ZY3-3	Isoform 3 of KN motif and ankyrin repeat domain-containing protein 2	KANK2		-0.432	
O60566-2	Isoform 2 of Mitotic checkpoint serine/threonine-protein kinase BUB1 beta	BUB1B		-0.399	
Q9BUP0	EF-hand domain-containing protein D1	EFHD1		-0.394	
O43678-2	Isoform 2 of NADH dehydrogenase [ubiquinone] 1 alpha subcomplex subunit 2	NDUFA2		-0.388	
Q5SZR4	Tudor and KH domain containing, isoform CRA_a	TDRKH		-0.372	
Q9HCJ1	Progressive ankylosis protein homolog	ANKH		-0.371	
J3QL03	Spindle and kinetochore-associated protein 2 (Fragment)	SKA2		-0.348	
A6NFI3	Zinc finger protein 316	ZNF316		-0.347	
E9PIQ3	Bromodomain-containing protein 2	BRD2		-0.342	
Q6IQ42	FUSIP1 protein	FUSIP1		-0.342	
Q96H79	Zinc finger CCCH-type antiviral protein 1-like	ZC3HAV1L		-0.330	

E9PEI0	Cell division cycle-associated protein 2	CDCA2		-0.326	
P11532-10	Isoform 10 of Dystrophin	DMD		-0.325	
Q8IYL3	UPF0688 protein C1orf174	C1orf174		-0.322	
B0V297	Zinc finger protein RFP (Fragment)	TRIM27		-0.303	
H0YNH6	ER membrane protein complex subunit 9	EMC9		-0.298	
Q8NFC6	Biorientation of chromosomes in cell division protein 1-like 1	BOD1L1		-0.295	
G3V3T0	Ataxin-3	ATXN3		-0.293	
P43357	Melanoma-associated antigen 3	MAGEA3		-0.284	
H0YMJ0	Mortality factor 4-like protein 1 (Fragment)	MORF4L1		-0.279	
B4DUS4	Ubiquitin carboxyl-terminal hydrolase 16	USP16		-0.278	
Q9UH17	DNA dC->dU-editing enzyme APOBEC-3B	APOBEC3B		-0.276	
P78563-2	Isoform 2 of Double-stranded RNA-specific editase 1	ADARB1		-0.274	
H0Y4R5	Transmembrane protein 201 (Fragment)	TMEM201		-0.267	
H0Y835	Rho GTPase-activating protein 26 (Fragment)	ARHGAP26		-0.262	
P27448-8	Isoform 7 of MAP/microtubule affinity-regulating kinase 3	MARK3		-0.260	
E7ESJ6	Histone deacetylase 3 (Fragment)	HDAC3		-0.259	
Q9Y620	DNA repair and recombination protein RAD54B	RAD54B		-0.250	
P33527-8	Isoform 8 of Multidrug resistance-associated protein 1	ABCC1		-0.249	
Q9Y4H2	Insulin receptor substrate 2	IRS2		-0.243	
Q9UP83-3	Isoform 3 of Conserved oligomeric Golgi complex subunit 5	COG5		-0.242	
P55199	RNA polymerase II elongation factor ELL	ELL		-0.241	
Q5VWK5-7	Isoform 7 of Interleukin-23 receptor	IL23R		-0.239	
P17028-2	Isoform 2 of Zinc finger protein 24	ZNF24		-0.238	
P61421	V-type proton ATPase subunit d 1	ATP6V0D1		-0.231	
C9JVE2	DCN1, defective in cullin neddylation 1, domain containing 1 (S. cerevisiae), isofor	DCUN1D1		-0.228	
H0YD08	Ubiquitin carboxyl-terminal hydrolase 33 (Fragment)	USP33		-0.223	
Q9UQR0	Sex comb on midleg-like protein 2	SCML2		-0.222	
F6VZ39	RNA-binding protein 38	RBM38		-0.214	
G3V434	Ubiquinone biosynthesis monooxygenase COQ6	COQ6		-0.211	
B5MCT9	Syntaxin-binding protein 4	STXBP4		-0.210	
O00255-3	Isoform 3 of Menin	MEN1		-0.204	
Q9BPZ3	Polyadenylate-binding protein-interacting protein 2	PAIP2		-0.199	
Q5JV61	FERM domain-containing protein 3 (Fragment)	FRMD3		-0.196	
Q96R05	Retinoid-binding protein 7	RBP7		-0.195	
Q08AE8-2	Isoform 2 of Protein spire homolog 1	SPIRE1		-0.195	
H0YG27	Tripartite motif-containing protein 65 (Fragment)	TRIM65		-0.193	
H3BSM5	Gamma-aminobutyric acid receptor-associated protein-like 2	GABARAPL2		-0.192	
Q8N2Z9-3	Isoform 3 of Centromere protein S	APITD1		-0.191	
Q9NS39	Double-stranded RNA-specific editase B2	ADARB2		-0.188	
Q9NSC5-4	Isoform 4 of Homer protein homolog 3	HOMER3		-0.188	
Q96GS4	Uncharacterized protein C17orf59	C17orf59		-0.184	
A6NLG9	Biglycan	BGN		-0.183	
O14936-3	Isoform 3 of Peripheral plasma membrane protein CASK	CASK		-0.183	
H0YDJ3	Serine/threonine-protein kinase PRP4 homolog (Fragment)	PRPF4B		-0.182	
H7C089	Tetratricopeptide repeat protein 38 (Fragment)	TTC38		-0.181	
B3EWG3	Protein FAM25A	FAM25A		-0.180	
Q5T9S2	Coiled-coil domain-containing protein 18 (Fragment)	CCDC18		-0.174	
E5RFY9	Uncharacterized protein C1orf198	C1orf198		-0.173	
Q9Y6X1	Stress-associated endoplasmic reticulum protein 1	SERP1		-0.172	
Q9NY12-2	Isoform 2 of H/ACA ribonucleoprotein complex subunit 1	GAR1		-0.169	
Q96HA7	Tonsoku-like protein	TONSL		-0.169	
Q16880	2-hydroxyacylsphingosine 1-beta-galactosyltransferase	UGT8		-0.166	
Q9UBT7-3	Isoform 3 of Alpha-catulin	CTNNAL1		-0.166	
A4D212	DKFZP586J0619 protein	DKFZP586J0619		-0.164	
Q9H7S9	Zinc finger protein 703	ZNF703		-0.162	
Q8NAV1	Pre-mRNA-splicing factor 38A	PRPF38A		-0.161	
Q9NX02-4	Isoform 4 of NACHT, LRR and PYD domains-containing protein 2	NLRP2		-0.160	
Q12926	ELAV-like protein 2	ELAVL2		-0.156	
H0Y7M8	Histone lysine demethylase PHF8 (Fragment)	PHF8		-0.156	
E9PKP0	Ribonuclease H2 subunit C	RNASEH2C		-0.155	
Q9H1E3-2	Isoform 2 of Nuclear ubiquitous casein and cyclin-dependent kinase substrate 1	NUCKS1		-0.155	
F5H074	Lysosomal-trafficking regulator	LYST		-0.155	
Q8IUW1	ANKRD10 protein	ANKRD10		-0.155	
B4DNU3	Niban-like protein 2	FAM129C		-0.154	
P36959	GMP reductase 1	GMPR		-0.151	
Q9BW61	DET1- and DDB1-associated protein 1	DDA1		-0.151	
Q86U70-3	Isoform 2 of LIM domain-binding protein 1	LDB1		-0.148	
H0YLM3	Pre-B-cell leukemia transcription factor 1 (Fragment)	PBX1		-0.145	
Q86WQ0	Nuclear receptor 2C2-associated protein	NR2C2AP		-0.145	
P61962	DDB1- and CUL4-associated factor 7	DCAF7		-0.141	
M0R2S2	Epidermal growth factor receptor substrate 15-like 1	EPS15L1		-0.141	
O96020-2	Isoform Short of G1/S-specific cyclin-E2	CCNE2		-0.140	
Q8NEV4	Myosin-IIIa	MYO3A		-0.139	
H0Y7Y8	AT-rich interactive domain-containing protein 4B (Fragment)	ARID4B		-0.139	
Q9Y546	Leucine-rich repeat-containing protein 42	LRRC42		-0.138	
Q9P013	Spliceosome-associated protein CWC15 homolog	CWC15		-0.138	
Q6Y2K3	INO80 complex subunit E	INO80E		-0.136	
H7C0D7	NADH dehydrogenase [ubiquinone] 1 alpha subcomplex subunit 3 (Fragment)	NDUFA3		-0.136	
Q03111	Protein ENL	MLLT1		-0.134	
P61968	LIM domain transcription factor LMO4	LMO4		-0.134	
E9PF46	Acylphosphatase	ACYP2		-0.133	
K7EMZ9	Protein LSM14 homolog A (Fragment)	LSM14A		-0.132	
Q9H939-2	Isoform 2 of Proline-serine-threonine phosphatase-interacting protein 2	PSTPIP2		-0.131	
Q15004	PCNA-associated factor	KIAA0101		-0.131	
Q9NU02	Ankyrin repeat and EF-hand domain-containing protein 1	ANKEF1		-0.131	
Q9GZT9-3	Isoform 3 of Egl nine homolog 1	EGLN1		-0.127	
H7C5S6	Leucine-rich repeat and WD repeat-containing protein 1 (Fragment)	LRWD1		-0.126	

P41229-4	Isoform 4 of Lysine-specific demethylase 5C	KDM5C		-0.124	
Q9NZI8	Insulin-like growth factor 2 mRNA-binding protein 1	IGF2BP1		-0.123	
Q5SXQ3	Procollagen galactosyltransferase 2	COLGALT2		-0.123	
Q12834	Cell division cycle protein 20 homolog	CDC20		-0.122	
Q9NZM3-4	Isoform 4 of Intersectin-2	ITSN2		-0.122	
F5H459	AP-3 complex subunit sigma-1	AP3S1		-0.120	
P43007	Neutral amino acid transporter A	SLC1A4		-0.119	
O95452	Gap junction beta-6 protein	GJB6		-0.118	
Q96BM9	ADP-ribosylation factor-like protein 8A	ARL8A		-0.117	
Q17RU2	Proto-oncogene c-Rel	REL		-0.117	
E5RK41	Pre-mRNA-splicing factor SLU7 (Fragment)	SLU7		-0.116	
Q5SW79-2	Isoform 2 of Centrosomal protein of 170 kDa	CEP170		-0.116	
I3L0U9	TBC1 domain family member 16 (Fragment)	TBC1D16		-0.116	
Q9Y4P8-3	Isoform 3 of WD repeat domain phosphoinositide-interacting protein 2	WIPI2		-0.116	
D6RAR7	Dysbindin (Fragment)	DTNBP1		-0.115	
Q9NYH9	U3 small nucleolar RNA-associated protein 6 homolog	UTP6		-0.115	
Q9H5V9-2	Isoform 2 of UPF0428 protein CXorf56	CXorf56		-0.115	
Q5VWP2	Protein FAM46C	FAM46C		-0.114	
E3W994	CLIP-associating protein 2	CLASP2		-0.113	
I3L4A2	Leucine carboxyl methyltransferase 1 (Fragment)	LCMT1		-0.111	
P15104	Glutamine synthetase	GLUL		-0.110	
Q96JM3	Chromosome alignment-maintaining phosphoprotein 1	CHAMP1		-0.108	
O15541	RING finger protein 113A	RNF113A		-0.106	
K7EN05	Transcription elongation factor 1 homolog	ELOF1		-0.105	
H0YE28	Selenoprotein H (Fragment)	C11orf31		-0.105	
Q8TF39	Zinc finger protein 483	ZNF483		-0.105	
H0YCZ7	Collagen alpha-1(XXIV) chain (Fragment)	COL24A1		-0.104	
C9JQE8	Nuclear receptor corepressor 2 (Fragment)	NCOR2		-0.104	
Q9NZ63	Uncharacterized protein C9orf78	C9orf78		-0.101	
Q6PJG2	ELM2 and SANT domain-containing protein 1	ELMSAN1		-0.100	
F8WC68	Decapping and exoribonuclease protein	DOM3Z		-0.099	
Q86UA6	RPA-interacting protein	RPAIN		-0.098	
Q2TB10	Zinc finger protein 800	ZNF800		-0.096	
Q99496	E3 ubiquitin-protein ligase RING2	RNF2		-0.096	
J3KQ72	F-box only protein 6 (Fragment)	FBXO6		-0.094	
B0QZ35	SirtT1 75 kDa fragment	SIRT1		-0.092	
P13498	Cytochrome b-245 light chain	CYBA		-0.092	
Q70CQ2-3	Isoform 3 of Ubiquitin carboxyl-terminal hydrolase 34	USP34		-0.092	
C9JTN7	Nucleolysin TIA-1 isoform p40	TIA1		-0.089	
O15121	Sphingolipid delta(4)-desaturase DES1	DEGS1		-0.088	
F8WE18	B-cell CLL/lymphoma 7 protein family member B	BCL7B		-0.087	
F8W6L4	RING finger and transmembrane domain-containing protein 2	RNFT2		-0.085	
B2R9C2	Family with sequence similarity 76, member B, isoform CRA_a	FAM76B		-0.084	
O14967	Calmegin	CLGN		-0.084	
Q13257	Mitotic spindle assembly checkpoint protein MAD2A	MAD2L1		-0.083	
E9PEW0	Ubiquitin carboxyl-terminal hydrolase 36	USP36		-0.080	
Q8IYS2	Uncharacterized protein KIAA2013	KIAA2013		-0.078	
Q02040	A-kinase anchor protein 17A	AKAP17A		-0.076	
Q9ULV3-5	Isoform 5 of Cip1-interacting zinc finger protein	CIZ1		-0.075	
E9PIW9	Atherin	SAMD1		-0.075	
Q7L523	Ras-related GTP-binding protein A	RRAGA		-0.074	
Q9BRR8	G patch domain-containing protein 1	GPATCH1		-0.074	
P05186	Alkaline phosphatase, tissue-nonspecific isozyme	ALPL		-0.073	
A6NDU8	UPF0600 protein C5orf51	C5orf51		-0.072	
P31146	Coronin-1A	CORO1A		-0.071	
Q6P3V2	Zinc finger protein 585A	ZNF585A		-0.070	
Q9ULJ3-2	Isoform 2 of Zinc finger and BTB domain-containing protein 21	ZBTB21		-0.069	
J3KS94	Myelin basic protein (Fragment)	MBP		-0.069	
Q13601-2	Isoform 2 of KRR1 small subunit processome component homolog	KRR1		-0.068	
Q8WZA0	Protein LZIC	LZIC		-0.066	
Q14781	Chromobox protein homolog 2	CBX2		-0.066	
B3KV95	F-box only protein 18	FBXO18		-0.066	
E7EP17	Dynein heavy chain 9, axonemal	DNAH9		-0.064	
O43414-3	Isoform 3 of ERI1 exoribonuclease 3	ERI3		-0.064	
Q5TBG2	Serine/threonine-protein kinase Nek6 (Fragment)	NEK6		-0.062	
M0R100	CD320 antigen (Fragment)	CD320		-0.062	
E9PF74	Mitochondrial thiamine pyrophosphate carrier	SLC25A19		-0.062	
I3L4I0	FLYWCH family member 2 (Fragment)	FLYWCH2		-0.061	
Q8IU81	Interferon regulatory factor 2-binding protein 1	IRF2BP1		-0.061	
Q9ULM3	YEATS domain-containing protein 2	YEATS2		-0.061	
I3L3E4	Charged multivesicular body protein 6 (Fragment)	CHMP6		-0.060	
E9PKT4	Porimin	TMEM123		-0.059	
Q96LW7-2	Isoform 2 of Bcl10-interacting CARD protein	C9orf89		-0.058	
Q9BZ95-3	Isoform 3 of Histone-lysine N-methyltransferase NSD3	WHSC1L1		-0.057	
Q9Y4U1	Methylmalonic aciduria and homocystinuria type C protein	MMACHC		-0.055	
P47224	Guanine nucleotide exchange factor MSS4	RABIF		-0.055	
F8WAF5	SOSS complex subunit B2	NABP1		-0.053	
K7EJ55	Zinc finger protein 440 (Fragment)	ZNF440		-0.052	
E9PJ43	Zinc finger C2HC domain-containing protein 1C (Fragment)	ZC2HC1C		-0.051	
P51531-2	Isoform Short of Probable global transcription activator SNF2L2	SMARCA2		-0.050	
Q09472	Histone acetyltransferase p300	EP300		-0.047	
E2J9M0	Truncated protein tyrosine phosphatase non-receptor type 14	PTPN14		-0.046	
Q969E8	Pre-rRNA-processing protein TSR2 homolog	TSR2		-0.046	
F8W1Z6	Protein MON2 homolog	MON2		-0.046	
C9JZL8	Myeloid-associated differentiation marker (Fragment)	MYADM		-0.044	
C9JXQ9	NADH dehydrogenase [ubiquinone] 1 beta subcomplex subunit 4	NDUFB4		-0.043	
Q8N5A5-3	Isoform 3 of Zinc finger CCCH-type with G patch domain-containing protein	ZGPAT		-0.042	

Q14050	Collagen alpha-3(IX) chain	COL9A3		-0.041	
J3KMW4	Centromere protein I	CENPI		-0.040	
Q92989	Polyribonucleotide 5'-hydroxyl-kinase Clp1	CLP1		-0.039	
Q8TBE9	N-acylneuraminate-9-phosphatase	NANP		-0.039	
Q8TCU6-2	Isoform 2 of Phosphatidylinositol 3,4,5-trisphosphate-dependent Rac exchanger 1	PREX1		-0.038	
P11171-6	Isoform 6 of Protein 4.1	EPB41		-0.037	
Q8N387-2	Isoform 2 of Mucin-15	MUC15		-0.036	
H3BPL4	Lysine-rich nucleolar protein 1 (Fragment)	KNOP1		-0.036	
A1X283	SH3 and PX domain-containing protein 2B	SH3PXD2B		-0.035	
F5H186	Testis-expressed sequence 40 protein	TEX40		-0.034	
Q6PIR0	CENPC1 protein	CENPC1		-0.034	
H0YIP2	DNA primase small subunit (Fragment)	PRIM1		-0.034	
Q8IU85-2	Isoform 2 of Calcium/calmodulin-dependent protein kinase type 1D	CAMK1D		-0.032	
B7Z2Y5	Ribosomal protein S6 kinase	RPS6KA5		-0.032	
H0YHP5	PRKC apoptosis WT1 regulator protein (Fragment)	PAWR		-0.031	
C9JX71	Apolipoprotein D (Fragment)	APOD		-0.030	
J3QRU4	Vesicle-associated membrane protein 2	VAMP2		-0.030	
C9JKA9	Kinesin-like protein KIF15	KIF15		-0.030	
Q5SZF2	Protein prune homolog (Fragment)	PRUNE		-0.028	
K7EJ32	Sphingosine kinase 1 (Fragment)	SPHK1		-0.027	
O75925	E3 SUMO-protein ligase PIAS1	PIAS1		-0.026	
E9PIF4	Sialidase-1	NEU1		-0.026	
P40763-2	Isoform Del-701 of Signal transducer and activator of transcription 3	STAT3		-0.026	
Q96BY7	Autophagy-related protein 2 homolog B	ATG2B		-0.026	
E5RJ99	AN1-type zinc finger protein 1	ZFAND1		-0.025	
Q9Y312	Protein AAR2 homolog	AAR2		-0.025	
Q9H7L9	Sin3 histone deacetylase corepressor complex component SDS3	SUDS3		-0.024	
D6R955	Pituitary homeobox 1 (Fragment)	PITX1		-0.023	
P61009	Signal peptidase complex subunit 3	SPCS3		-0.023	
P78358	Cancer/testis antigen 1	CTAG1A		-0.021	
Q9Y608	Leucine-rich repeat flightless-interacting protein 2	LRRFIP2		-0.021	
Q9H246	Uncharacterized protein C1orf21	C1orf21		-0.021	
Q9ULX6-2	Isoform 2 of A-kinase anchor protein 8-like	AKAP8L		-0.021	
A2A3M4	Cingulin (Fragment)	CGN		-0.019	
Q7Z3K3-5	Isoform 5 of Pogo transposable element with ZNF domain	POGZ		-0.019	
H7C117	Protein LOC728763 (Fragment)	LOC728763		-0.018	
H0YCI1	PHD finger protein 21A (Fragment)	PHF21A		-0.017	
Q9H3Q1	Cdc42 effector protein 4	CDC42EP4		-0.017	
C9JMV0	Uncharacterized protein C20orf26 (Fragment)	C20orf26		-0.011	
Q9Y6X9-2	Isoform 2 of MORC family CW-type zinc finger protein 2	MORC2		-0.010	
G5E9I6	Microtubule-associated protein RP/EB family member 2	MAPRE2		-0.008	
K7EPK6	CBP80/20-dependent translation initiation factor (Fragment)	CTIF		-0.007	
O75369-8	Isoform 8 of Filamin-B	FLNB		-0.007	
I3L412	Beta-arrestin-2 (Fragment)	ARRB2		-0.007	
Q9UBK9	Protein UXT	UXT		-0.005	
Q70Z53-2	Isoform 2 of Protein FRA10AC1	FRA10AC1		-0.004	
Q9H9Q2-2	Isoform 2 of COP9 signalosome complex subunit 7b	COPS7B		-0.003	
Q9P0U1	Mitochondrial import receptor subunit TOM7 homolog	TOMM7		-0.001	
Q9Y383	Putative RNA-binding protein Luc7-like 2	LUC7L2		0.000	
Q96RU3-4	Isoform 4 of Formin-binding protein 1	FNBP1		0.001	
M0QZC9	Long-chain fatty acid transport protein 1 (Fragment)	SLC27A1		0.002	
Q9H6X2	Anthrax toxin receptor 1	ANTXR1		0.002	
Q5T8Z6	Polycomb complex protein BMI-1 (Fragment)	BMI1		0.002	
E9PM37	Forkhead box protein K2 (Fragment)	FOXK2		0.003	
Q14181	DNA polymerase alpha subunit B	POLA2		0.003	
Q5EBL4-3	Isoform 3 of RILP-like protein 1	RILPL1		0.003	
Q9Y608-2	Isoform 2 of Leucine-rich repeat flightless-interacting protein 2	LRRFIP2		0.003	
Q5SYT8	Protein NAMPTL (Fragment)	NAMPTL		0.004	
P03928	ATP synthase protein 8	MT-ATP8		0.004	
Q9H3Y8	Pancreatic progenitor cell differentiation and proliferation factor	PPDPF		0.004	
K7EPR7	Ubiquitin-conjugating enzyme E2 G1 (Fragment)	UBE2G1		0.004	
P57772	Selenocysteine-specific elongation factor	EEFSEC		0.005	
Q9C040	Tripartite motif-containing protein 2	TRIM2		0.005	
Q9UL26	Ras-related protein Rab-22A	RAB22A		0.006	
O75177-2	Isoform 2 of Calcium-responsive transactivator	SS18L1		0.006	
Q8WWX9	Selenoprotein M	SELM		0.008	
O75638	Cancer/testis antigen 2	CTAG2		0.009	
Q96A70-3	Isoform 3 of Arginine decarboxylase	ADC		0.010	
H7C048	SWI/SNF-related matrix-associated actin-dependent regulator of chromatin subfar	SMARCE1		0.010	
Q8WXX5	DnaJ homolog subfamily C member 9	DNAJC9		0.011	
K7ES23	Lysine-specific demethylase 4B (Fragment)	KDM4B		0.012	
P62745	Rho-related GTP-binding protein RhoB	RHOB		0.012	
O60675	Transcription factor MafK	MAFK		0.014	
G3XAJ1	Deleted in leukemia 8 protein, isoform CRA_b	RNASEH2B		0.015	
O15164-2	Isoform Short of Transcription intermediary factor 1-alpha	TRIM24		0.016	
H7C3T5	Arf-GAP with GTPase, ANK repeat and PH domain-containing protein 1 (Fragment)	AGAP1		0.018	
Q12873-2	Isoform 2 of Chromodomain-helicase-DNA-binding protein 3	CHD3		0.018	
I3L4D5	B-cell CLL/lymphoma 7 protein family member C	BCL7C		0.018	
A0JLT2-2	Isoform 2 of Mediator of RNA polymerase II transcription subunit 19	MED19		0.019	
H7C529	PX domain-containing protein kinase-like protein (Fragment)	PXK		0.020	
Q9BVI4	Nucleolar complex protein 4 homolog	NOC4L		0.021	
Q13459-2	Isoform Short of Unconventional myosin-IXb	MYO9B		0.021	
Q9NZZ3-2	Isoform 2 of Charged multivesicular body protein 5	CHMP5		0.022	
B7Z2W5	Ral guanine nucleotide dissociation stimulator-like 1	RGL1		0.023	
Q7Z7H5	Transmembrane emp24 domain-containing protein 4	TMED4		0.023	
Q9H875	PRKR-interacting protein 1	PRKRIP1		0.023	
F8W1Q3	Biotinidase	BTD		0.023	

Q96P16	Regulation of nuclear pre-mRNA domain-containing protein 1A	RPRD1A		0.023	
Q9GZU2-4	Isoform 4 of Paternally-expressed gene 3 protein	PEG3		0.023	
F5H785	Trafficking protein particle complex subunit 2	TRAPPC2		0.025	
Q92917	G patch domain and KOW motifs-containing protein	GPKOW		0.025	
C9JAY7	Autophagy-related protein 16-1 (Fragment)	ATG16L1		0.025	
M0R175	Nuclear receptor subfamily 2 group F member 6 (Fragment)	NR2F6		0.027	
Q8N3C7	CAP-Gly domain-containing linker protein 4	CLIP4		0.027	
Q92994-9	Isoform 9 of Transcription factor IIIB 90 kDa subunit	BRF1		0.028	
H7C2S1	Guanine nucleotide exchange factor DBS (Fragment)	MCF2L		0.028	
G3V2B8	C-1-tetrahydrofolate synthase, cytoplasmic	MTHFD1		0.029	
Q8NGR8	Olfactory receptor 1L8	OR1L8		0.030	
B5MC98	Prolactin regulatory element-binding protein	PREB		0.030	
F5H1X6	Mitogen-activated protein kinase kinase kinase 4	MAP3K4		0.031	
M0QXT0	Upstream stimulatory factor 2	USF2		0.031	
H0Y5B0	Band 4.1-like protein 2 (Fragment)	EPB41L2		0.031	
Q9H1B7	Interferon regulatory factor 2-binding protein-like	IRF2BPL		0.032	
Q9H6W3-2	Isoform 2 of Bifunctional lysine-specific demethylase and histidyl-hydroxylase NO6	NO66		0.033	
Q14202-3	Isoform 3 of Zinc finger MYM-type protein 3	ZMYM3		0.034	
Q7L4I2-2	Isoform 2 of Arginine/serine-rich coiled-coil protein 2	RSRC2		0.034	
P03891	NADH-ubiquinone oxidoreductase chain 2	MT-ND2		0.034	
Q15750-2	Isoform 2 of TGF-beta-activated kinase 1 and MAP3K7-binding protein 1	TAB1		0.035	
Q3ZCW2	Galectin-related protein	LGALSL		0.036	
O15111	Inhibitor of nuclear factor kappa-B kinase subunit alpha	CHUK		0.037	
Q32P28	Prolyl 3-hydroxylase 1	LEPRE1		0.037	
O95429-2	Isoform 2 of BAG family molecular chaperone regulator 4	BAG4		0.038	
Q9UHD2	Serine/threonine-protein kinase TBK1	TBK1		0.038	
Q6IBW4-2	Isoform 2 of Condensin-2 complex subunit H2	NCAPH2		0.038	
P83876	Thioredoxin-like protein 4A	TXNL4A		0.039	
Q9H0U3	Magnesium transporter protein 1	MAGT1		0.040	
Q7Z392-4	Isoform 4 of Trafficking protein particle complex subunit 11	TRAPPC11		0.041	
Q9UHV5-2	Isoform 2 of Rap guanine nucleotide exchange factor-like 1	RAPGEFL1		0.043	
B5MCS1	NAD-dependent protein deacetylase sirtuin-2 (Fragment)	SIRT2		0.043	
O75175-2	Isoform 2 of CCR4-NOT transcription complex subunit 3	CNOT3		0.043	
P98160	Basement membrane-specific heparan sulfate proteoglycan core protein	HSPG2		0.044	
Q9Y679-2	Isoform Short of Ancient ubiquitous protein 1	AUP1		0.045	
F5H1R9	Alpha-parvin	PARVA		0.045	
P28749-2	Isoform 2 of Retinoblastoma-like protein 1	RBL1		0.046	
Q68EM7-2	Isoform 2 of Rho GTPase-activating protein 17	ARHGAP17		0.046	
H0Y750	Renin receptor (Fragment)	ATP6AP2		0.046	
O43852-2	Isoform 2 of Calumenin	CALU		0.047	
C9J2A2	Myotubularin (Fragment)	MTM1		0.049	
E9PSH3	Tetraspanin-4 (Fragment)	TSPAN4		0.049	
Q9NZJ4-2	Isoform 2 of Sacsin	SACS		0.051	
C9JZ85	NF-kappa-B essential modulator (Fragment)	IKBKG		0.051	
E9PJB8	Selenoprotein S	VIMP		0.051	
Q15067	Peroxisomal acyl-coenzyme A oxidase 1	ACOX1		0.051	
Q8N806	Putative E3 ubiquitin-protein ligase UBR7	UBR7		0.052	
F5H4S9	Tight junction protein ZO-3	TJP3		0.053	
B1AKP2	Tetraspanin-2 (Fragment)	TSPAN2		0.053	
Q9P021	Cysteine-rich PDZ-binding protein	CRIPT		0.054	
Q5RI15	Cytochrome c oxidase protein 20 homolog	COX20		0.054	
K7EIU8	Mothers against decapentaplegic homolog 4	SMAD4		0.055	
O94875-9	Isoform 9 of Sorbin and SH3 domain-containing protein 2	SORBS2		0.055	
H0Y3K3	RNA-binding protein 33 (Fragment)	RBM33		0.056	
F8WF27	Transmembrane 4 L6 family member 1	TM4SF1		0.057	
Q96TA2-3	Isoform 3 of ATP-dependent zinc metalloprotease YME1L1	YME1L1		0.057	
Q5JY01	tRNA (guanine(10)-N2)-methyltransferase homolog (Fragment)	TRMT11		0.062	
F8W938	Nuclear valosin-containing protein-like	NVL		0.062	
B4DQ76	Kaptin	KPTN		0.063	
Q96Q89-5	Isoform 5 of Kinesin-like protein KIF20B	KIF20B		0.063	
O95396	Adenylyltransferase and sulfurtransferase MOCS3	MOCS3		0.063	
Q8N0T1	Uncharacterized protein C8orf59	C8orf59		0.065	
Q9NYZ3	G2 and S phase-expressed protein 1	GTSE1		0.066	
H7C3H1	Transcriptional repressor p66-alpha (Fragment)	GATAD2A		0.066	
C9J795	Poliovirus receptor-related protein 3 (Fragment)	PVRL3		0.068	
F8WE45	Leucine-rich repeat-containing protein 7	LRRC7		0.069	
K7EJW7	Ras-related protein Rab-34, isoform NARR (Fragment)	RAB34		0.069	
P22830	Ferrochelatase, mitochondrial	FECH		0.070	
H0Y7V4	Dynein heavy chain 8, axonemal	DNAH8		0.071	
Q9H6E4	Coiled-coil domain-containing protein 134	CCDC134		0.071	
P78362	SRSF protein kinase 2	SRPK2		0.072	
F8WC39	Glycerol kinase	GK		0.073	
F8W8W7	Trans-Golgi network integral membrane protein 2	TGOLN2		0.074	
Q92889	DNA repair endonuclease XPF	ERCC4		0.074	
Q8N490-2	Isoform 2 of Probable hydrolase PNKD	PNKD		0.075	
H0YB59	Tyrosine-protein phosphatase non-receptor type 12 (Fragment)	PTPN12		0.075	
Q58FF7	Putative heat shock protein HSP 90-beta-3	HSP90AB3P		0.077	
O60563	Cyclin-T1	CCNT1		0.078	
O60934	Nibrin	NBN		0.078	
Q969P0-3	Isoform 3 of Immunoglobulin superfamily member 8	IGSF8		0.079	
J3KND9	Electron transfer flavoprotein-ubiquinone oxidoreductase, mitochondrial	ETFDH		0.079	
O75052	Carboxyl-terminal PDZ ligand of neuronal nitric oxide synthase protein	NOS1AP		0.079	
E9PG22	Centrosomal protein of 97 kDa	CEP97		0.079	
P43121	Cell surface glycoprotein MUC18	MCAM		0.080	
P51809	Vesicle-associated membrane protein 7	VAMP7		0.080	
Q9BZE9	Tether containing UBX domain for GLUT4	ASPSCR1		0.081	
M0R3G1	Uncharacterized protein (Fragment)	4		0.081	

Q9BQB6-3	Isoform 3 of Vitamin K epoxide reductase complex subunit 1	VKORC1		0.082	
P07711	Cathepsin L1	CTSL1		0.082	
I3L427	Protein FAM64A	FAM64A		0.083	
Q5VWJ9	Sorting nexin-30	SNX30		0.083	
K7EIE8	Methyl-CpG binding domain protein 3, isoform CRA_b	MBD3		0.084	
H7C1I7	Zinc finger MYM-type protein 4 (Fragment)	ZMYM4		0.085	
F8WAN4	FERM domain-containing protein 4A	FRMD4A		0.085	
Q6P587	Acylpyruvase FAHD1, mitochondrial	FAHD1		0.086	
O43150-2	Isoform 2 of Arf-GAP with SH3 domain, ANK repeat and PH domain-containing pro	ASAP2		0.086	
Q96K76-2	Isoform 2 of Ubiquitin carboxyl-terminal hydrolase 47	USP47		0.086	
Q9BRR6-4	Isoform 4 of ADP-dependent glucokinase	ADPGK		0.086	
Q9NYB0	Telomeric repeat-binding factor 2-interacting protein 1	TERF2IP		0.086	
Q9UII4	E3 ISG15--protein ligase HERC5	HERC5		0.087	
Q8IY95-2	Isoform 2 of Transmembrane protein 192	TMEM192		0.089	
Q16533	snRNA-activating protein complex subunit 1	SNAPC1		0.089	
B4E0W6	TATA-binding protein-associated factor 172	BTAF1		0.090	
H0Y7I6	Bromodomain and PHD finger-containing protein 3 (Fragment)	BRPF3		0.090	
Q9UJV9	Probable ATP-dependent RNA helicase DDX41	DDX41		0.093	
O14920	Inhibitor of nuclear factor kappa-B kinase subunit beta	IKBKB		0.093	
B5MC96	HCLS1-binding protein 3	HS1BP3		0.095	
Q8IX01-4	Isoform 4 of SURP and G-patch domain-containing protein 2	SUGP2		0.096	
I3L480	NAD-dependent protein deacetylase sirtuin-7 (Fragment)	SIRT7		0.097	
Q8WY54-2	Isoform 2 of Protein phosphatase 1E	PPM1E		0.099	
O60725	Protein-S-isoprenylcysteine O-methyltransferase	ICMT		0.099	
Q9BXV9	Uncharacterized protein C14orf142	C14orf142		0.100	
O43379	WD repeat-containing protein 62	WDR62		0.101	
H0YEW4	ATP synthase mitochondrial F1 complex assembly factor 1 (Fragment)	ATPAF1		0.101	
Q9NUW8	Tyrosyl-DNA phosphodiesterase 1	TDP1		0.102	
Q9BRL6-2	Isoform 2 of Serine/arginine-rich splicing factor 8	SRSF8		0.103	
O43709	Uncharacterized methyltransferase WBSCR22	WBSCR22		0.103	
Q9HAN9	Nicotinamide mononucleotide adenylyltransferase 1	NMNAT1		0.104	
E9PLD3	Uncharacterized protein	4		0.104	
B1APE1	Transcription initiation factor IIB (Fragment)	GTF2B		0.105	
O43490-2	Isoform 2 of Prominin-1	PROM1		0.106	
K7ENL3	Signal transducer and activator of transcription 3	STAT3		0.106	
O95182	NADH dehydrogenase [ubiquinone] 1 alpha subcomplex subunit 7	NDUFA7		0.106	
Q3V6T2-5	Isoform 5 of Girdin	CCDC88A		0.107	
B8ZZ75	Aldose 1-epimerase	GALM		0.107	
I3L4X7	G protein pathway suppressor 2 (Fragment)	GPS2		0.108	
Q9HBM6	Transcription initiation factor TFIID subunit 9B	TAF9B		0.113	
H0YAY4	AP-3 complex subunit mu-2 (Fragment)	AP3M2		0.115	
E9PRK7	ARL14 effector protein (Fragment)	ARL14EP		0.115	
H0YIX3	Zinc finger protein 385A (Fragment)	ZNF385A		0.116	
Q9H617	Putative uncharacterized protein RP13-360B22.2	TMEM164		0.118	
P84095	Rho-related GTP-binding protein RhoG	RHOG		0.118	
P23297	Protein S100-A1	S100A1		0.118	
P17252	Protein kinase C alpha type	PRKCA		0.118	
Q5JUP3	Allograft inflammatory factor 1-like	AIF1L		0.123	
Q9Y2T7	Y-box-binding protein 2	YBX2		0.123	
Q6PK04	Coiled-coil domain-containing protein 137	CCDC137		0.123	
J3KSG3	ADP-ribosylation factor-binding protein GGA3 (Fragment)	GGA3		0.124	
Q9Y646	Carboxypeptidase Q	CPQ		0.127	
O00291-3	Isoform 3 of Huntingtin-interacting protein 1	HIP1		0.127	
F8W7M9	Fibulin-1	FBLN1		0.128	
Q6UB98-2	Isoform 2 of Ankyrin repeat domain-containing protein 12	ANKRD12		0.128	
Q9UHR5-2	Isoform 2 of SAP30-binding protein	SAP30BP		0.131	
Q5W006	Interleukin-2 receptor subunit alpha	IL2RA		0.131	
F5H3Y4	Probable ATP-dependent RNA helicase DHX37	DHX37		0.131	
M0R2E1	Protein GPR108 (Fragment)	GPR108		0.131	
Q08431-3	Isoform 3 of Lactadherin	MFGE8		0.132	
G3XAE9	KIAA0423, isoform CRA_a	FAM179B		0.133	
C9JVD5	Cysteine protease ATG4B (Fragment)	ATG4B		0.133	
P10398	Serine/threonine-protein kinase A-Raf	ARAF		0.133	
Q9UBF8-3	Isoform 3 of Phosphatidylinositol 4-kinase beta	PI4KB		0.134	
Q7Z478	ATP-dependent RNA helicase DHX29	DHX29		0.134	
B4DYL1	Coiled-coil domain-containing protein KIAA1407	KIAA1407		0.134	
Q5XKP0	Protein QIL1	QIL1		0.138	
Q9NZU5	LIM and cysteine-rich domains protein 1	LMCD1		0.141	
P51153	Ras-related protein Rab-13	RAB13		0.142	
B7Z4K6	Deoxyribonuclease-2-alpha	DNASE2		0.142	
K7EQ93	RING finger protein unkempt homolog (Fragment)	UNK		0.143	
Q9BTX3-2	Isoform 2 of Transmembrane protein 208	TMEM208		0.143	
Q9BWH2	FUN14 domain-containing protein 2	FUNDC2		0.144	
B4DVQ6	Tetratricopeptide repeat protein 39B	TTC39B		0.145	
H0YIT1	NADH dehydrogenase [ubiquinone] 1 alpha subcomplex subunit 12 (Fragment)	NDUFA12		0.145	
Q9UMN6	Histone-lysine N-methyltransferase MLL4	WBP7		0.148	
D6RGD1	Unconventional myosin-X (Fragment)	MYO10		0.149	
K7ES57	UNC93-like protein MFSD11 (Fragment)	MFSD11		0.149	
Q8TET4	Neutral alpha-glucosidase C	GANC		0.151	
O94886	Transmembrane protein 63A	TMEM63A		0.152	
Q2M389-2	Isoform 2 of WASH complex subunit 7	KIAA1033		0.153	
B4DGW0	E3 SUMO-protein ligase PIAS2	PIAS2		0.156	
H0Y8S5	Vacuolar-sorting protein SNF8 (Fragment)	SNF8		0.157	
A6NP52	PRA1 family protein 2	PRAF2		0.157	
P54274-2	Isoform 2 of Telomeric repeat-binding factor 1	TERF1		0.157	
J3KSY3	Conserved oligomeric Golgi complex subunit 1 (Fragment)	COG1		0.159	
P19387	DNA-directed RNA polymerase II subunit RPB3	POLR2C		0.159	

H0Y9I3	Exportin-5 (Fragment)	XPO5		0.160	
E9PCV0	Beta-glucuronidase	GUSB		0.163	
Q9HAU5-2	Isoform 2 of Regulator of nonsense transcripts 2	UPF2		0.164	
H0YMP3	G2/mitotic-specific cyclin-B2	CCNB2		0.167	
Q9Y5A7-2	Isoform 2 of NEDD8 ultimate buster 1	NUB1		0.169	
H0YE51	Methionine-R-sulfoxide reductase B2, mitochondrial (Fragment)	MSRB2		0.169	
P31323	cAMP-dependent protein kinase type II-beta regulatory subunit	PRKAR2B		0.170	
Q9H9G7	Protein argonaute-3	AGO3		0.172	
K7ERQ0	Protein YIF1B (Fragment)	YIF1B		0.173	
Q5R3B4	Mitochondrial pyruvate carrier 2 (Fragment)	MPC2		0.175	
C9IY79	Phospholipase D1 (Fragment)	PLD1		0.178	
Q9NRK6	ATP-binding cassette sub-family B member 10, mitochondrial	ABCB10		0.179	
Q9UHL4	Dipeptidyl peptidase 2	DPP7		0.179	
O43677	NADH dehydrogenase [ubiquinone] 1 subunit C1, mitochondrial	NDUFC1		0.180	
P08572	Collagen alpha-2(IV) chain	COL4A2		0.180	
Q8TAF3-4	Isoform 4 of WD repeat-containing protein 48	WDR48		0.182	
Q8WUU5	GATA zinc finger domain-containing protein 1	GATAD1		0.182	
Q15599-2	Isoform 2 of Na(+)/H(+) exchange regulatory cofactor NHE-RF2	SLC9A3R2		0.182	
G3V1B6	Apolipoprotein O	APOO		0.184	
J3QLK5	Serine/threonine-protein kinase tousled-like 2	TLK2		0.184	
Q96S90-2	Isoform 2 of LysM and putative peptidoglycan-binding domain-containing protein	LYSMD1		0.185	
Q96ME7-2	Isoform 2 of Zinc finger protein 512	ZNF512		0.186	
Q8IYL9	Psychosine receptor	GPR65		0.187	
P53794	Sodium/myo-inositol cotransporter	SLC5A3		0.187	
C9JX82	Ankyrin repeat domain-containing protein 54 (Fragment)	ANKRD54		0.188	
B7Z5N5	Mothers against decapentaplegic homolog 2	SMAD2		0.190	
Q53ET0	CREB-regulated transcription coactivator 2	CRTC2		0.191	
Q6ZRH9	Uncharacterized protein FLJ46347	2		0.199	
Q9Y5B0-3	Isoform 3 of RNA polymerase II subunit A C-terminal domain phosphatase	CTDP1		0.203	
Q9NX14	NADH dehydrogenase [ubiquinone] 1 beta subcomplex subunit 11, mitochondrial	NDUFB11		0.204	
Q9UDT6-2	Isoform 2 of CAP-Gly domain-containing linker protein 2	CLIP2		0.204	
Q9BRT3	Migration and invasion enhancer 1	MIEN1		0.208	
H3BR31	Non-structural maintenance of chromosomes element 1 homolog	NSMCE1		0.210	
E9PBA8	Enhancer of polycomb homolog 2	EPC2		0.210	
Q14249	Endonuclease G, mitochondrial	ENDOG		0.217	
Q15072	Zinc finger protein OZF	ZNF146		0.219	
Q71U36	Tubulin alpha-1A chain	TUBA1A		0.220	
Q96PP9	Guanylate-binding protein 4	GBP4		0.221	
H3BUS7	UPF0505 protein C16orf62 (Fragment)	C16orf62		0.221	
Q8TDW0	Leucine-rich repeat-containing protein 8C	LRRC8C		0.227	
O94875-3	Isoform 3 of Sorbin and SH3 domain-containing protein 2	SORBS2		0.227	
Q86V85	Integral membrane protein GPR180	GPR180		0.229	
Q9H313-5	Isoform 5 of Protein tweety homolog 1	TTYH1		0.230	
Q86VW1-2	Isoform 2 of Solute carrier family 22 member 16	SLC22A16		0.232	
C9JV49	Probable histidine--tRNA ligase, mitochondrial	HARS2		0.233	
O60503	Adenylate cyclase type 9	ADCY9		0.237	
O00329	Phosphatidylinositol 4,5-bisphosphate 3-kinase catalytic subunit delta isoform	PIK3CD		0.237	
O75410-7	Isoform 7 of Transforming acidic coiled-coil-containing protein 1	TACC1		0.237	
Q13938	Calcyphosin	CAPS		0.238	
B5MCB5	Cellular retinoic acid-binding protein 1 (Fragment)	CRABP1		0.238	
P55268	Laminin subunit beta-2	LAMB2		0.242	
Q9UBF1	Melanoma-associated antigen C2	MAGEC2		0.244	
Q8NAT1	Glycosyltransferase-like domain-containing protein 2	GTDC2		0.245	
Q6UWJ1-3	Isoform 3 of Transmembrane and coiled-coil domain-containing protein 3	TMCO3		0.246	
P43155-3	Isoform 3 of Carnitine O-acetyltransferase	CRAT		0.250	
Q6GQQ9-2	Isoform 2 of OTU domain-containing protein 7B	OTUD7B		0.254	
Q96S94	Cyclin-L2	CCNL2		0.254	
H7BY83	WD repeat-containing protein 44 (Fragment)	WDR44		0.260	
B1AK45	Mitotic spindle assembly checkpoint protein MAD2B (Fragment)	MAD2L2		0.263	
Q9BXJ4-2	Isoform 2 of Complement C1q tumor necrosis factor-related protein 3	C1QTNF3		0.264	
B5MDQ0	DNA excision repair protein ERCC-6-like	ERCC6L		0.265	
Q63HK3-2	Isoform 2 of Zinc finger protein with KRAB and SCAN domains 2	ZKSCAN2		0.265	
C9JRX2	Kelch domain-containing protein 10	KLHDC10		0.265	
Q9NVR0	Kelch-like protein 11	KLHL11		0.266	
Q765P7	MTSS1-like protein	MTSS1L		0.273	
P0CJ78	Zinc finger protein 865	ZNF865		0.277	
J3KMY4	E3 ubiquitin-protein ligase E3D	UBE3D		0.277	
E7ET89	Probable E3 ubiquitin-protein ligase DTX2	DTX2		0.277	
B1AJQ6	Syntaxin-12 (Fragment)	STX12		0.278	
A4D1S0-2	Isoform 2 of Killer cell lectin-like receptor subfamily G member 2	KLRG2		0.279	
Q6SZW1-2	Isoform 2 of Sterile alpha and TIR motif-containing protein 1	SARM1		0.280	
F2Z3J4	Torsin-3A	TOR3A		0.281	
B1APR7	Eyes absent homolog 3	EYA3		0.281	
G3V1U0	Activating transcription factor 7 interacting protein, isoform CRA_f	ATF7IP		0.292	
F8W038	Chromatin complexes subunit BAP18	C17orf49		0.294	
Q8TAV0-2	Isoform 2 of Protein FAM76A	FAM76A		0.294	
H7C5R1	Ceruloplasmin (Fragment)	CP		0.312	
Q8WUB8-3	Isoform 3 of PHD finger protein 10	PHF10		0.313	
Q6ZWT7	Lysophospholipid acyltransferase 2	MBOAT2		0.313	
Q9NSU2-2	Isoform 2 of Three prime repair exonuclease 1	TREX1		0.313	
Q9H1K0	Rabenosyn-5	ZFYVE20		0.315	
Q8IU60-2	Isoform 2 of m7GpppN-mRNA hydrolase	DCP2		0.333	
O15439-2	Isoform 2 of Multidrug resistance-associated protein 4	ABCC4		0.334	
Q8IYQ7	Threonine synthase-like 1	THNSL1		0.336	
O75940	Survival of motor neuron-related-splicing factor 30	SMNDC1		0.336	
E9PFP1	Phosphatidylinositol 3-kinase regulatory subunit beta	PIK3R2		0.338	
Q8NHP6-2	Isoform 2 of Motile sperm domain-containing protein 2	MOSPD2		0.342	

Q08722-2	Isoform OA3-293 of Leukocyte surface antigen CD47	CD47		0.343	
C9JXP4	Zinc finger BED domain-containing protein 1 (Fragment)	ZBED1		0.354	
H0YC12	Integrator complex subunit 8 (Fragment)	INTS8		0.356	
Q8TB72-2	Isoform 2 of Pumilio homolog 2	PUM2		0.358	
Q9NYQ8	Protocadherin Fat 2	FAT2		0.362	
Q9UK22	F-box only protein 2	FBXO2		0.363	
P06213-2	Isoform Short of Insulin receptor	INSR		0.374	
Q9UEY8-2	Isoform 1 of Gamma-adducin	ADD3		0.374	
H0YHD8	Cysteine-rich protein 2 (Fragment)	CRIP2		0.375	
E7EQU6	Transcription cofactor vestigial-like protein 4 (Fragment)	VGLL4		0.381	
Q69YL0	Uncharacterized protein DKFZp762I1415	4		0.390	
Q9NUL5	UPF0515 protein C19orf66	C19orf66		0.391	
Q86Y91-2	Isoform 2 of Kinesin-like protein KIF18B	KIF18B		0.412	
Q9UJY4	ADP-ribosylation factor-binding protein GGA2	GGA2		0.416	
Q5TD07	Ribosyldihydronicotinamide dehydrogenase [quinone]	NQO2		0.420	
H0YJH7	Thymopentin (Fragment)	TMPO		0.425	
Q02318	Sterol 26-hydroxylase, mitochondrial	CYP27A1		0.429	
O60462-4	Isoform B0 of Neuropilin-2	NRP2		0.438	
H0YF09	TRAF3-interacting JNK-activating modulator (Fragment)	TRAF3IP3		0.444	
Q5JSB5	Transcription factor Dp-1 (Fragment)	TFDP1		0.445	
Q9UBN7-2	Isoform 2 of Histone deacetylase 6	HDAC6		0.451	
P17342-2	Isoform 2 of Atrial natriuretic peptide receptor 3	NPR3		0.460	
P12277	Creatine kinase B-type	CKB		0.461	
C9JKQ2	NADH dehydrogenase [ubiquinone] 1 beta subcomplex subunit 3 (Fragment)	NDUFB3		0.464	
H7C1E5	Trafficking protein particle complex subunit 12 (Fragment)	TRAPPC12		0.472	
H0Y6D8	TATA-box-binding protein (Fragment)	TBP		0.494	
P30504	HLA class I histocompatibility antigen, Cw-4 alpha chain	HLA-C		0.497	
Q9UDT1	Rhomboid domain-containing protein 2	GSC:H_RG122E10.2a		0.502	
Q13946-3	Isoform PDE7A3 of High affinity cAMP-specific 3',5'-cyclic phosphodiesterase 7A	PDE7A		0.502	
F2Z2W8	Selenium-binding protein 1	SELENBP1		0.504	
F5GWS8	Heat shock 70 kDa protein 13	HSPA13		0.607	
G3V1T3	TAP binding protein-like, isoform CRA_c	TAPBPL		0.611	
C9JEV0	Zinc-alpha-2-glycoprotein	AZGP1		0.615	
P05091	Aldehyde dehydrogenase, mitochondrial	ALDH2		0.660	
Q6P275	Cohesin subunit SA-1	STAG1		0.666	
A2IDA3	DNA-3-methyladenine glycosylase (Fragment)	MPG		0.694	
P15941-13	Isoform 13 of Mucin-1	MUC1		0.702	
K7EJH8	Alpha-actinin-4 (Fragment)	ACTN4		0.714	
Q13310-2	Isoform 2 of Polyadenylate-binding protein 4	PABPC4		0.719	
E9PE24	Visinin-like protein 1 (Fragment)	VSNL1		0.873	
F5H1F6	Vacuolar protein sorting-associated protein 37B (Fragment)	VPS37B		1.249	
P06732	Creatine kinase M-type	CKM		1.356	
A6NGH7	Coiled-coil domain-containing protein 160	CCDC160			
B0S8I7	L antigen family member 3	LAGE3			
B0UZ83	Complement C4 beta chain	C4A			
B0V0C2	HLA class I histocompatibility antigen, Cw-14 alpha chain	HLA-C			
B1AMB2	Target of rapamycin complex 2 subunit MAPKAP1 (Fragment)	MAPKAP1			
B3KMG0	Nucleoporin GLE1	GLE1			
B3KQF6	Vacuolar protein sorting 33B (Yeast), isoform CRA_b	VPS33B			
B4DHD4	Integrin beta	ITGB8			
B4DSH5	SLIT and NTRK-like protein 5	SLITRK5			
B4DV96	Ribokinase	RBKS			
B4E0J7	RNA polymerase I-specific transcription initiation factor RRN3	RRN3			
B4E2M2	Protein FAM154B	FAM154B			
B7Z7N2	Conserved oligomeric Golgi complex subunit 2	COG2			
B8ZZG1	MAGUK p55 subfamily member 6	MPP6			
B8ZZR0	Dual-specificity protein kinase CLK1	CLK1			
C9J4W5	Eukaryotic translation initiation factor 5A-2 (Fragment)	EIF5A2			
C9J6A7	Ribulose-phosphate 3-epimerase (Fragment)	RPE			
C9JUJ0	CGG triplet repeat-binding protein 1 (Fragment)	CGGBP1			
C9JXC3	O-acetyl-ADP-ribose deacetylase 1	OARD1			
C9K0P5	Glycerol-3-phosphate dehydrogenase 1-like protein (Fragment)	GPD1L			
D6RCB7	DCN1-like protein 4	DCUN1D4			
E7ENC2	Regulating synaptic membrane exocytosis protein 1	RIMS1			
E7ENN3	Nesprin-1	SYNE1			
E7EPF2	RNA-binding motif, single-stranded-interacting protein 1 (Fragment)	RBMS1			
E9PG82	Sorcin	SRI			
E9PGR5	Ecto-ADP-ribosyltransferase 3 (Fragment)	ART3			
E9PHX8	Tyrosine-protein kinase Mer	MERTK			
E9PMR6	Rho guanine nucleotide exchange factor 12	ARHGEF12			
F5GXB3	DNA/RNA-binding protein KIN17	KIN			
F5H070	Lysine-specific demethylase 3A	KDM3A			
F5H2U8	High mobility group protein HMGI-C	HMGA2			
F5H553	Vacuolar fusion protein CCZ1 homolog	CCZ1B			
F5H5Q8	Coiled-coil domain-containing protein 77 (Fragment)	CCDC77			
F8WAI8	Zinc finger and BTB domain-containing protein 40	ZBTB40			
F8WEJ3	Phosphatidylinositol-glycan biosynthesis class X protein	PIGX			
G3V1N2	HCG1745306, isoform CRA_a	HBA2			
G3V1U5	Golgi transport 1 homolog B (S. cerevisiae), isoform CRA_c	GOLT1B			
G3V5W3	Son of sevenless homolog 2	SOS2			
G3XAE6	Extracellular sulfatase Sulf-2	SULF2			
H0Y4U8	Tuftelin-interacting protein 11 (Fragment)	TFIP11			
H0Y621	Endoplasmic reticulum-Golgi intermediate compartment protein 3 (Fragment)	ERGIC3			
H0Y6M2	FCH and double SH3 domains protein 2 (Fragment)	FCHSD2			
H0Y9M8	NADH dehydrogenase [ubiquinone] iron-sulfur protein 4, mitochondrial (Fragment	NDUFS4			
H0YDM2	C-terminal 80 kDa form (Fragment)	SOGA1			
H0YJ74	Bromodomain adjacent to zinc finger domain protein 1A (Fragment)	BAZ1A			

H0YKH0	Transducin-like enhancer protein 3	TLE3			
H0YNF6	TM2 domain-containing protein 3 (Fragment)	TM2D3			
H3BQ67	Lon protease homolog 2, peroxisomal (Fragment)	LONP2			
H3BS42	Zinc finger protein 768	ZNF768			
H3BV87	Armadillo repeat-containing X-linked protein 3 (Fragment)	ARMCX3			
H7BZ41	Lipoxygenase homology domain-containing protein 1	LOXHD1			
H7C0J5	Centrosomal protein of 104 kDa (Fragment)	CEP104			
H7C207	R3H domain-containing protein 1 (Fragment)	R3HDM1			
H7C3U4	Probable E3 ubiquitin-protein ligase MYCBP2 (Fragment)	MYCBP2			
H7C487	Oxysterol-binding protein (Fragment)	OSBPL10			
I3L170	Microtubule-associated protein	MAPT			
J3KR86	GRAM domain-containing protein 1A	GRAMD1A			
J3KRW7	Transmembrane protein 199	TMEM199			
J3QLA9	Zinc transporter ZIP11 (Fragment)	SLC39A11			
J3QQR8	Intercellular adhesion molecule 2 (Fragment)	ICAM2			
J3QSE8	39S ribosomal protein L36, mitochondrial	BRIP1			
K7EIJ6	Unconventional myosin-Vb (Fragment)	MYO5B			
O15320-9	Isoform 10 of cTAGE family member 5	CTAGE5			
O15392	Baculoviral IAP repeat-containing protein 5	BIRC5			
O75030-11	Isoform Mdel of Microphthalmia-associated transcription factor	MITF			
O76050-2	Isoform 2 of Neuralized-like protein 1A	NEURL			
O95382-3	Isoform 3 of Mitogen-activated protein kinase kinase kinase 6	MAP3K6			
O95822	Malonyl-CoA decarboxylase, mitochondrial	MLYCD			
P01009	Alpha-1-antitrypsin	SERPINA1			
P04114	Apolipoprotein B-100	APOB			
P04908	Histone H2A type 1-B/E	HIST1H2AB			
P06753	Tropomyosin alpha-3 chain	TPM3			
P0CG39	POTE ankyrin domain family member J	POTEJ			
P14543-2	Isoform 2 of Nidogen-1	NID1			
P16298-3	Isoform 3 of Serine/threonine-protein phosphatase 2B catalytic subunit beta isofor	PPP3CB			
P20648	Potassium-transporting ATPase alpha chain 1	ATP4A			
P22466	Galanin peptides	GAL			
P22670	MHC class II regulatory factor RFX1	RFX1			
P26374	Rab proteins geranylgeranyltransferase component A 2	CHML			
P29728	2'-5'-oligoadenylate synthase 2	OAS2			
P30464	HLA class I histocompatibility antigen, B-15 alpha chain	HLA-B			
P30466	HLA class I histocompatibility antigen, B-18 alpha chain	HLA-B			
P30479	HLA class I histocompatibility antigen, B-41 alpha chain	HLA-B			
P30480	HLA class I histocompatibility antigen, B-42 alpha chain	HLA-B			
P30487	HLA class I histocompatibility antigen, B-49 alpha chain	HLA-B			
P30490	HLA class I histocompatibility antigen, B-52 alpha chain	HLA-B			
P30491	HLA class I histocompatibility antigen, B-53 alpha chain	HLA-B			
P30495	HLA class I histocompatibility antigen, B-56 alpha chain	HLA-B			
P30501	HLA class I histocompatibility antigen, Cw-2 alpha chain	HLA-C			
P33947	ER lumen protein retaining receptor 2	KDELR2			
P35612-2	Isoform 2 of Beta-adducin	ADD2			
P35869	Aryl hydrocarbon receptor	AHR			
P48681	Nestin	NES			
P56378	6.8 kDa mitochondrial proteolipid	MP68			
P61073	C-X-C chemokine receptor type 4	CXCR4			
P62877	E3 ubiquitin-protein ligase RBX1	RBX1			
P78310-4	Isoform 4 of Coxsackievirus and adenovirus receptor	CXADR			
P81605	Dermcidin	DCD			
Q00613-2	Isoform Short of Heat shock factor protein 1	HSF1			
Q04721	Neurogenic locus notch homolog protein 2	NOTCH2			
Q07000	HLA class I histocompatibility antigen, Cw-15 alpha chain	HLA-C			
Q13395	Probable methyltransferase TARBP1	TARBP1			
Q13637	Ras-related protein Rab-32	RAB32			
Q14004-2	Isoform 2 of Cyclin-dependent kinase 13	CDK13			
Q14185	Dedicator of cytokinesis protein 1	DOCK1			
Q15067-3	Isoform 3 of Peroxisomal acyl-coenzyme A oxidase 1	ACOX1			
Q15776	Zinc finger protein with KRAB and SCAN domains 8	ZKSCAN8			
Q15847	Adipogenesis regulatory factor	ADIRF			
Q2M1P5	Kinesin-like protein KIF7	KIF7			
Q2PZI1	Probable C-mannosyltransferase DPY19L1	DPY19L1			
Q2YD98	UV-stimulated scaffold protein A	UVSSA			
Q3ZCM7	Tubulin beta-8 chain	TUBB8			
Q53SF7-4	Isoform 4 of Cordon-bleu protein-like 1	COBLL1			
Q5M9N0	Coiled-coil domain-containing protein 158	CCDC158			
Q5R3E6	Mitogen-activated protein kinase 13	MAPK13			
Q5SXH7-4	Isoform 4 of Pleckstrin homology domain-containing family S member 1	PLEKHS1			
Q5T6C5	Ataxin-7-like protein 2	ATXN7L2			
Q5XLC2	6-phosphofructo-2-kinase/fructose-2, 6-biphosphatase 4 splice isoform 5	PFKFB4			
Q6MZV4	Protein NOXP20	DKFZp686F20250			
Q6NUK4	Receptor expression-enhancing protein 3	REEP3			
Q6P161	39S ribosomal protein L54, mitochondrial	MRPL54			
Q6PIJ6-3	Isoform 3 of F-box only protein 38	FBXO38			
Q7Z3T8-3	Isoform 2 of Zinc finger FYVE domain-containing protein 16	ZFYVE16			
Q86SX3-4	Isoform 4 of Uncharacterized protein C14orf80	C14orf80			
Q8IY47	Kelch repeat and BTB domain-containing protein 2	KBTBD2			
Q8N184-2	Isoform 2 of Zinc finger protein 567	ZNF567			
Q8N427	Thioredoxin domain-containing protein 3	NME8			
Q8N4C8-5	Isoform 5 of Misshapen-like kinase 1	MINK1			
Q8N4V1	Membrane magnesium transporter 1	MMGT1			
Q8N8A6	ATP-dependent RNA helicase DDX51	DDX51			
Q8NB16	Mixed lineage kinase domain-like protein	MLKL			
Q8NCU4	Coiled-coil domain-containing protein KIAA1407	KIAA1407			

Q8NDH2	Coiled-coil domain-containing protein 168	CCDC168			
Q8NEF9	Serum response factor-binding protein 1	SRFBP1			
Q8TAP9	M-phase-specific PLK1-interacting protein	MPLKIP			
Q8TB22-4	Isoform 4 of Spermatogenesis-associated protein 20	SPATA20			
Q8TBZ6	tRNA methyltransferase 10 homolog A	TRMT10A			
Q8TE60-2	Isoform 2 of A disintegrin and metalloproteinase with thrombospondin motifs 18	ADAMTS18			
Q8WUH6	UPF0444 transmembrane protein C12orf23	C12orf23			
Q96A73-2	Isoform 2 of Putative monooxygenase p33MONOX	KIAA1191			
Q96MR6	WD repeat-containing protein 65	WDR65			
Q96S99	Pleckstrin homology domain-containing family F member 1	PLEKHF1			
Q96SY0-4	Isoform 4 of von Willebrand factor A domain-containing protein 9	VWA9			
Q9BQ04	RNA-binding protein 4B	RBM4B			
Q9BTT6	Leucine-rich repeat-containing protein 1	LRRC1			
Q9BV35-4	Isoform 4 of Calcium-binding mitochondrial carrier protein SCaMC-3	SLC25A23			
Q9C037-2	Isoform Beta of Tripartite motif-containing protein 4	TRIM4			
Q9C0F1	Centrosomal protein of 44 kDa	CEP44			
Q9C0I1-3	Isoform 3 of Myotubularin-related protein 12	MTMR12			
Q9H0E3-2	Isoform 2 of Histone deacetylase complex subunit SAP130	SAP130			
Q9H3N8-2	Isoform 2 of Histamine H4 receptor	HRH4			
Q9H7V2	Synapse differentiation-inducing gene protein 1	SYNDIG1			
Q9H9C1-2	Isoform 2 of Spermatogenesis-defective protein 39 homolog	VIPAS39			
Q9H9L3	Interferon-stimulated 20 kDa exonuclease-like 2	ISG20L2			
Q9HBD1-2	Isoform 2 of RING finger and CCCH-type zinc finger domain-containing protein 2	RC3H2			
Q9P1U0	DNA-directed RNA polymerase I subunit RPA12	ZNRD1			
Q9P2K3-4	Isoform 4 of REST corepressor 3	RCOR3			
Q9UBB5	Methyl-CpG-binding domain protein 2	MBD2			
Q9UET6-2	Isoform 2 of Putative tRNA (cytidine(32)/guanosine(34)-2'-O)-methyltransferase	FTSJ1			
Q9UHX1	Poly(U)-binding-splicing factor PUF60	PUF60			
Q9UKJ3-2	Isoform 2 of G patch domain-containing protein 8	GPATCH8			
Q9UPZ9	Serine/threonine-protein kinase ICK	ICK			
Q9Y252	E3 ubiquitin-protein ligase RNF6	RNF6			
Q9Y2H5	Pleckstrin homology domain-containing family A member 6	PLEKHA6			
Q9Y2K7-4	Isoform 4 of Lysine-specific demethylase 2A	KDM2A			
Q9Y3I1-3	Isoform 3 of F-box only protein 7	FBXO7			
R4GNG8	Zinc finger matrin-type protein 2 (Fragment)	ZMAT2			
